# Measures to monitor the implementation of Essential Health Benefit Packages at a national scale

**DOI:** 10.1038/s44401-026-00081-4

**Published:** 2026-04-29

**Authors:** Sali Ahmed, Jiashuo Sun, Yanjia Cao, David Watkins, Yanfang Su

**Affiliations:** 1 Department of Global Health, School of Public Health and School of Medicine, University of Washington, Seattle, WA USA; 2 Department of Geography, The University of Hong Kong, Hong Kong, China; 3 Division of General Internal Medicine, Department of Medicine, University of Washington, Seattle, WA USA; 4 Evans School of Public Policy and Governance, University of Washington, Seattle, WA USA

**Keywords:** Diseases, Health care, Medical research

## Abstract

Universal health coverage requires identifying who is covered by which services. However, weak health information systems hinder monitoring coverage of Essential Health Benefit Packages (EHBPs). We developed a method for measuring input-adjusted coverage, a potential coverage metric, combining geographic proximity with minimum-input facility readiness, using Malawi as a case study. We mapped the Disease Control Priorities Project interventions to the 2019 Harmonized Health Facility Assessment, constructing transparent input-based readiness indicators. We assessed readiness and generated readiness bottleneck cascades for 129 interventions in 563 publicly financed healthcare facilities. We conducted service area analysis, assessing national and district-level coverage within 5 and 25 km catchment areas based on health service level. Findings revealed high childhood vaccinations, HIV, and malaria management readiness and coverage compared to non-communicable diseases and surgical care. This method repurposes facility surveys and provides actionable insights on supply-side gaps for EHBP planning, resource allocation, and monitoring in low-resource settings.

## Introduction

Many countries have adopted Essential Health Benefits Packages (EHBPs) as a policy tool to optimize limited resources and achieve universal health coverage (UHC), yet their implementation remains limited^1–3^. To effectively achieve UHC, it is essential to identify who is covered and by which specific services^4^. The availability of reliable and high-quality information is crucial for evidence-informed policy development and effective implementation^5–9^. The lack of reliable coverage data hinders UHC efforts. Researchers and policymakers are often compelled to use proxy indicators and expert opinions to inform policy decisions, thereby increasing uncertainty in quantifying and allocating UHC investments^10^. Leveraging existing datasets to estimate coverage for a broad range of health services is crucial, particularly for health systems with limited resources to expand health information infrastructure^11^.

Weak national health information systems hinder tracking EHBP implementation^12^. Consequently, countries and global health partners often rely on household surveys, such as the Demographic and Health Surveys (DHS) and the Multiple Indicator Cluster Surveys (MICS), which focus on basic, high-value services for children, reproductive-age women, and some infectious diseases. While valuable for advocacy and global benchmarking, these surveys are less useful for country-level planning and lack the breadth necessary to comprehensively evaluate EHBP implementation^13–16^. Further, these surveys do not account for geographical accessibility, which can limit the number of patients who can utilize services^17^. Many studies use geographic proximity to healthcare facilities as a proxy for coverage; however, this does not guarantee service availability if the facilities are not ready to provide care^18^. The World Health Organization (WHO) Harmonized Health Facility Assessment (HHFA) survey is a comprehensive assessment of health service inputs across a wide range of health areas, including communicable and non-communicable diseases, reproductive health, and others^19^. As such, the HHFA provides an opportunity to track progress on coverage of specific high-priority health services.

Malawi has had an EHBP for over twenty years, but limited resources and a lack of enforcement challenge its implementation^20^. According to the Health Sector Strategic Plan II, in 2016, 75% of Malawi’s population lived within 8 km of a primary healthcare facility, which is considered covered^21^. Yet, 55.6% of women aged 15–49 considered distance a significant barrier to healthcare access^22^. Phiri et al. estimated that 62% of households in the Zomba district need to walk 60 min to the health facility^23^. However, the geographic proximity does not mean essential services can be accessed, especially if necessary inputs (e.g., drugs and diagnostics) are lacking^24,25^.

Achieving high coverage of an EHBP is critical to achieving UHC, so EHBP coverage must be characterized periodically to support evidence-informed policies and practices, including EHBP revision and implementation^26^. The extent of coverage of supply-side determinants is critical for identifying national and subnational priorities. The Effective Coverage Think Tank states that service contact, inputs, interventions, quality of care, and user adherence must be combined to assess effective coverage and identifies that health facility assessments are suitable for estimating input-adjusted coverage^27^. Our study aims to develop measures to monitor the national adoption and implementation of a broad number of health interventions that serve as the basis of EHBP across countries, using the findings of the 2019 HHFA in Malawi as a proof of concept for our method. We combine facility readiness assessment and spatial analysis to assess input-adjusted coverage, accounting for geographic coverage disparities. More specifically, our study aims to:

I. Explore the possible set of interventions tracked by the HHFA
II. Assess health service coverage within the publicly financed delivery system by synthesizing data from the 2019 HHFA survey and the spatial distribution of population estimates.

## Results

### Descriptive statistics

For the analysis, we mapped 280 interventions to the input availability and readiness questions in the HHFA 2018 core questionnaire. Of these, we assessed readiness and input-adjusted coverage for 129 health interventions; the remaining interventions were excluded due to the absence of related HHFA questions and are listed in Supplementary Data 1. Most of these interventions are provided at health centers and rural community hospitals. Table 1 summarizes the number of health interventions included in the analysis by health service level, catchment area, and composite indicator data.

**Table 1:** Number of health interventions and facilities included in the analysis

Health facility level		*n*	Interventions	Evaluated based on multiple components of care (*n* = 115)	Evaluated based on the availability of medicines only (*n* = 14)
	Catchment area (km, radius)				
All health facilities	5	563	Basic primary care	58	2
All hospitals (rural community hospital (*n* = 41), district hospital (*n* = 47), central hospital (*n* = 4))	5	92	Enhanced primary care	34	9
District hospital, central hospital	25	51	Secondary care	23	3

### Readiness bottleneck analysis

**Fig. 1:**
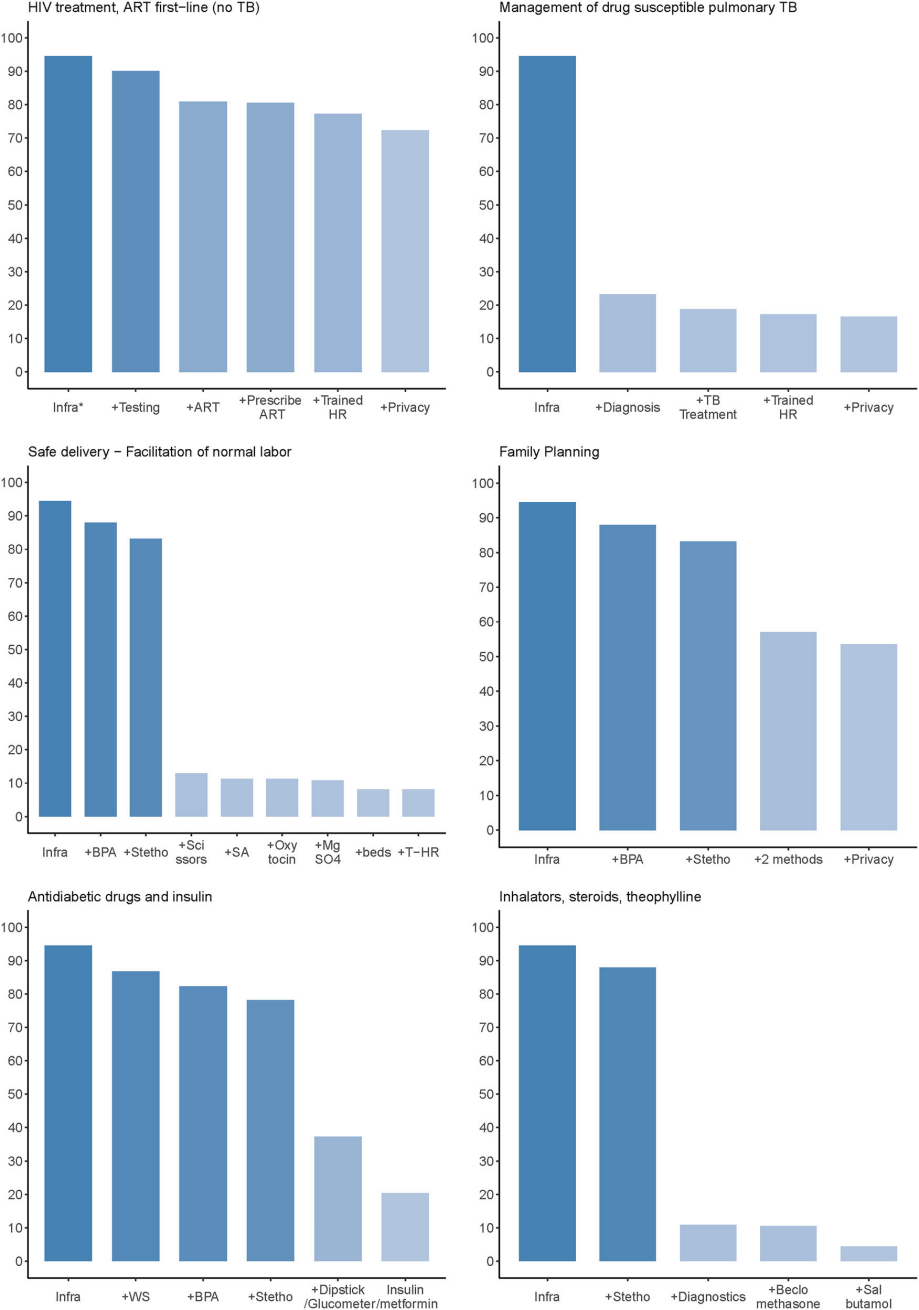
Readiness percentage by composite indicator component for selected interventions. Infra infrastructure, WS weighing scale, BPA blood pressure apparatus, Stetho Stethoscope, SA suction apparatus, T-HR trained human resource.

We identified supply-side inputs contributing to low readiness and coverage scores across various health interventions. Figure 1 illustrates readiness cascades, how the least available component of care drives the indicator variable for each intervention. For example, the most significant drop in readiness for the intervention called “safe delivery” occurred due to the low availability of surgical scissors. For the intervention called “management of type 2 diabetes,” two readiness drops are noticeable: first, when diagnostic tests were included, and second, when medication was added. Supplementary File 1 included bottleneck analysis for the 129 interventions included in the study.

### Readiness estimates by service categories

**Fig. 2:**
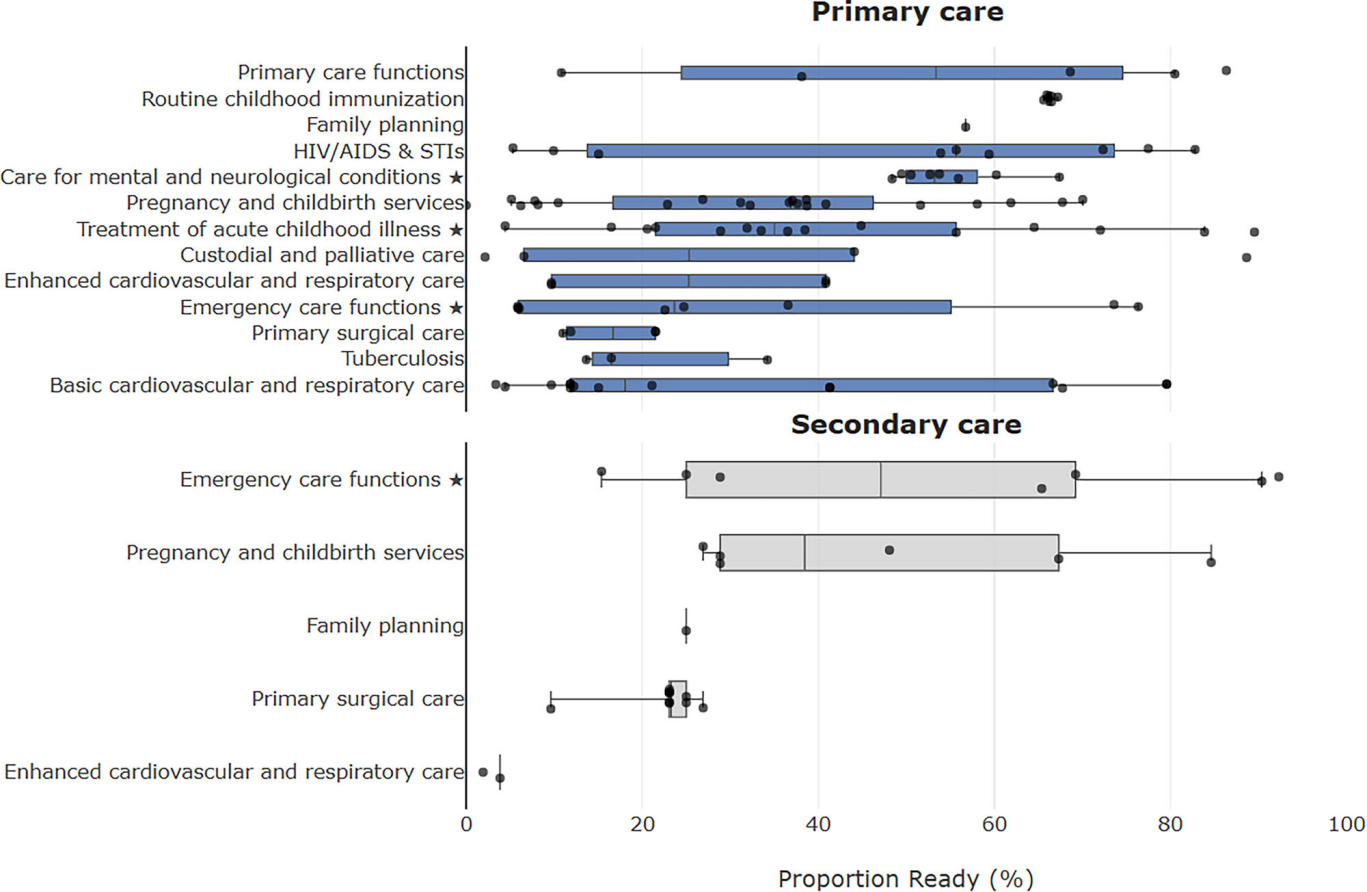
Readiness by service level. ★ Interventions assessed based on the availability of medicines only. The interactive version is available online at: https://salia25.github.io/Coverage_Malawi_HHFA/figure_4_facility_readiness_plot.html.

Figure 2 presents detailed facility readiness for 129 health interventions grouped into 13 service categories by health service level. The 13 categories were informed by a recent publication from the *Lancet* Commission on Investing in Health, which grouped interventions by program area with related policies and financing arrangements^28^. Among basic PHC interventions, those with adequately composed indicators for meaningful readiness assessment demonstrated varying scores. The treatment of uncomplicated malaria achieved the highest readiness (84%). Readiness for first-line treatment of HIV patients with no TB was 72%. Facilities ready to provide STI management vary between 54% for treatment of gonorrhea and 77% for treatment of trichomoniasis. Readiness for six routine childhood vaccinations was around 65%. In contrast, the readiness of interventions addressing chronic respiratory diseases, such as routine longitudinal management of chronic asthma and COPD, was below 5%.

Readiness for PHC interventions requiring more advanced clinical skills, assessed starting at the RCH level, for example, the management of severe malaria among children, was 64%. Readiness for secondary prevention of ischemic heart disease and longitudinal management of heart failure was around 10%. Approximately 40% of facilities, excluding health centers, were ready to provide stabilization and referral of severe cases of asthma and COPD. In comparison, only 10% were ready to manage those severe cases. For interventions requiring district hospital-level care, around 85% of secondary and tertiary facilities were ready to provide management of post-abortion complications. However, less than 5% were ready to provide cardiovascular disease interventions. Readiness for surgical interventions ranged from 10% to 27% of secondary and tertiary facilities.

### Input-adjusted coverage by service categories

**Fig. 3:**
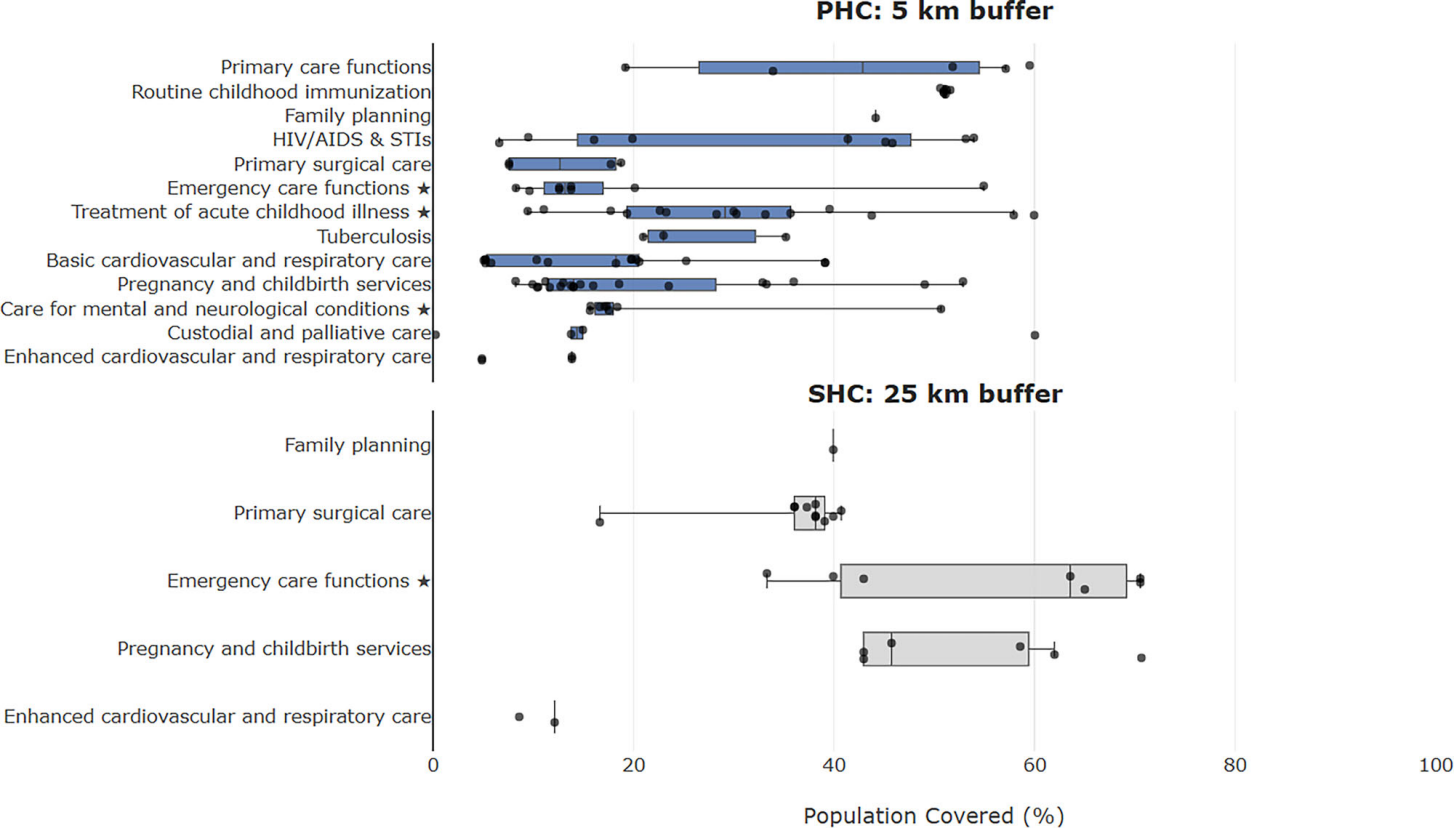
Input-adjusted coverage by service categories. ★ Intervention assessed based on the availability of medicines only. The interactive version is available online at: https://salia25.github.io/Coverage_Malawi_HHFA/Figure_5_population_coverage_all_bufferplot.html.

Figure 3 presents detailed input-adjusted coverage for 129 health interventions, emphasizing the levels at which readiness was assessed and the catchment area used to determine coverage. Input-adjusted coverage of treatment of uncomplicated malaria was at 58% of the population living within a 5 km radius of a ready facility. Other PHC interventions with a 50% input-adjusted coverage included the management of trichomoniasis, HIV treatment, and childhood vaccinations. In contrast, interventions addressing chronic respiratory diseases, such as routine longitudinal management of chronic asthma and COPD, had the lowest input-adjusted coverage, at around 10–15%.

The input-adjusted coverage for PHC interventions, assessed starting at the RCH level, for example, the management of complicated malaria among children, was 18%. Input-adjusted coverage for secondary prevention of ischemic heart disease and longitudinal management of heart failure was around 5%. Approximately 13% of the population lived within 5 km of facilities ready to provide stabilization and referral of severe cases of asthma and COPD. In comparison, only 5% lived within 5 km of a facility ready to manage those severe cases. For interventions requiring district hospital-level care, around 70% of the population lived within 25 km of a facility ready to provide management of post-abortion complications. However, input-adjusted coverage for cardiovascular disease interventions, such as the treatment of acute coronary syndromes and acute heart failure, was around 12% and 8.6%, respectively. Input-adjusted coverage for surgical interventions ranged from 17% to 40% of the population living within a 25 km radius of a facility equipped to perform these procedures. We also present the sensitivity analysis of different Euclidean distances in Supplementary Data 2 and 3.

### Input-adjusted coverage variation across districts

**Fig. 4:**
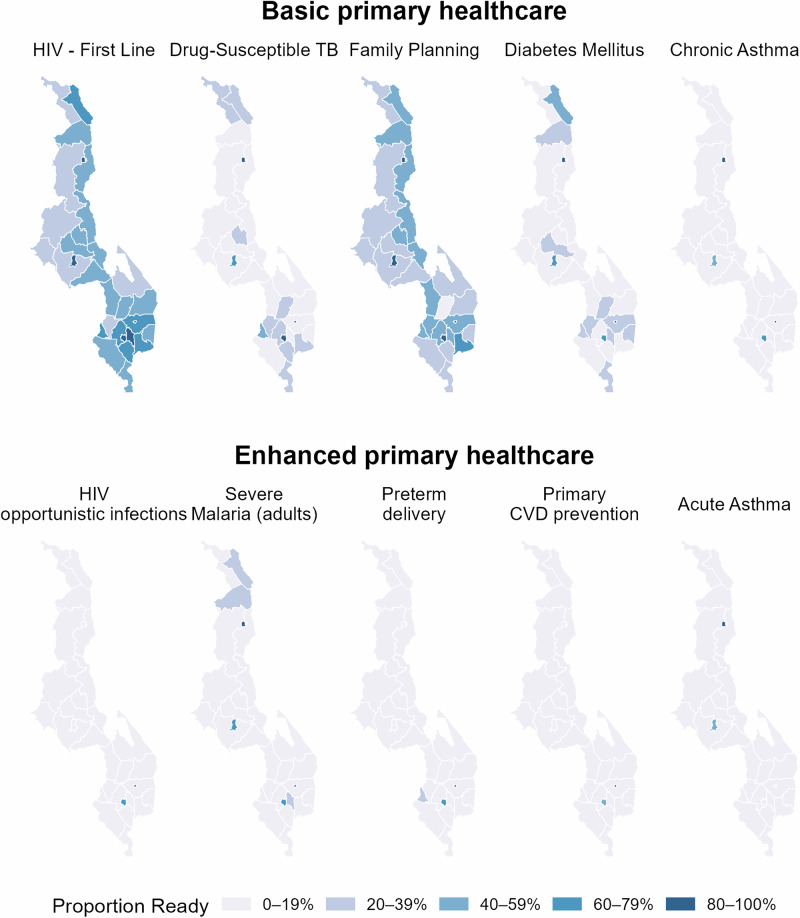
Input-adjusted coverage comparison across selected primary healthcare services.

Figure 4 highlights variations in input-adjusted coverage for selected basic and enhanced PHC services. Coverage of basic PHC services varied across services and districts. In contrast, the coverage of enhanced PHC services that should be available at the RCH level was low across districts. Coverage for first-line HIV treatment was generally at the level of 20–39% or 40–59%. Five districts in the southern region (Blantyre, Mulanje, Mwanza, Thyolo, and Zomba) and one district in the northern region (Karonga) had higher coverage levels in the 60–79% range. The Coverage for managing drug-susceptible TB was comparatively lower, with most districts below 20%. For family planning services, coverage ranged between 20–39% and 40–59% in most districts, except for the district of Balaka, which had a notably low coverage level (0–19%), and Mulanje, where coverage was between 60 and 79%. Apart from the district of Karonga, where the coverage was 40–59% for diabetes management, coverage varied between 20–39% and <20% across the other districts in Malawi. On the other hand, the coverage for managing chronic asthma was mainly below 20%. Supplementary File 2 details the input-adjusted coverage variation across districts for all interventions included in the analysis.

### Missingness and sensitivity analysis

Our missingness analysis (Supplementary Data 4 and 5) indicates that t_na, indicating not applicable entries, are not randomly distributed but instead follow systematic patterns consistent with facility level and service unavailability. Across multiple service domains, these entries appear to function as a substitute for explicit “not available” responses. For example, in health centers, X-ray machines and oxygen cylinders were coded as not applicable in all 471 health centers rather than recorded as “not available.” A similar pattern was observed for tuberculosis services: all alternative inputs for microscopy, chest x-ray, and GeneXpert, and TB medication options (14 variables) were reported as not applicable in 205 facilities.

Further examination of service-specific questions showed accurate documentation of availability for services with high readiness, such as HIV, STI, and malaria management interventions, where not applicable is not frequently used. For example, in 4 inputs regarding ELIZA analysis were not applicable for 25 out of 471 HC of the health centers. In contrast, for questions assessing whether facilities provide cardiovascular or respiratory disease care, facilities predominantly reported either “yes” or not applicable, with virtually no observations coded as “no” or “not available.” Based on these findings, we assume that these entries reflect systemic service unavailability conditioned on facility role and scope rather than data quality failures.

A similar pattern emerged in the sensitivity analyses examining the role of human resources for health (HRH) inputs (Supplementary Data 6). Removing HRH variables from the intervention definitions resulted in two distinct phenomena, depending on how service availability was documented. For interventions with well-documented availability and non-availability, excluding HRH inputs led to noticeable increases in input-adjusted coverage, for example, in the intervention “intermittent malaria prevention during pregnancy.” In contrast, for interventions primarily documented as not applicable with little or no explicit reporting of availability, the increase in input-adjusted coverage estimates was modest. For example, input-adjusted coverage for longitudinal management of asthma and COPD increased from 11% to 16% and 10% to 11%, respectively. A similar pattern was observed for tuberculosis interventions. These findings reinforce the interpretation that not applicable often reflects structural non-provision rather than data absence, and that HRH constraints interact differently with intervention readiness depending on whether services are formally within a facility’s expected scope of care. We observed a similar pattern when examining not applicable entries for diagnostic equipment (Supplementary Data 7).

## Discussion

This study presents a comprehensive analysis of input-adjusted coverage for a wide range of essential health interventions in Malawi. The findings highlight significant variations in coverage across service categories, facility levels, and geographical locations, offering critical insights into the implementation efforts of UHC policies and plans in Malawi. We used equal-weight composite indicators of essential health service inputs to assess health facility readiness based on 2019 Malawi HHFA data. We evaluated the adequacy of data points in the HHFA survey that can provide information on these essential service inputs and documented the comparative adequacy of each indicator. We developed readiness cascades for each intervention to ensure transparency of the composite indicators and identify bottleneck inputs, which provide critical insights into the input-related gaps that undermine service readiness and, consequently, coverage. We created a hierarchical catchment and used SAA, a robust method for determining input-adjusted coverage in Malawi.

Our analysis revealed high readiness and coverage for childhood vaccinations, HIV/AIDS, and malaria management, which reflects the prioritization of these services in national health policies and the support of international donors. For example, malaria management readiness was 84%, an improvement over previous estimates using 2013–2014 Service Provision Assessment (SPA) data, which reported readiness at 25%^29^. Despite methodological differences, this progress indicates focused investments in these high-priority interventions. Conversely, readiness and coverage for NCDs and surgical care remain low. For example, readiness for cardiovascular disease management at district hospitals was less than 15%, with only 8.6% of the population living within 25 km of a facility capable of providing care for acute heart failure. This disparity reflects critical imbalances in health service prioritization. It highlights the urgent need for targeted investments in under-resourced areas of care, especially since NCDs are starting to become a leading cause of mortality and morbidity in Malawi.

Our findings are broadly consistent with estimates of readiness-weighted population coverage for thirteen NCD services in Malawi. Cao et al. reported that population coverage at PHC facilities was extremely low across all NCD conditions, below 1%, with only chronic asthma, hypertension, and oral pain care reaching coverage levels of 10–30%^30^. Their study also demonstrated that incorporating road-network travel times, rather than Euclidean distance, yielded substantially lower estimates of accessibility compared to distance-based^30^.

Our study found that 41% of health facilities in Malawi were ready to provide early detection and treatment for neonatal sepsis and pneumonia. Similarly, Penzias et al. reported that 50% of neonatal units in Malawi that implemented the Newborn Essential Solutions and Technologies (NEST360) initiative met the service readiness criteria^31^. While our study uses composite indicators, Penzias et al. (2023) applied two alternative scoring frameworks. Despite the differences in the number and level of facilities assessed, both studies reveal comparable readiness gaps for neonatal care.

To contextualize our findings, we compared input-adjusted coverage estimates with commonly reported coverage indicators from the Global Health Observatory and DHS (Supplementary Data 8), primarily derived from household surveys, administrative data, or modeled estimates. These indicators are designed to measure broad service contact and do not incorporate geographic coverage or facility readiness considerations, limiting the ability to make direct comparisons. For example, although the Global Health Observatory reported IPTp3 coverage of 34% in Malawi in 2019, and we report it as 33%, we cannot conclude that these two values are basically the same.

Commonly reported coverage estimates tend to be higher than input-adjusted coverage estimates, highlighting the gap between nominal service use and the underlying capacity of the health system to deliver care. Wang et al. demonstrated that although Malawi achieved 93% crude coverage for facility deliveries based on DHS data, coverage drops to 66% after accounting for readiness gaps^32^. Also, crude coverage rates mask the hardships patients face in accessing geographically remote health facilities. Additionally, a lack of proximity to ready health facilities can discourage patients from seeking care. Anselmi et al. demonstrated that, in Mozambique, greater availability of human resources and equipment increases the probability of seeking care when sick^33^. Therefore, information on input-adjusted input coverage is a crucial complementary coverage measure to map supply-side insufficiencies.

Unlike studies that use a broad service readiness index to assess general facility capacity, our study adopted an intervention-specific approach. For example, while Leslie et al. provided an overall readiness index based on a fixed set of WHO indicators^34^, we evaluated readiness in terms of a facility’s capacity to deliver targeted interventions, providing a more precise measure of how facility-level readiness translates into population-level service coverage. We also estimated coverage at the district level, revealing subnational variations obscured by national aggregates.

Health service coverage estimation literature is increasingly utilizing geospatial mapping. Safura AH et al. used SAA to evaluate health service coverage and access in North Jakarta^35^. Amoah Nuamah J et al. used SAA to identify the number of communities outside the WHO-recommended 5 km health facilities catchment area^36^. Shaba et al. used SAA to assess the distribution of healthcare facilities in Plateau State, Nigeria^37^. This study advances coverage estimation by combining facility readiness and spatial analyses. The hierarchical catchment area approach allows for service-specific coverage assessments tailored to service levels and intervention types. Our analysis reveals that proximity to health facilities alone does not ensure access to all the services needed, particularly for conditions such as NCDs. Previous studies have not examined variations in coverage across interventions within the same facility, which is where this study adds value. McBride et al. reported that most households in Malawi live within 5 km of a health facility offering comprehensive PHC services^38^. Their definition of “comprehensive” services was based on facilities providing more than the median percentage of required services for their level. However, not including readiness consideration, they likely overstated overage.

Our method enhances the application of health facility surveys by incorporating geospatial mapping to generate credible estimates of health service coverage. These estimates are not merely descriptive of health system performance but serve as valuable insights for policy analysis and decision-making. The intervention-specific indicators we generate are more readily mapped to the EHBPs’ structure and can be used to monitor the implementation of specific EHBP components more accurately than assessing service coverage in aggregate, which can mask coverage variations across interventions and is often driven by high performance in HIV and child health.

Additionally, the intervention-specific and district-level disaggregation of input-adjusted coverage enables ministries of health to identify which inputs most constrain delivery of priority EHBP services and to target investments accordingly during annual planning, procurement, and phased scale-up decisions. By identifying supply-side bottlenecks, our findings provide practical information for policymakers seeking to strengthen service delivery and prioritize investments across EHBP components and countries, where multiple census facility data points can compare changes over time directly. This methodological advancement addresses the limitations of current practices, which often rely on proxy estimates and expert opinions to fill in gaps in coverage data, thereby reducing uncertainty and improving the accuracy of health policy planning.

Further, as our analysis shows, coverage of health services depends on facility readiness, catchment area, and number of facilities, which highlights that task shifting to health centers is critical to achieve meaningful gains in coverage. For example, increasing the readiness of RCH, which are limited to 47 facilities, cannot achieve meaningful gains in chronic disease coverage. Engaging HC in providing basic services for chronic diseases is critical to achieve a meaningful increase in coverage.

Although the HHFA is not designed to assess service coverage, it provides a rich source for evaluating input-adjusted coverage across various health interventions. In particular, being a census survey and the inclusion of facility geospatial coordinates allow integration with geospatial population data, enabling coverage calculations that offer nuanced insights into the relationship between service readiness and geographical health facility coverage. Several countries have already conducted HHFA surveys and hence can use them as an essential complementary source for monitoring UHC implementation efforts in addition to household surveys such as DHS and MICS. Although input-adjusted coverage only partially reflects elements of the broader definition of effective coverage, it represents an advancement in the current coverage estimation methods that is less resource-intensive than attempting to estimate effective coverage for a broad list of interventions. This study utilized the HHFA to assess 129 interventions that included most of the interventions outlined in Malawi’s EHBP. As such, it can serve as a baseline for monitoring ongoing and future efforts to improve access to essential services: the HHFA could be repeated every few years, and coverage metrics for the same service could be compared between years to assess trends and persistent challenges in covering hard-to-reach geographies. The level of disaggregation of information can guide local planning efforts and allow targeting investment and available budgets toward high-priority interventions. Furthermore, the flexibility of the composite indicators approach will enable tailoring them to reflect locally relevant inputs, ensuring alignment with national health priorities and contextual needs. Furthermore, the same composite indicator method can be applied to additional interventions or surveys tailored to the local context.

A key limitation of our analysis relates to the scope and design of the HHFA. The HHFA is primarily designed for primary healthcare and less comprehensively assesses the readiness requirements of secondary and tertiary care facilities. For example, the survey does not cover any input regarding the management of cancer. As a result, our assessment of secondary services is limited by the inputs measured by the HHFA, and we do not attempt to evaluate tertiary care readiness. When only a narrow set of inputs is used for the indicator (for example, medicine availability alone), readiness scores may be biased upward because essential but unmeasured diagnostics, or human resources cannot be evaluated. Further, our estimates may also underestimate service availability for interventions commonly delivered through outreach activities, such as routine vaccination.

Also, even though our analysis is based on census data, our results might still be subject to measurement error stemming from non-response, reporting inaccuracies, or incomplete documentation. As shown in the sensitivity and missingness analysis, reporting inaccuracies adds uncertainty to readiness and coverage estimates.

Our metric, Input-adjusted coverage, does not measure actual service utilization, quality of care, or health outcomes, and should therefore be interpreted as an estimate of potential service capacity rather than effective coverage. Also, the use of equal-weight composite indicators reflects a pragmatic decision driven by data availability and transparency, but it necessarily simplifies the relative importance of inputs across interventions.

Another major limitation is the absence of direct measures of service provider availability. Workforce capacity is a core determinant of service delivery, particularly in under-resourced settings where shortages of trained staff present substantial barriers. Despite including the available HHFA questions around health workers and capping our analysis to functional health centers and hospitals without HRH measures, estimates of readiness would be substantially overestimated, as the Malawi health system suffers from high vacancy rates reaching up to 80%^39^. Yet, nonetheless, because ministries of health typically exert greater operational control over commodity procurement and distribution than over workforce allocation, a function often influenced by broader economic and civil service systems, tracking commodities still provides meaningful insight into where EHBP implementation is most actionable.

Our geospatial analysis relies on Euclidean distance to define facility catchments, which does not account for road networks, terrain, transportation availability, or seasonal barriers. As a result, geographic coverage may be overestimated, particularly in remote or hard-to-reach areas. To mitigate this limitation, we applied conservative distance thresholds grounded in the literature and conducted sensitivity analyses (Supplementary Data 2 and 3) demonstrating how alternative distance assumptions affect coverage estimates. These analyses confirm that increasing catchment thresholds systematically inflate coverage, reinforcing the importance of cautious interpretation. Additionally, district-level coverage estimates assume that facility catchments do not cross administrative boundaries; this simplification may underestimate coverage in border areas where patients frequently seek care across districts.

Our analysis focuses exclusively on publicly financed health facilities and does not include private for-profit providers, which account for approximately 11% of health facilities in Malawi. Although private facilities serve a relatively small proportion of the population and offer a limited range of services, their exclusion may lead to an underestimation of overall service availability and population coverage. Consequently, our estimates should be interpreted as reflecting coverage within the public health system and its capacity to deliver services under the UHC framework, rather than total service provision across all sectors. Fortunately, PFP facilities provide less than 3% of the health services in Malawi, so the potential bias is minimal in our study^40^. As the PFP sector role grows or in other countries where private healthcare is more frequently used (e.g., in South Asia), countries would need to consider including private facilities both during the implementation of the HHFA and in their analysis.

Despite these methodological limitations, we present a statistical method that leverages HHFA data and can be used in numerous countries to understand geographical differences in readiness and input-adjusted coverage. Combining census facility data with geospatial population distributions provides ministries of health with actionable insights on investments needed to close service coverage gaps that complement the routine data system and utilization survey data, such as the DHS. The method can be applied to similar facility surveys, such as SARA and SPA, and can complement routine administrative and household survey data in settings with limited external support. Further countries can use this method for a subset of priority services and integrate it within the routine monitoring system for EHBPs. Expanding future HHFA rounds to include more complete questions on HRH, NCDs, and advanced care would substantially improve the precision of readiness estimates. Future analysis could integrate quality-of-care data from the HHFA quality module to estimate quality-adjusted coverage. Furthermore, incorporating road-network travel times and human travel behavior into geospatial analyses would refine proximity estimates and generate more accurate coverage assessments.

## Methods

### Study setting

Malawi’s healthcare system operates through public, private for-profit (PFP), and private not-for-profit (PNFP) facilities. Government and Christian Health Association of Malawi (CHAM) facilities do not require point-of-care payments^21^. PFP facilities collectively represent 11% of all healthcare institutions and provide 3% of services, catering to a small population segment^23,41^. Apart from a limited number of large PFPs, most facilities are rudimentary and are not adequately coordinated or regulated. The public healthcare system, which is the main service provider in the country, is organized into three tiers: primary care is provided in health centers and rural community hospitals (RCHs), district hospitals (DHs) are the first referral level, and central hospitals (CHs) offer advanced and specialized medical services^21^.

### Data sources

The study utilized multiple sources to ensure a comprehensive assessment of input-adjusted population coverage. The list of interventions included in the analysis was derived from the Disease Control Priorities Project^42^. These interventions span major health areas, such as infectious diseases, surgical care, and mental health, and were recommended for national EHBPs based on value for money, feasibility, and relevance in low- and middle-income countries. The most recent Disease Control Priorities contained 218 interventions; these underwent further standardization and harmonization with the WHO’s UHC Compendium through an additional literature review and experience working with practitioners from several low- and middle-income countries who participate in our Bergen Center for Ethics and Priority Setting consortium^43,44^. We mapped these interventions to the HHFA core questionnaire questions created in 2018 to determine which interventions are best captured by the survey^19^.

We used a structured review process to define readiness composite indicators. At the first stage, we relied on the Service Availability and Readiness Assessment (SARA) reference manual, which defines internationally recognized availability and readiness indicators and serves as the global standard for facility readiness assessment. For interventions not specified in SARA, we consulted WHO clinical guidelines and the Malawi Standard Treatment Guidelines. At the second stage, we reviewed peer-reviewed literature to identify clinically essential inputs for interventions not specified in the sources above. Supplementary Data 9 lists interventions included in the analysis, the inputs used to assess readiness, and sources used.

### Population and sample

We used the 2019 Malawi HHFA Data to assess healthcare facilities’ service-specific readiness. The 2019 HHFA in Malawi covered all health facilities in Malawi. The analysis focused on 563 publicly financed health centers and hospitals, owned by the government and CHAM, that are functional and have qualified staff to provide outpatient services. The analysis excluded PFP facilities (64 facilities) since they serve a limited population and require high user fees, which prevent most Malawians from accessing their service^45^. Reports show that government facilities provide 68% of services in Malawi, while the CHAM provides around 29%, and the commercial health sector provides less than 3% of the services^39,40,46^.

### Operational definitions

We define “service readiness” for a given intervention (service) as a facility being fully functional and having the inputs necessary for delivering that intervention. Our approach builds on the WHO definition of service readiness as the facility’s capacity to provide services, based on the presence of a core set of inputs, including basic amenities, trained staff, clinical guidelines, basic infrastructure, equipment, medicines, and diagnostic tests^47^. Guided by this definition, we operationalized readiness using an intervention-specific “minimum inputs” approach, in which facilities were classified as ready only if all essential inputs required for service delivery of a given intervention were present. Our approach is consistent with prior applications of SPA, SARA, and HHFA data, where readiness was measured using composite indicators reflecting the availability of essential service inputs. Similar approaches have been used to assess readiness for NCD services across multiple countries (Moucheraud et al.^48^), readiness for NCDs and injuries at first-referral hospitals (Gupta et al.)^49^, readiness of primary care centers in Nepal^50^, family planning service readiness in low-resource settings (Rahman et al.^51^), and NCD service readiness in publicly financed facilities in Malawi (Ahmed et al.^52^).

**Fig. 5:**
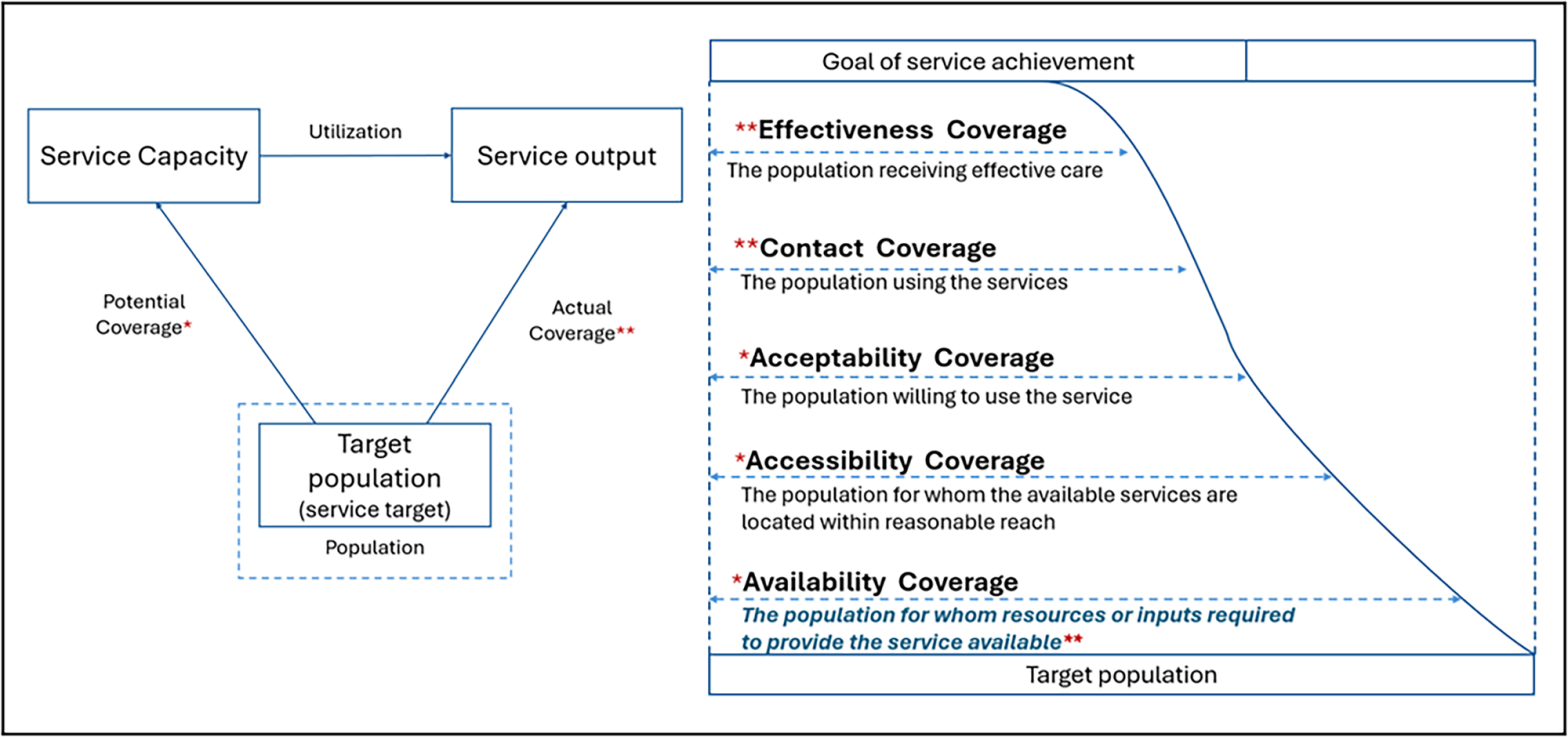
Tanahashi’s health service coverage and utilization framework. *within the diagram delineate Tanahashi’s classification of coverage measurements according to their relation to coverage type (potential* vs actual**).

Our definition of input-adjusted coverage is informed by Tanahashi’s Health Service Coverage and Utilization Framework^25^, and the Effective Coverage Think Tank’s^27^ standardized cascade for measuring effective coverage. Based on the premise that health service coverage depends on a service’s ability to engage with the intended population and improve their health, Tanahashi’s framework (Fig. 5) distinguishes between potential and actual coverage and specifies five related measures: availability, accessibility, acceptability, contact, and effectiveness coverage.

The Effective Coverage Think Tank in 2019 proposed a standardized cascade that aligns closely with Tanahashi’s stages and defined input-adjusted coverage as the proportion of the population in need who come into contact with a health service that is *ready* to provide care, emphasizing that health facility assessments are the most suitable data source for this step in the cascade^27^. In this study, we define input-adjusted coverage as the share of the population living within the catchment of a facility that meets minimum readiness requirements for a given intervention. However, it reflects only the early, potential coverage stages of service delivery and does not represent access or utilization.

### Data management

In the first phase we mapped the HHFA- Core Module with the Disease Control Priorities interventions list, we categorized the interventions into three distinct groups: (1) interventions with multiple inputs comprehensively covered by the HHFA, (2) interventions for which only the availability of medicines is assessed, and (3) interventions that have no relevant questions in the HHFA, which we did not include in the analysis.

Following the mapping, we developed composite indicators to assess facility readiness, incorporating key minimal readiness inputs. We classified the composite indicators based on their quality, reflecting the extent to which they comprehensively captured the necessary readiness elements (Supplementary Data 9). Additionally, we mapped the interventions to Malawi’s EHBP^21^ to align the analysis with the planned level of service availability across Malawi’s health system.

### Data analysis

For conducting the facility readiness assessment we classified interventions into three groups: (1) Basic primary care that should be available at health centers but might be available at higher-level facilities (RCHs, DHs, CHs), (2) Enhanced primary care that should be available at RCHs but may be available at higher-level facilities, and (3) Secondary care that should be available in DHs as well as CHs. Restricting readiness estimation to the lowest health system level expected to provide service ensures that readiness is not overestimated, while assessing readiness at a higher than planned service level accounts for the fact that Gate-keeping is not strictly enforced in Malawi^21^. We calculated intervention readiness as:

1 $${Intervention}\,{Readiness}=\frac{\displaystyle {\sum }_{r=1}^{N}{I}_{r}}{N}$$

N= Total number of facilities expected to provide the service

Ir=1 if facility r meets readiness criteria, otherwise 0

**Fig. 6:**
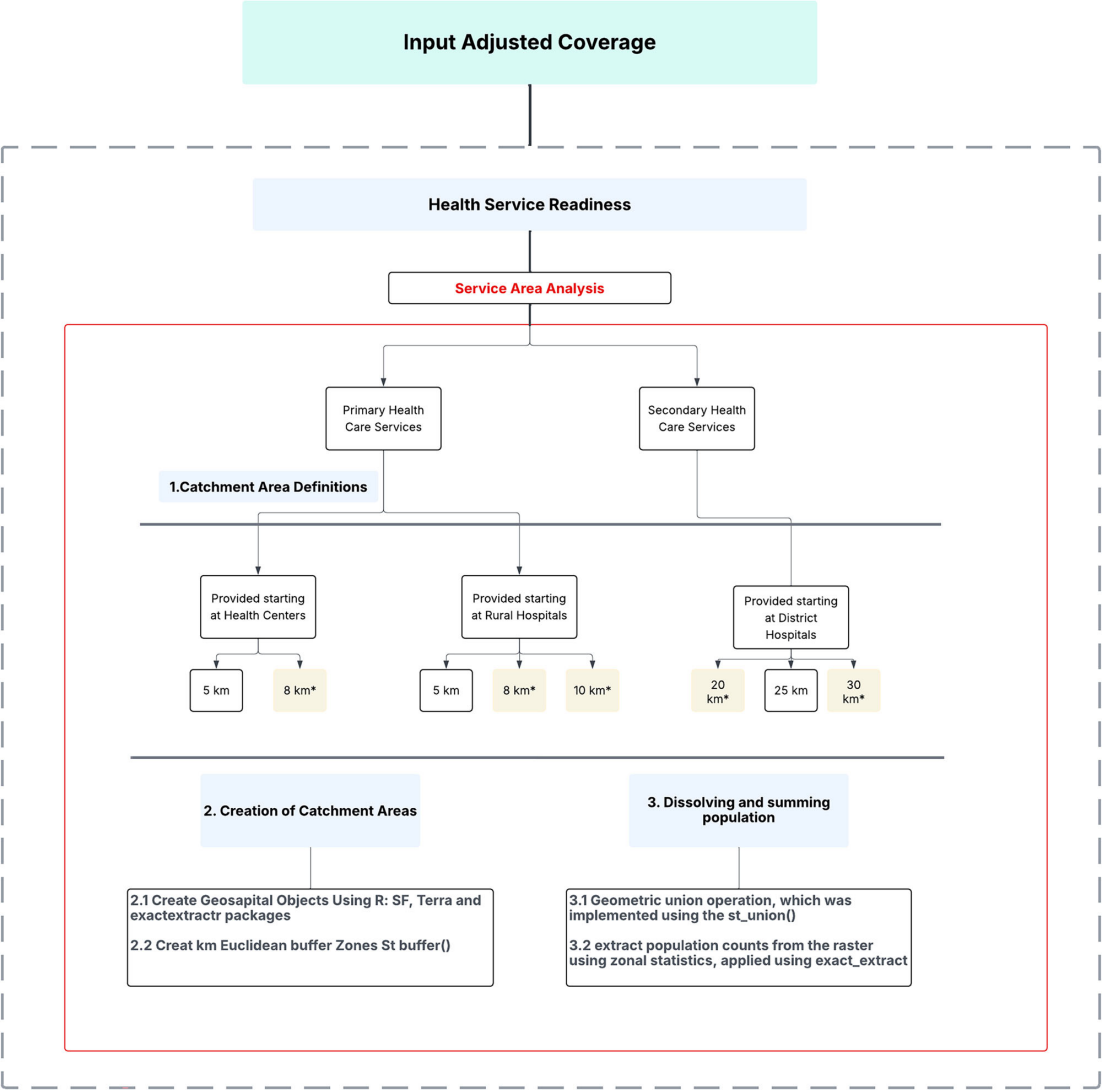
Framework for estimating Input-adjusted coverage. * Sensitivity analysis.

To estimate population coverage, we conducted a service area analysis (SAA) to estimate the proportion of the population that encounters a health facility that is ready to provide the service in question (Fig. 6). Chen et al. state that catchment areas represent the acceptable distance that populations are willing to travel^53^. SAA delineates the service and maps the geographical boundaries within which the population resides or is expected to travel to access care^54,55^. We utilized the WorldPop 2020 Population Raster dataset, which provides high-resolution population estimates per 1 km grid cell for Malawi^56^. Despite using data from HHFA 2019 and population data from 2020, with known population growth patterns, we do not anticipate significant population shifts that would drastically alter population distributions or counts within a single year.

As summarized by Fig. 6, we adopted a nuanced approach to catchment area determination, emphasizing the importance of considering the type of health service rather than solely relying on the classification of health facilities.

- A 5km catchment area was delineated around all health facilities included in the analysis, assuming that the population should be able to access primary care within 5 km of any facility ready to provide care. We employed catchment area definitions that are widely used globally and serve as health service planning standards for many ministries of health, particularly in Sub-Saharan Africa. The WHO accepts that a 5 km radius is associated with the capacity and access component of the UHC and is used as part of the UHC Service Coverage Index (SCI)^57^. Mitikie et al. defined accessibility of institutional delivery service as the availability of a health facility providing delivery service within a 5 km radius, and Ashiagbor et al. used a 5 km radius to assess geographic accessibility to healthcare in the Ashanti Region of Ghana^58,59^. Also, evidence from Malawi demonstrates that larger thresholds, such as the 8 km national planning standard, tend to overestimate accessibility because they do not account for travel barriers. Phiri and Munthali showed that, while a simple 8 km radius suggested that 94% of households had access to a facility, network-based distance and walking-time modeling reduced this estimate to 87% and 66%, respectively, which highlights that realistic coverage is considerably lower when terrain and road networks are incorporated^23^. These findings support the use of a more conservative 5 km threshold for PHC catchments when relying on Euclidean distance.
- To assess the coverage of secondary care health services, a 25km catchment area was established around secondary and tertiary hospitals^60^. Specialized services offered at central hospitals were excluded from our analysis due to the lack of related HHFA questions. Using the R, facility coordinates were converted to spatial objects and reprojected to UTM Zone 36S for accurate distance calculations. We generated 5 and 25 Km Euclidean buffers around each facility based on the expected service level.

Because catchment areas often overlap, particularly in urban and peri-urban settings, analyzing buffers independently may lead to double-counting. Therefore, all buffers surrounding ready facilities were dissolved using a geometric union operation. Dissolving merges overlapping polygons into a single continuous service area, and hence, each population grid cell is counted once. The population counts from the raster were extracted from the dissolved polygon using zonal statistics and summing raster values intersecting the polygon, yielding the unique population residing within reach of at least one ready facility.

The percentage of the population covered for each condition, nationally, was computed as follows:

2 $${\mathrm{Input}}\,{\mathrm{adjusted}}\,{population}\,{coverge}\,\left( \% \right):\frac{{P}_{{dissolved}}}{{P}_{{total}}}$$

*P*_dissolved_: the total population residing within the merged (dissolved) catchment area of all ready facilities.

*P*_total_: The total population of Malawi.

The percentage of the population covered for each condition within the district was computed as follows:

3 $${\mathrm{Input}}\,{\mathrm{adjusted}}\,{population}\,{coverge}\,\left( \% \right):\frac{{P}_{{covered}\,{within}\,{didtrict}}}{{P}_{{district}\,{total}}}$$

*P*_covered within district_: The total population residing within a 5 km or 25 Km buffer of all ready facilities located within the district, depending on the service level.

P_district total_: The total population of the district.

### Ethical considerations

All analyses are reported in an aggregated format with no facility or individual identifiers to ensure that the use of geospatial analyses does not pose risks to privacy or re-identification. Since this study did not involve human subjects, no formal ethical approval was required.

## Supplementary information

Supplementary Data

Supplementary File 1

Supplementary File 2

## Data Availability

The data that support the findings of this study are available from the Ministry of Health, Malawi, but restrictions apply to the availability of these data, which were used under license for the current study, and so are not publicly available. Data are, however, available from the authors upon reasonable request and with permission of the Ministry of Health, Malawi.

## Code availability

The underlying code [and training/validation datasets] for this study are available in “Coverage_Malawi_HHFA” and can be accessed via this link https://github.com/salia25/Coverage_Malawi_HHFA.
