# Supplementary material for: Measures to monitor the implementation of Essential Health Benefit Packages at a national scale
Source: Npj Health Syst. 2026 Apr 29;3:27. doi: 10.1038/s44401-026-00081-4 (PMC13354221; doi:10.1038/s44401-026-00081-4)
Supplement: Supplementary file 2 — Supplementary File 1 [file 44401_2026_81_MOESM2_ESM.pdf]

Readiness Cascades (PHC_HC+)

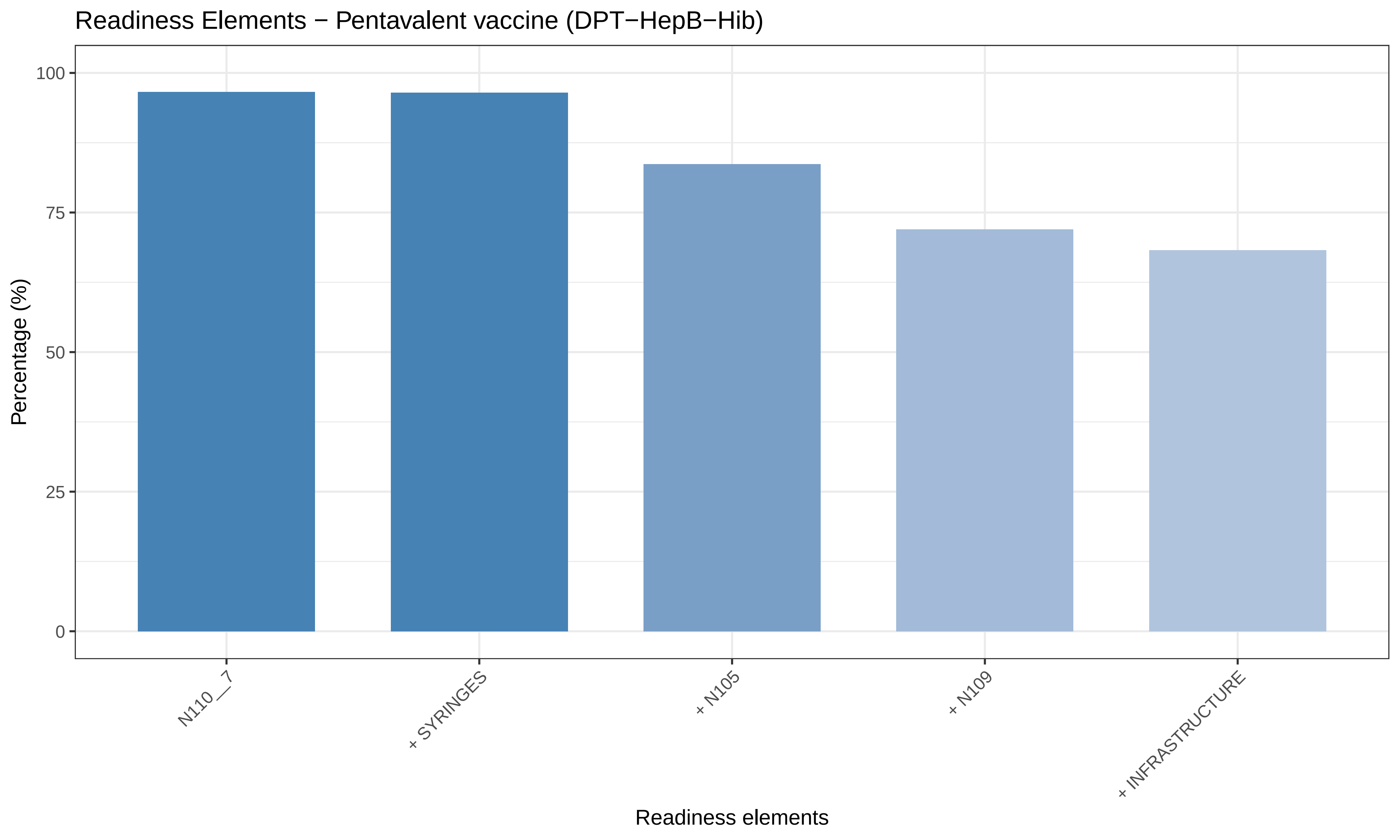

Readiness Elements − Pentavalent vaccine (DPT−HepB−Hib)
Percentage (%)
100
75
50
25
0
N110__7
+ SYRINGES
+ N105
+ N109
+ INFRASTRUCTURE
Readiness elements

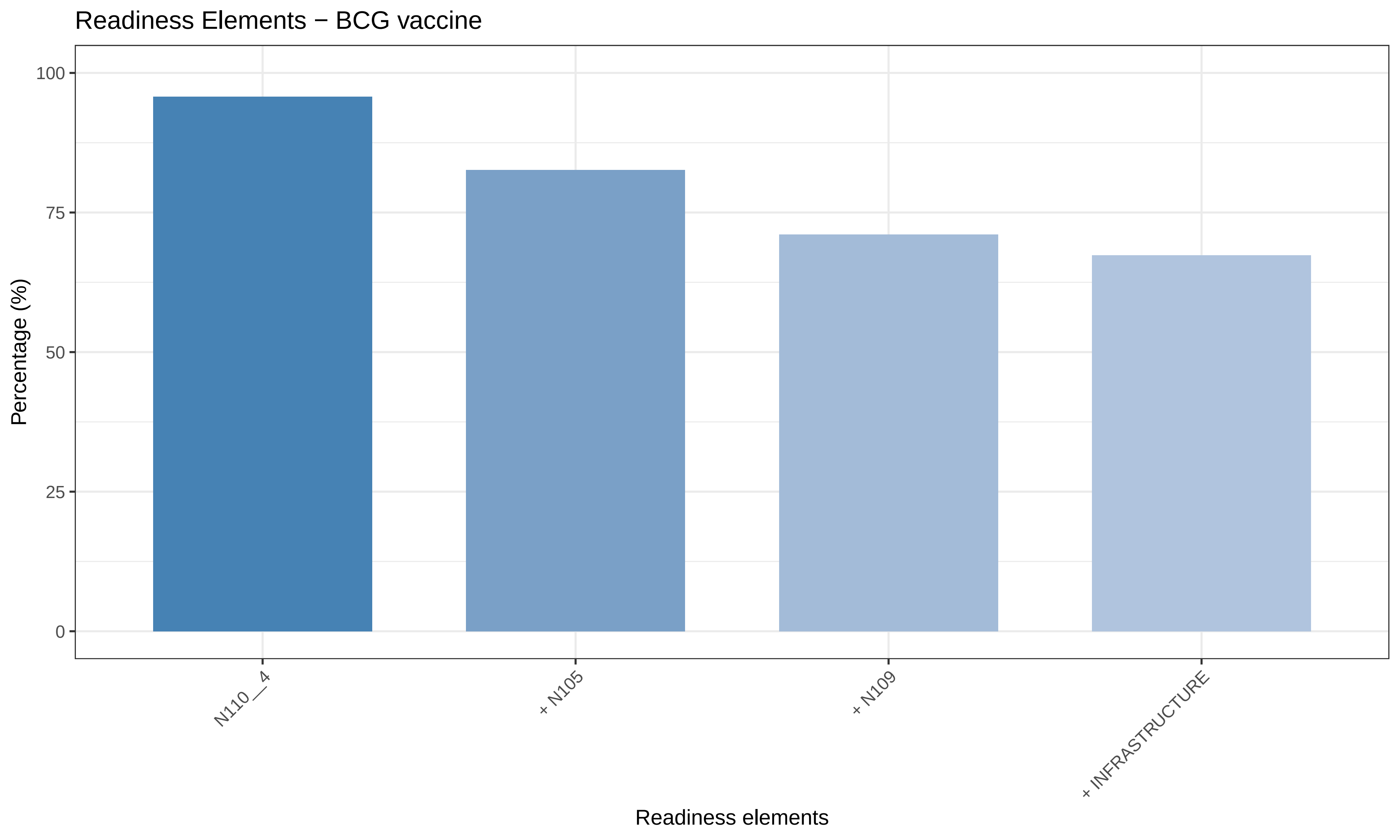
Readiness Elements − BCG vaccine
Percentage (%)
100
75
50
25
0
N110__4
+ N105
+ N109
+ INFRASTRUCTURE
Readiness elements

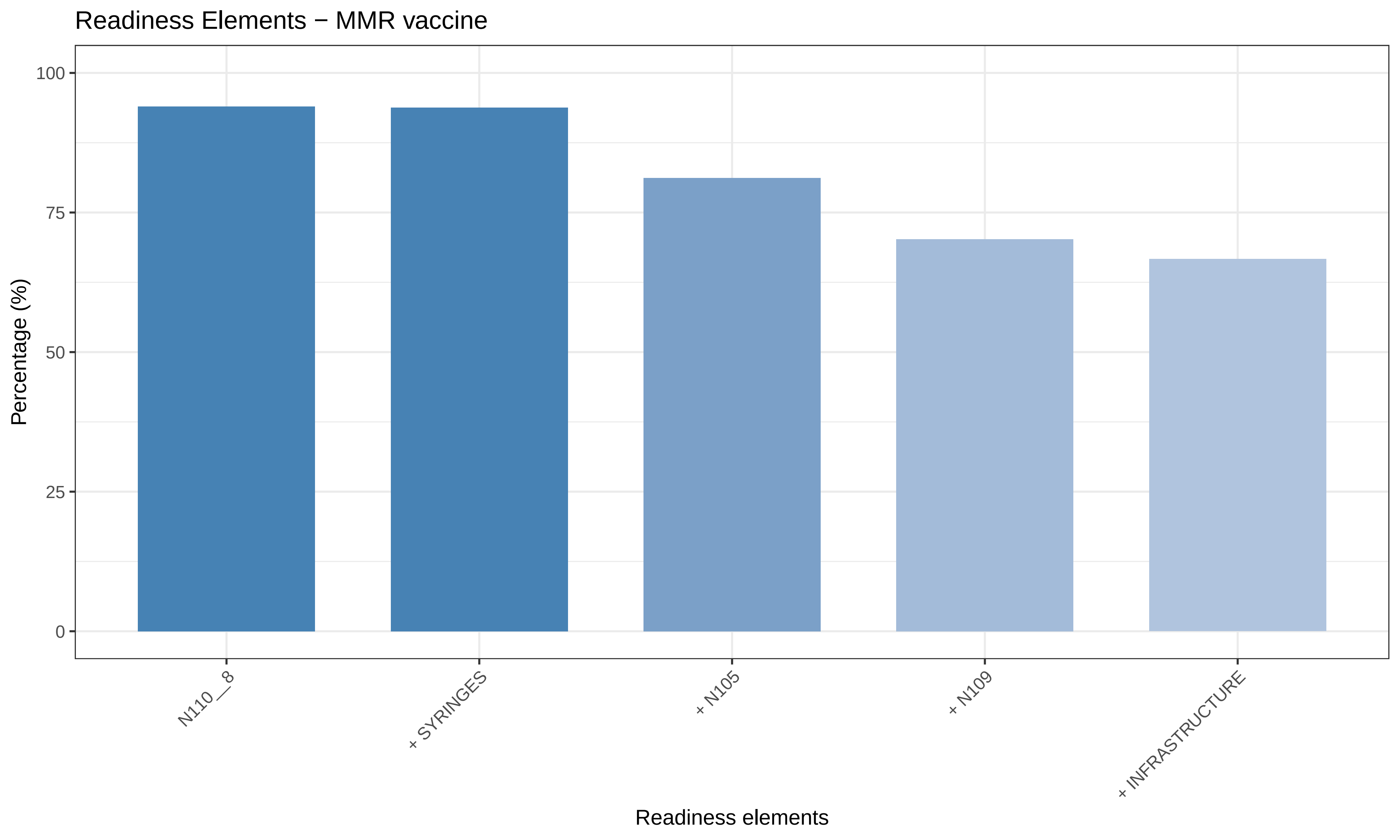
Readiness Elements − MMR vaccine
Percentage (%)
100
75
50
25
0
N110__8
+ SYRINGES
+ N105
+ N109
+ INFRASTRUCTURE
Readiness elements

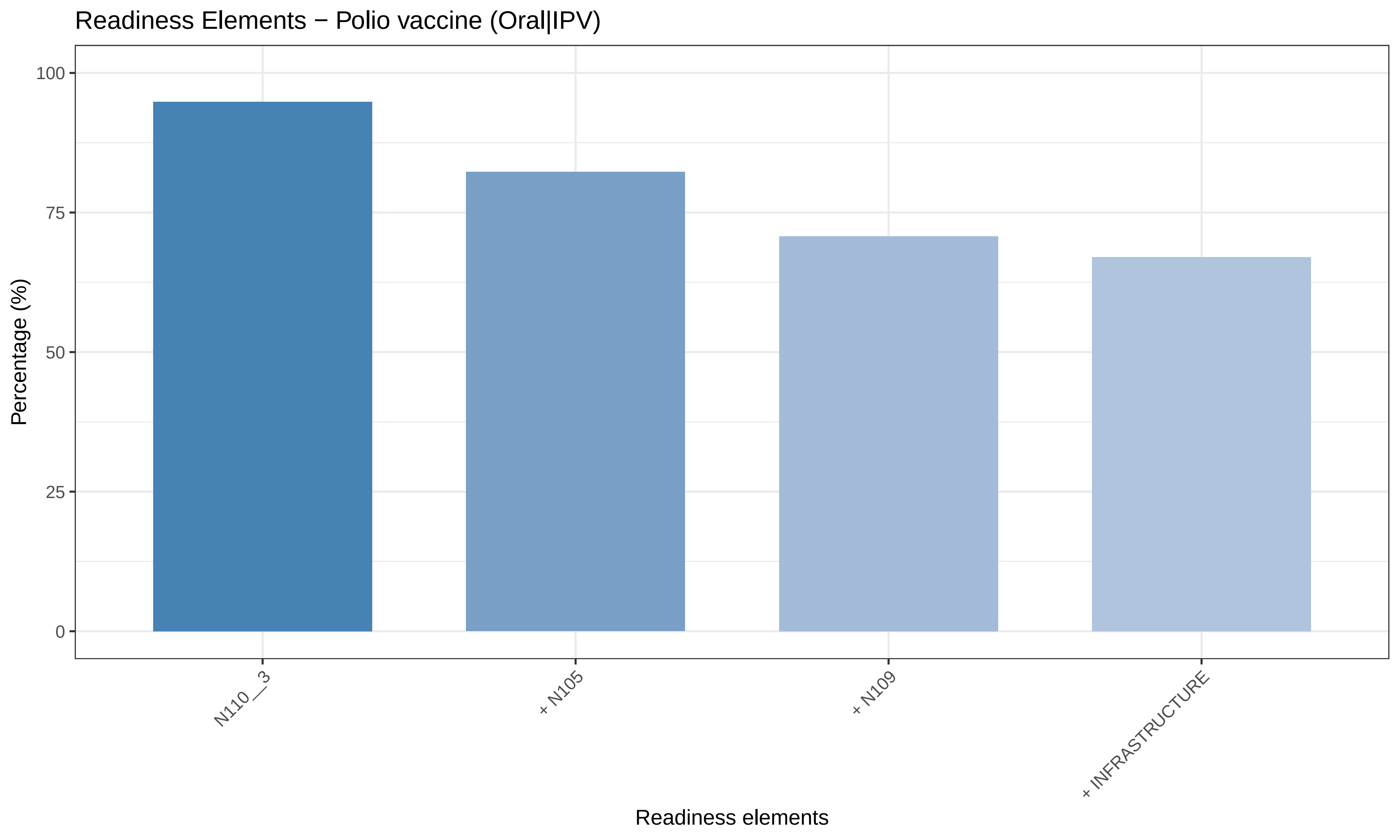
Readiness Elements − Polio vaccine (Oral|IPV)
Percentage (%)
100
75
50
25
0
N110__3
+ N105
+ N109
+ INFRASTRUCTURE
Readiness elements

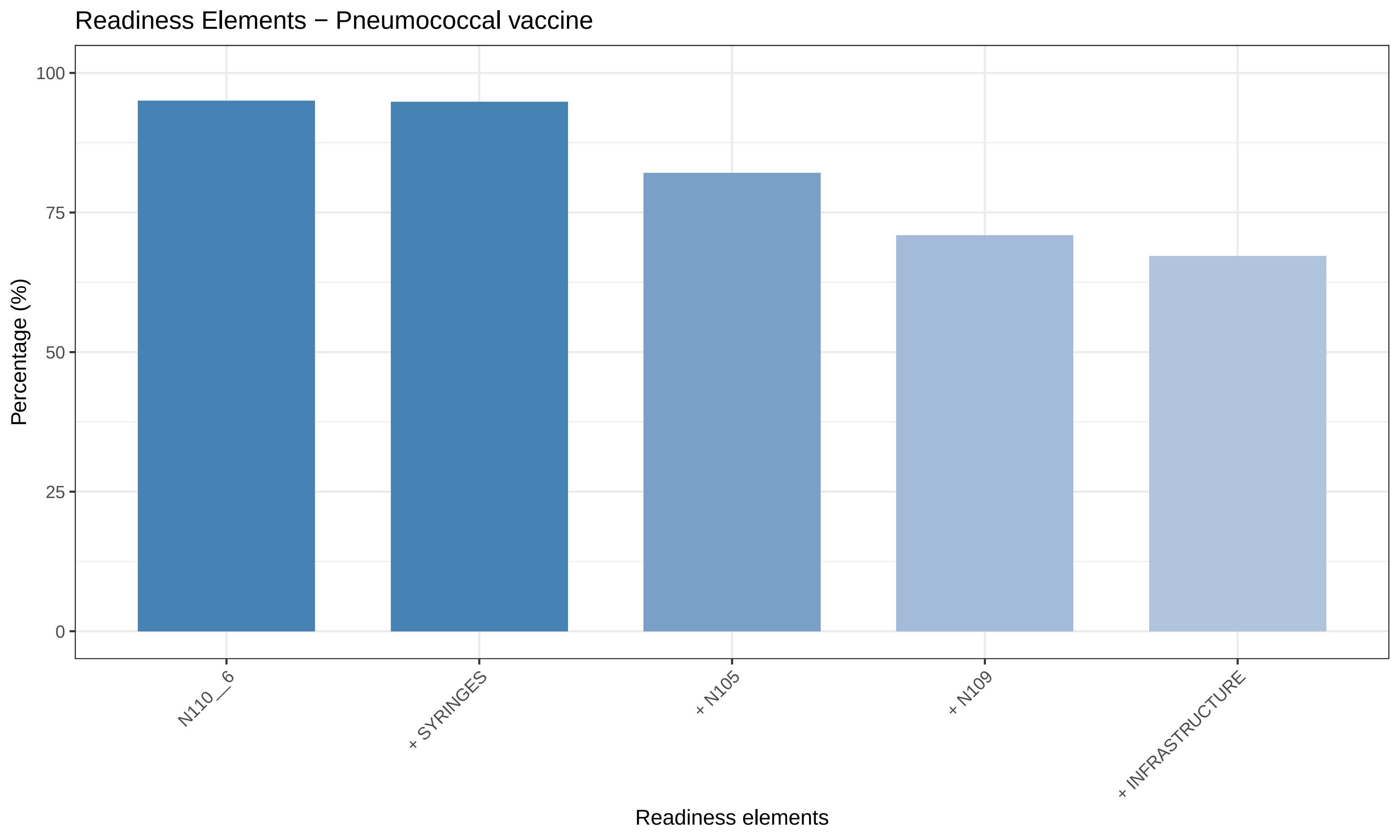

Readiness Elements − Pneumococcal vaccine
100
75
50
25
0
Percentage (%)
N110__6
+ SYRINGES
+ N105
+ N109
+ INFRASTRUCTURE
Readiness elements

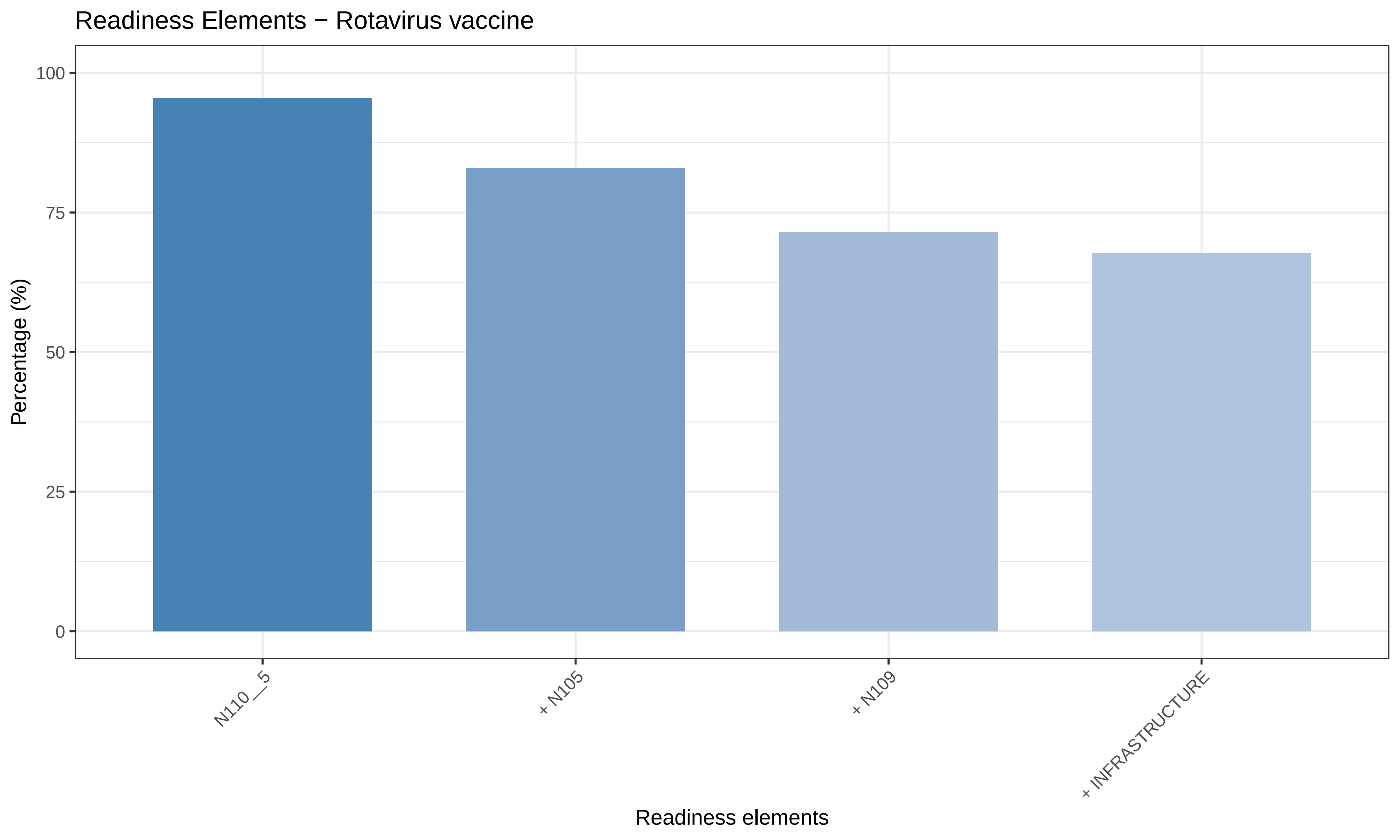
Readiness Elements − Rotavirus vaccine
Percentage (%)
100
75
50
25
0
N110__5
+ N105
+ N109
+ INFRASTRUCTURE
Readiness elements

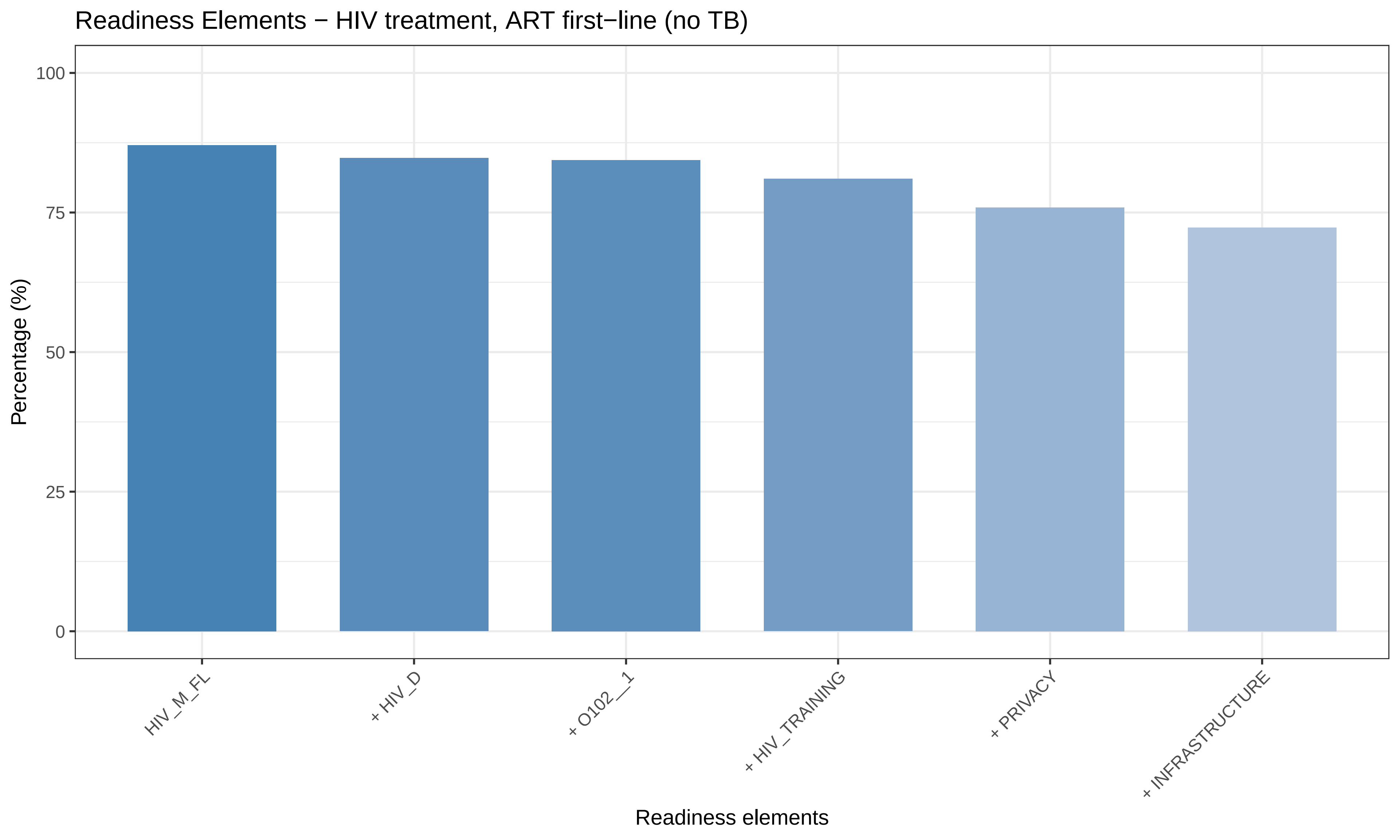
Readiness Elements − HIV treatment, ART first−line (no TB)
Percentage (%)
100
75
50
25
0
HIV_M_FL
+ HIV_D
+ O102__1
+ HIV_TRAINING
+ PRIVACY
+ INFRASTRUCTURE
Readiness elements

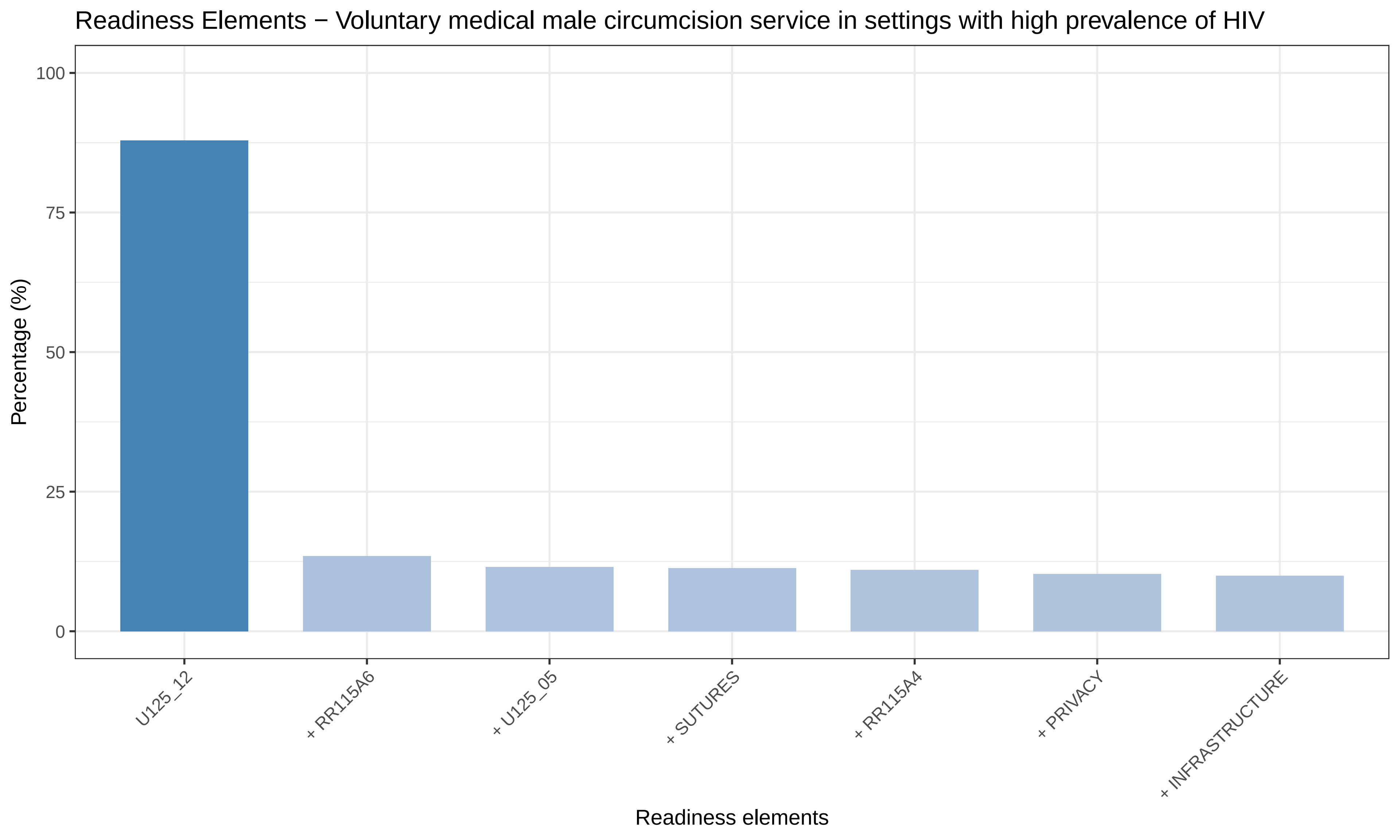

Readiness Elements − Voluntary medical male circumcision service in settings with high prevalence of HIV
Percentage (%)
100
75
50
25
0
U125_12
+ RR115A6
+ U125_05
+ SUTURES
+ RR115A4
+ PRIVACY
+ INFRASTRUCTURE
Readiness elements

Readiness Elements − TB preventive therapy.(Isoniazide) for high risk people (e.g. PLHIV)

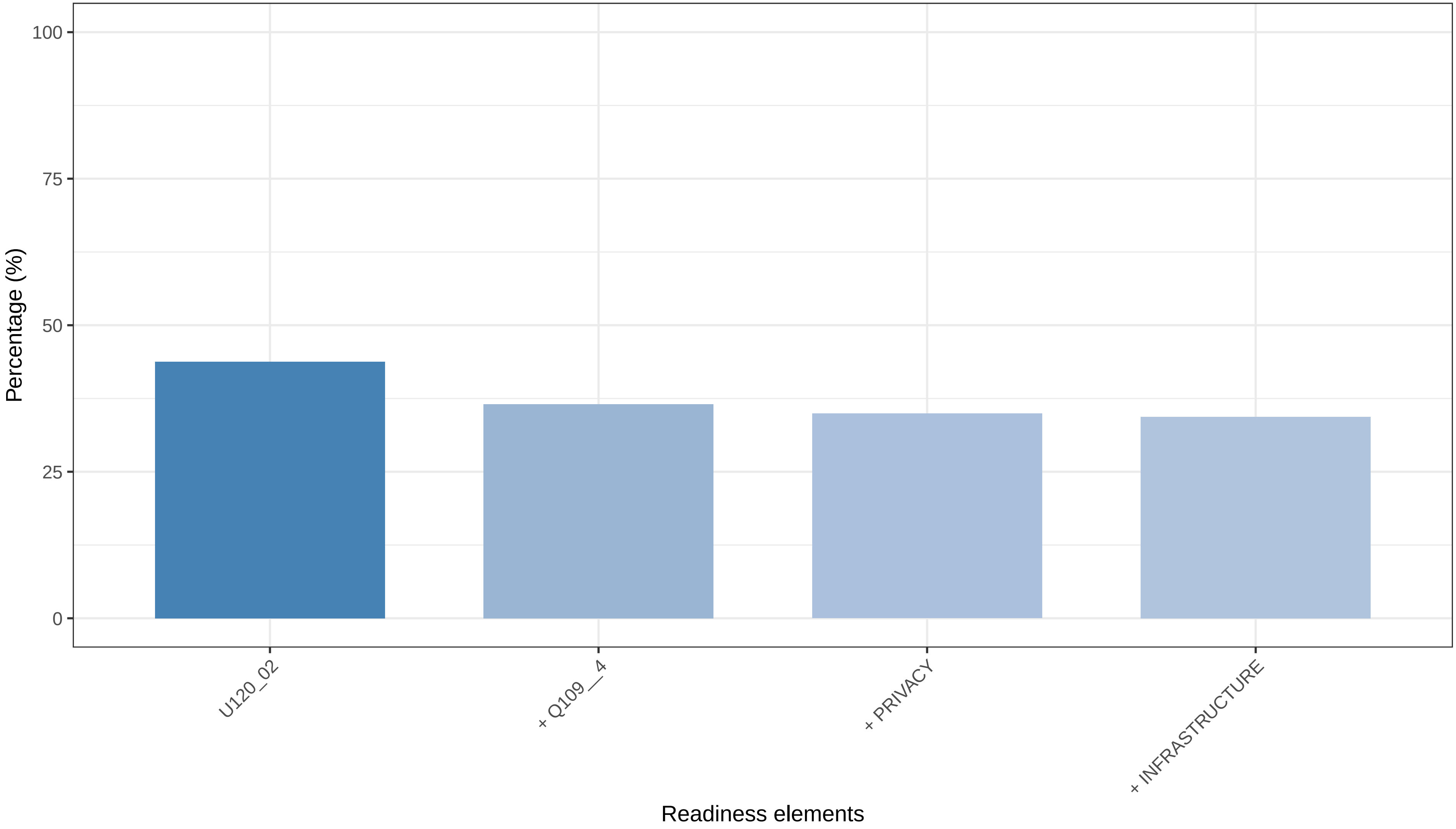

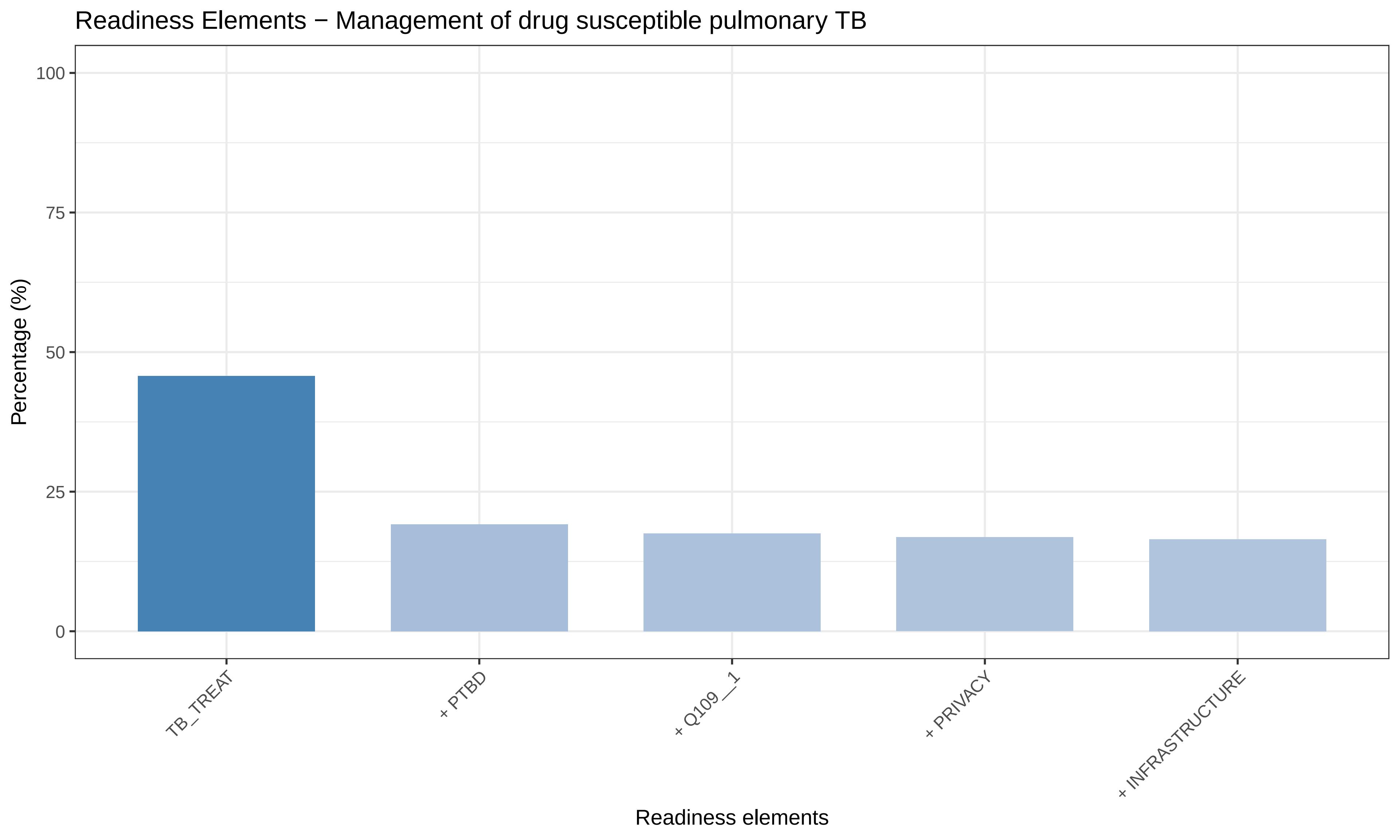

Readiness Elements − Management of drug susceptible pulmonary TB
Percentage (%)
100
75
50
25
0
TB_TREAT
+ PTBD
+ Q109__1
+ PRIVACY
+ INFRASTRUCTURE
Readiness elements

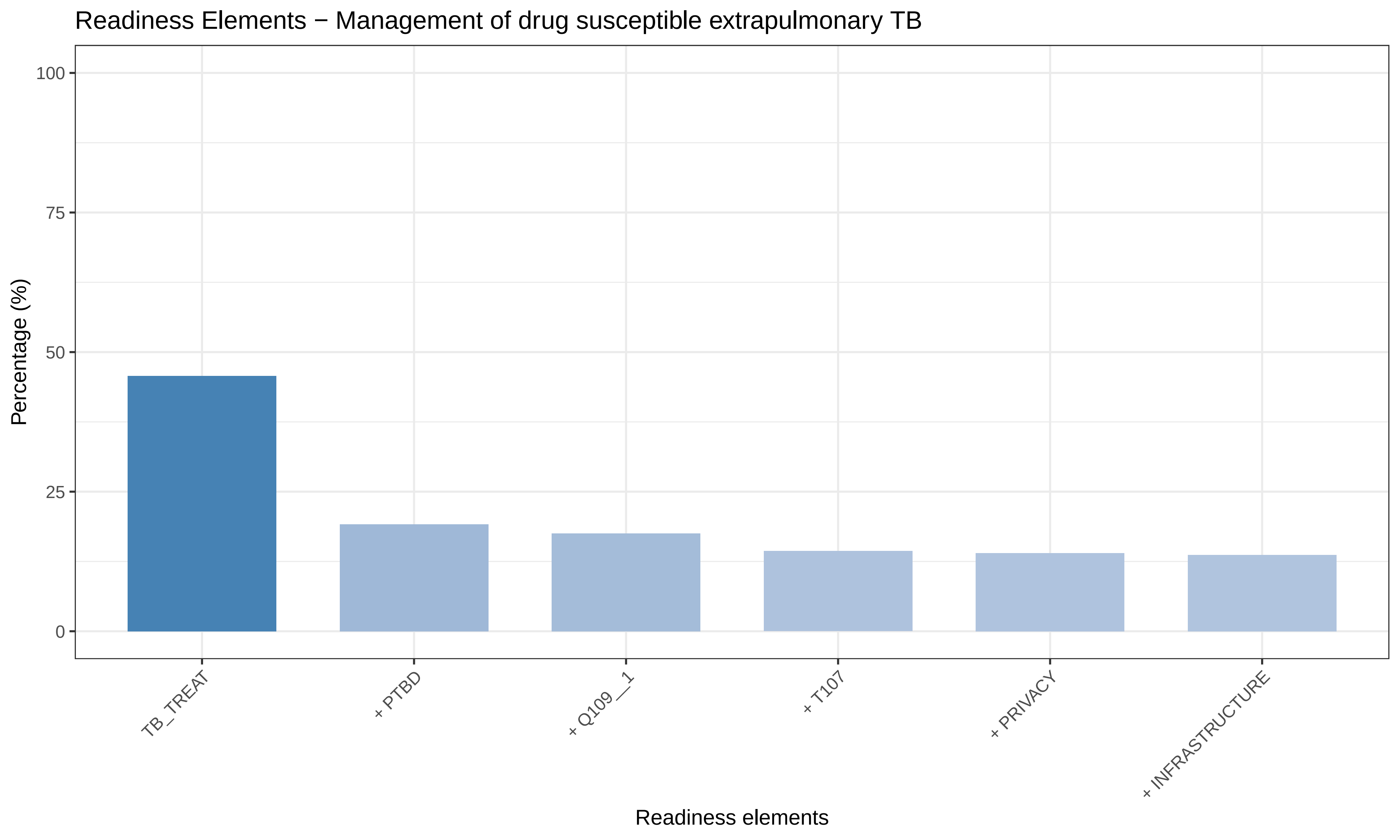
Readiness Elements − Management of drug susceptible extrapulmonary TB
Percentage (%)
100
75
50
25
0
TB_TREAT
+ PTBD
+ Q109__1
+ T107
+ PRIVACY
+ INFRASTRUCTURE
Readiness elements

Readiness Elements − Intermittent malaria prevention in infancy

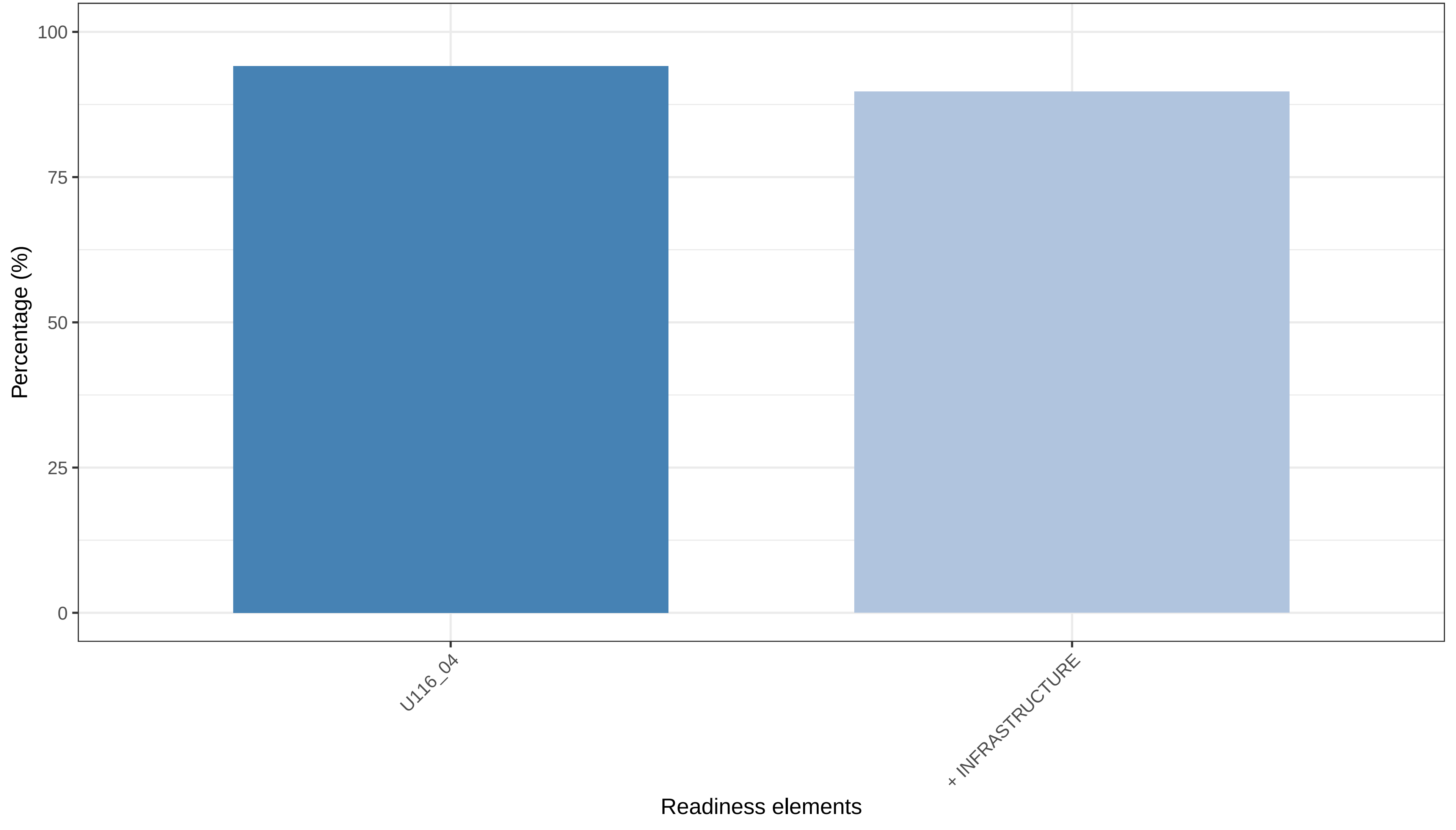

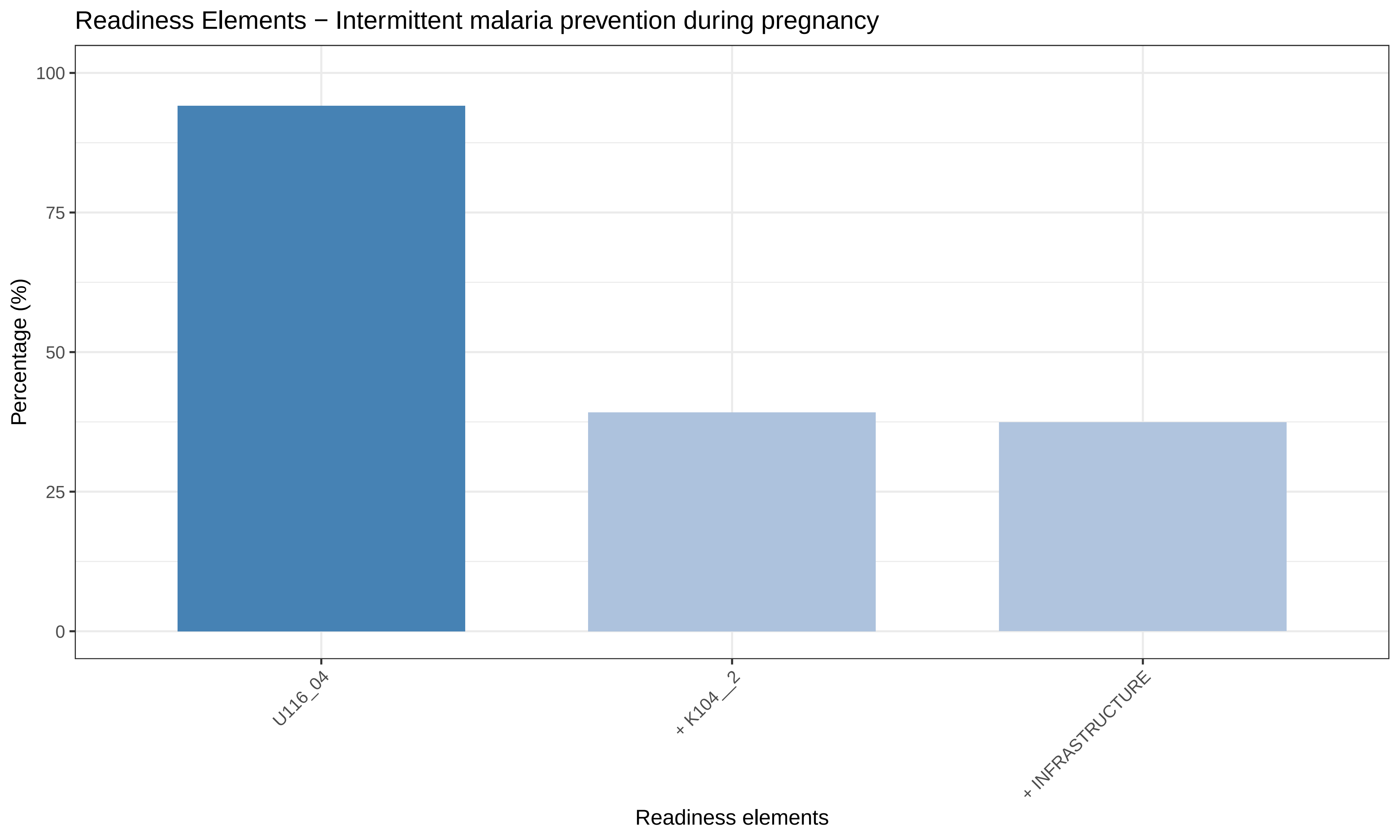
Readiness Elements − Intermittent malaria prevention during pregnancy
Percentage (%)
100
75
50
25
0
U116_04
+ K104__2
+ INFRASTRUCTURE
Readiness elements

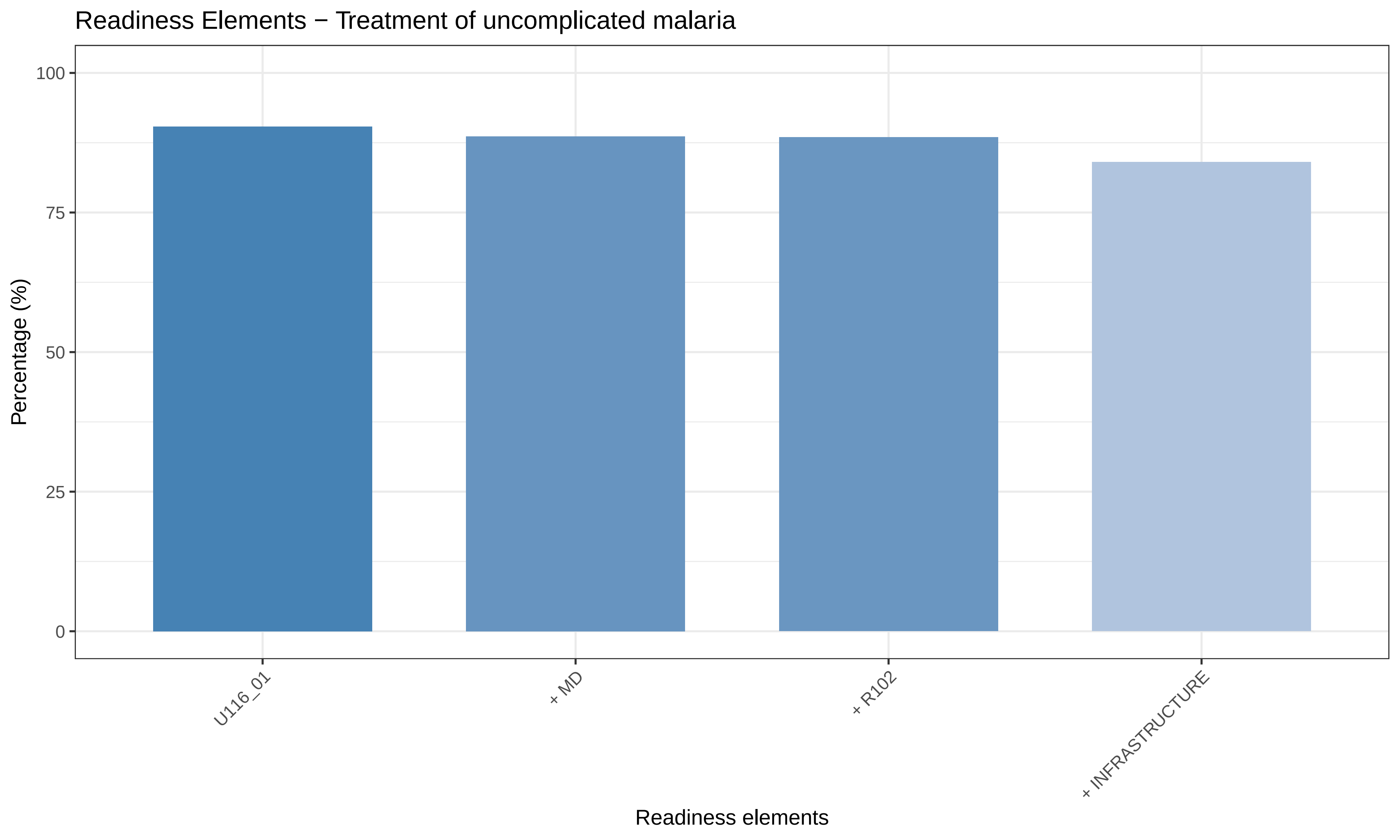
Readiness Elements − Treatment of uncomplicated malaria
100
75
50
25
0
Percentage (%)
U116_01
+ MD
+ R102
+ INFRASTRUCTURE
Readiness elements

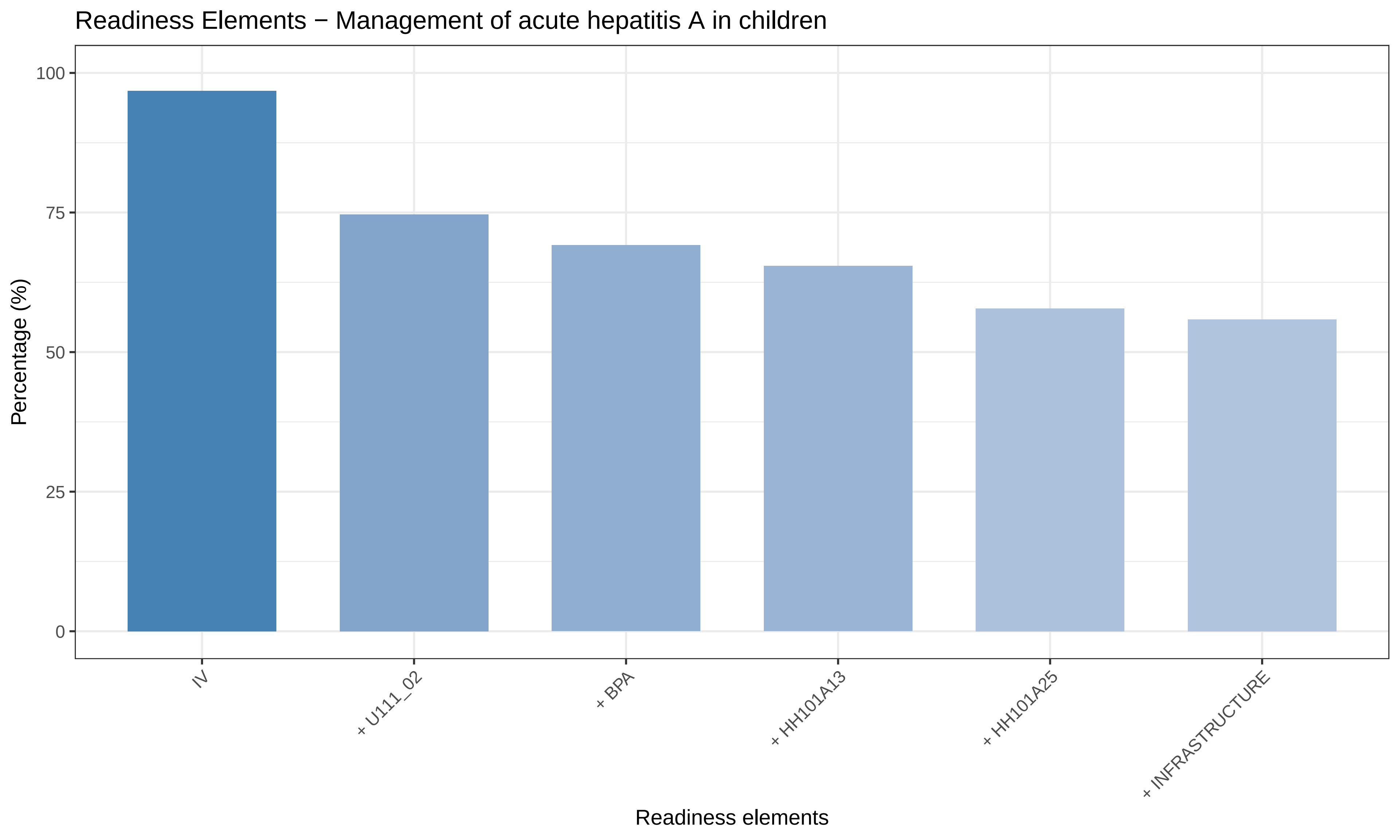
Readiness Elements − Management of acute hepatitis A in children
Percentage (%)
100
75
50
25
0
IV
+ U111_02
+ BPA
+ HH101A13
+ HH101A25
+ INFRASTRUCTURE
Readiness elements

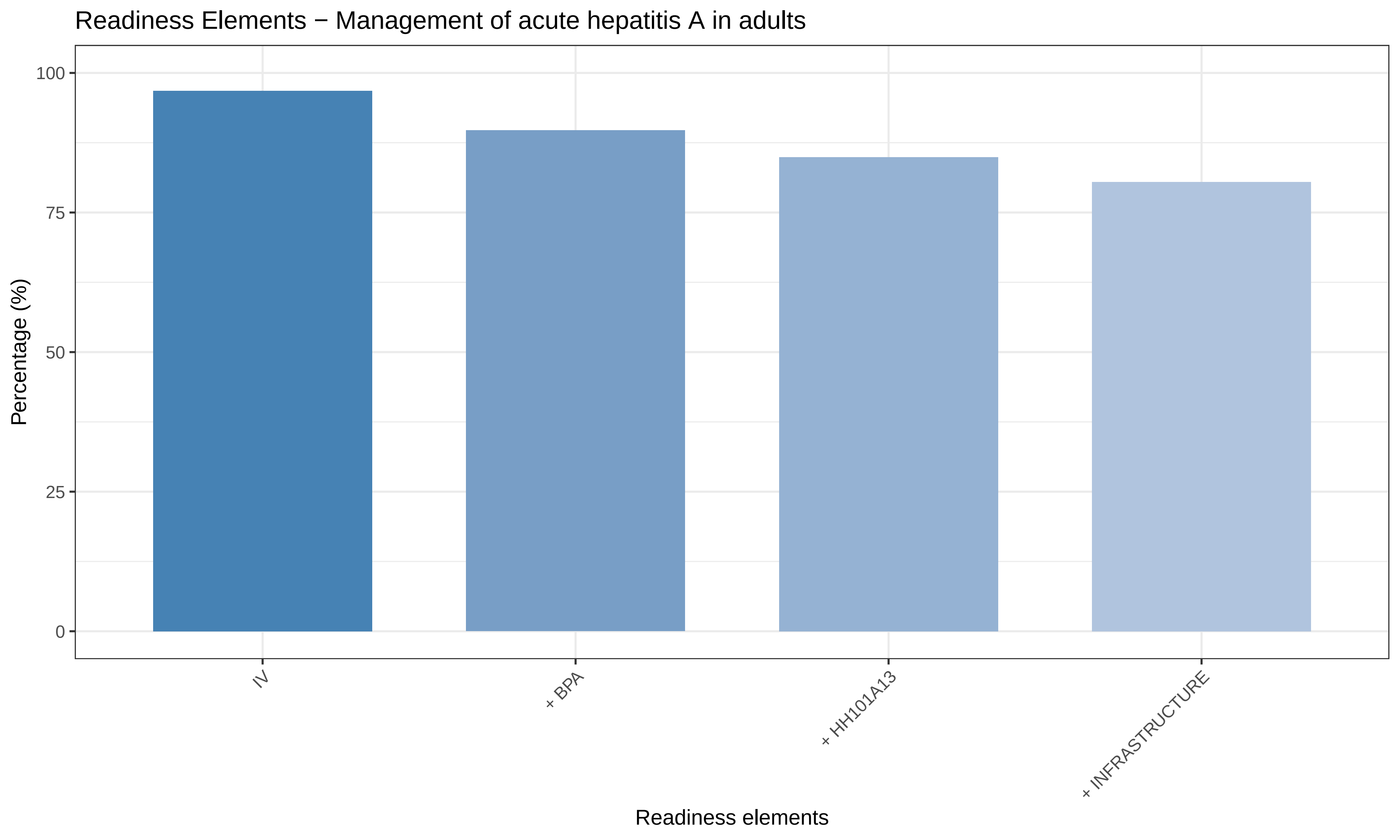
Readiness Elements − Management of acute hepatitis A in adults
Percentage (%)
100
75
50
25
0
IV
+ BPA
+ HH101A13
+ INFRASTRUCTURE
Readiness elements

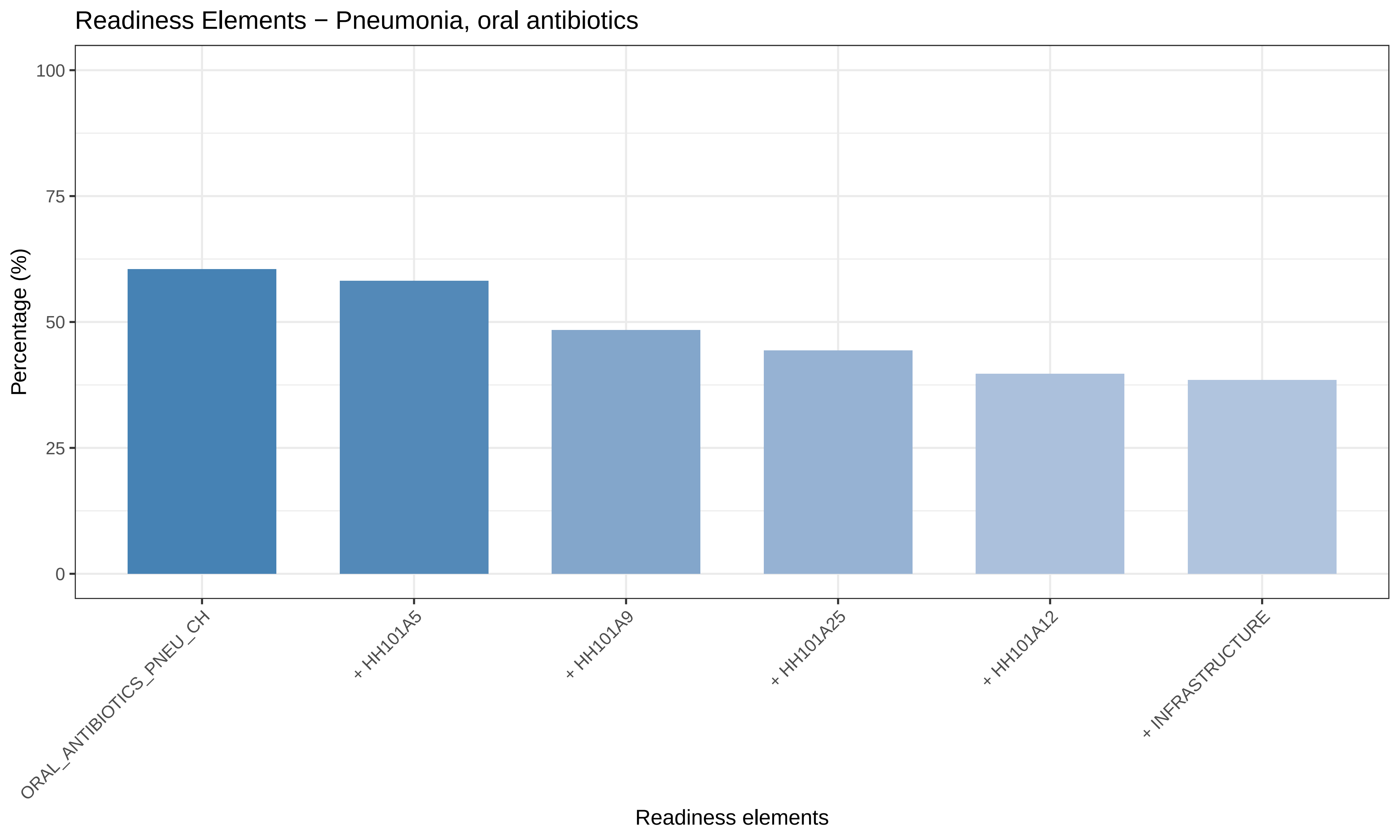
Readiness Elements − Pneumonia, oral antibiotics
Percentage (%)
0
25
50
75
100
ORAL_ANTIBIOTICS_PNEU_CH
+ HH101A5
+ HH101A9
+ HH101A25
+ HH101A12
+ INFRASTRUCTURE
Readiness elements

Readiness Elements − Pneumonia (severe), IV antibiotics

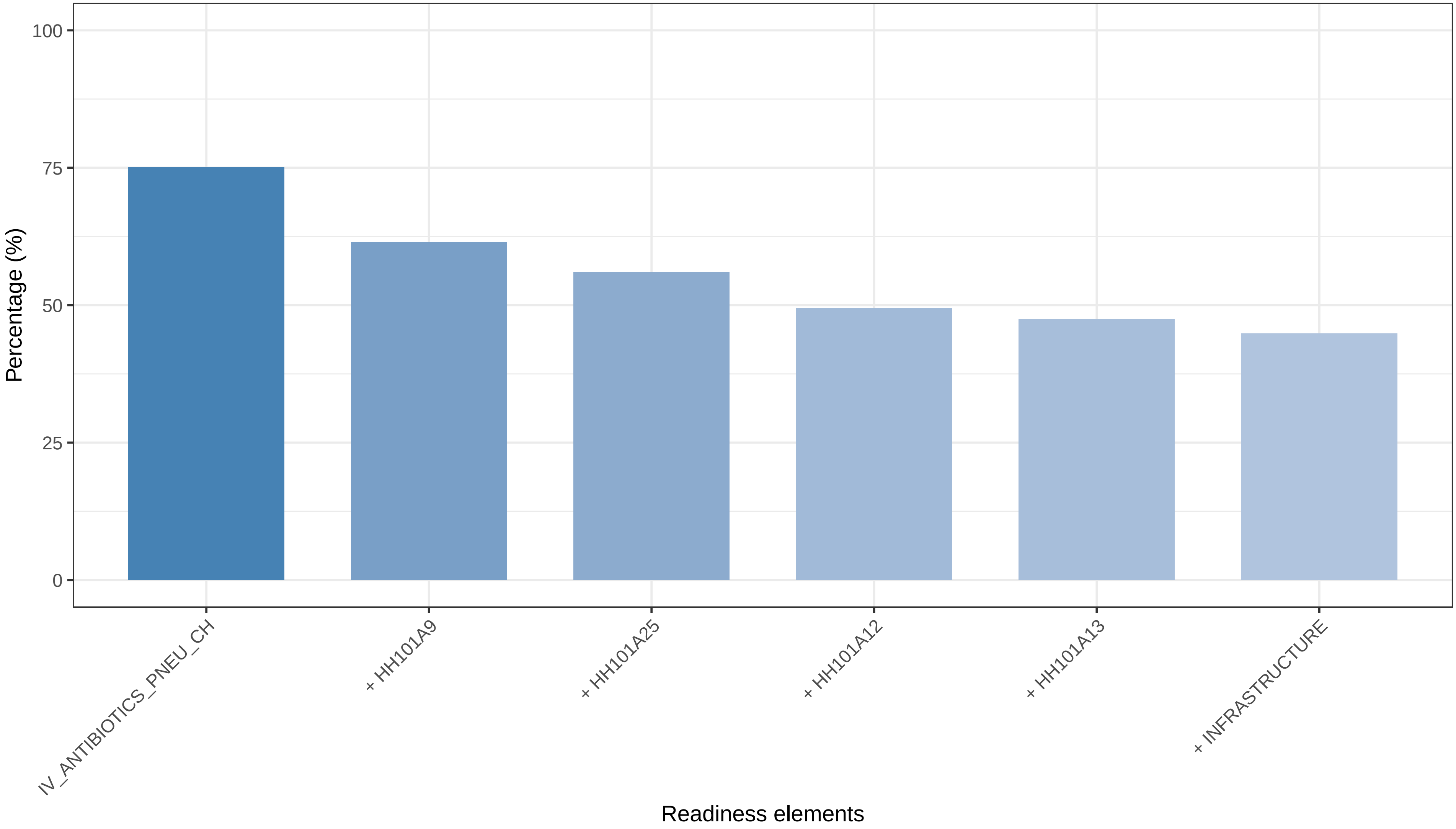

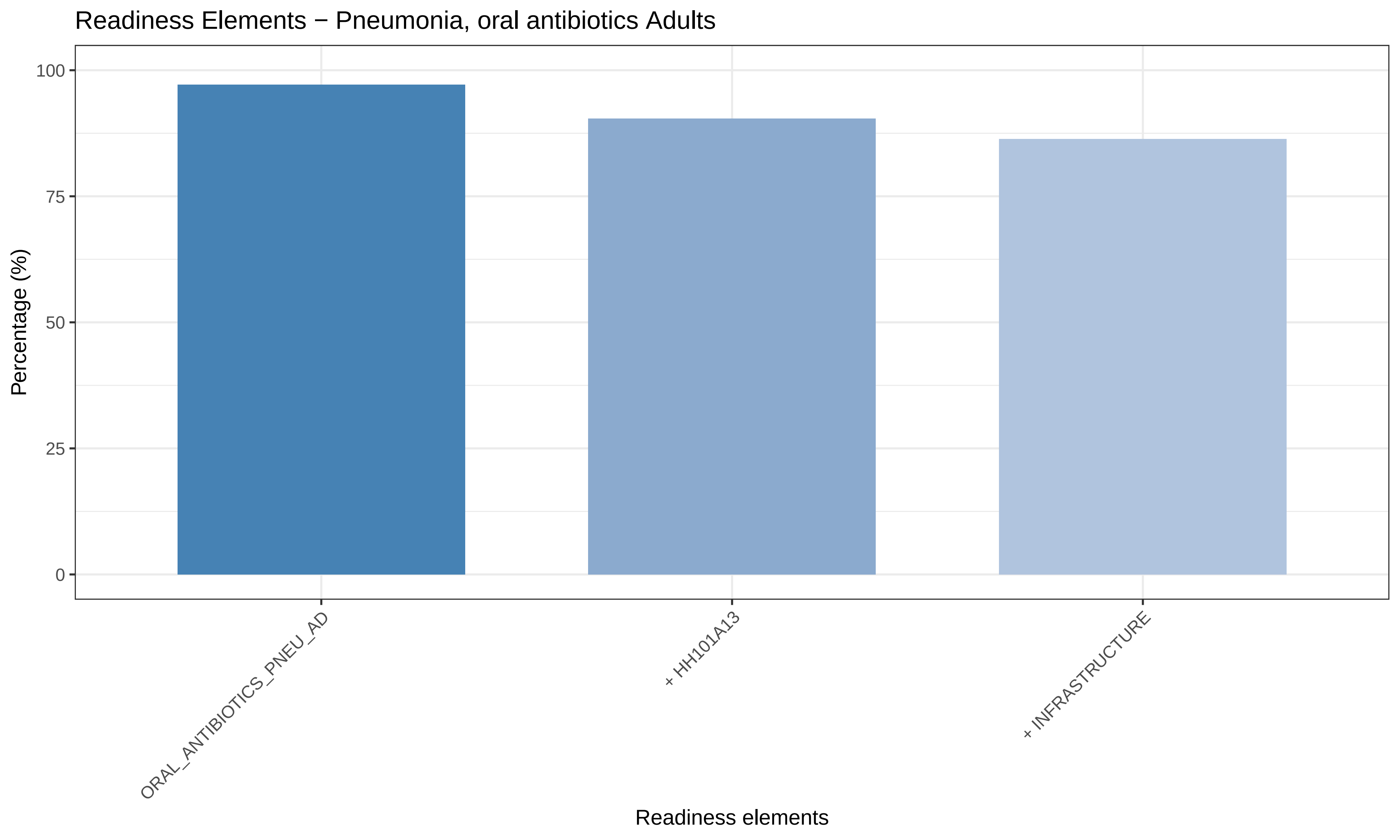
Readiness Elements − Pneumonia, oral antibiotics Adults
Percentage (%)
100
75
50
25
0
ORAL_ANTIBIOTICS_PNEU_AD
+ HH101A13
+ INFRASTRUCTURE
Readiness elements

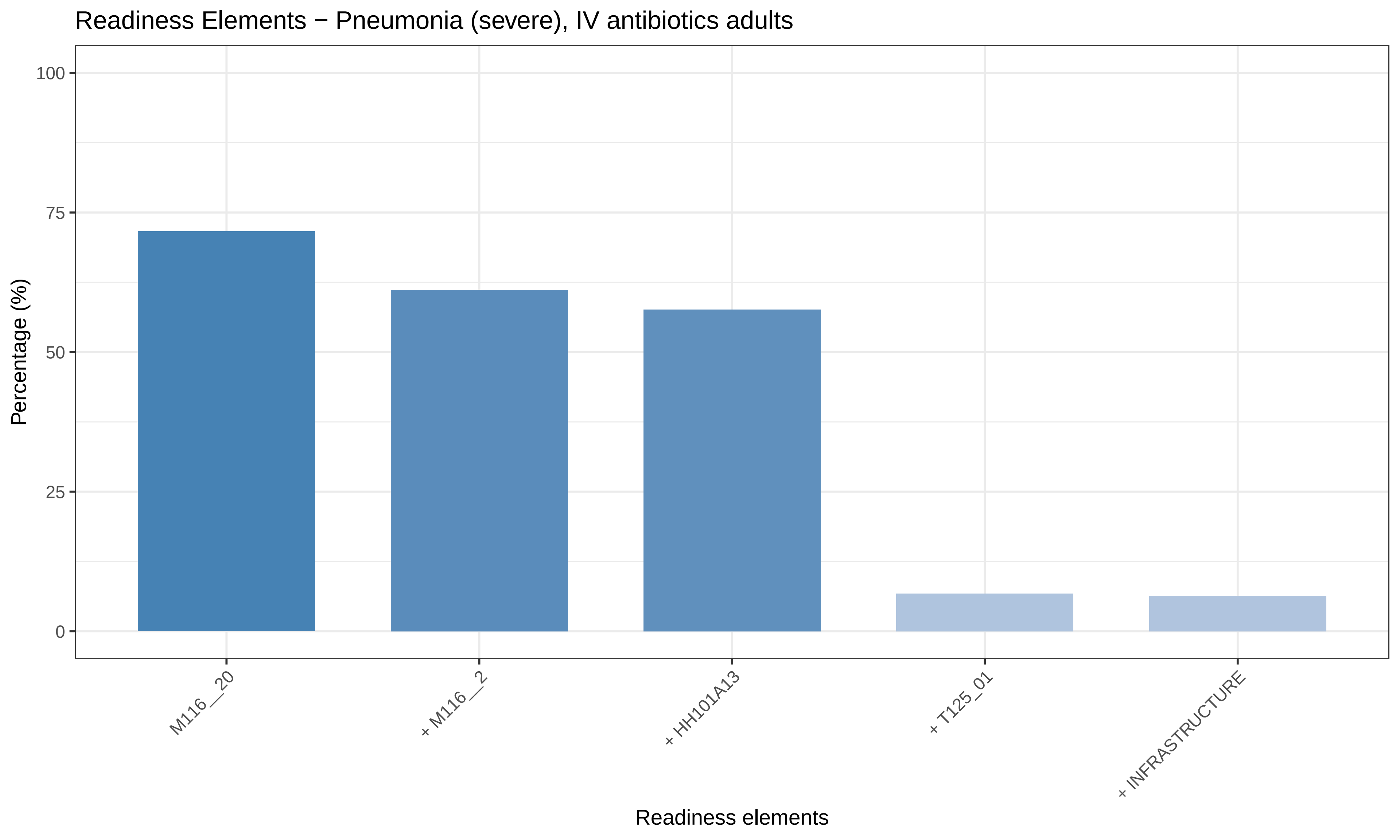

Readiness Elements − Pneumonia (severe), IV antibiotics adults
Percentage (%)
100
75
50
25
0
M116__20
+ M116__2
+ HH101A13
+ T125_01
+ INFRASTRUCTURE
Readiness elements

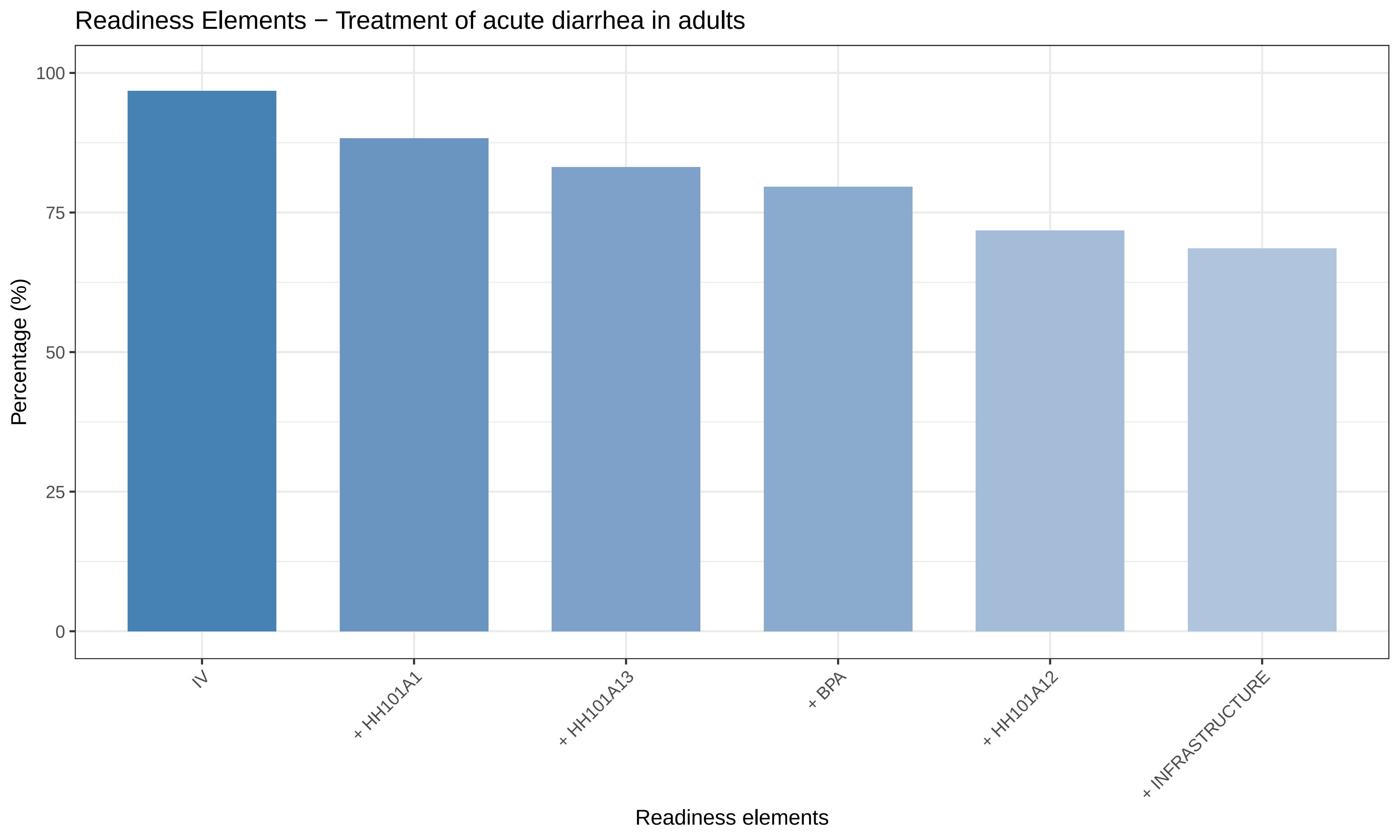
Readiness Elements − Treatment of acute diarrhea in adults
Percentage (%)
0
25
50
75
100
IV
+ HH101A1
+ HH101A13
+ BPA
+ HH101A12
+ INFRASTRUCTURE
Readiness elements

Readiness Elements − Treatment of acute diarrhea in children

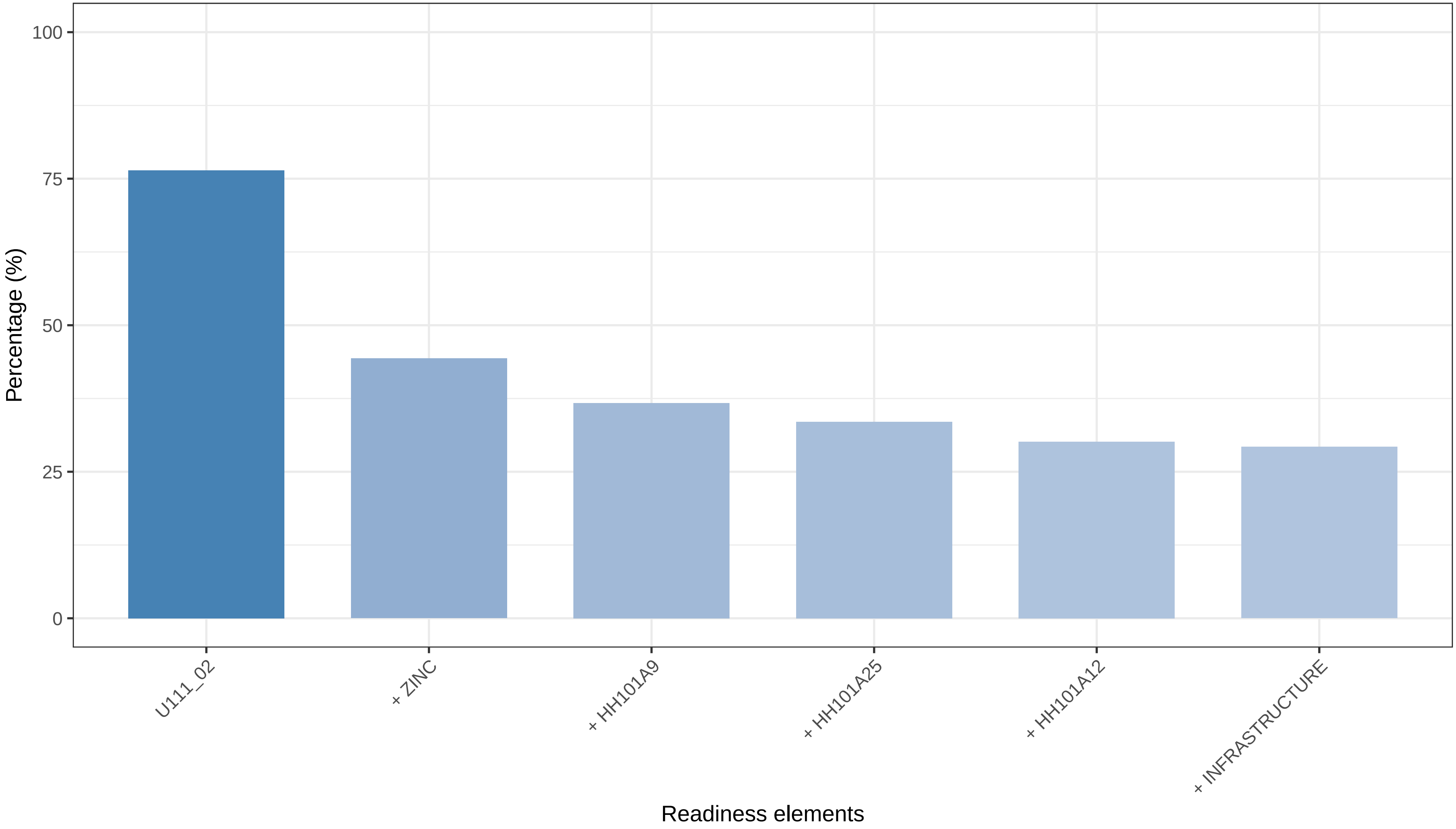

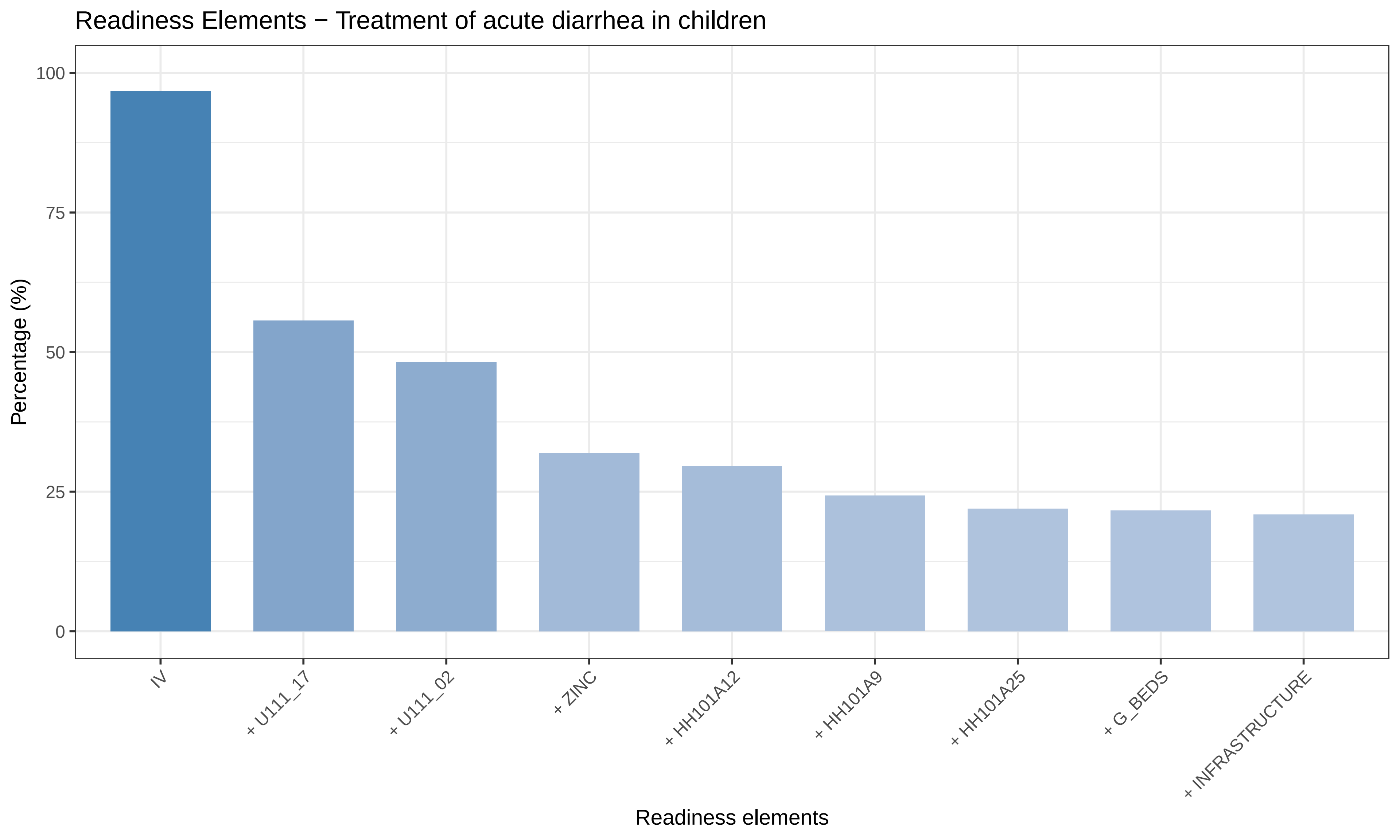

Readiness Elements − Treatment of acute diarrhea in children
Percentage (%)
100
75
50
25
0
IV
+ U111_17
+ U111_02
+ ZINC
+ HH101A12
+ HH101A9
+ HH101A25
+ G_BEDS
+ INFRASTRUCTURE
Readiness elements

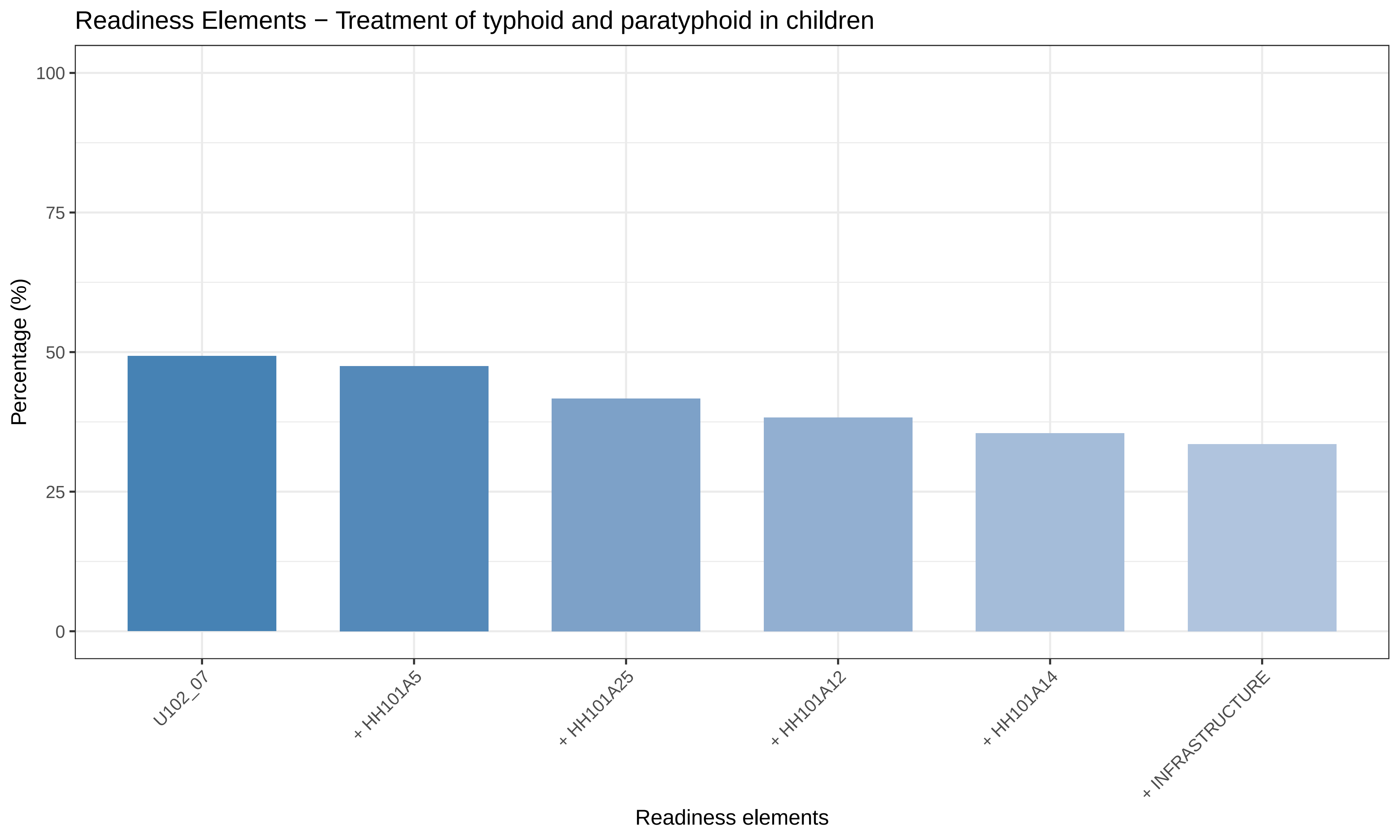

Readiness Elements − Treatment of typhoid and paratyphoid in children
Percentage (%)
100
75
50
25
0
U102_07
+ HH101A5
+ HH101A25
+ HH101A12
+ HH101A14
+ INFRASTRUCTURE
Readiness elements

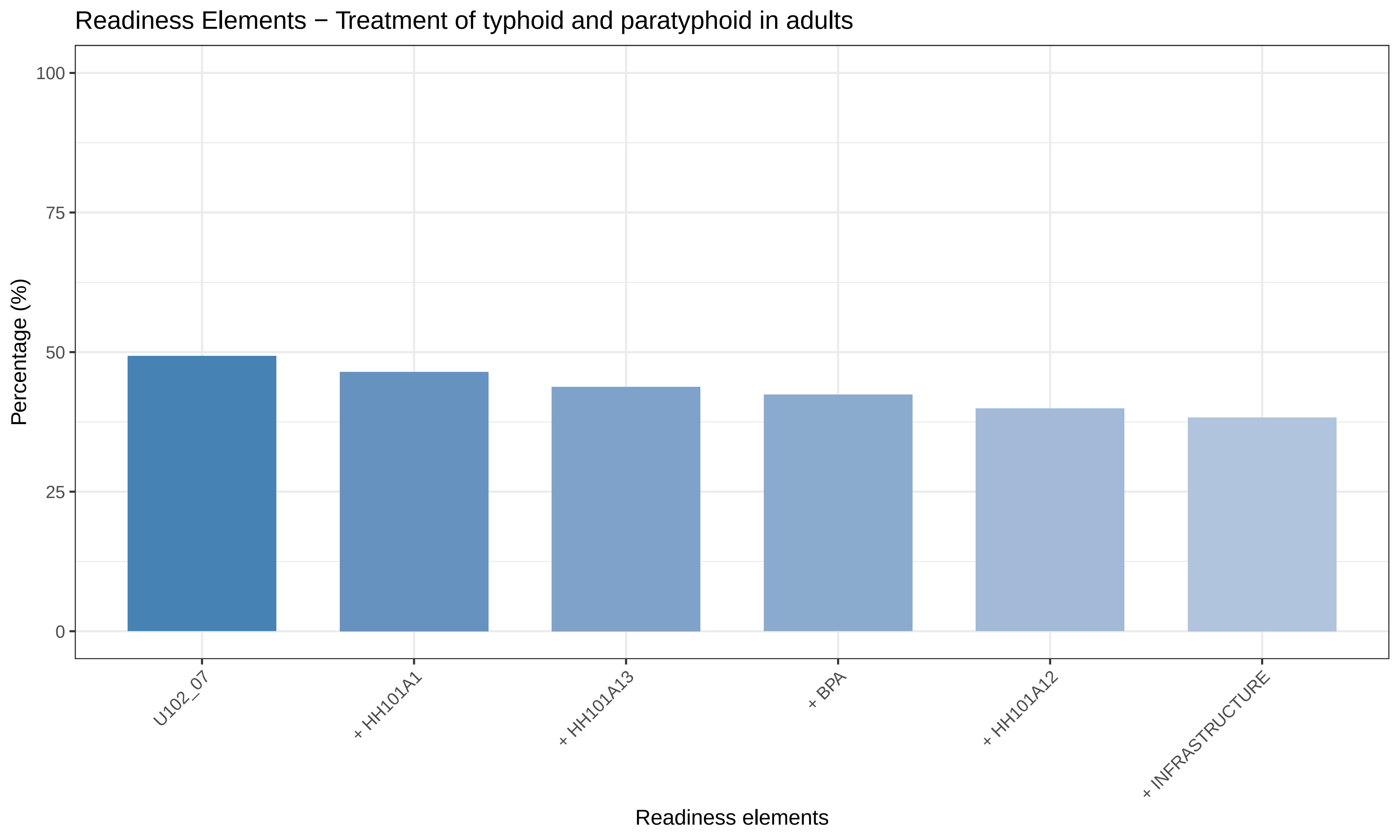
Readiness Elements − Treatment of typhoid and paratyphoid in adults
Percentage (%)
100
75
50
25
0
U102_07
+ HH101A1
+ HH101A13
+ BPA
+ HH101A12
+ INFRASTRUCTURE
Readiness elements

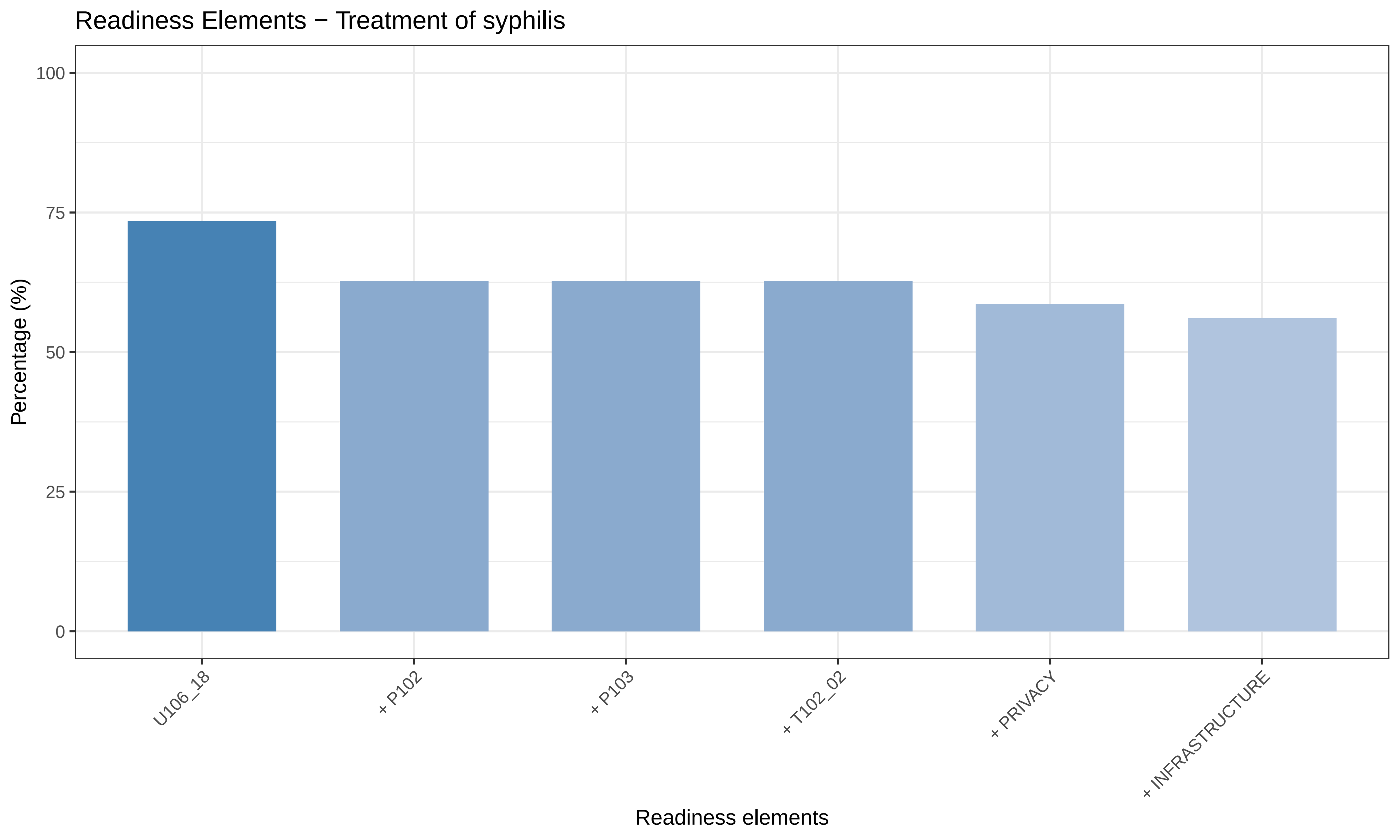
Readiness Elements − Treatment of syphilis
Percentage (%)
100
75
50
25
0
U106_18
+ P102
+ P103
+ T102_02
+ PRIVACY
+ INFRASTRUCTURE
Readiness elements

Readiness Elements − Treatment of gonorrhea

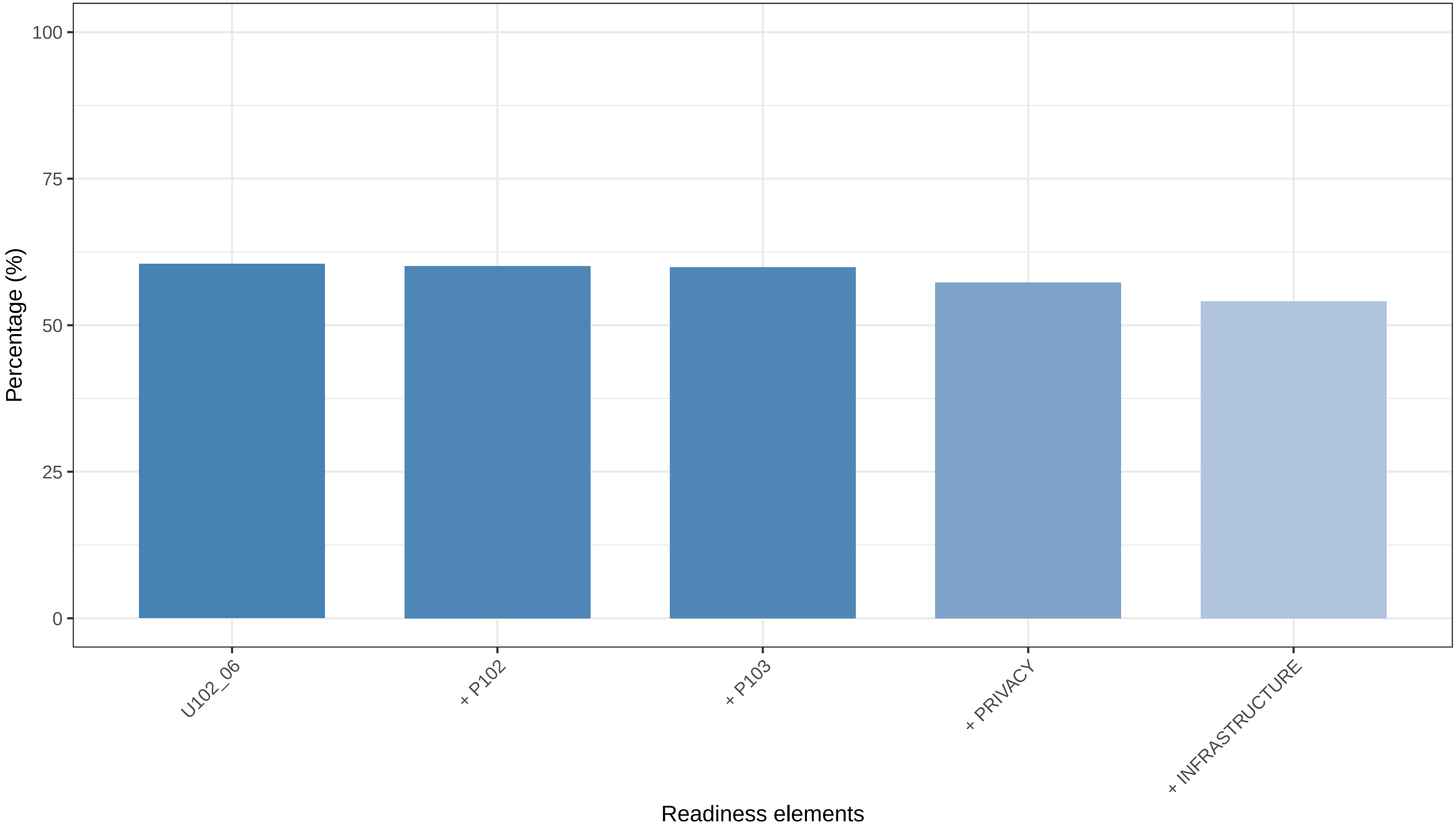

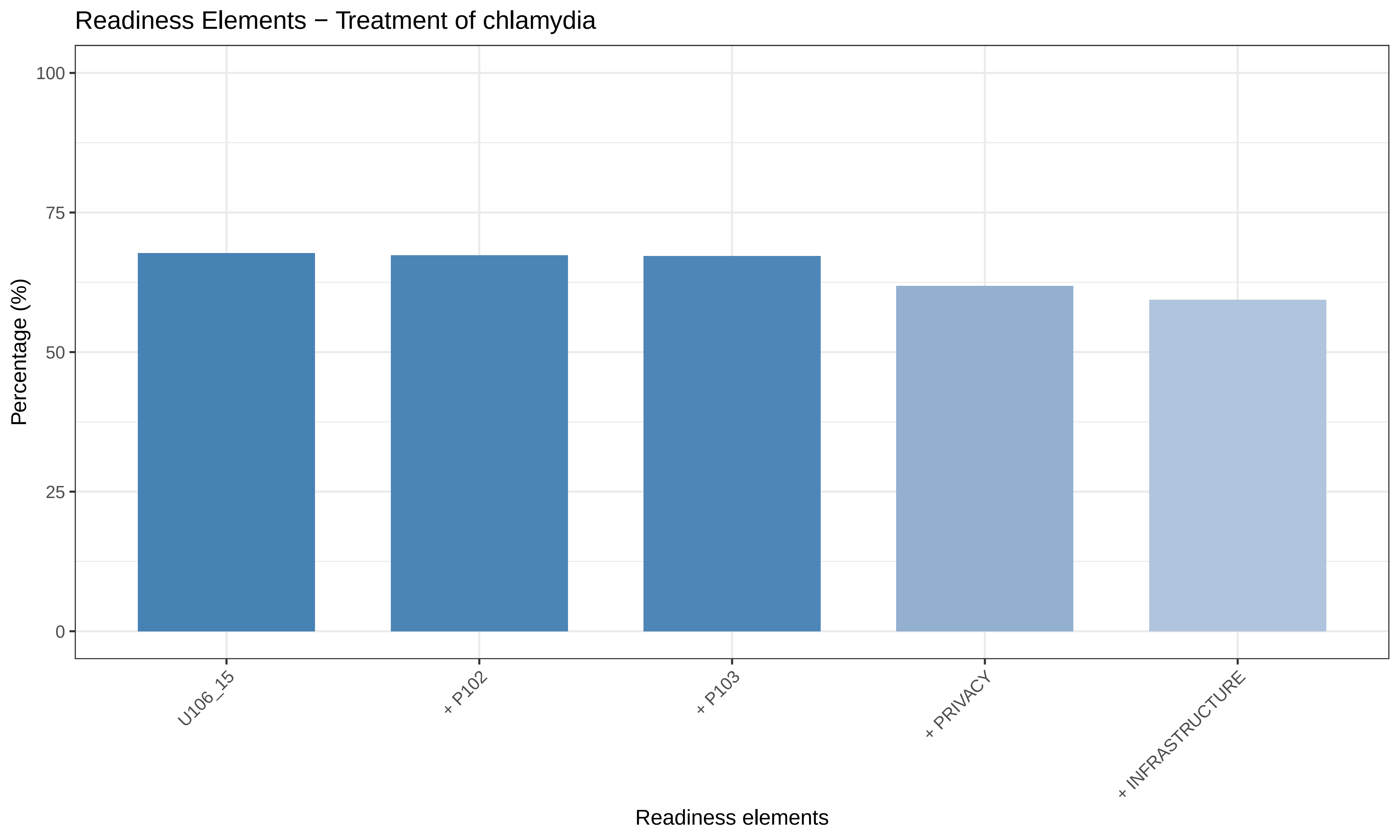
Readiness Elements − Treatment of chlamydia
Percentage (%)
100
75
50
25
0
U106_15
+ P102
+ P103
+ PRIVACY
+ INFRASTRUCTURE
Readiness elements

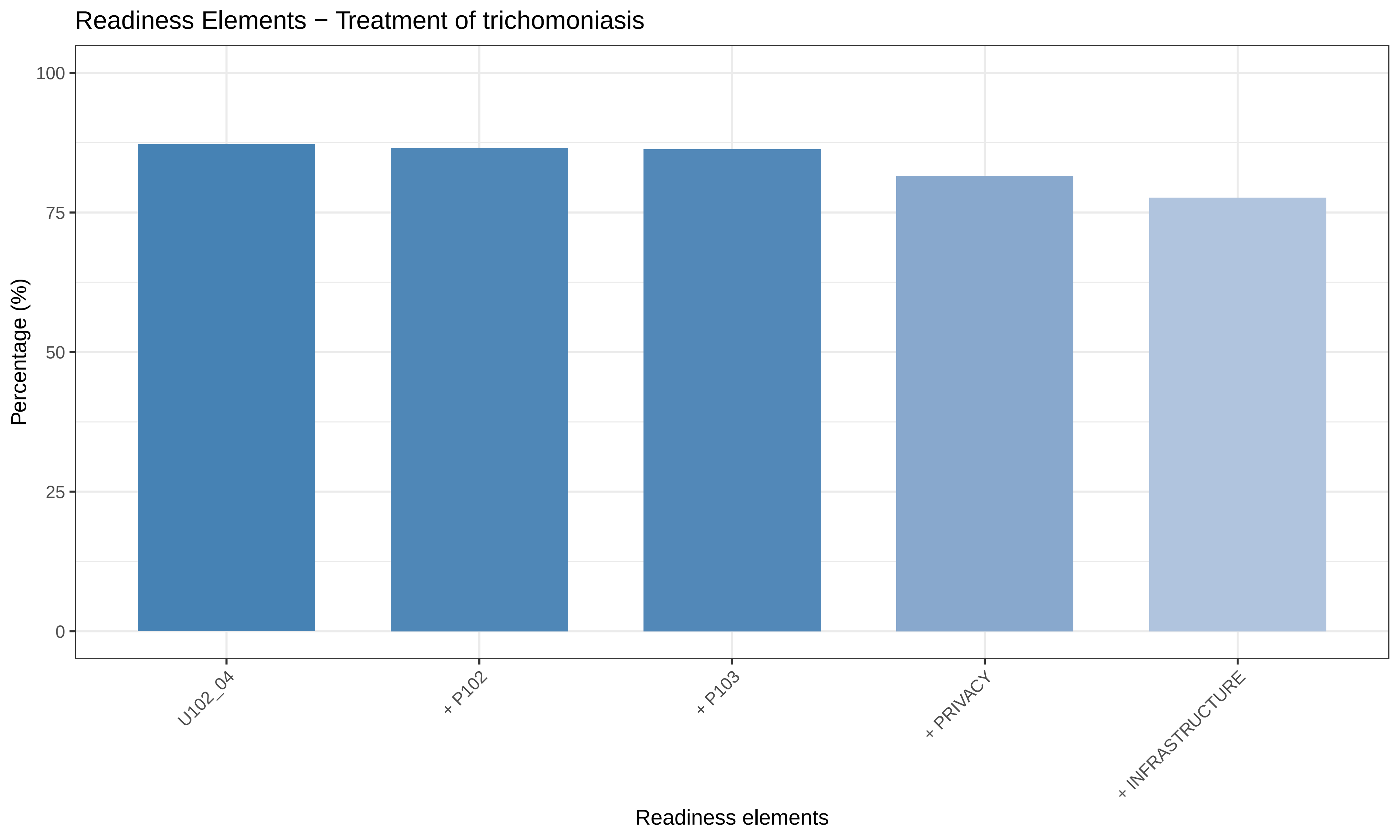
Readiness Elements − Treatment of trichomoniasis
Percentage (%)
100
75
50
25
0
U102_04
+ P102
+ P103
+ PRIVACY
+ INFRASTRUCTURE
Readiness elements

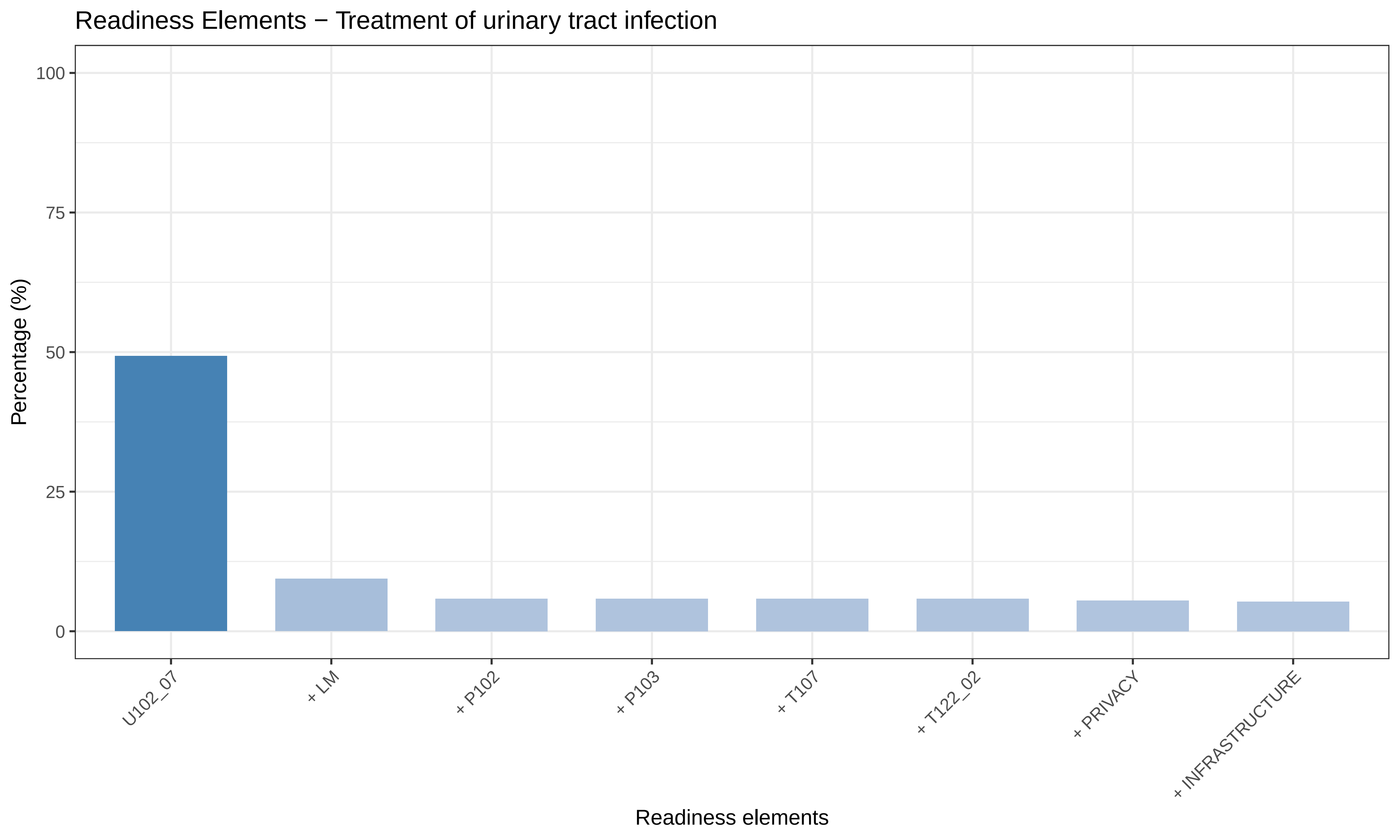

Readiness Elements − Treatment of urinary tract infection
Percentage (%)
100
75
50
25
0
U102_07
+ LM
+ P102
+ P103
+ T107
+ T122_02
+ PRIVACY
+ INFRASTRUCTURE
Readiness elements

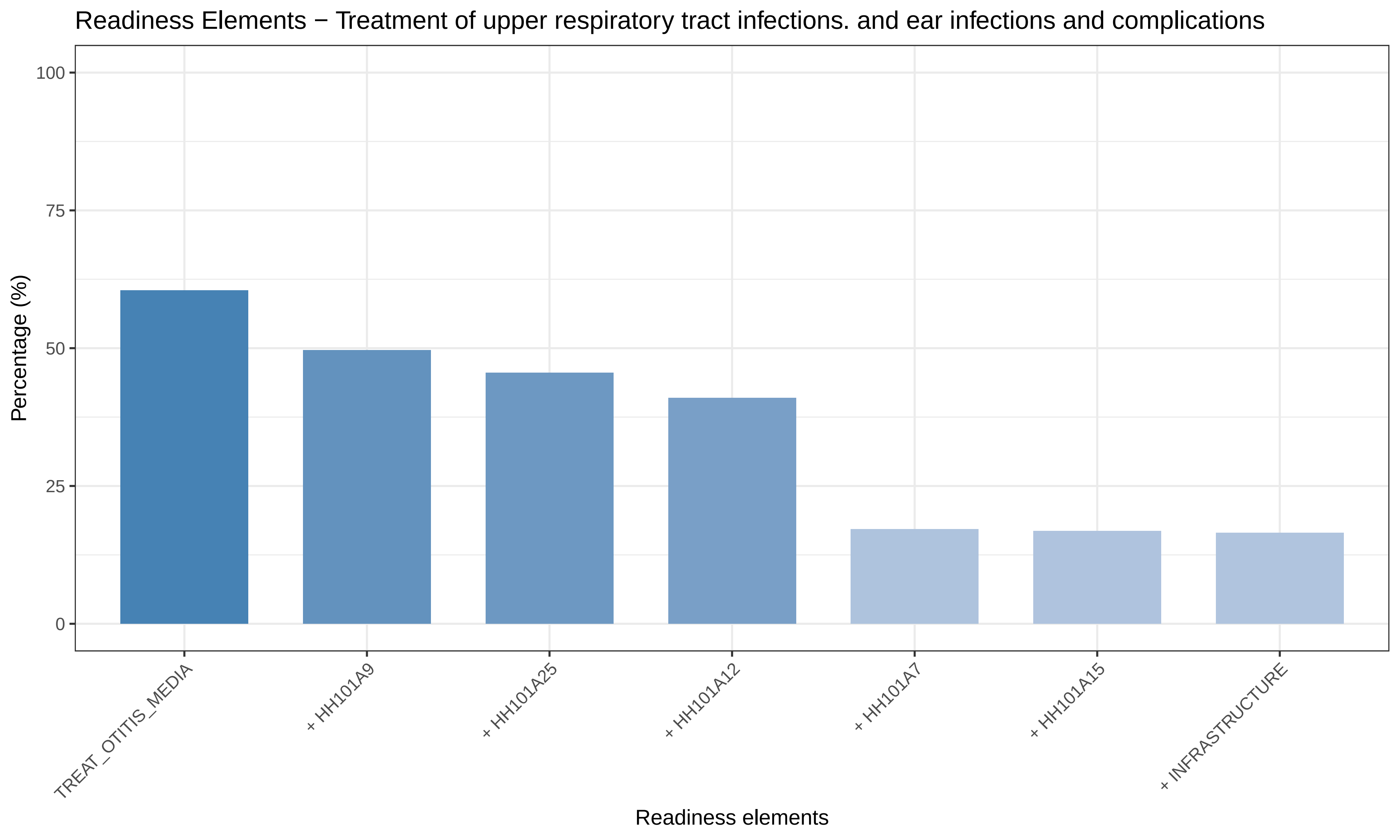
Readiness Elements − Treatment of upper respiratory tract infections. and ear infections and complications
Percentage (%)
100
75
50
25
0
TREAT_OTITIS_MEDIA
+ HH101A9
+ HH101A25
+ HH101A12
+ HH101A7
+ HH101A15
+ INFRASTRUCTURE
Readiness elements

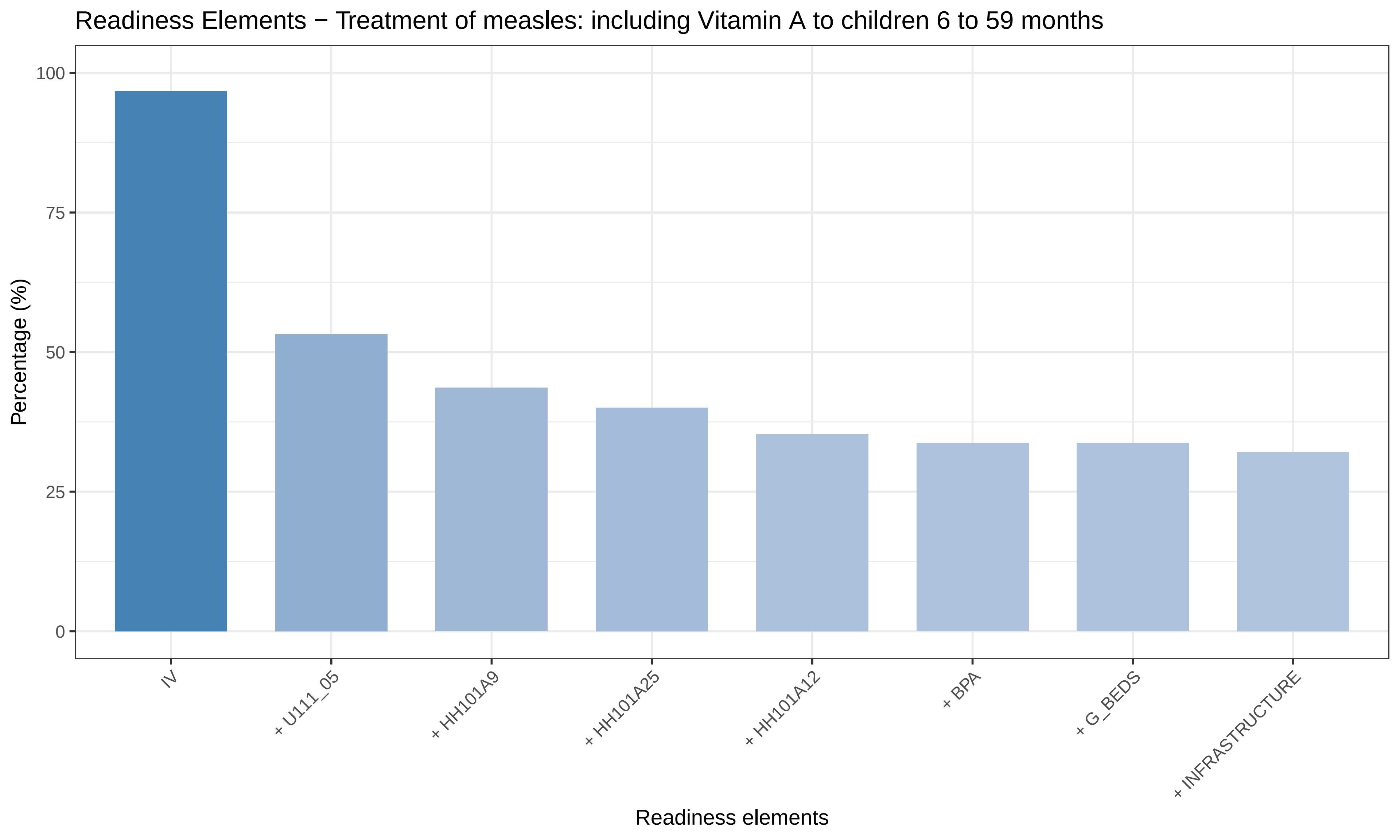

Readiness Elements − Treatment of measles: including Vitamin A to children 6 to 59 months
Percentage (%)
100
75
50
25
0
IV
+ U111_05
+ HH101A9
+ HH101A25
+ HH101A12
+ BPA
+ G_BEDS
+ INFRASTRUCTURE
Readiness elements

Readiness Elements − Fever evaluation, basic

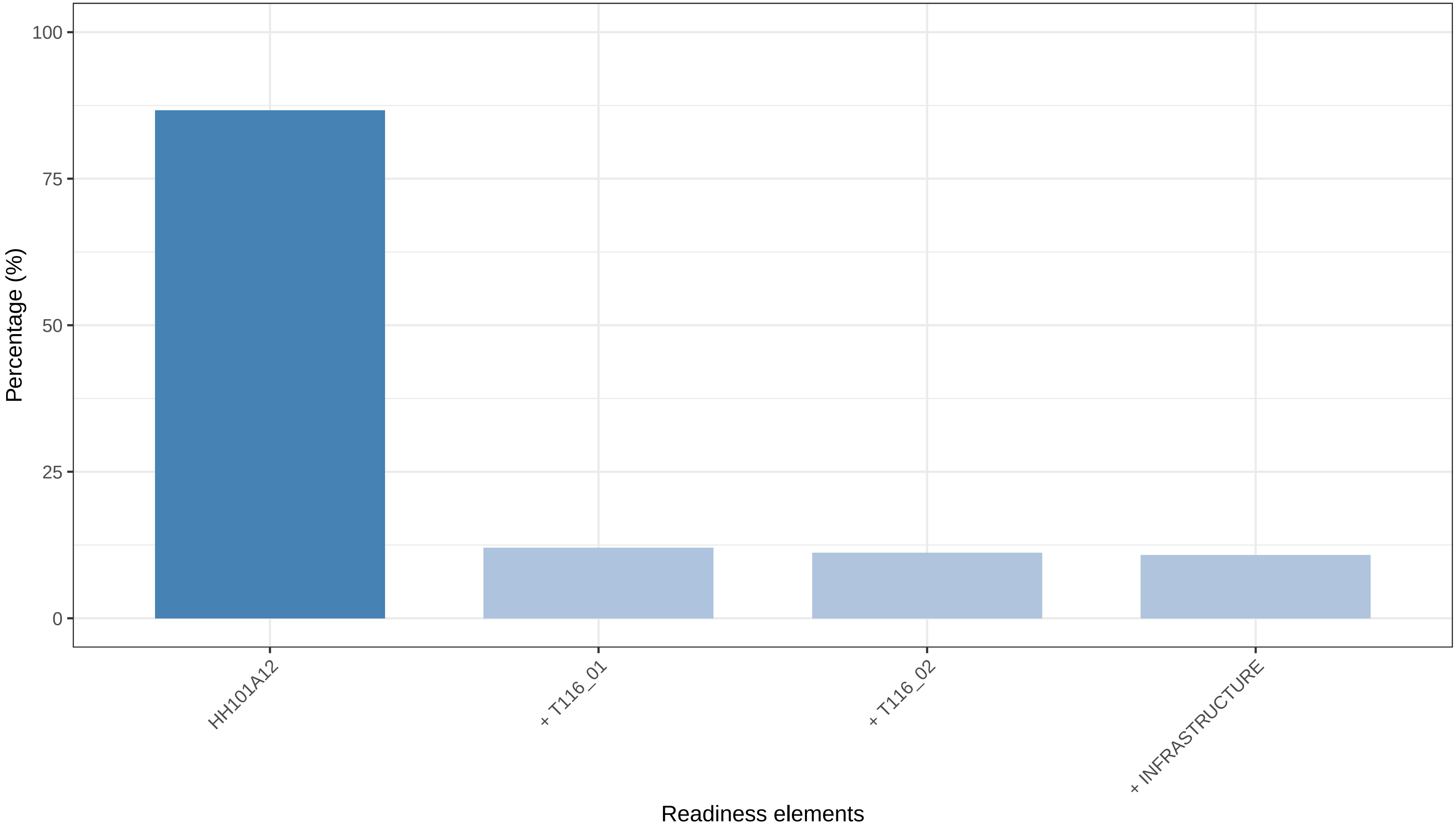

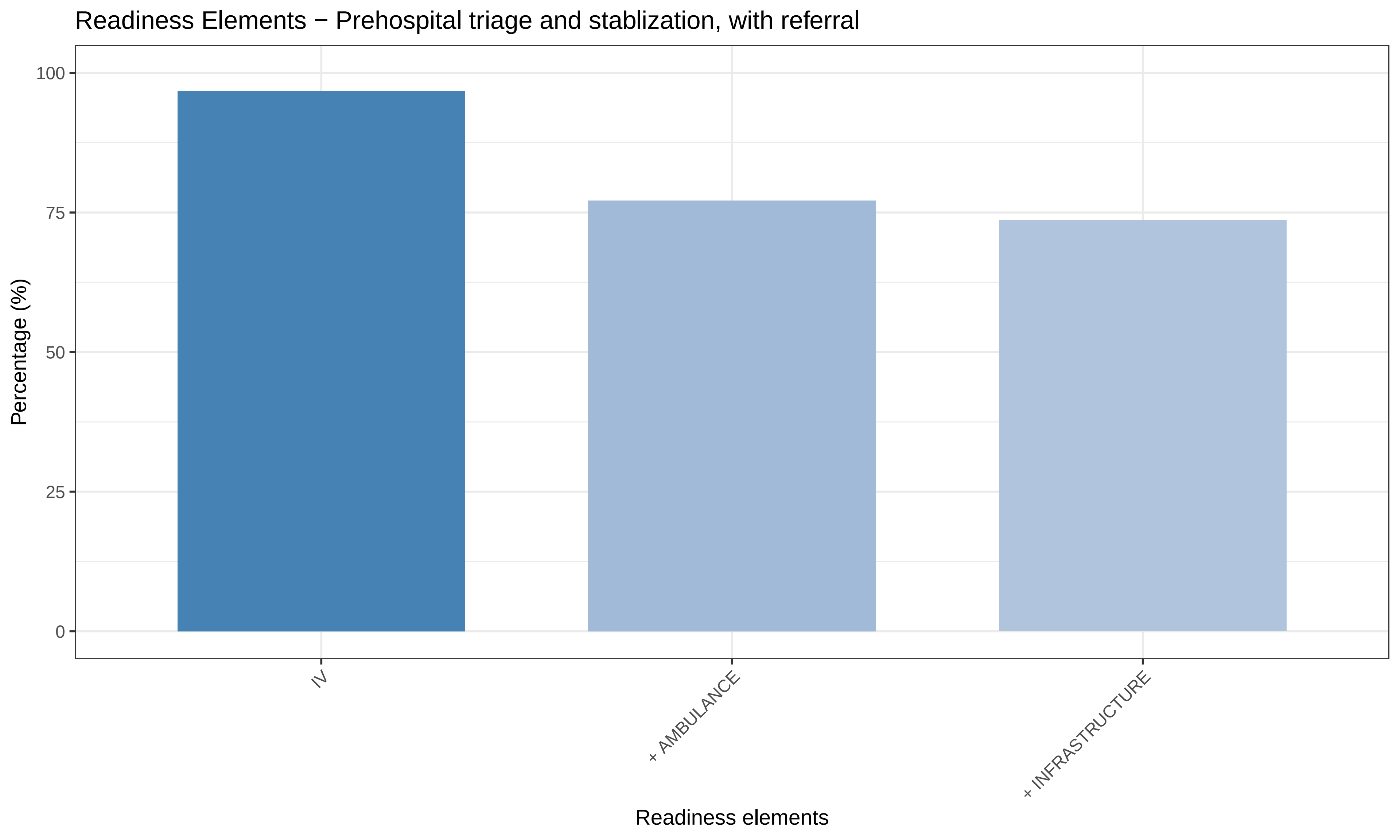
Readiness Elements − Prehospital triage and stablization, with referral
100
75
50
25
0
Percentage (%)
IV
+ AMBULANCE
+ INFRASTRUCTURE
Readiness elements

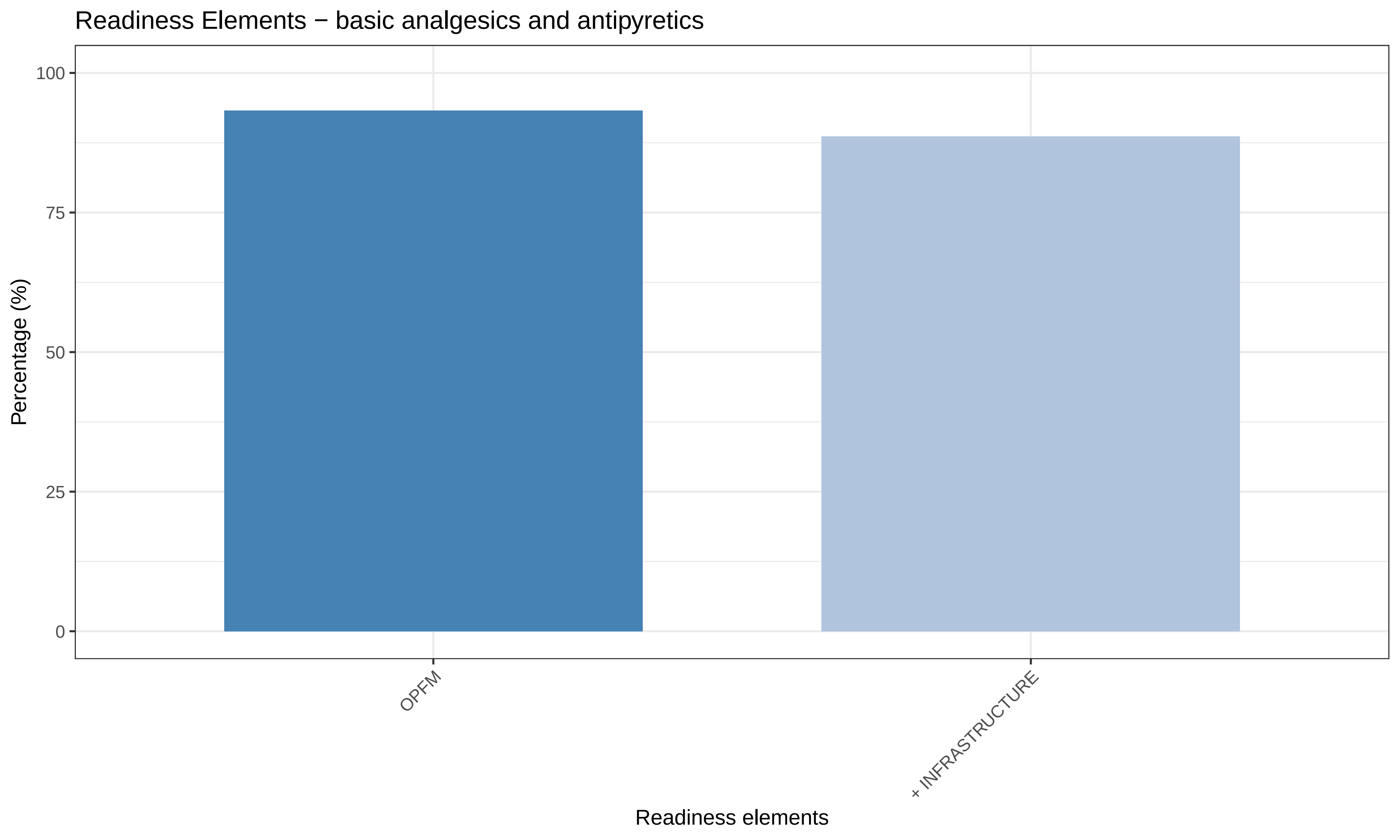
Readiness Elements − basic analgesics and antipyretics
Percentage (%)
100
75
50
25
0
OPFM
+ INFRASTRUCTURE
Readiness elements

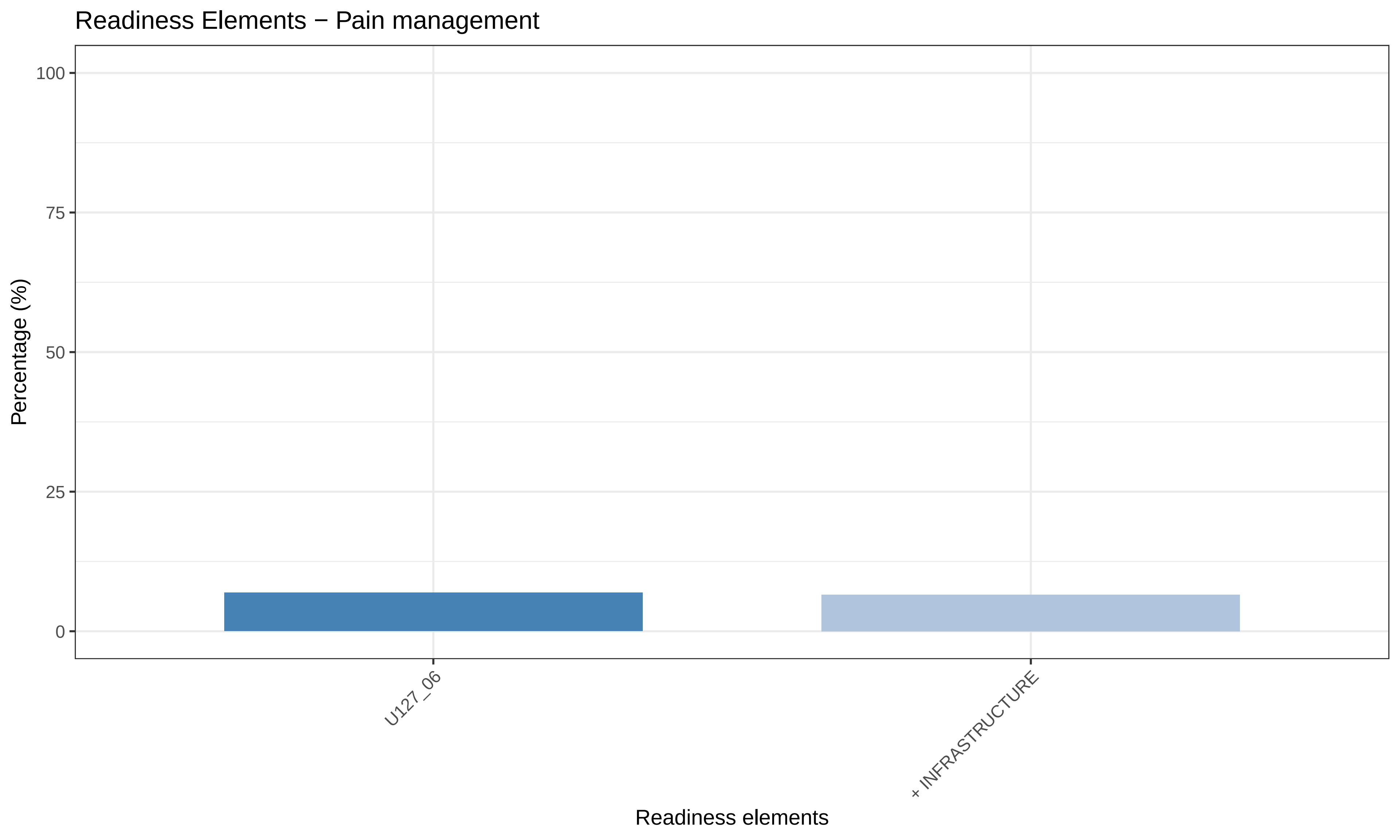
Readiness Elements − Pain management
Percentage (%)
100
75
50
25
0
U127_06
+ INFRASTRUCTURE
Readiness elements

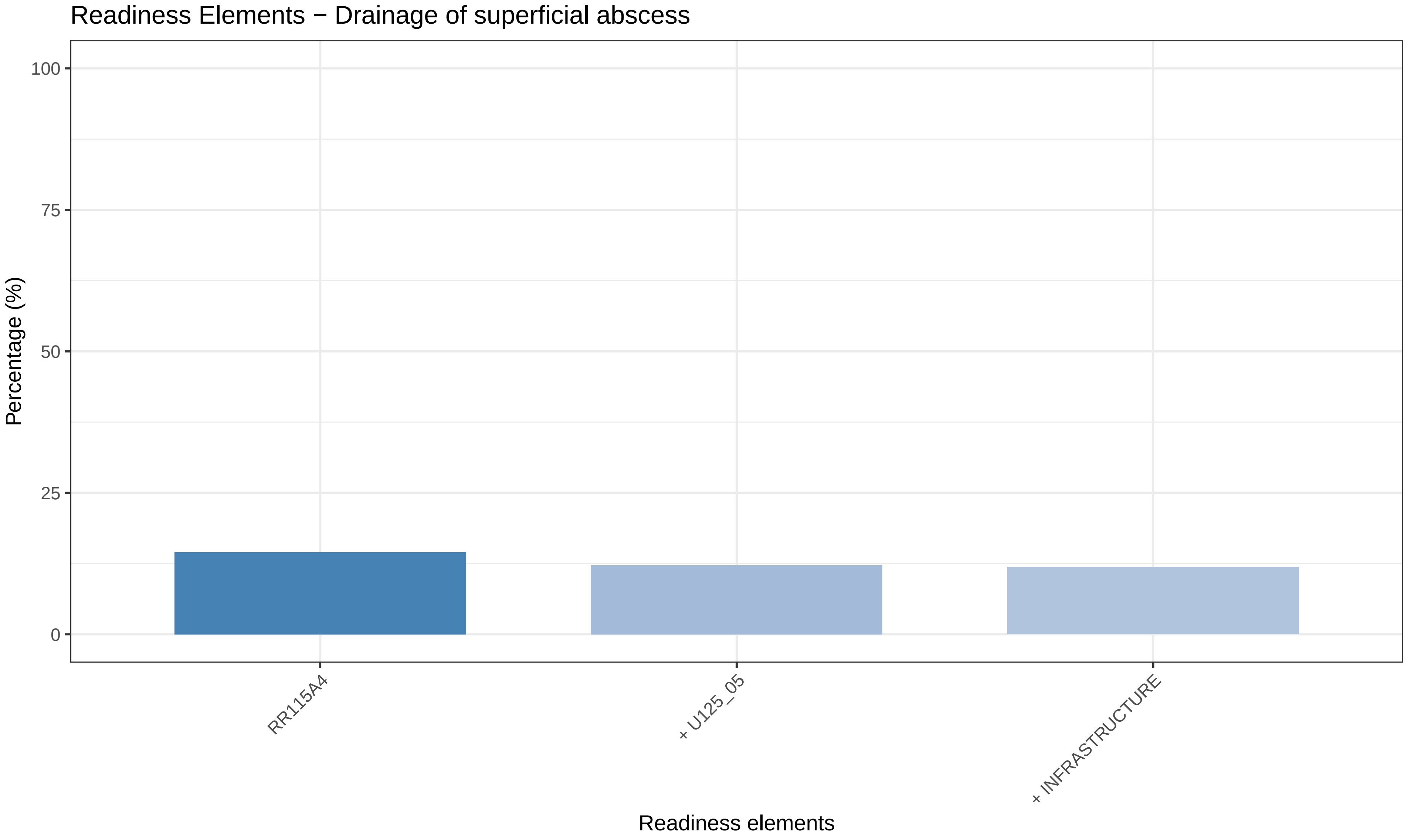

Readiness Elements − Drainage of superficial abscess
Percentage (%)
100
75
50
25
0
RR115A4
+ U125_05
+ INFRASTRUCTURE
Readiness elements

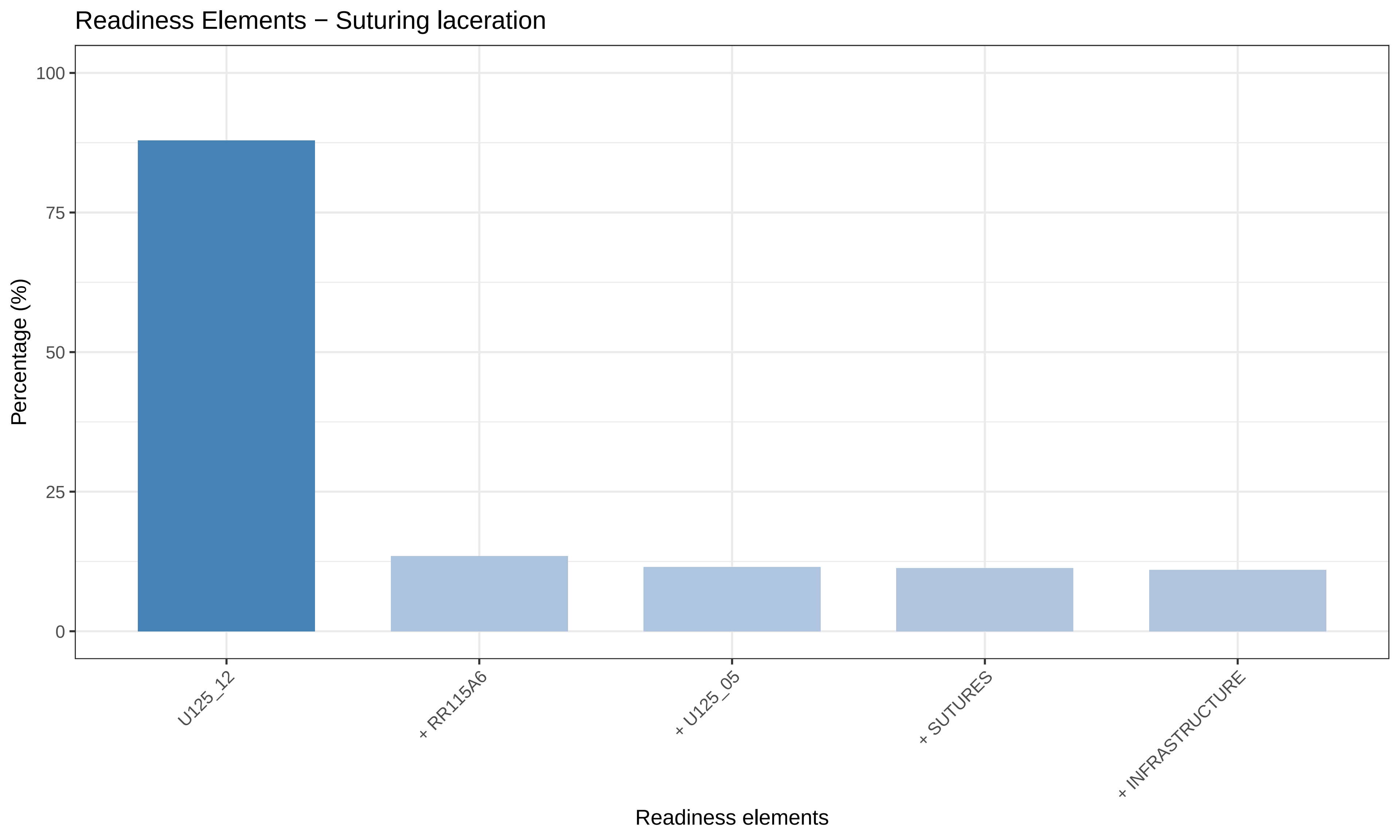

Readiness Elements − Suturing laceration
Percentage (%)
100
75
50
25
0
U125_12
+ RR115A6
+ U125_05
+ SUTURES
+ INFRASTRUCTURE
Readiness elements

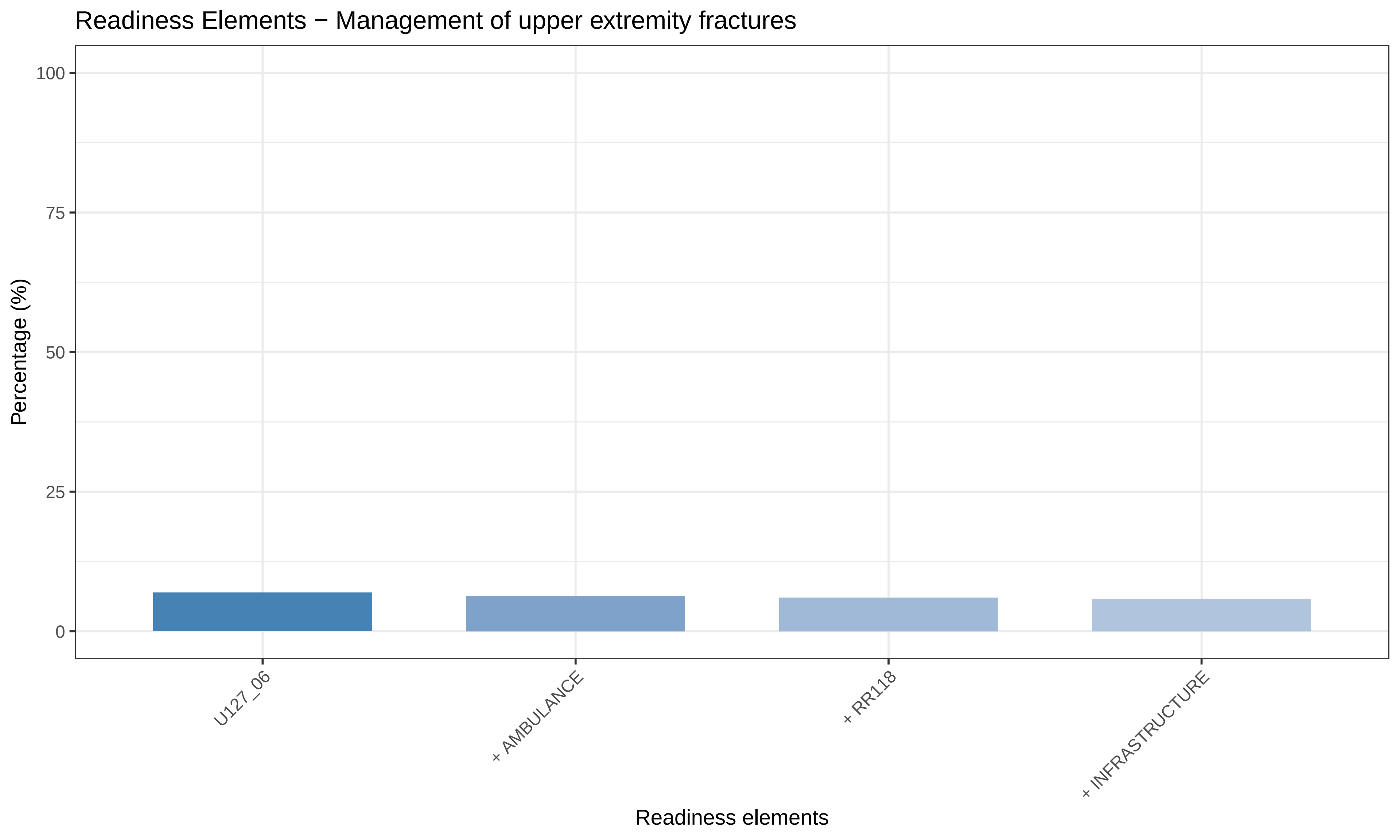

Readiness Elements − Management of upper extremity fractures
Percentage (%)
100
75
50
25
0
U127_06
+ AMBULANCE
+ RR118
+ INFRASTRUCTURE
Readiness elements

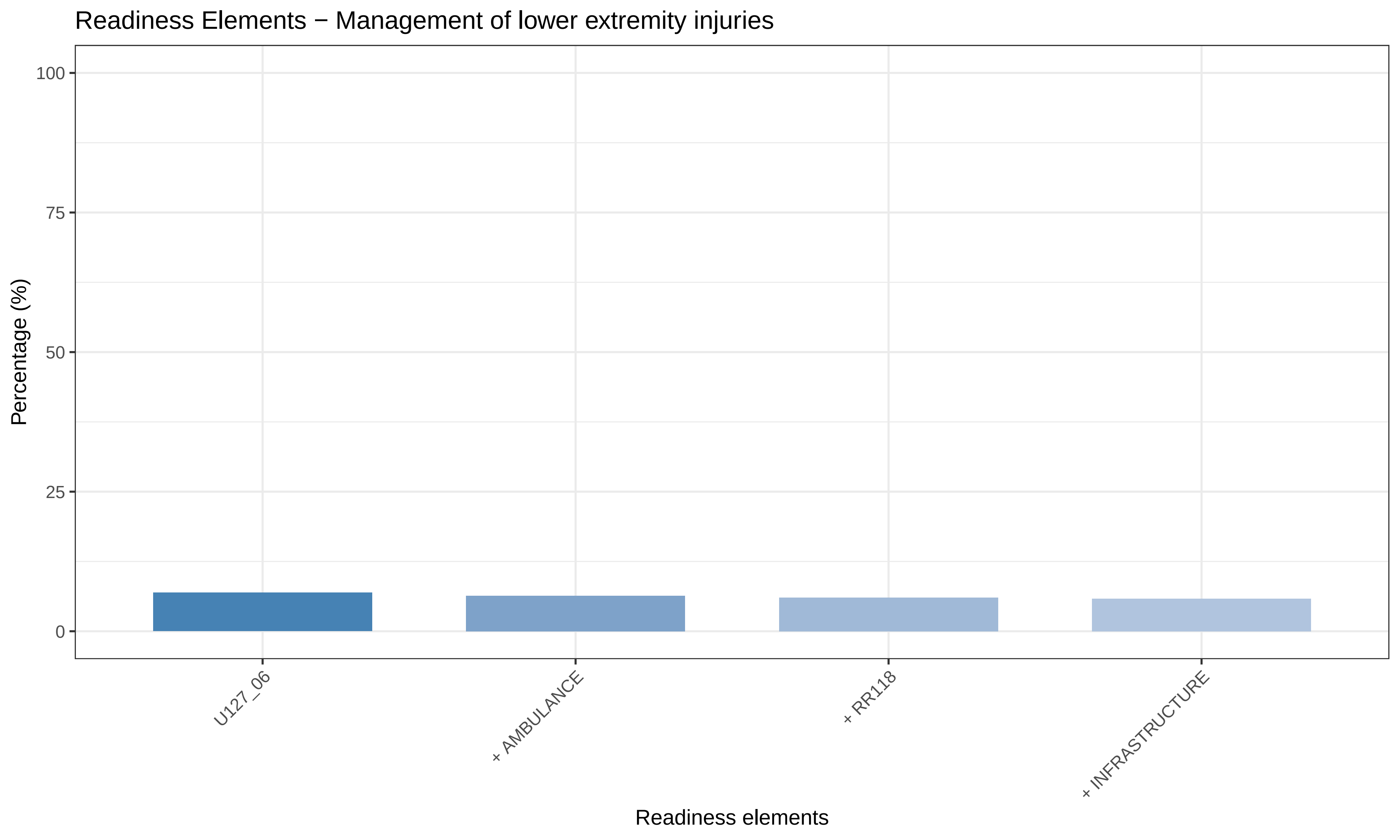
Readiness Elements − Management of lower extremity injuries
Percentage (%)
100
75
50
25
0
U127_06
+ AMBULANCE
+ RR118
+ INFRASTRUCTURE
Readiness elements

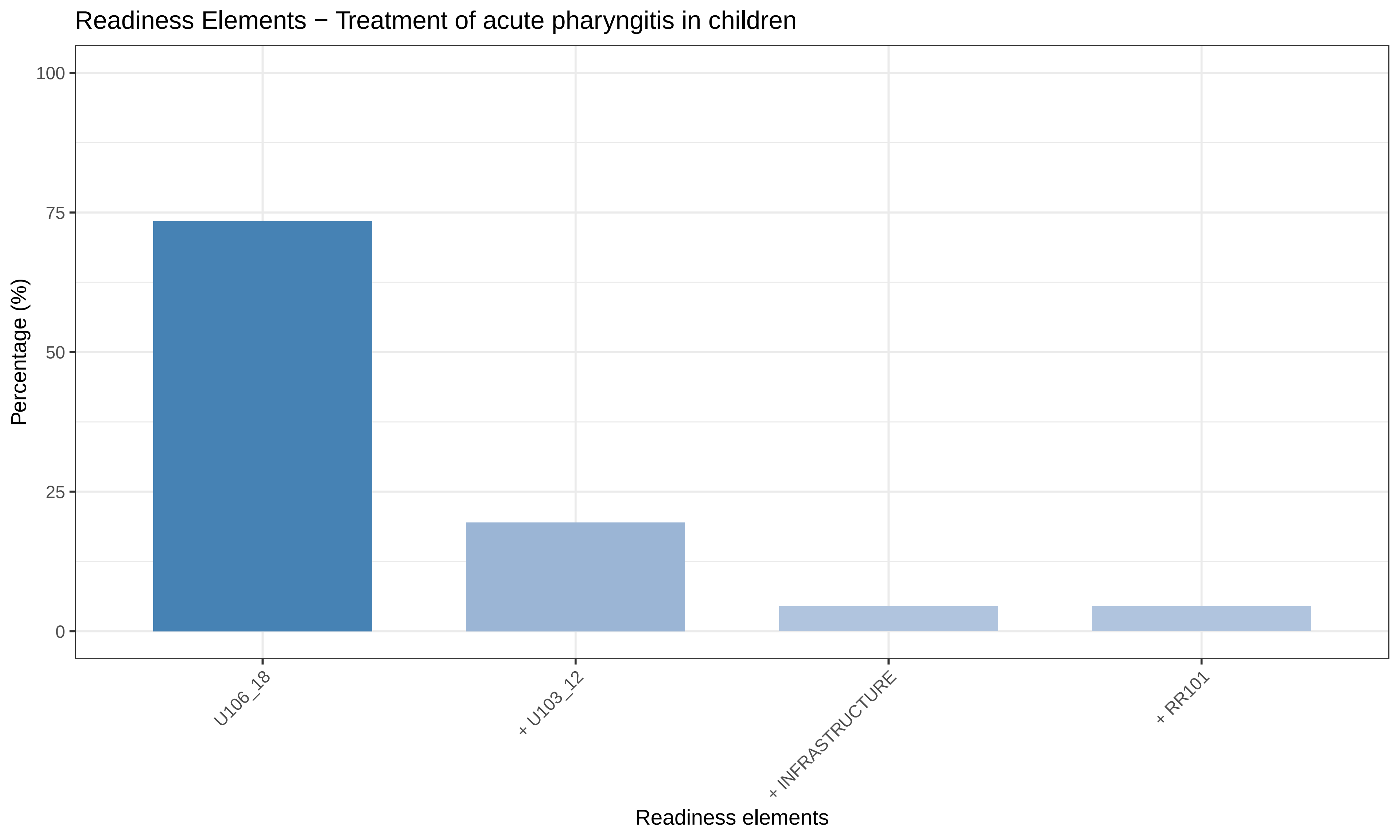
Readiness Elements − Treatment of acute pharyngitis in children
Percentage (%)
100
75
50
25
0
U106_18
+ U103_12
+ INFRASTRUCTURE
+ RR101
Readiness elements

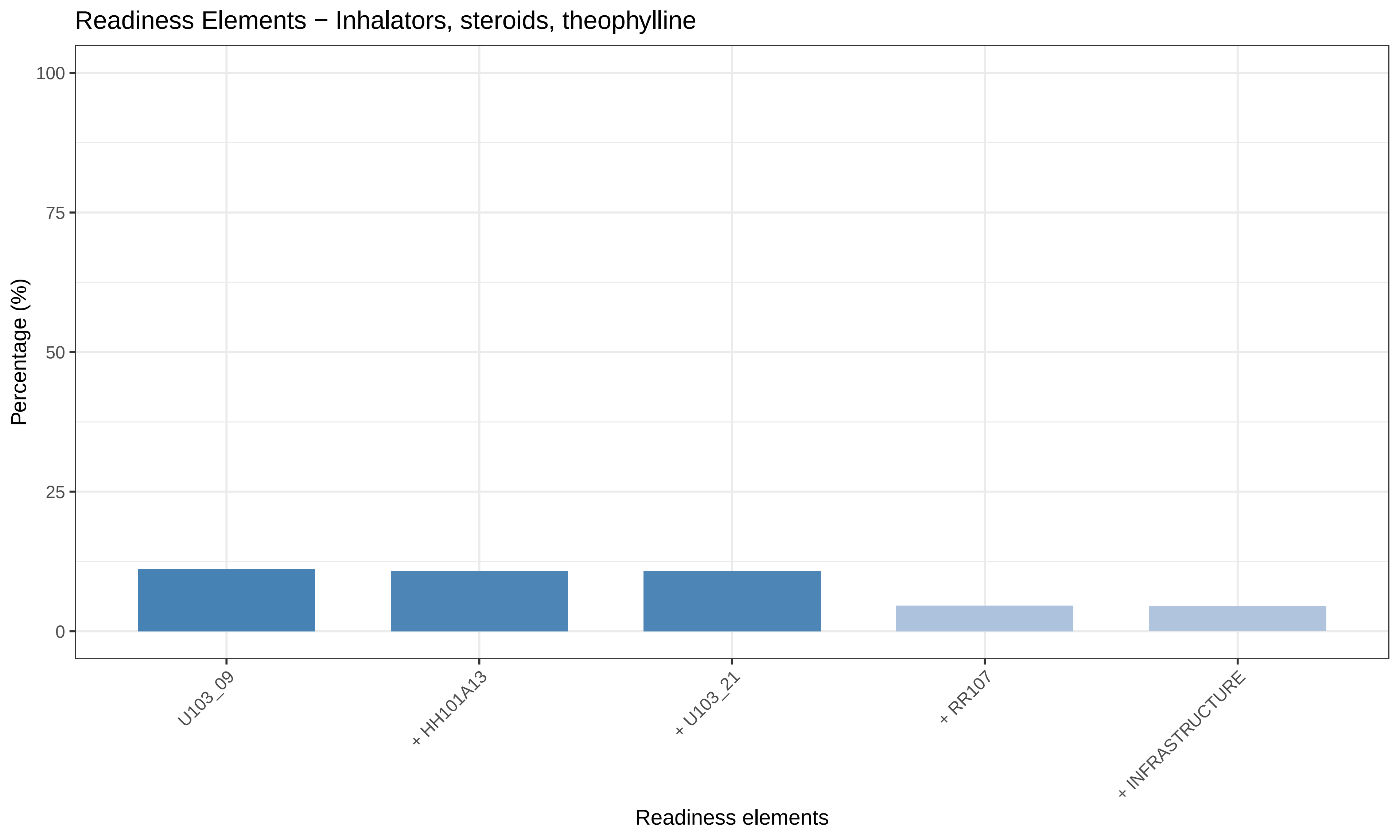
Readiness Elements − Inhalators, steroids, theophylline
Percentage (%)
100
75
50
25
0
U103_09
+ HH101A13
+ U103_21
+ RR107
+ INFRASTRUCTURE
Readiness elements

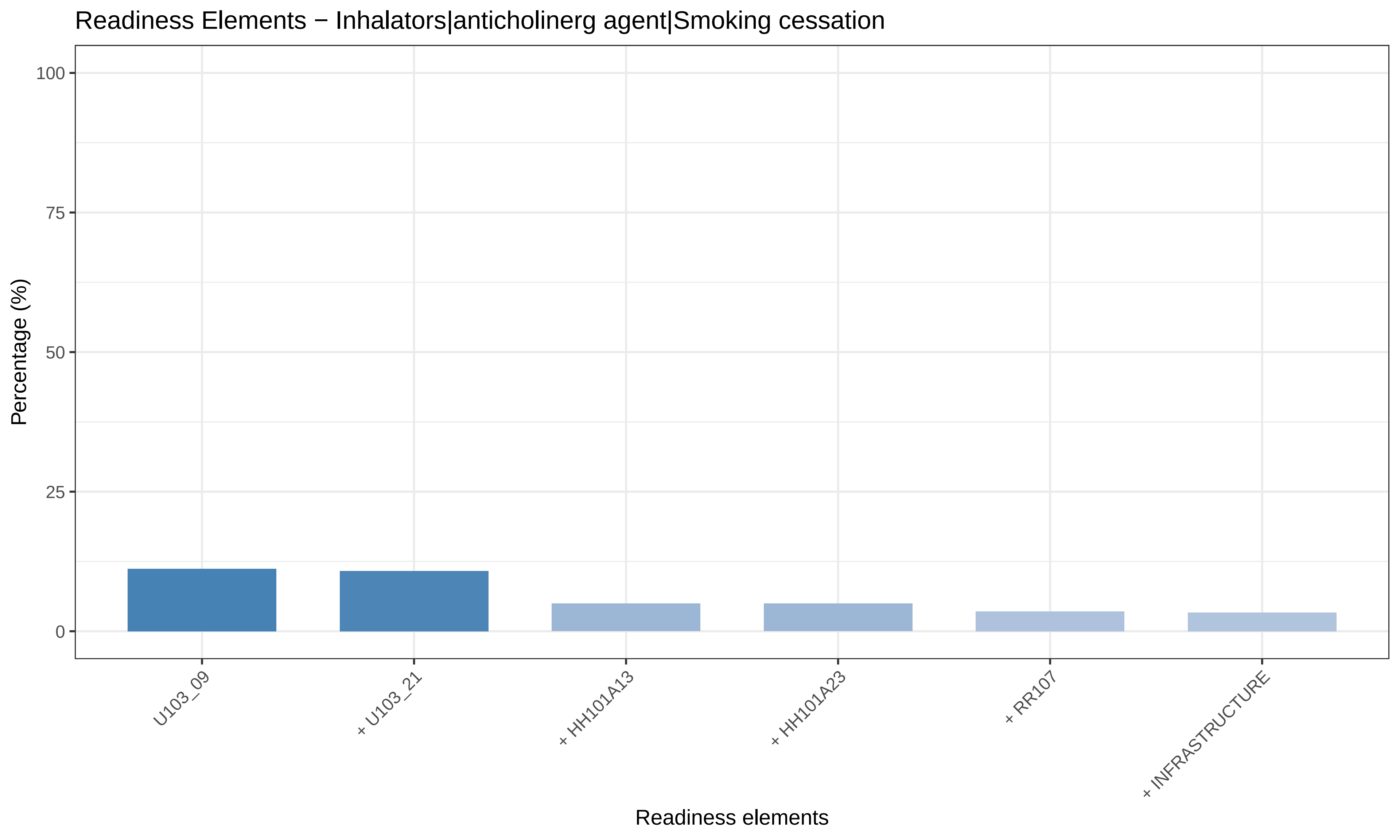
Readiness Elements − Inhalators|anticholinerg agent|Smoking cessation
Percentage (%)
100
75
50
25
0
U103_09
+ U103_21
+ HH101A13
+ HH101A23
+ RR107
+ INFRASTRUCTURE
Readiness elements

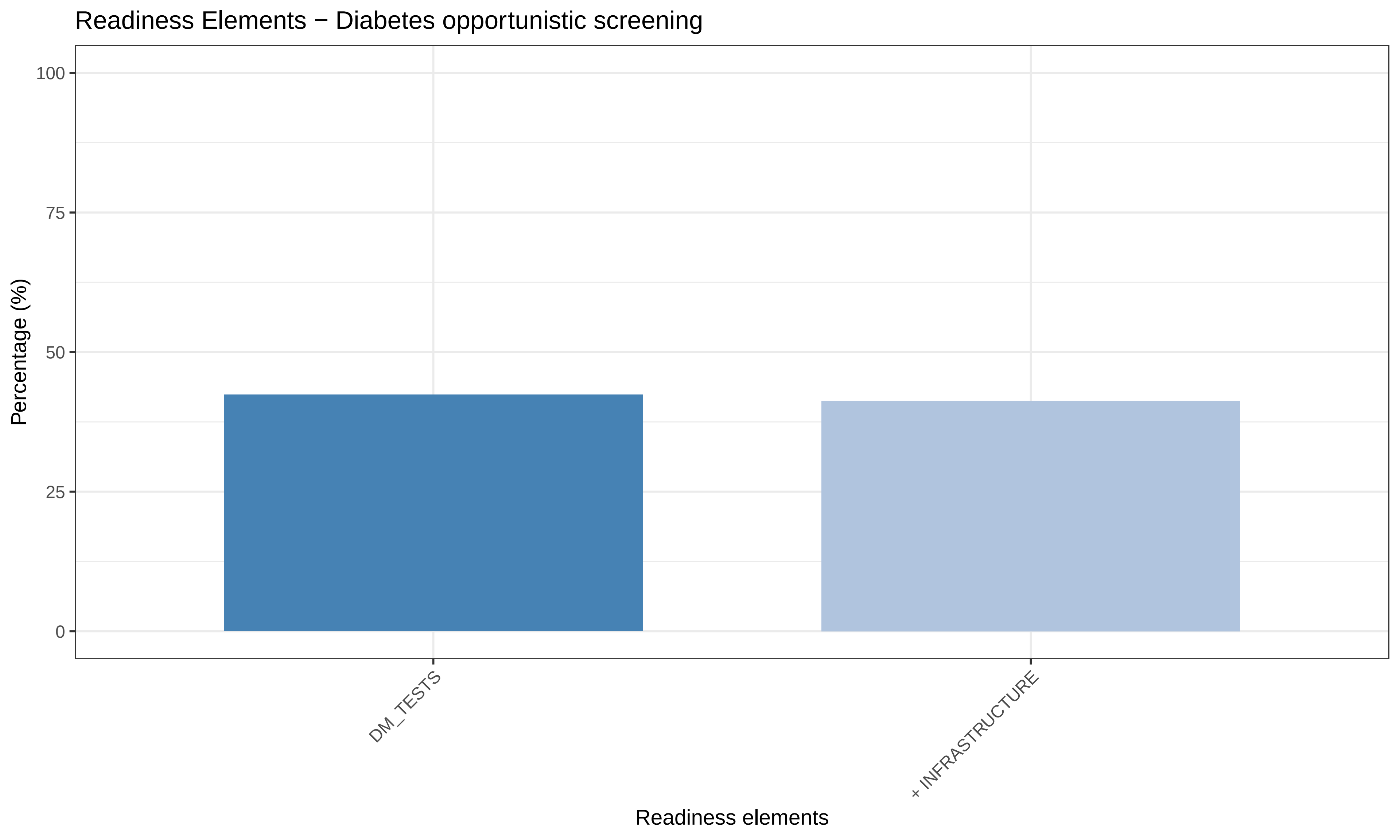

Readiness Elements − Diabetes opportunistic screening
Percentage (%)
100
75
50
25
0
DM_TESTS
+ INFRASTRUCTURE
Readiness elements

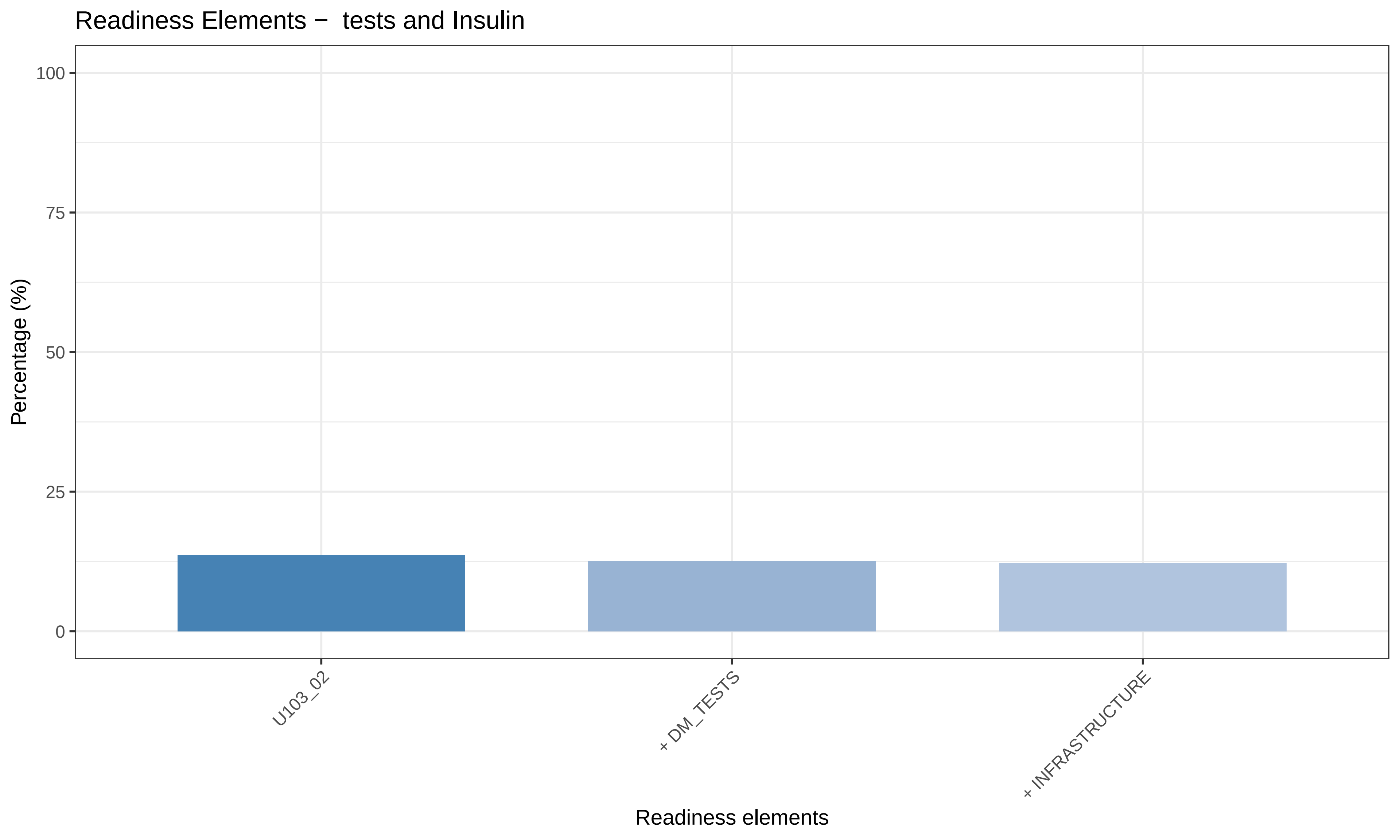
Readiness Elements − tests and Insulin
Percentage (%)
100
75
50
25
0
U103_02
+ DM_TESTS
+ INFRASTRUCTURE
Readiness elements

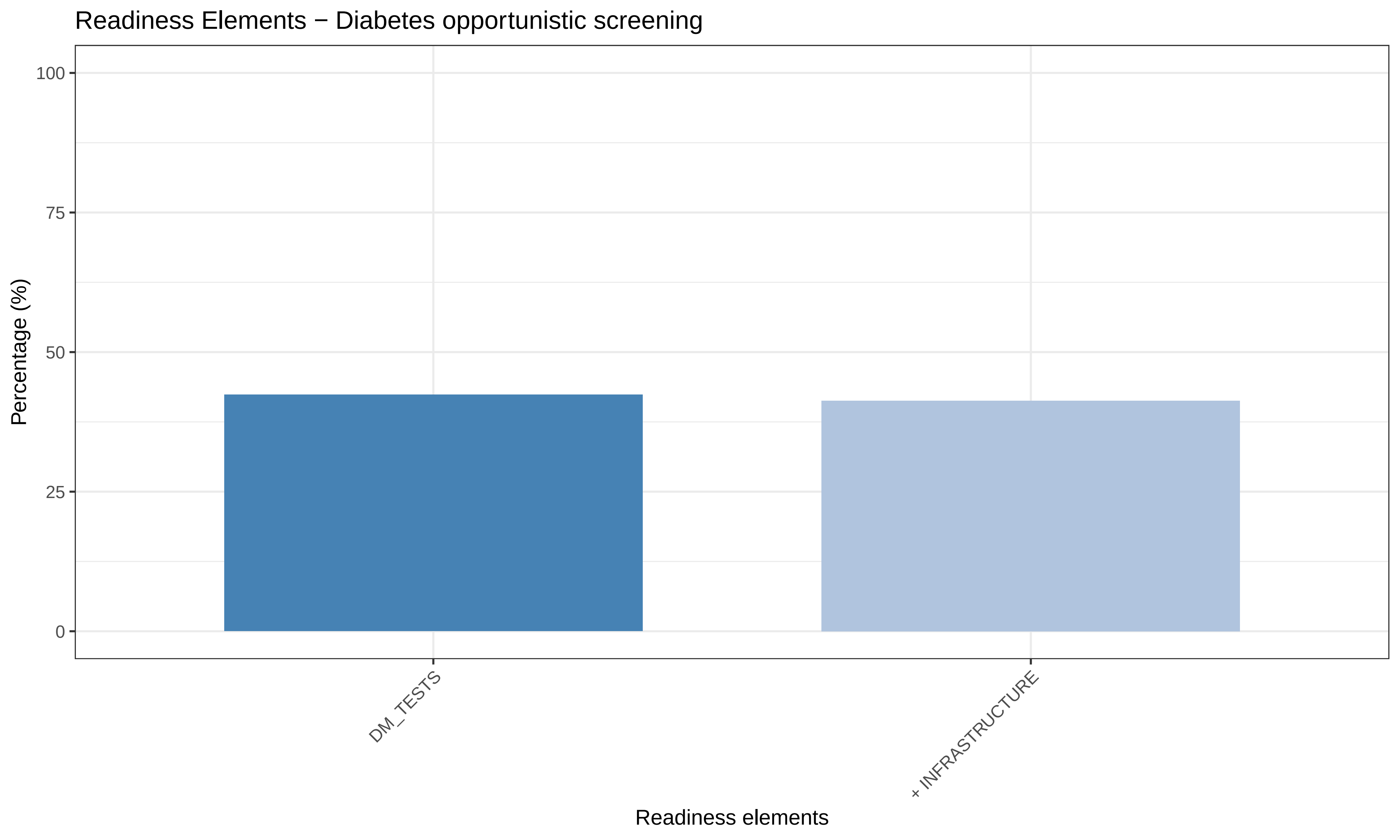
Readiness Elements − Diabetes opportunistic screening
Percentage (%)
100
75
50
25
0
DM_TESTS
+ INFRASTRUCTURE
Readiness elements

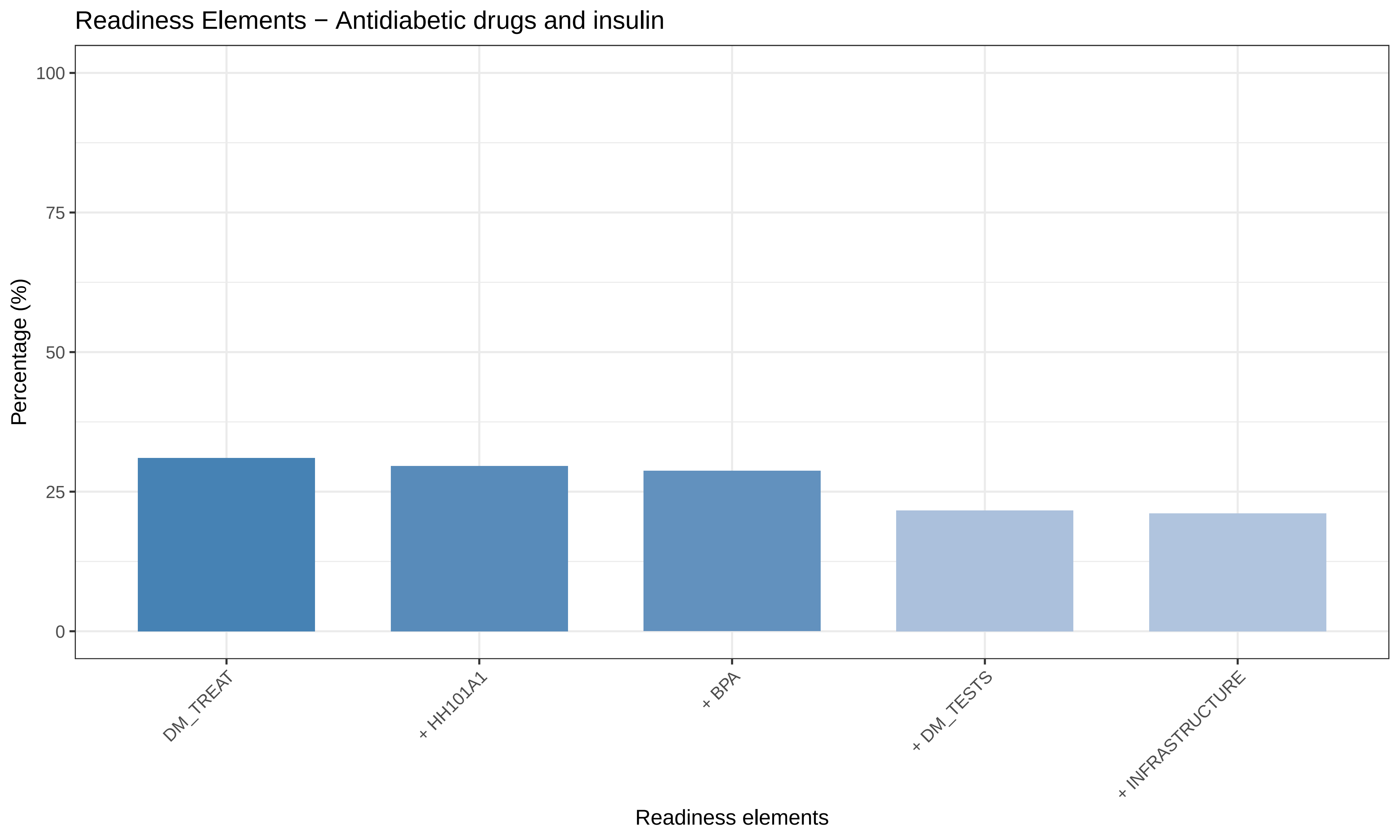
Readiness Elements − Antidiabetic drugs and insulin
Percentage (%)
100
75
50
25
0
DM_TREAT
+ HH101A1
+ BPA
+ DM_TESTS
+ INFRASTRUCTURE
Readiness elements

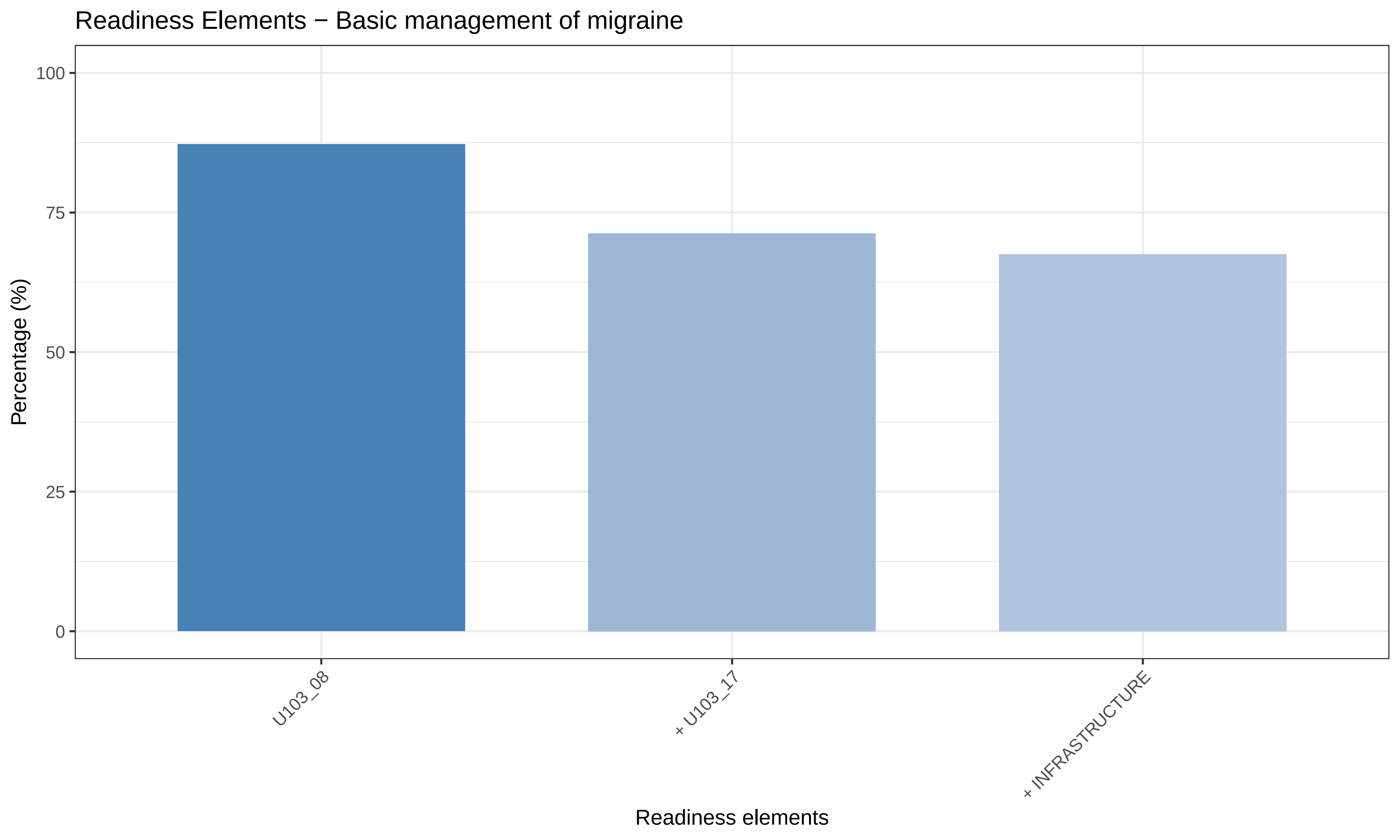
Readiness Elements − Basic management of migraine
Percentage (%)
100
75
50
25
0
U103_08
+ U103_17
+ INFRASTRUCTURE
Readiness elements

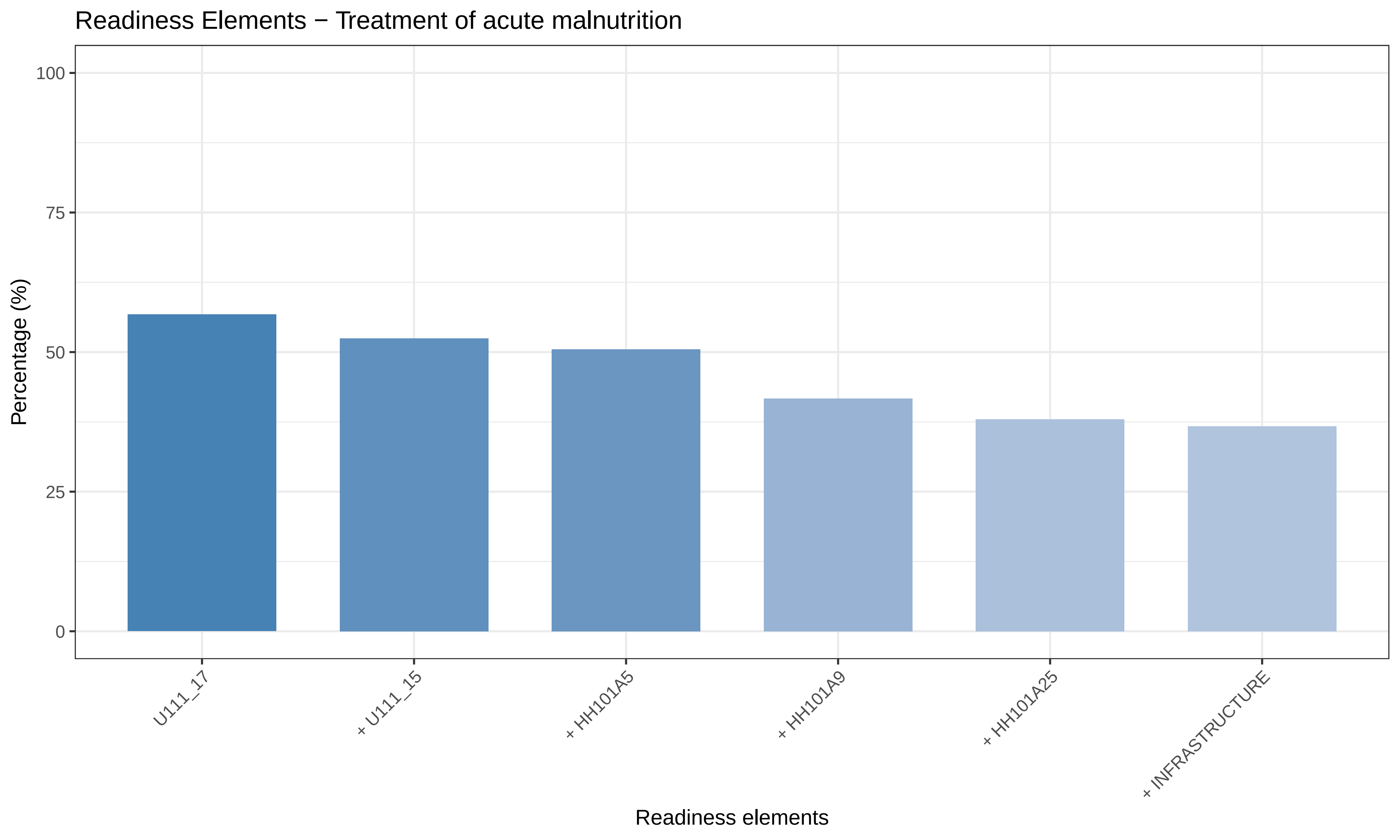
Readiness Elements − Treatment of acute malnutrition
100
75
50
25
0
Percentage (%)
U111_17
+ U111_15
+ HH101A5
+ HH101A9
+ HH101A25
+ INFRASTRUCTURE
Readiness elements

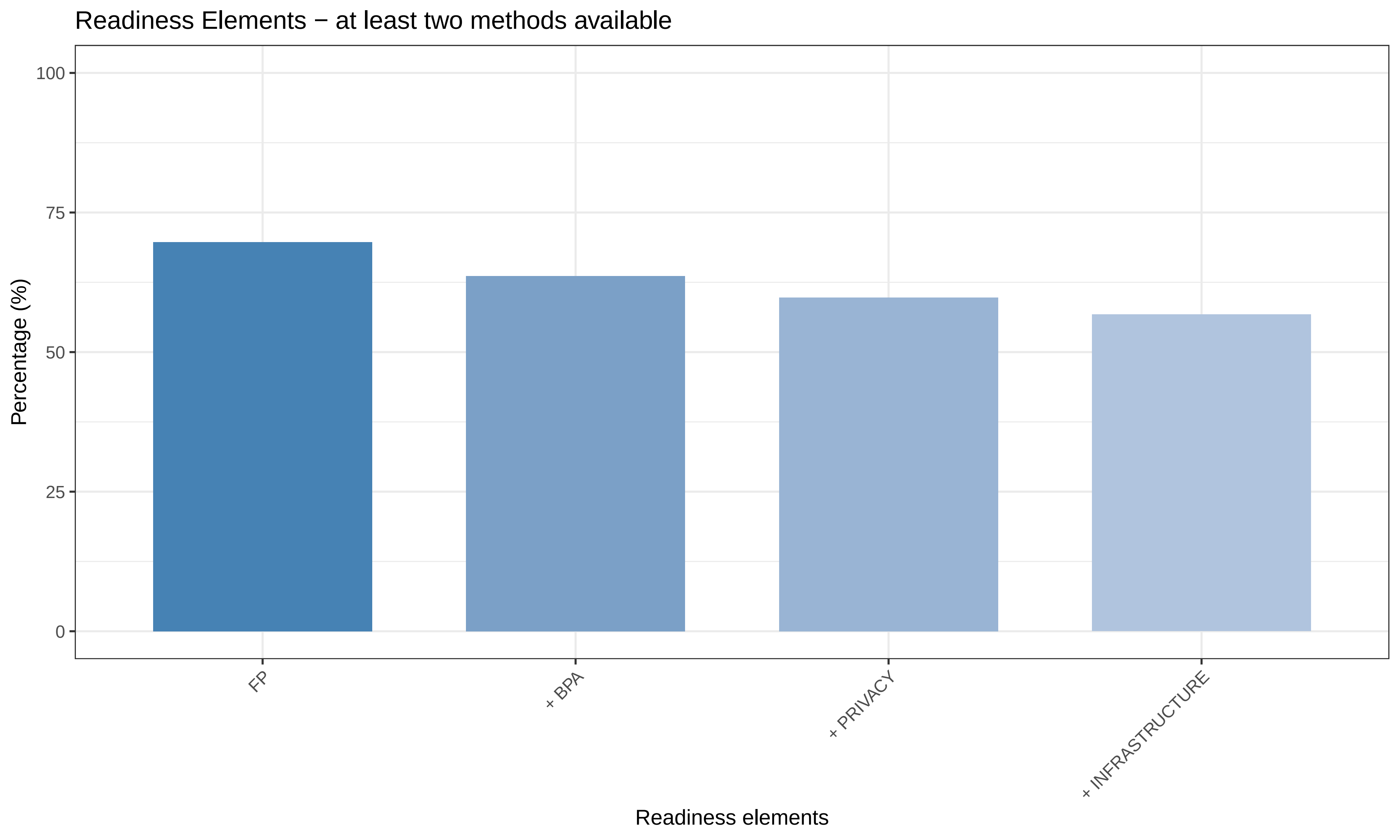

Readiness Elements − at least two methods available
Percentage (%)
100
75
50
25
0
FP
+ BPA
+ PRIVACY
+ INFRASTRUCTURE
Readiness elements

Readiness Elements − Uneventful pregnancy

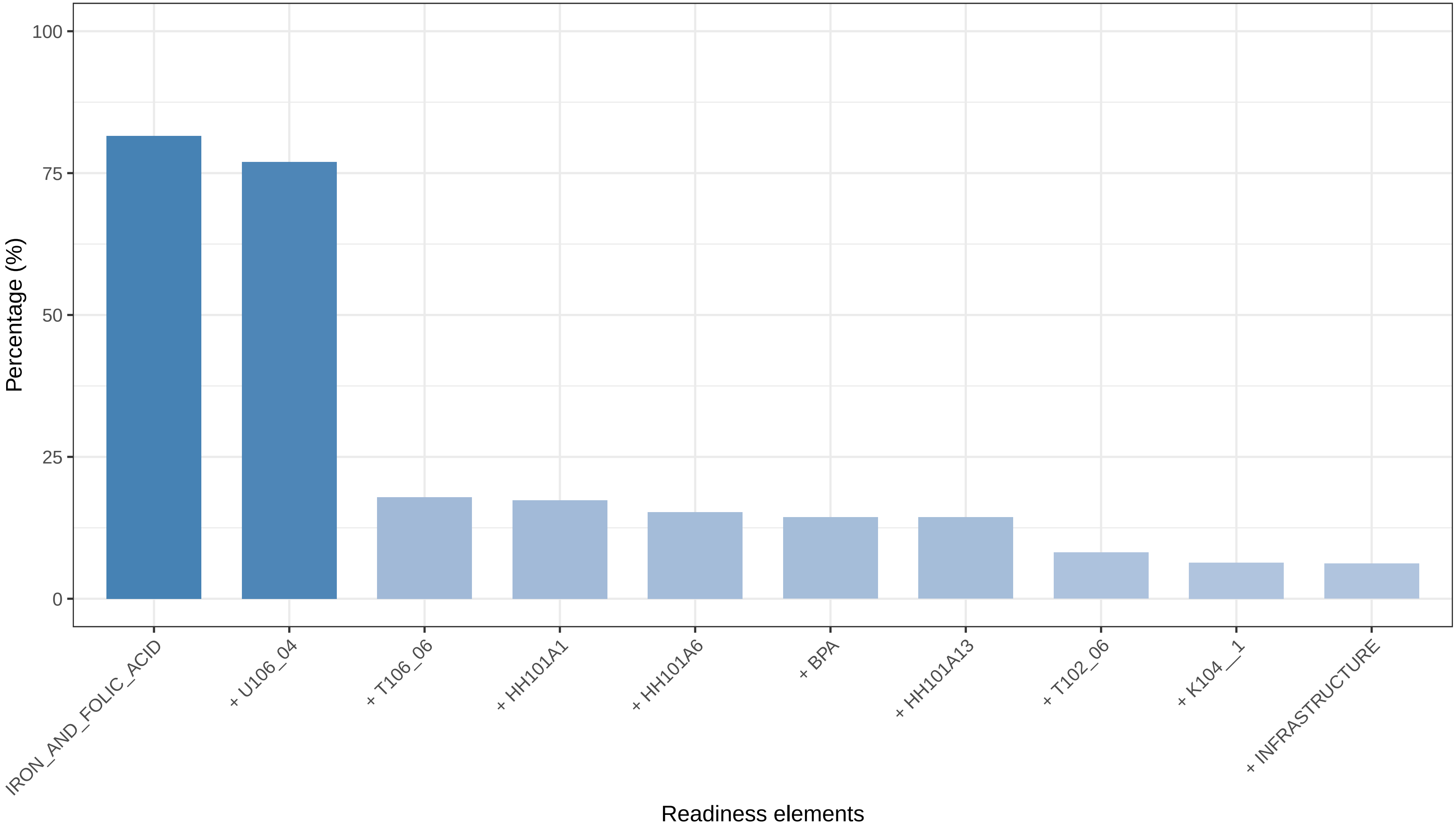

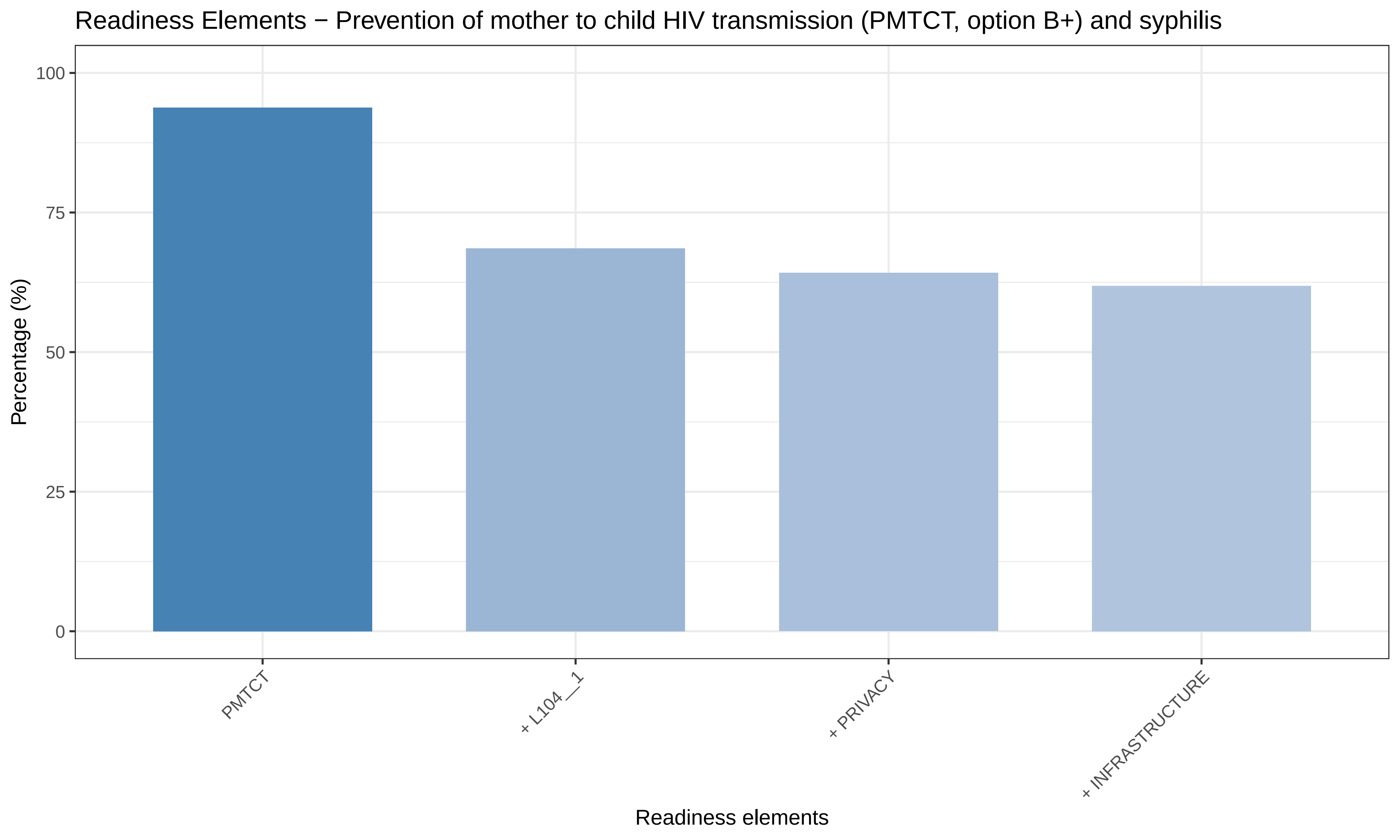
Readiness Elements − Prevention of mother to child HIV transmission (PMTCT, option B+) and syphilis
Percentage (%)
100
75
50
25
0
PMTCT
+ L104__1
+ PRIVACY
+ INFRASTRUCTURE
Readiness elements

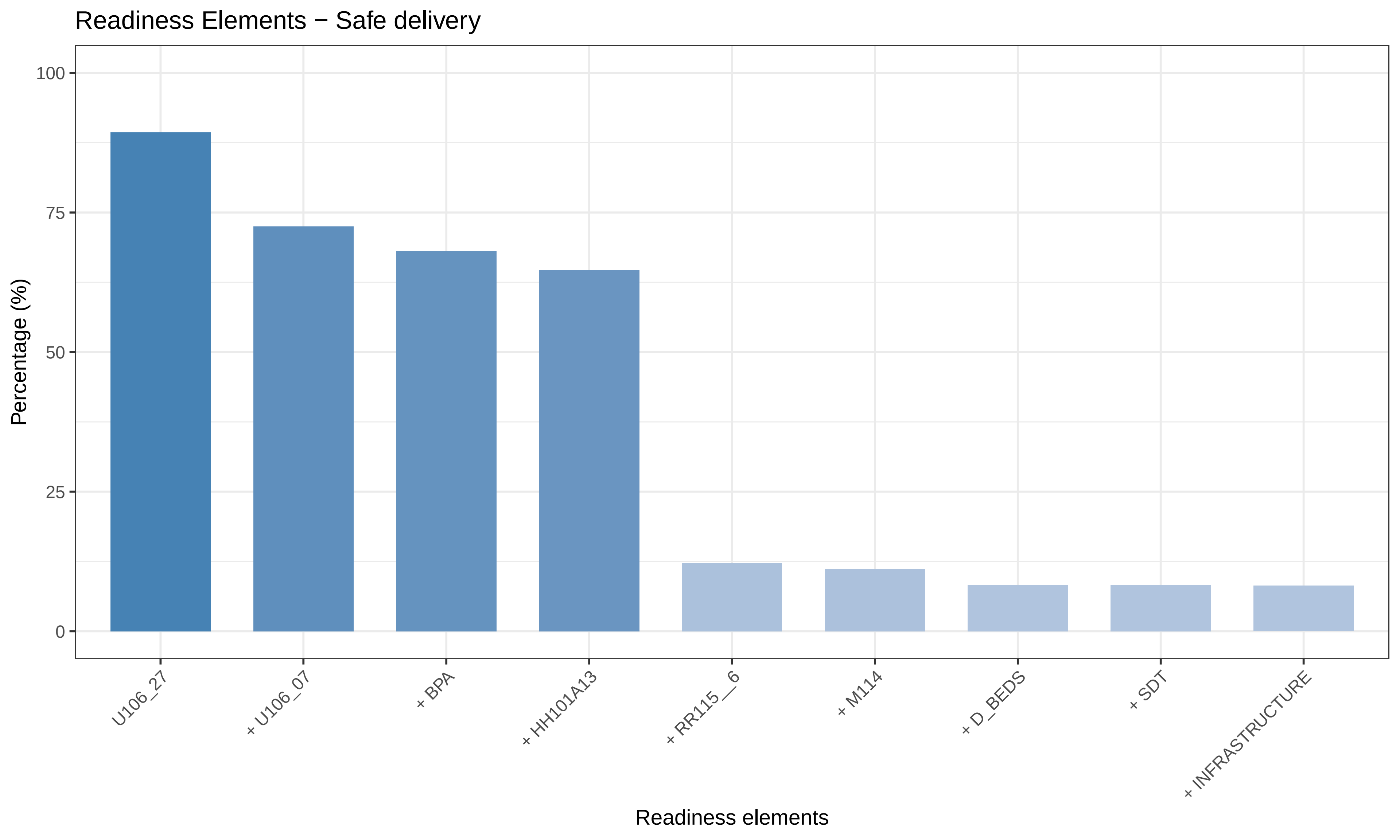
Readiness Elements − Safe delivery
Percentage (%)
100
75
50
25
0
U106_27
+ U106_07
+ BPA
+ HH101A13
+ RR115__6
+ M114
+ D_BEDS
+ SDT
+ INFRASTRUCTURE
Readiness elements

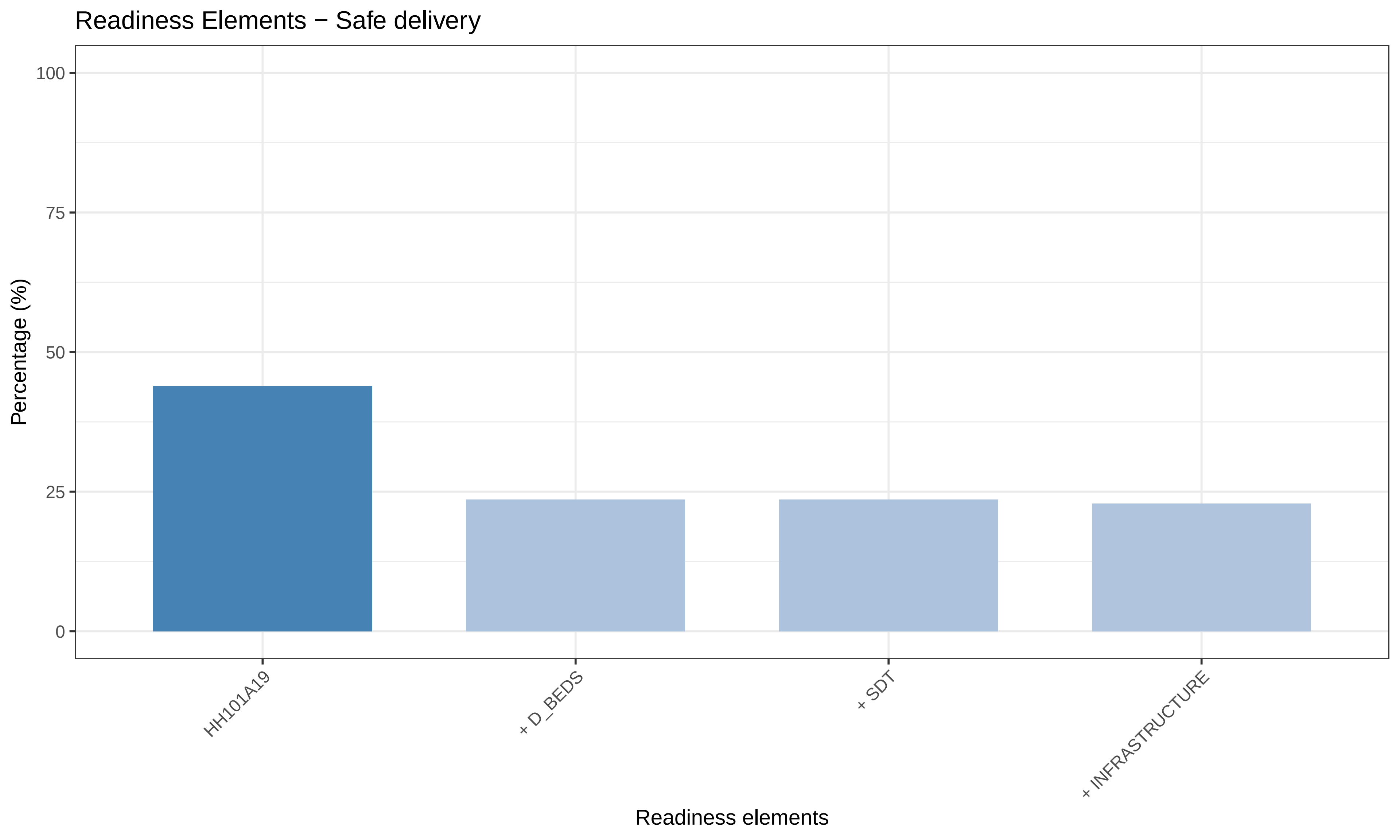
Readiness Elements − Safe delivery
Percentage (%)
100
75
50
25
0
HH101A19
+ D_BEDS
+ SDT
+ INFRASTRUCTURE
Readiness elements

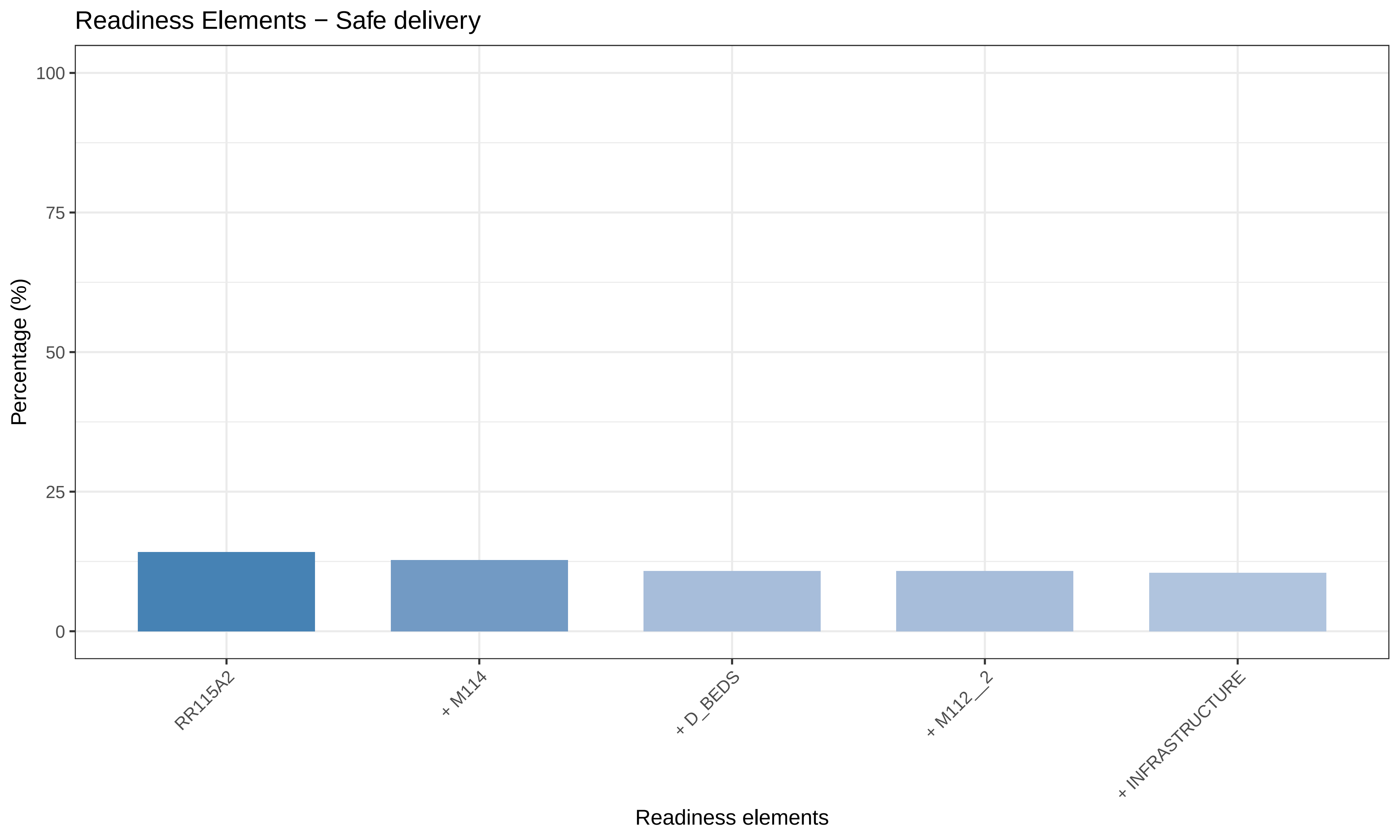
Readiness Elements − Safe delivery
Percentage (%)
100
75
50
25
0
RR115A2
+ M114
+ D_BEDS
+ M112__2
+ INFRASTRUCTURE
Readiness elements

Readiness Elements − Management of maternal sepsis

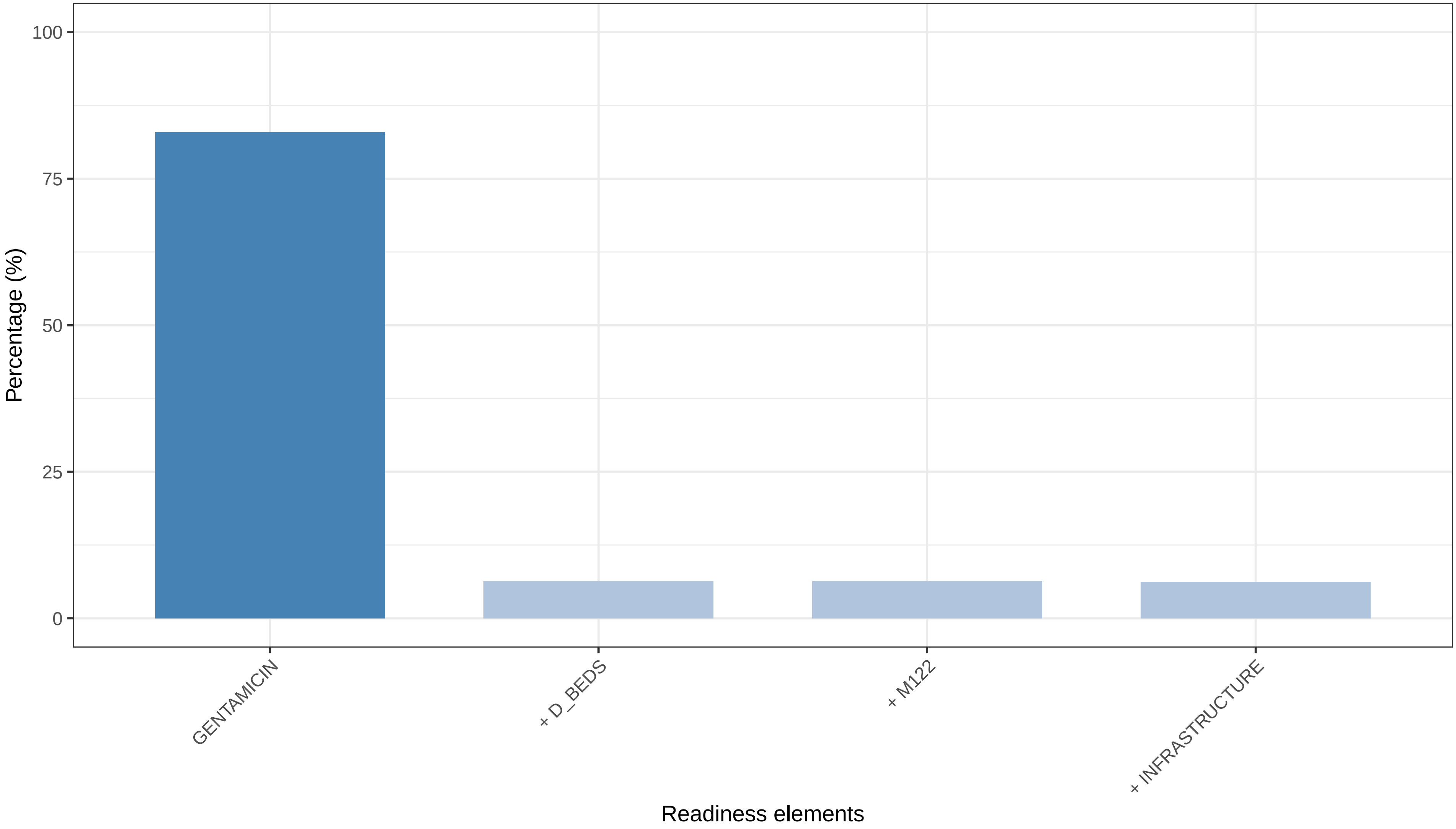

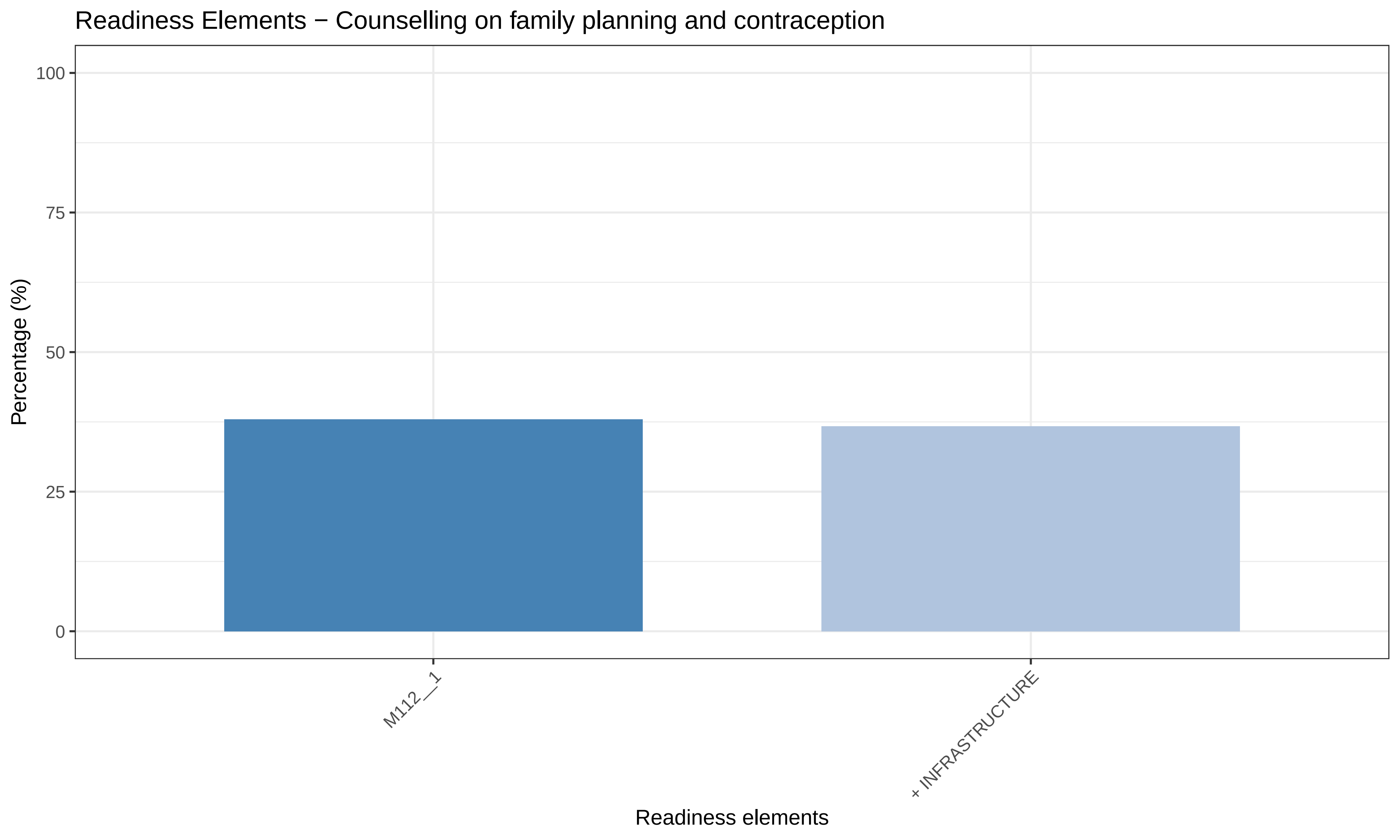

Readiness Elements − Counselling on family planning and contraception
Percentage (%)
100
75
50
25
0
M112__1
+ INFRASTRUCTURE
Readiness elements

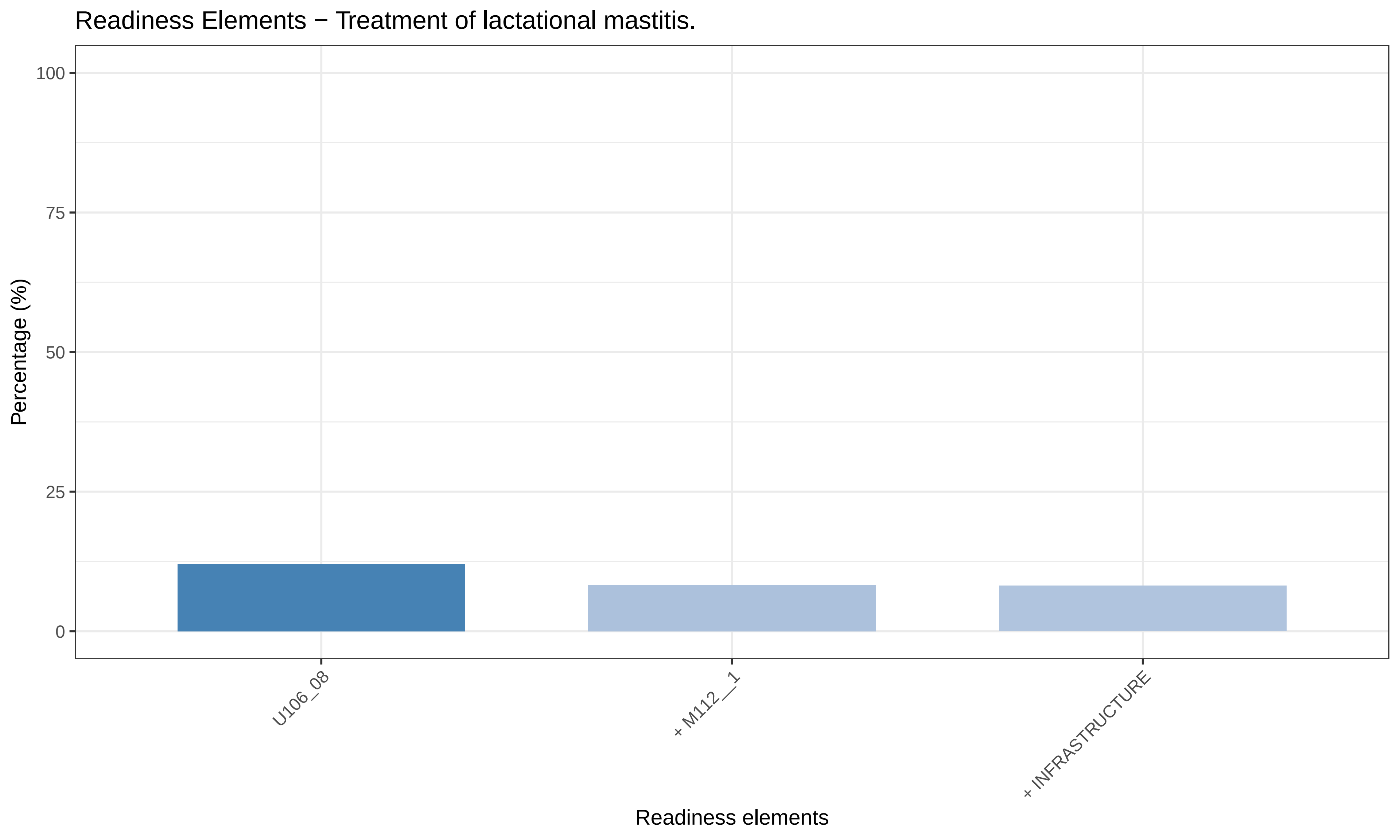
Readiness Elements − Treatment of lactational mastitis.
Percentage (%)
100
75
50
25
0
U106_08
+ M112__1
+ INFRASTRUCTURE
Readiness elements

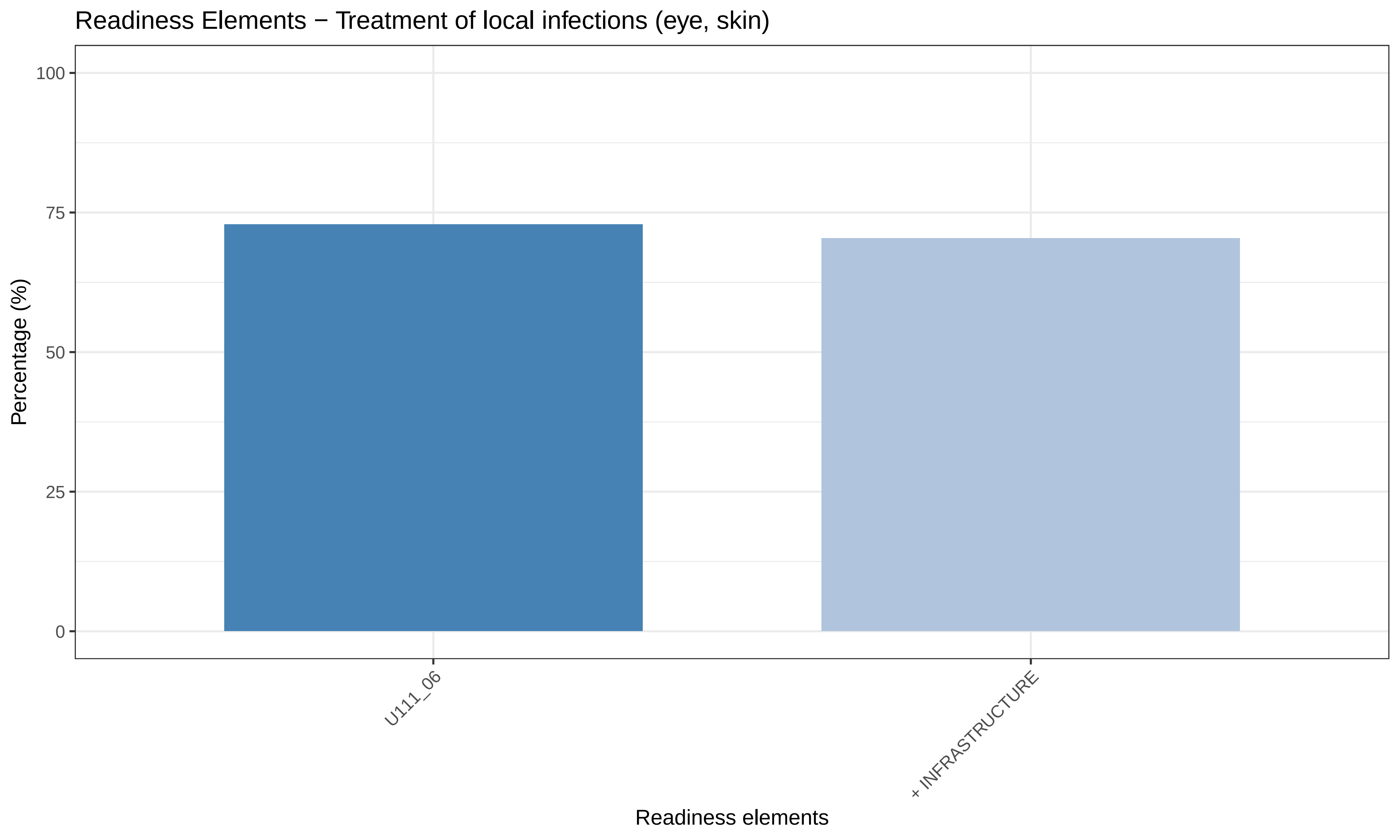
Readiness Elements − Treatment of local infections (eye, skin)
Percentage (%)
100
75
50
25
0
U111_06
+ INFRASTRUCTURE
Readiness elements

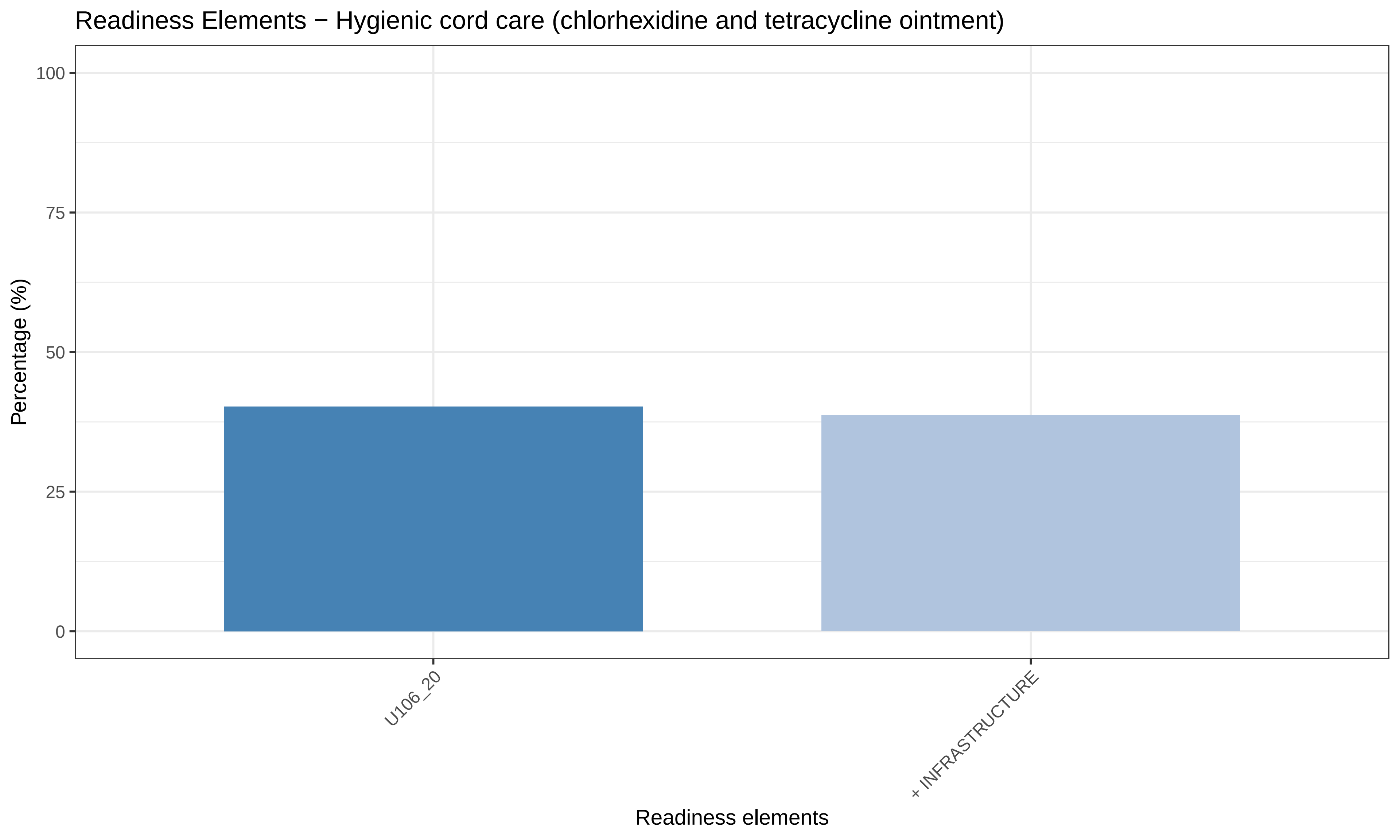
Readiness Elements − Hygienic cord care (chlorhexidine and tetracycline ointment)
100
75
50
25
0
Percentage (%)
U106_20
+ INFRASTRUCTURE
Readiness elements

Readiness Cascades_RCH+

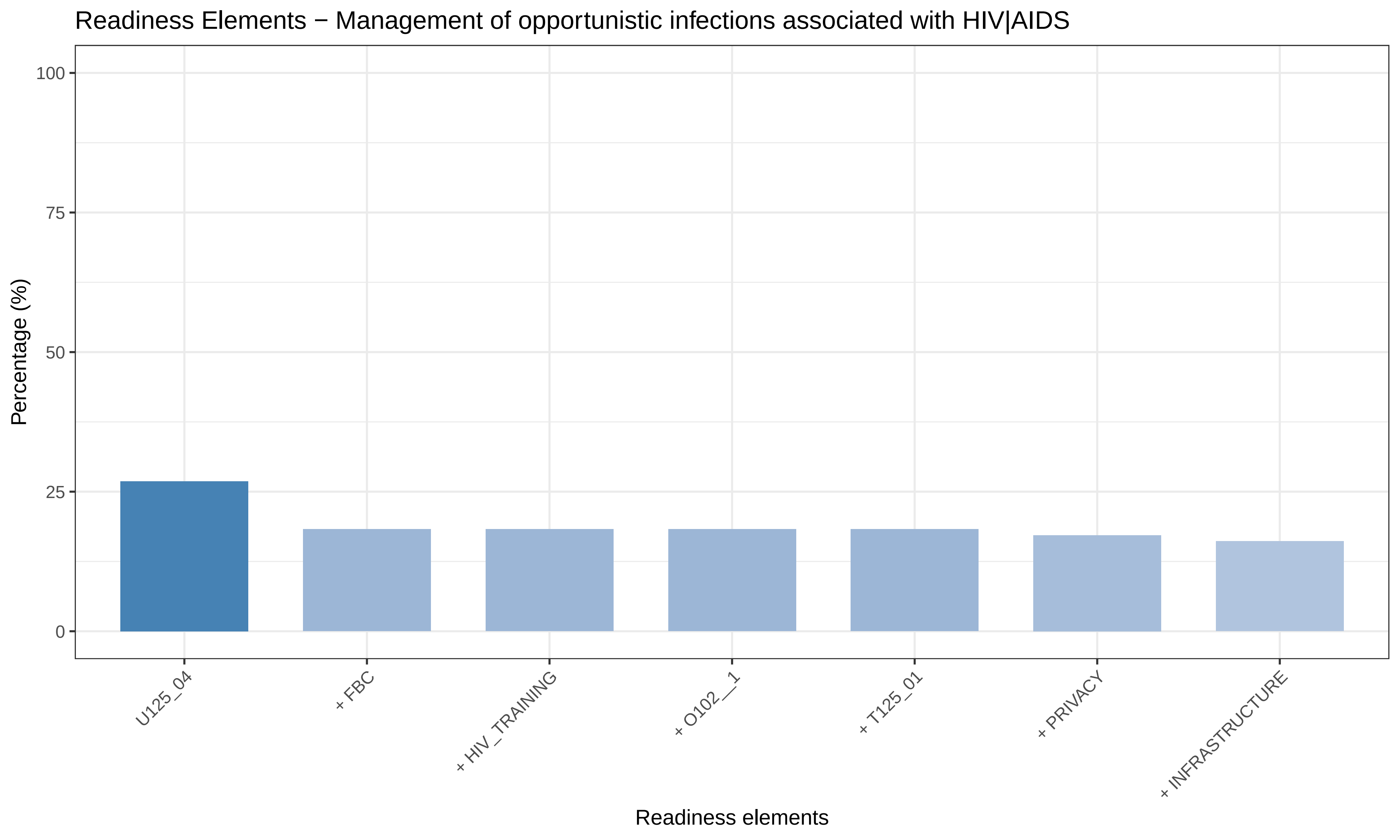

Readiness Elements − Management of opportunistic infections associated with HIV|AIDS
Percentage (%)
100
75
50
25
0
U125_04
+ FBC
+ HIV_TRAINING
+ O102__1
+ T125_01
+ PRIVACY
+ INFRASTRUCTURE
Readiness elements

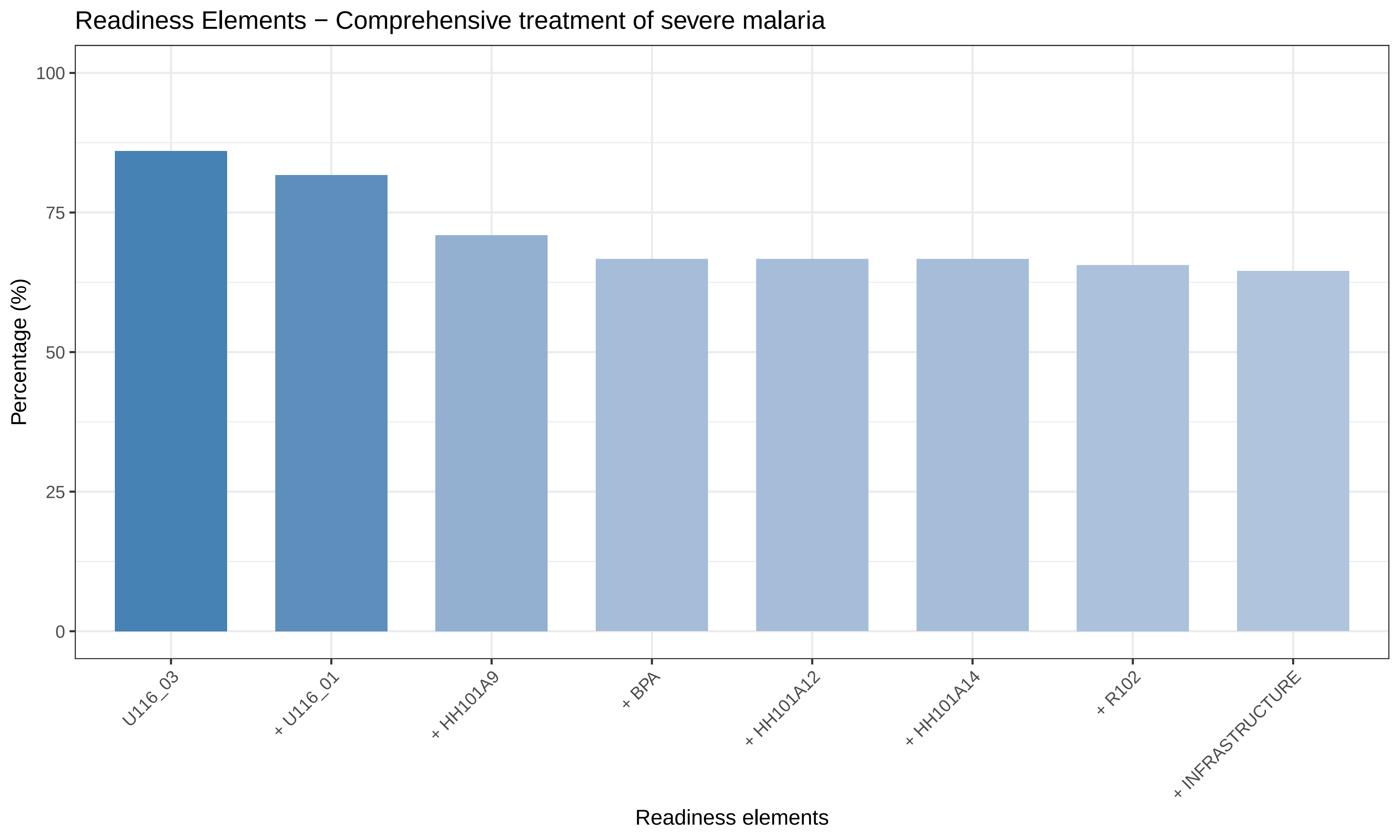
Readiness Elements − Comprehensive treatment of severe malaria
Percentage (%)
100
75
50
25
0
U116_03
+ U116_01
+ HH101A9
+ BPA
+ HH101A12
+ HH101A14
+ R102
+ INFRASTRUCTURE
Readiness elements

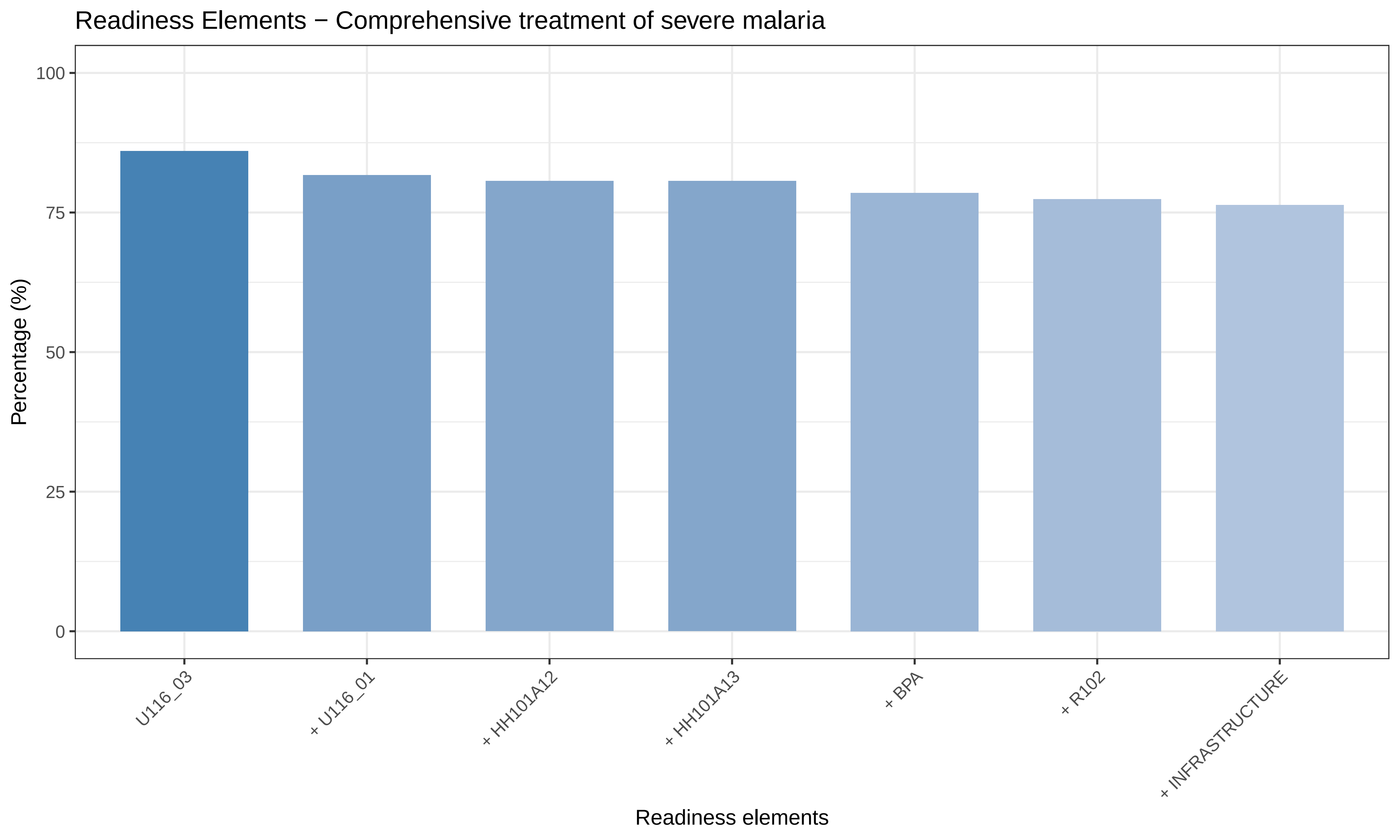

Readiness Elements − Comprehensive treatment of severe malaria
Percentage (%)
100
75
50
25
0
U116_03
+ U116_01
+ HH101A12
+ HH101A13
+ BPA
+ R102
+ INFRASTRUCTURE
Readiness elements

Readiness Elements − Treatment of PID (Pelvic Inflammatory Disease)

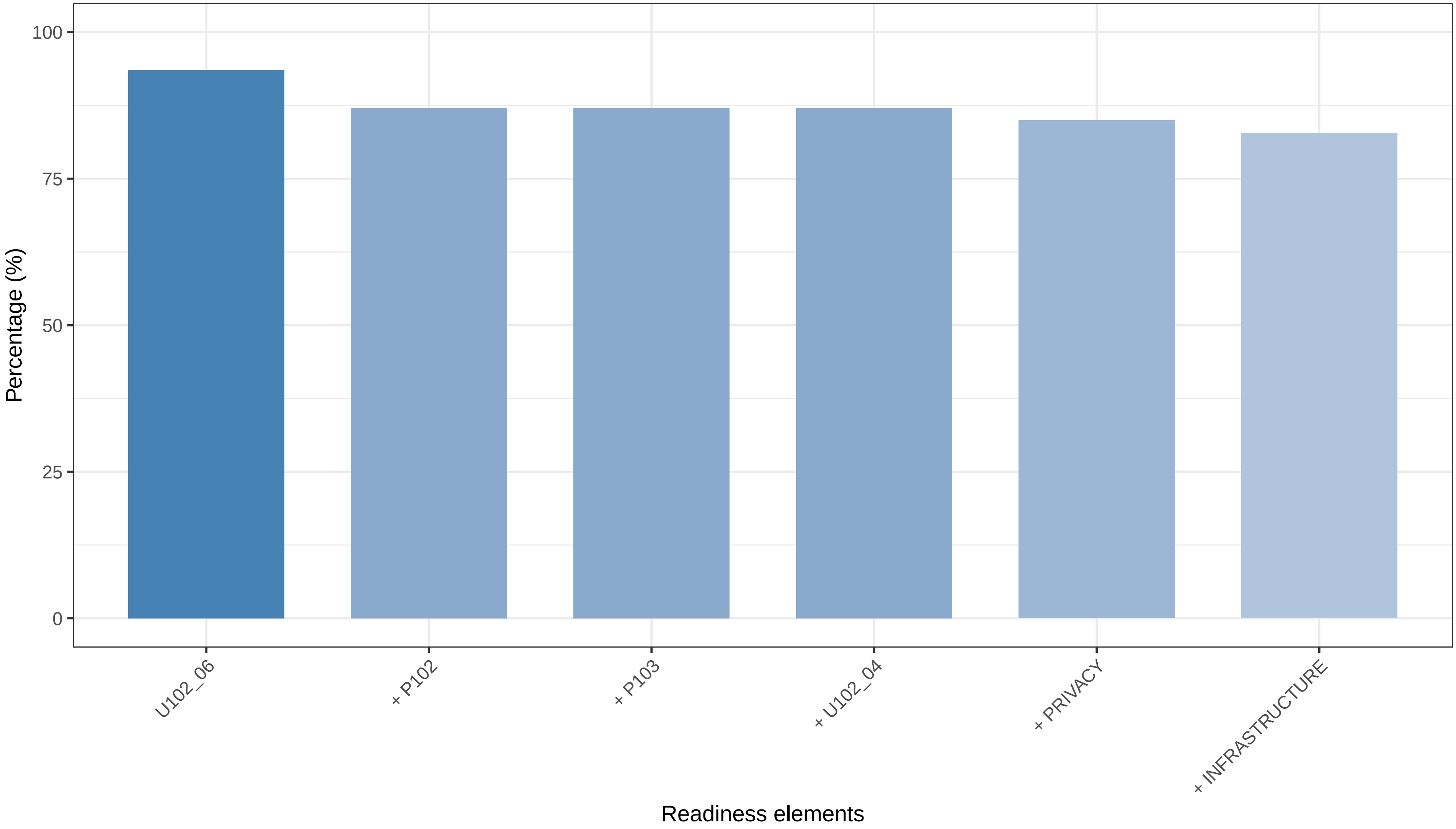

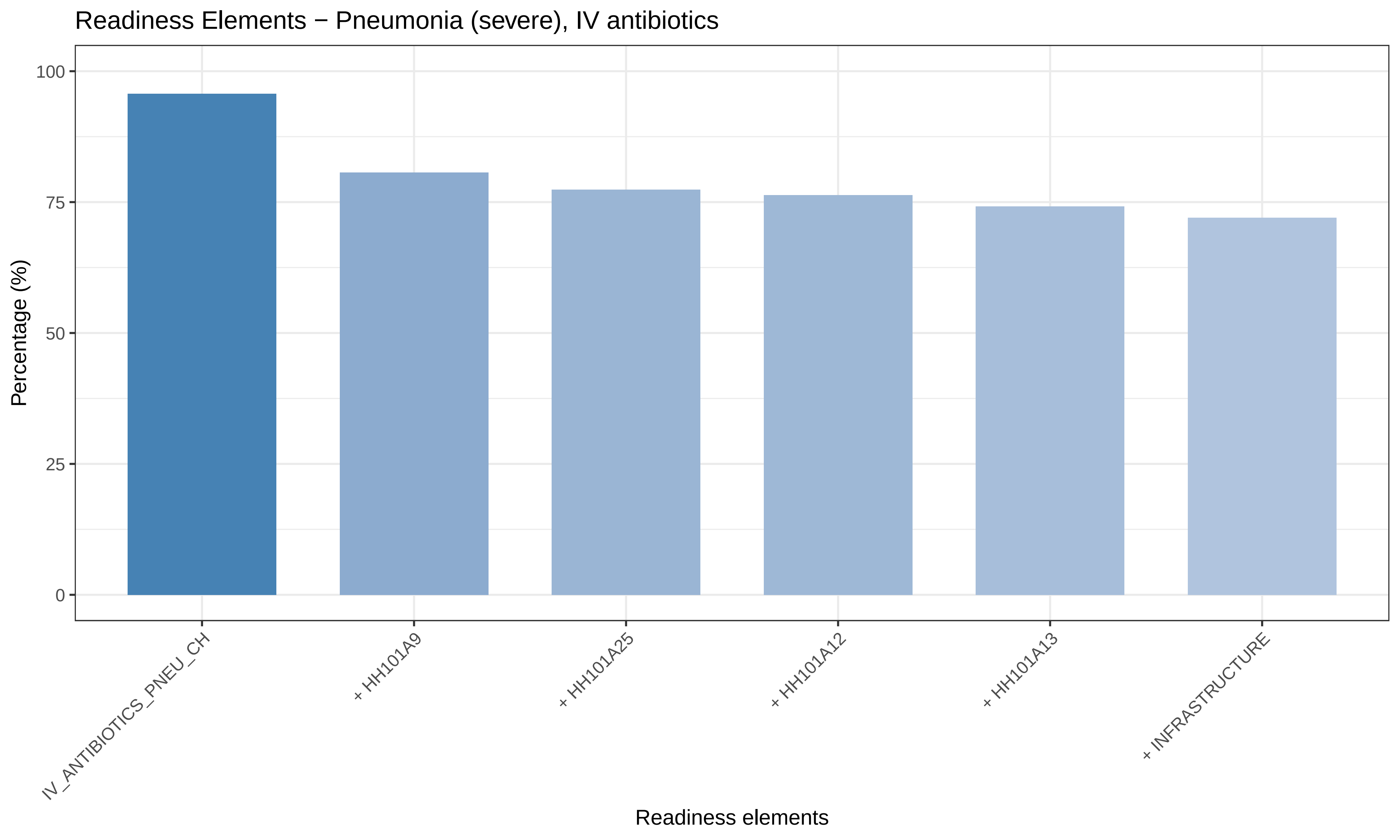
Readiness Elements − Pneumonia (severe), IV antibiotics
Percentage (%)
100
75
50
25
0
IV_ANTIBIOTICS_PNEU_CH
+ HH101A9
+ HH101A25
+ HH101A12
+ HH101A13
+ INFRASTRUCTURE
Readiness elements

Readiness Elements − Pneumonia (severe), IV antibiotics adults

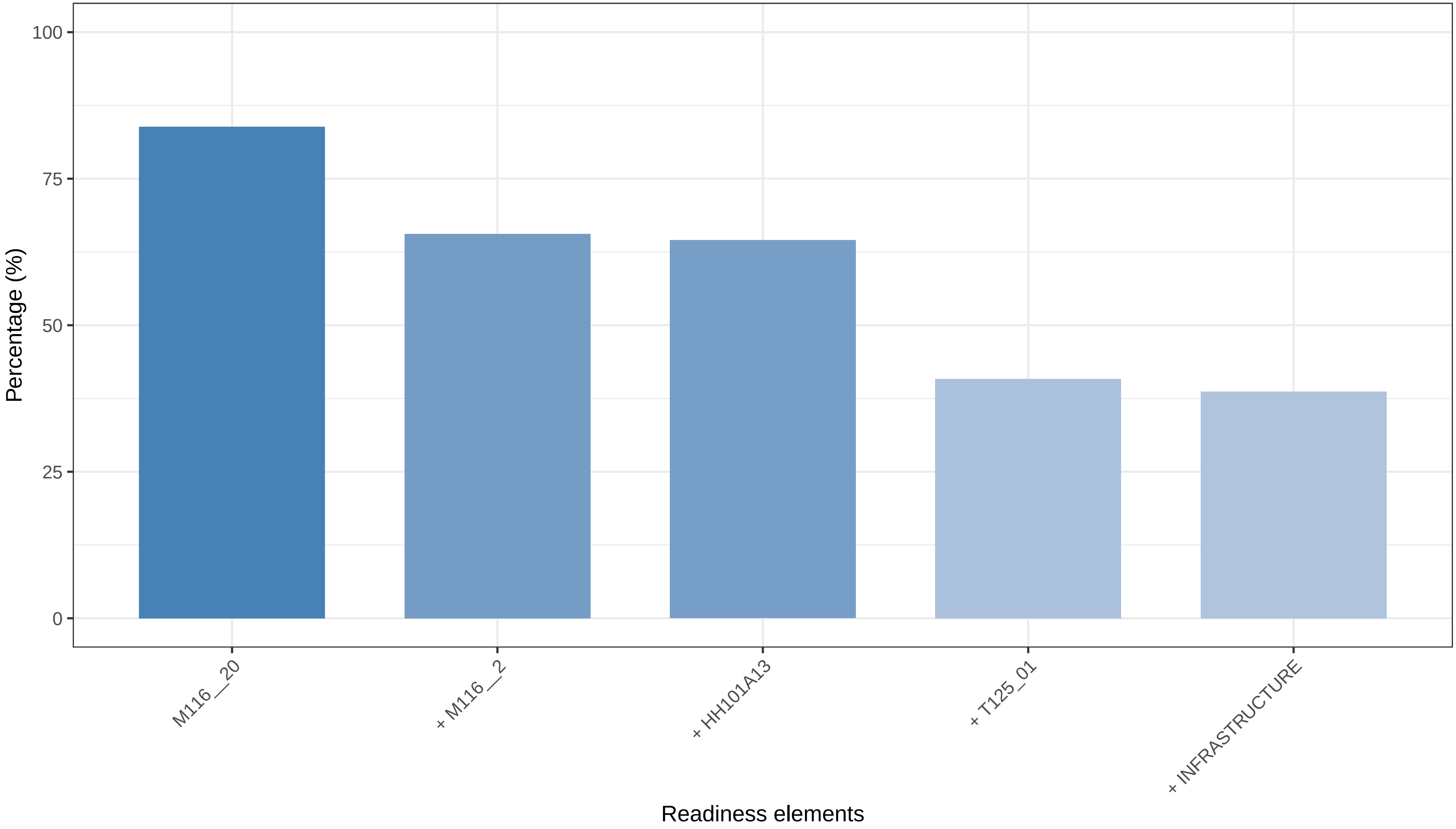

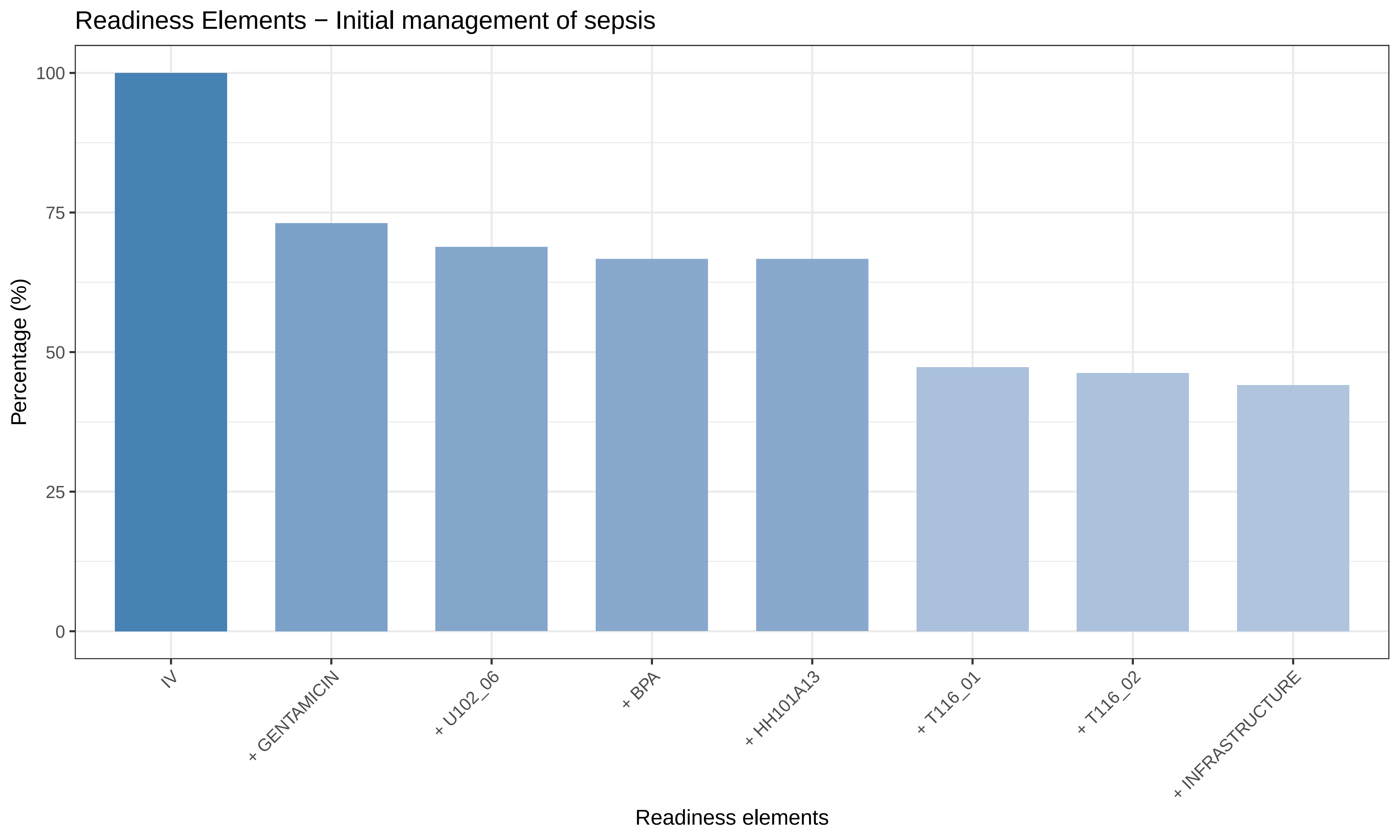

Readiness Elements − Initial management of sepsis
Percentage (%)
100
75
50
25
0
IV
+ GENTAMICIN
+ U102_06
+ BPA
+ HH101A13
+ T116_01
+ T116_02
+ INFRASTRUCTURE
Readiness elements

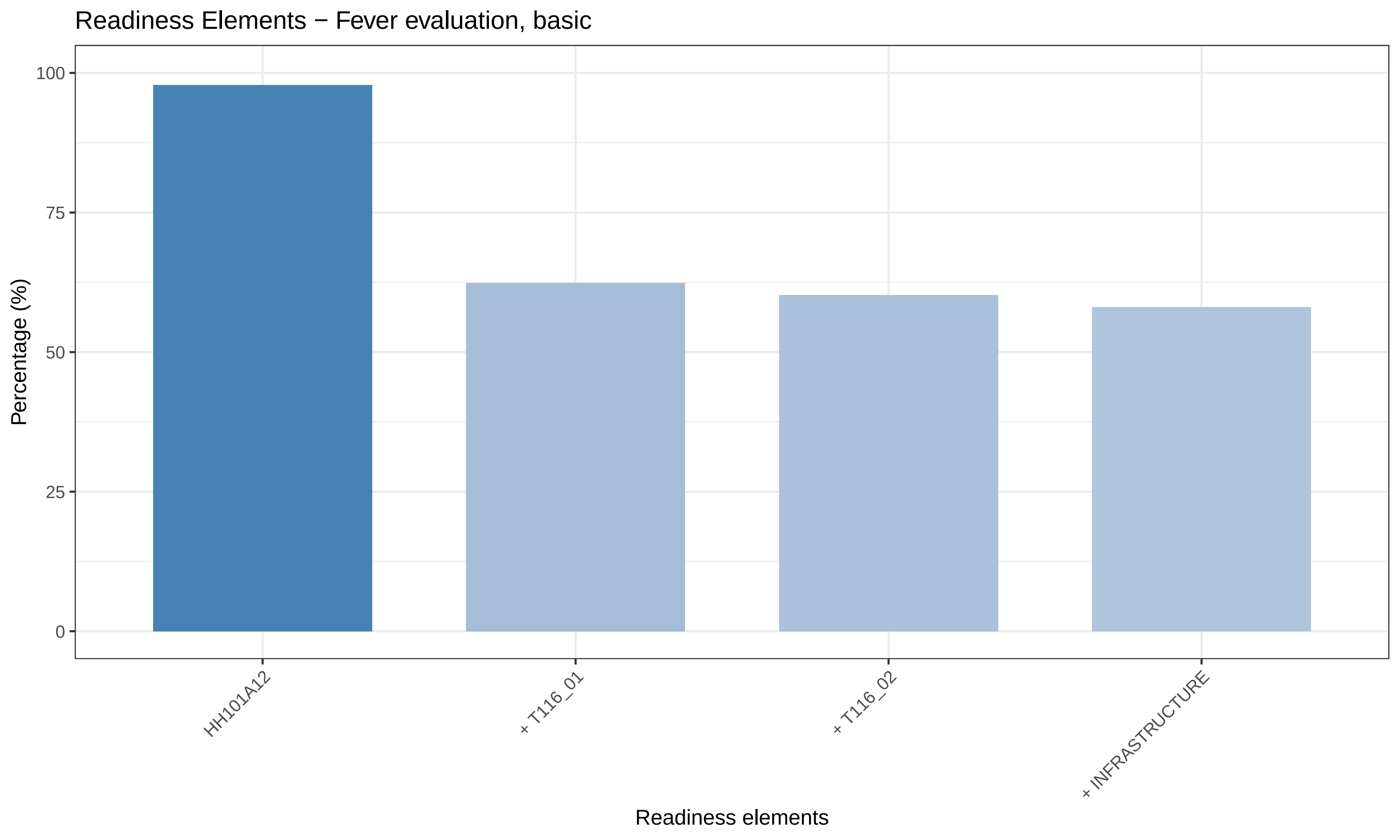
Readiness Elements − Fever evaluation, basic
Percentage (%)
100
75
50
25
0
HH101A12
+ T116_01
+ T116_02
+ INFRASTRUCTURE
Readiness elements

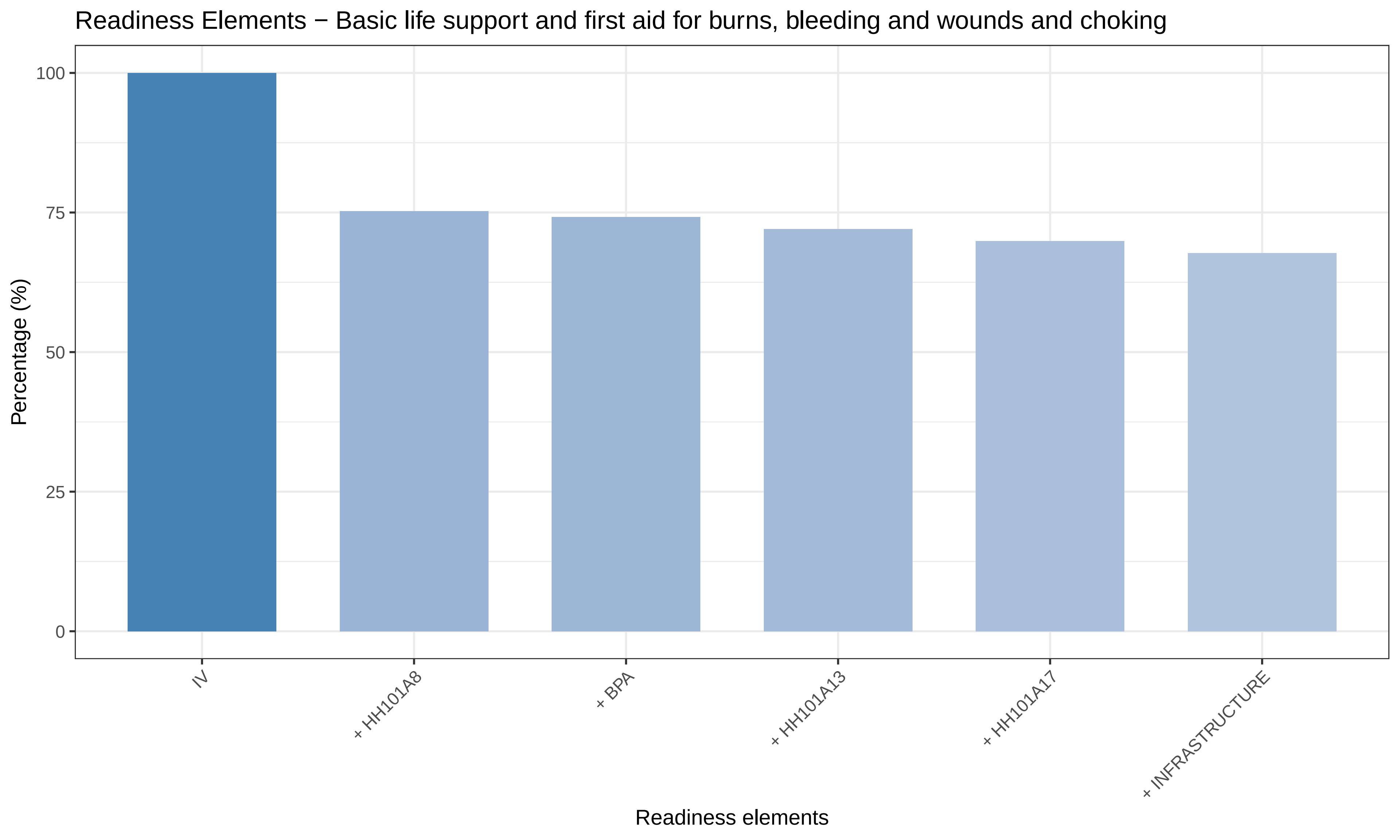
Readiness Elements − Basic life support and first aid for burns, bleeding and wounds and choking
Percentage (%)
100
75
50
25
0
IV
+ HH101A8
+ BPA
+ HH101A13
+ HH101A17
+ INFRASTRUCTURE
Readiness elements

Readiness Elements − Resuscitation with advanced life support measures

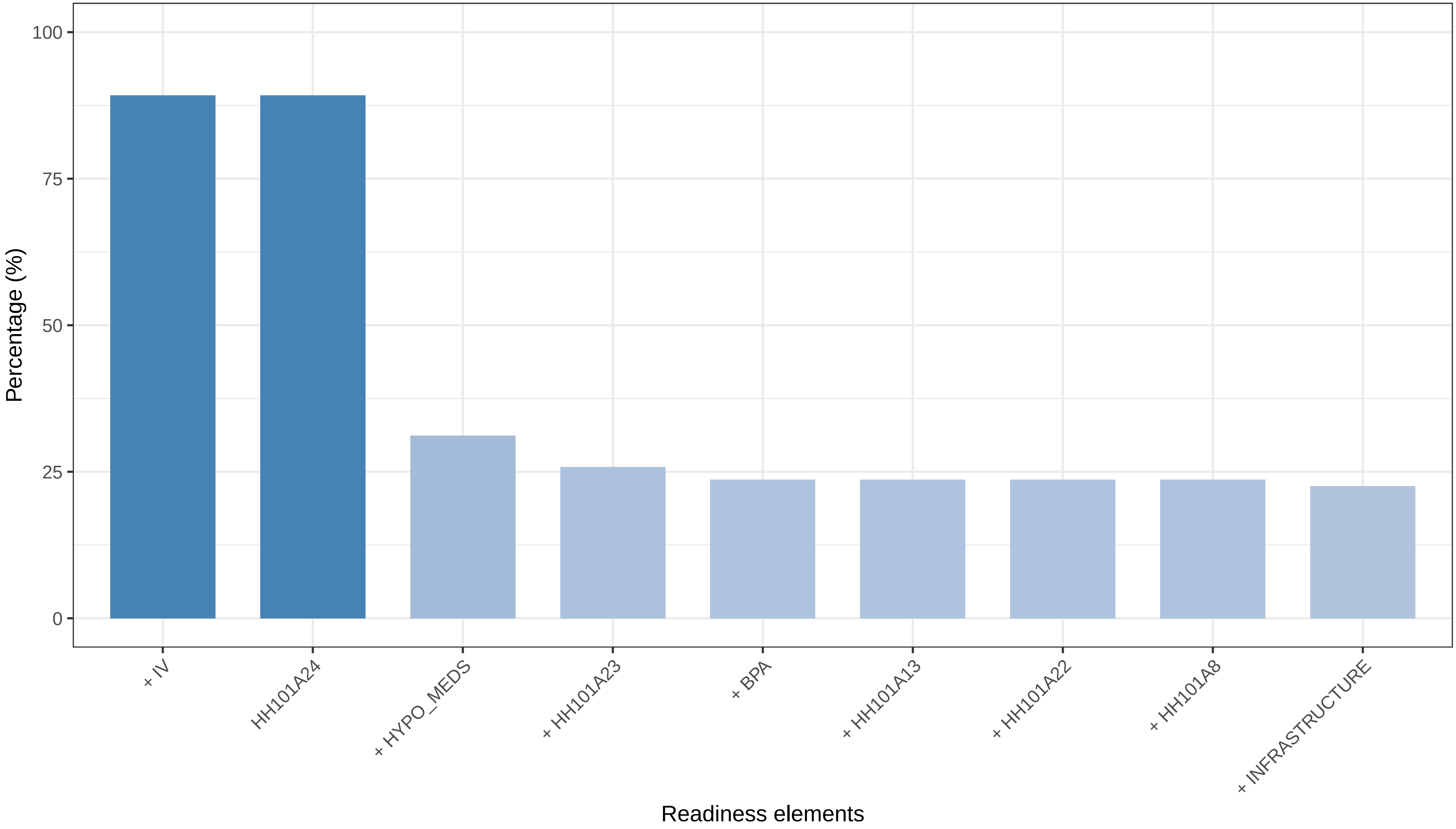

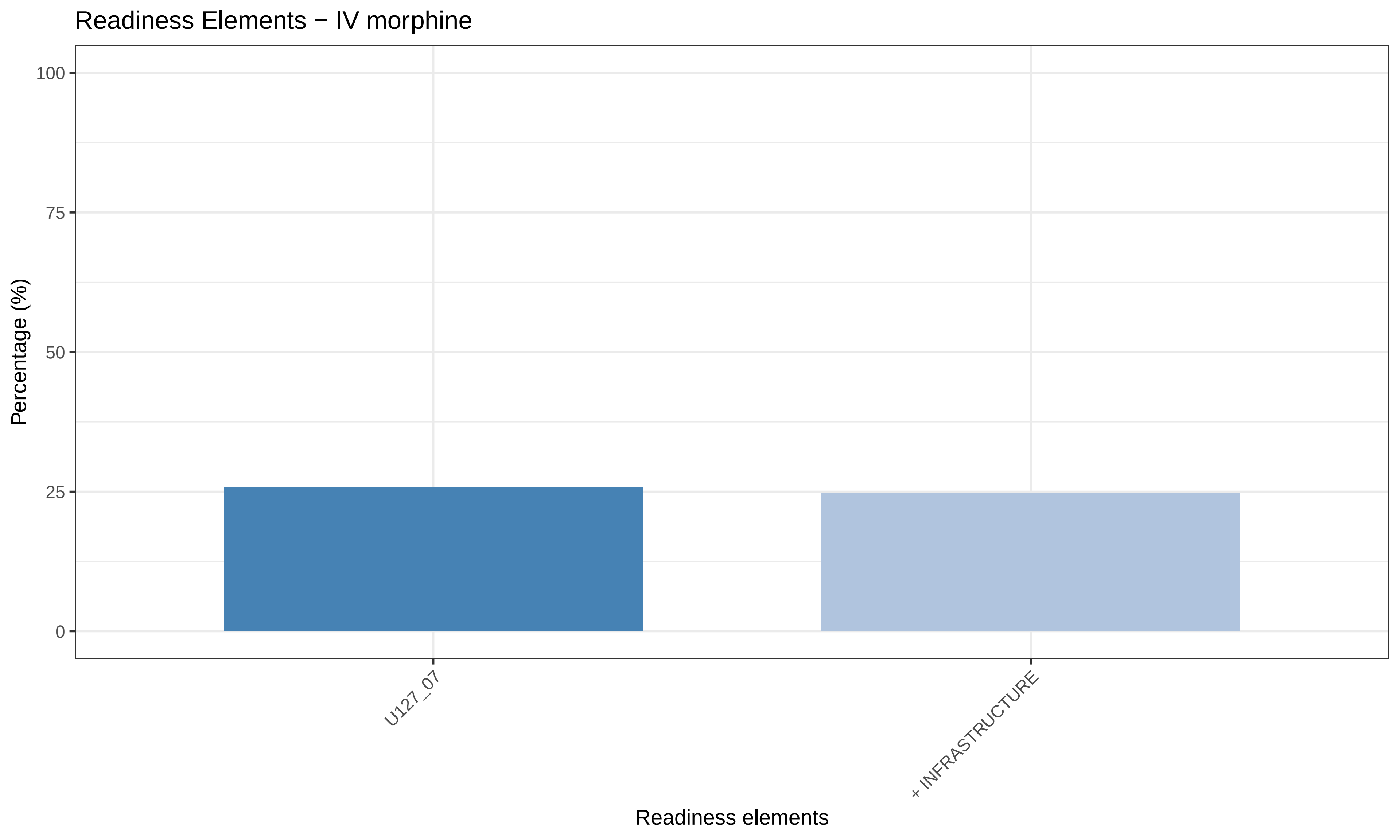
Readiness Elements − IV morphine
Percentage (%)
100
75
50
25
0
U127_07
+ INFRASTRUCTURE
Readiness elements

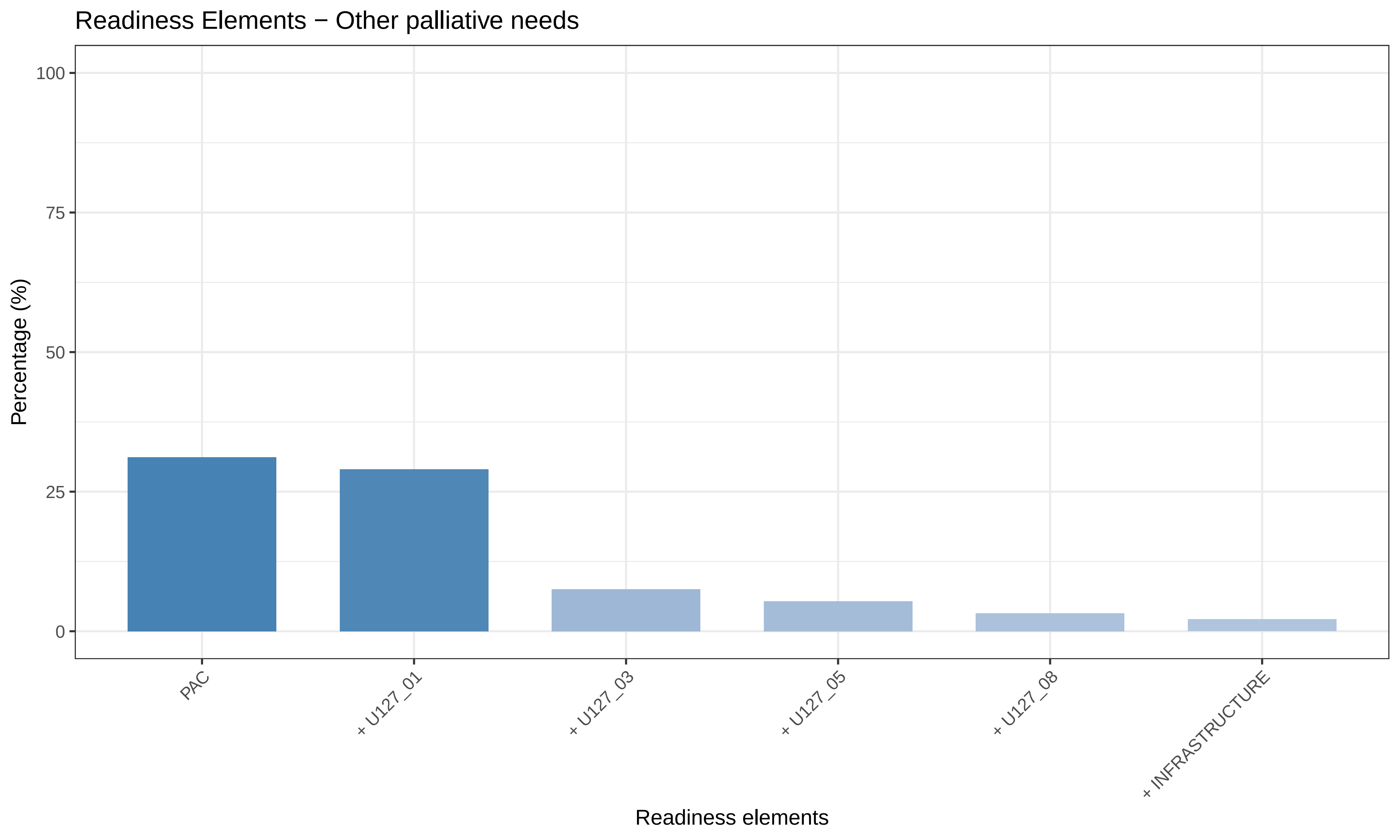
Readiness Elements − Other palliative needs
Percentage (%)
100
75
50
25
0
PAC
+ U127_01
+ U127_03
+ U127_05
+ U127_08
+ INFRASTRUCTURE
Readiness elements

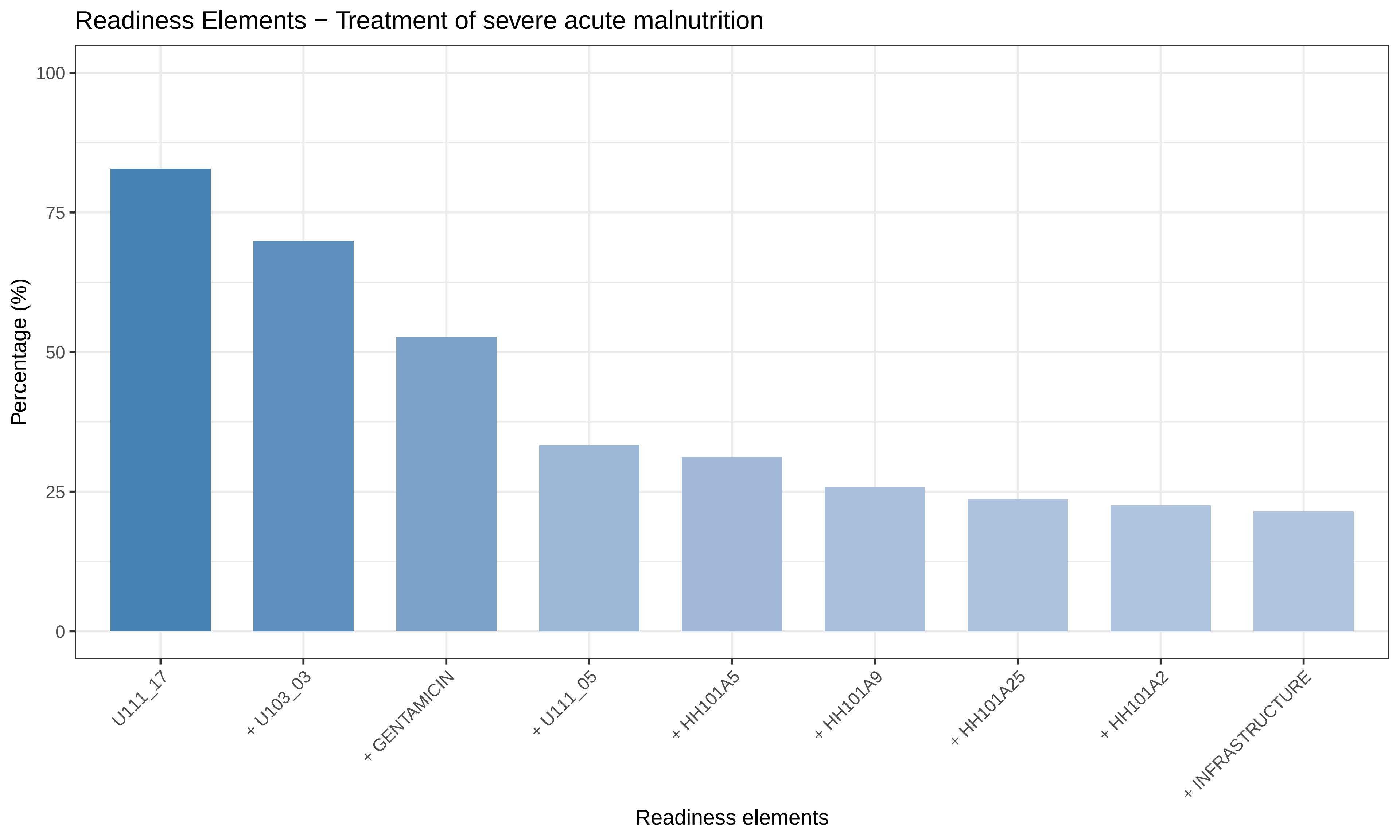
Readiness Elements − Treatment of severe acute malnutrition
Percentage (%)
100
75
50
25
0
U111_17
+ U103_03
+ GENTAMICIN
+ U111_05
+ HH101A5
+ HH101A9
+ HH101A25
+ HH101A2
+ INFRASTRUCTURE
Readiness elements

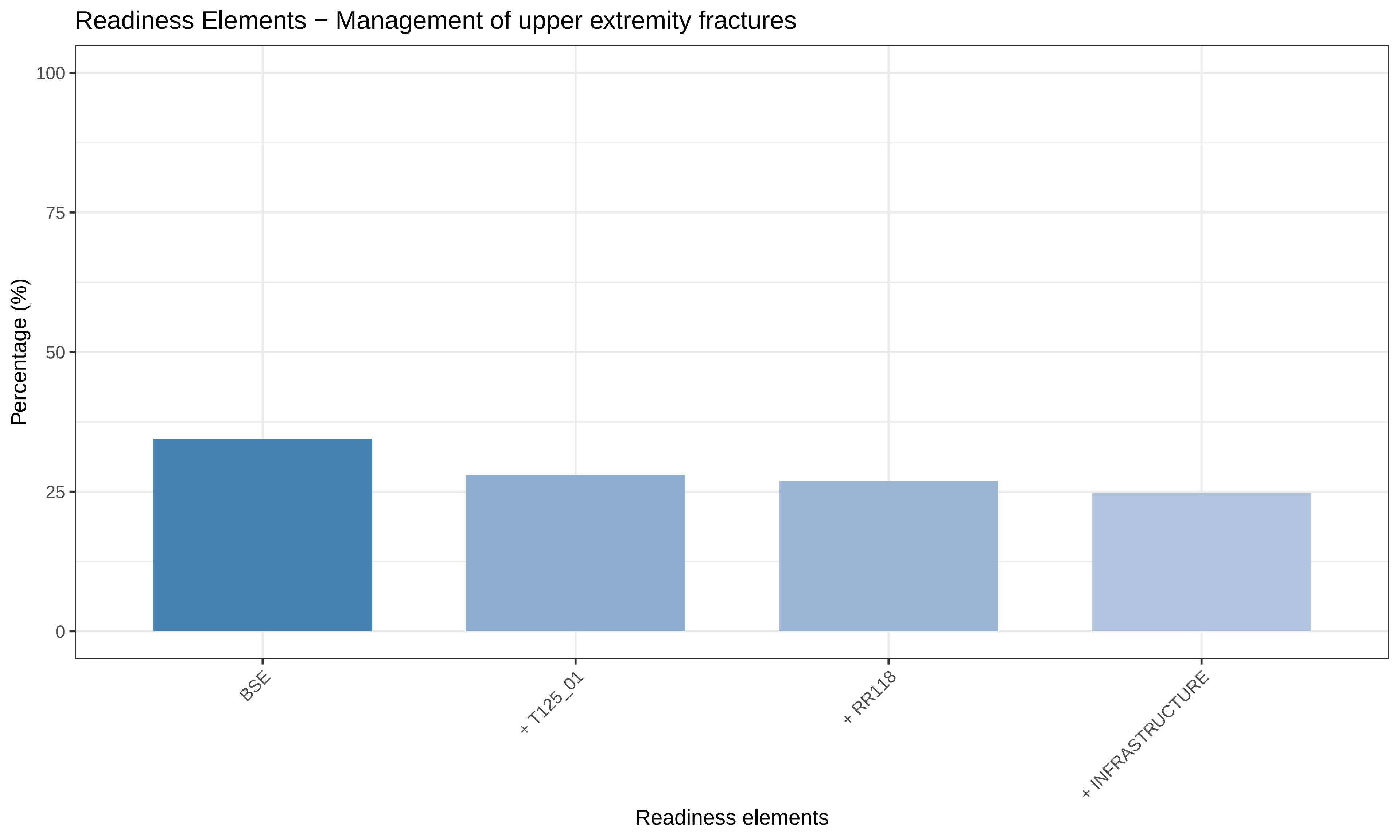
Readiness Elements − Management of upper extremity fractures
Percentage (%)
100
75
50
25
0
BSE
+ T125_01
+ RR118
+ INFRASTRUCTURE
Readiness elements

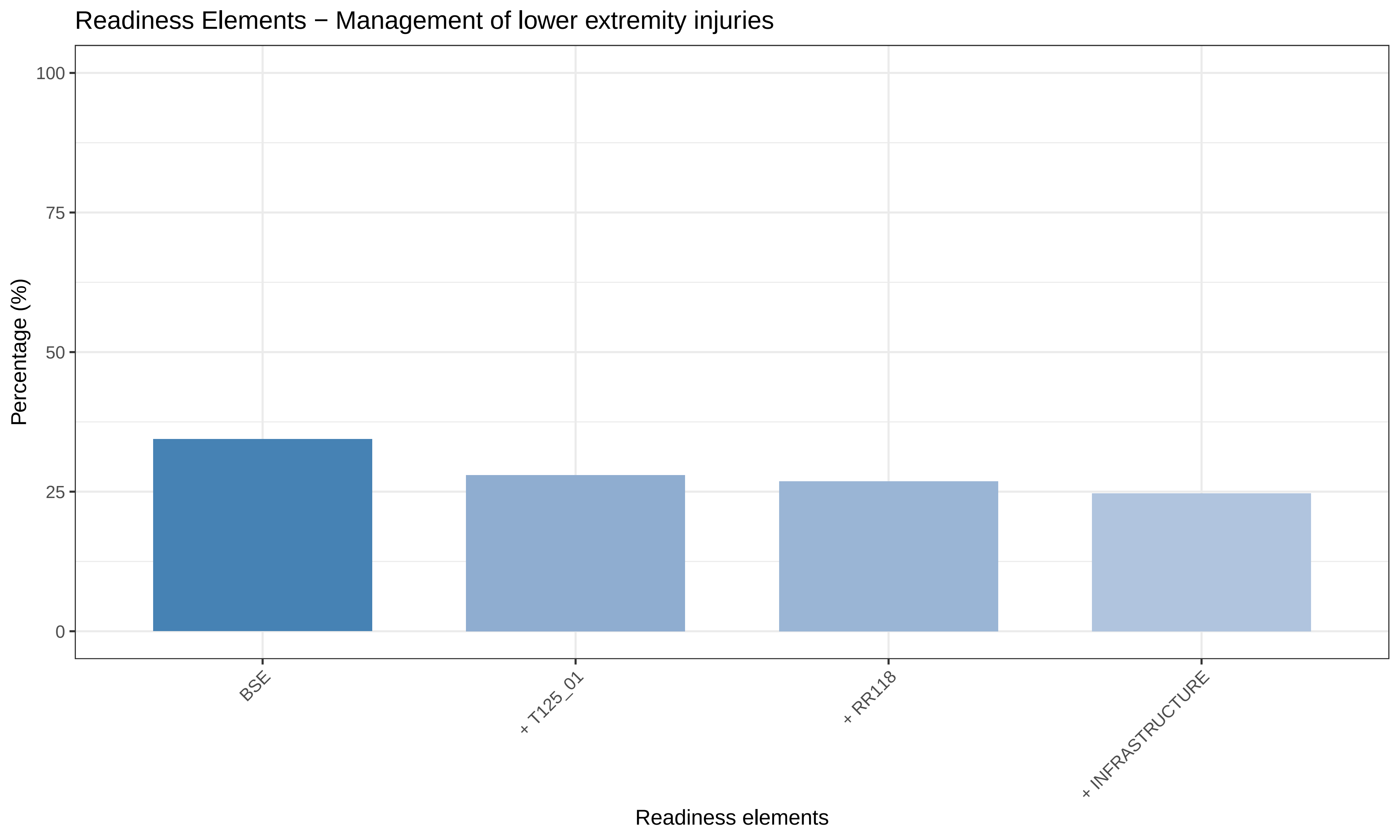

Readiness Elements − Management of lower extremity injuries
Percentage (%)
100
75
50
25
0
BSE
+ T125_01
+ RR118
+ INFRASTRUCTURE
Readiness elements

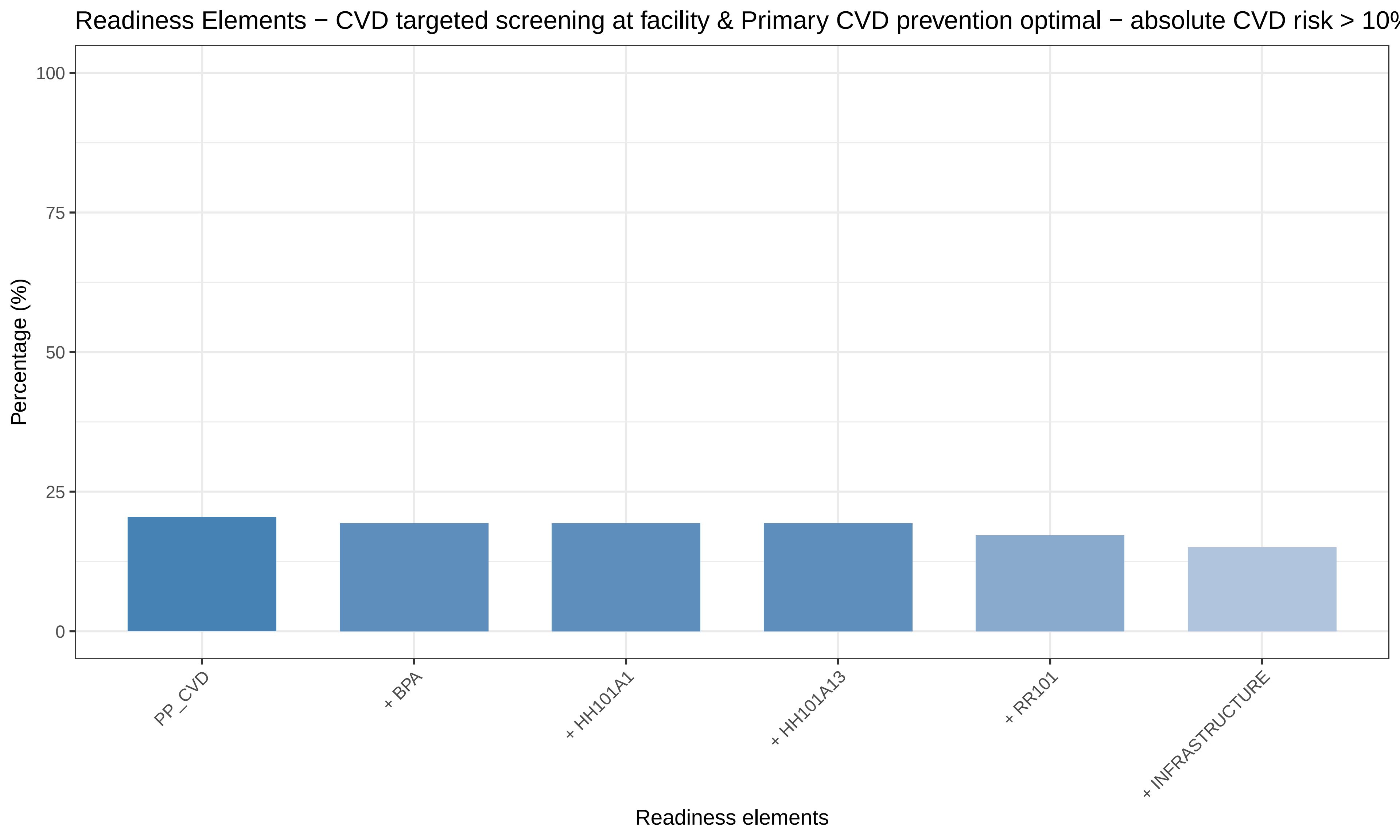
Readiness Elements − CVD targeted screening at facility & Primary CVD prevention optimal − absolute CVD risk > 10%
Percentage (%)
100
75
50
25
0
PP_CVD
+ BPA
+ HH101A1
+ HH101A13
+ RR101
+ INFRASTRUCTURE
Readiness elements

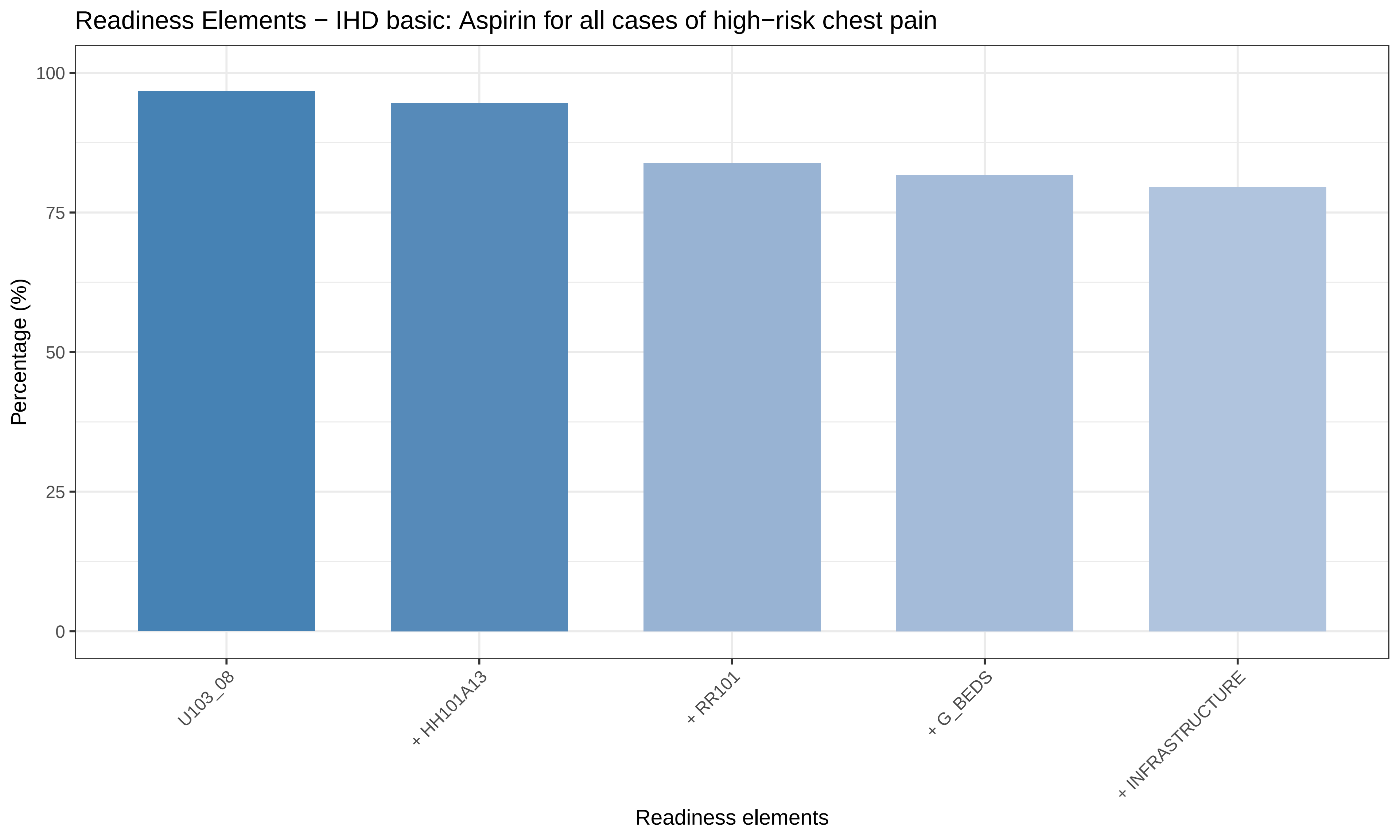
Readiness Elements − IHD basic: Aspirin for all cases of high−risk chest pain
Percentage (%)
100
75
50
25
0
U103_08
+ HH101A13
+ RR101
+ G_BEDS
+ INFRASTRUCTURE
Readiness elements

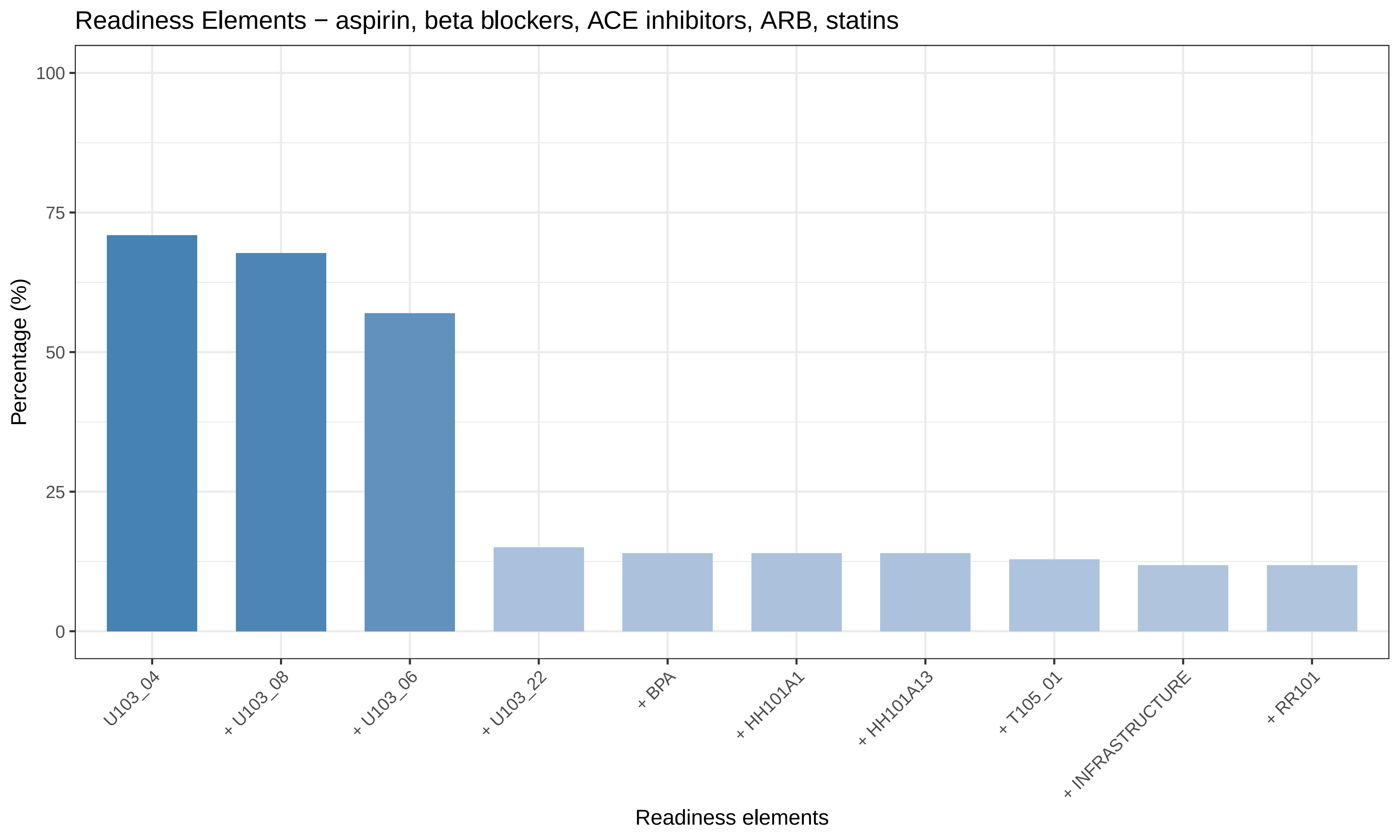
Readiness Elements − aspirin, beta blockers, ACE inhibitors, ARB, statins
Percentage (%)
100
75
50
25
0
U103_04
+ U103_08
+ U103_06
+ U103_22
+ BPA
+ HH101A1
+ HH101A13
+ T105_01
+ INFRASTRUCTURE
+ RR101
Readiness elements

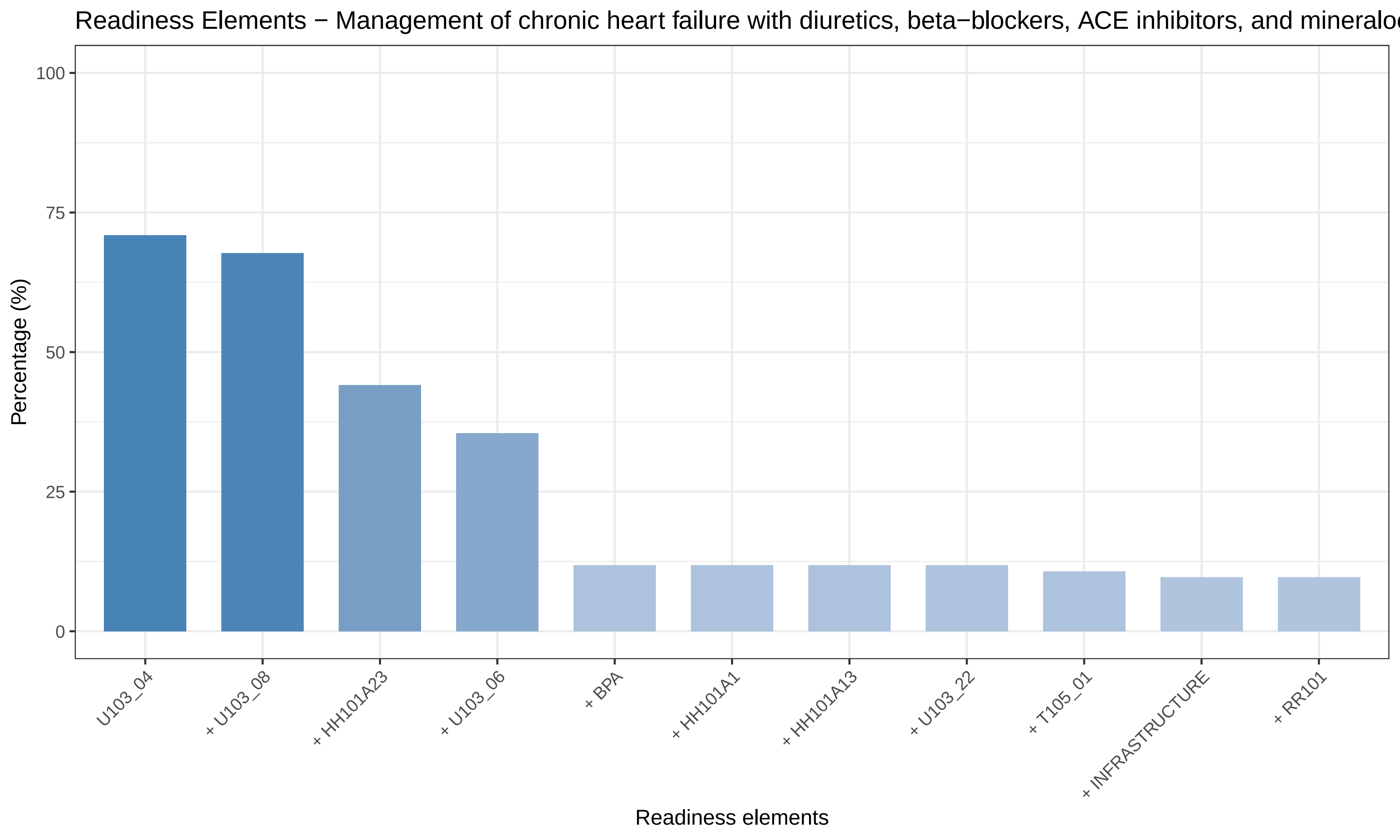

Readiness Elements − Management of chronic heart failure with diuretics, beta−blockers, ACE inhibitors, and mineralo
Percentage (%)
100
75
50
25
0
U103_04
+ U103_08
+ HH101A23
+ U103_06
+ BPA
+ HH101A1
+ HH101A13
+ U103_22
+ T105_01
+ INFRASTRUCTURE
+ RR101
Readiness elements

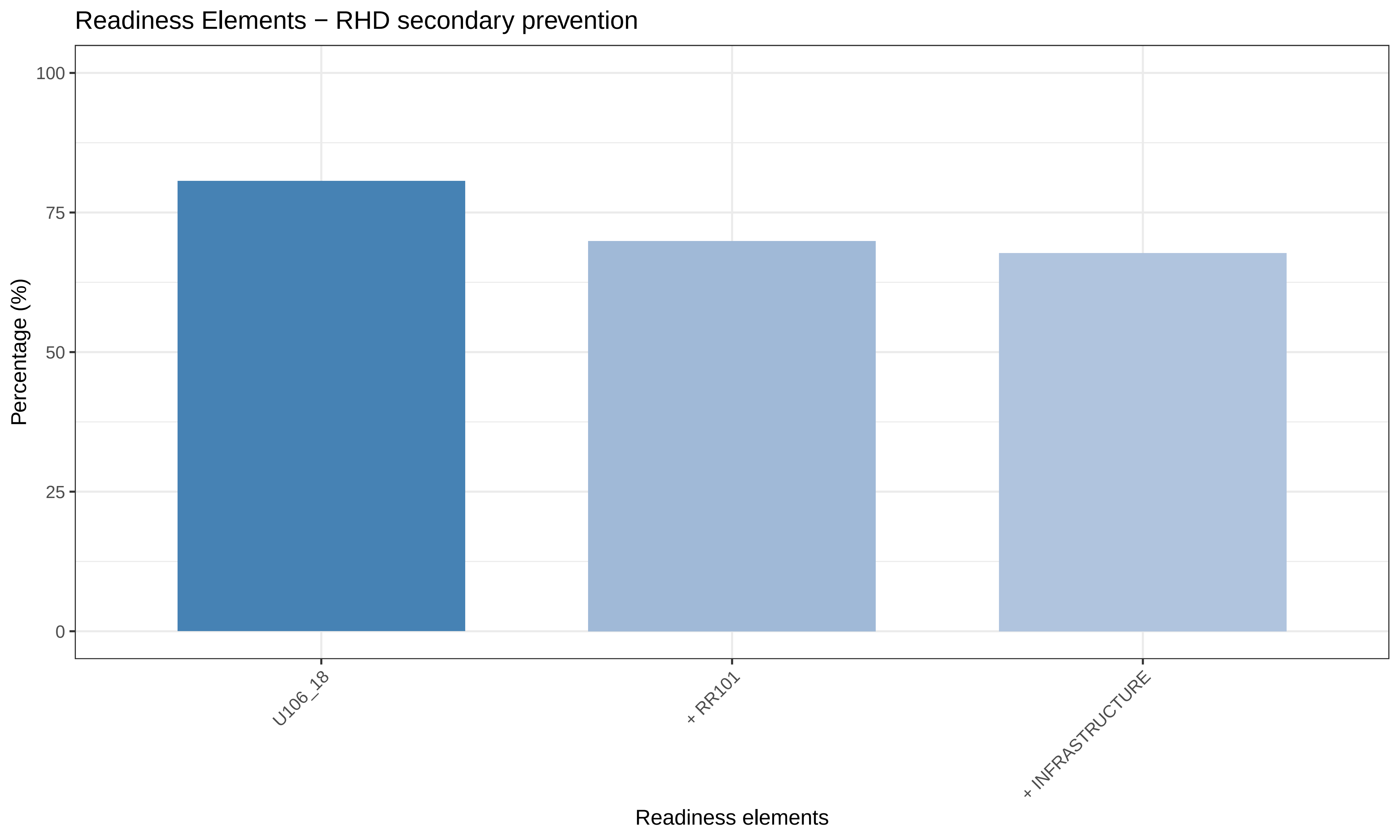
Readiness Elements − RHD secondary prevention
100
75
50
25
0
Percentage (%)
U106_18
+ RR101
+ INFRASTRUCTURE
Readiness elements

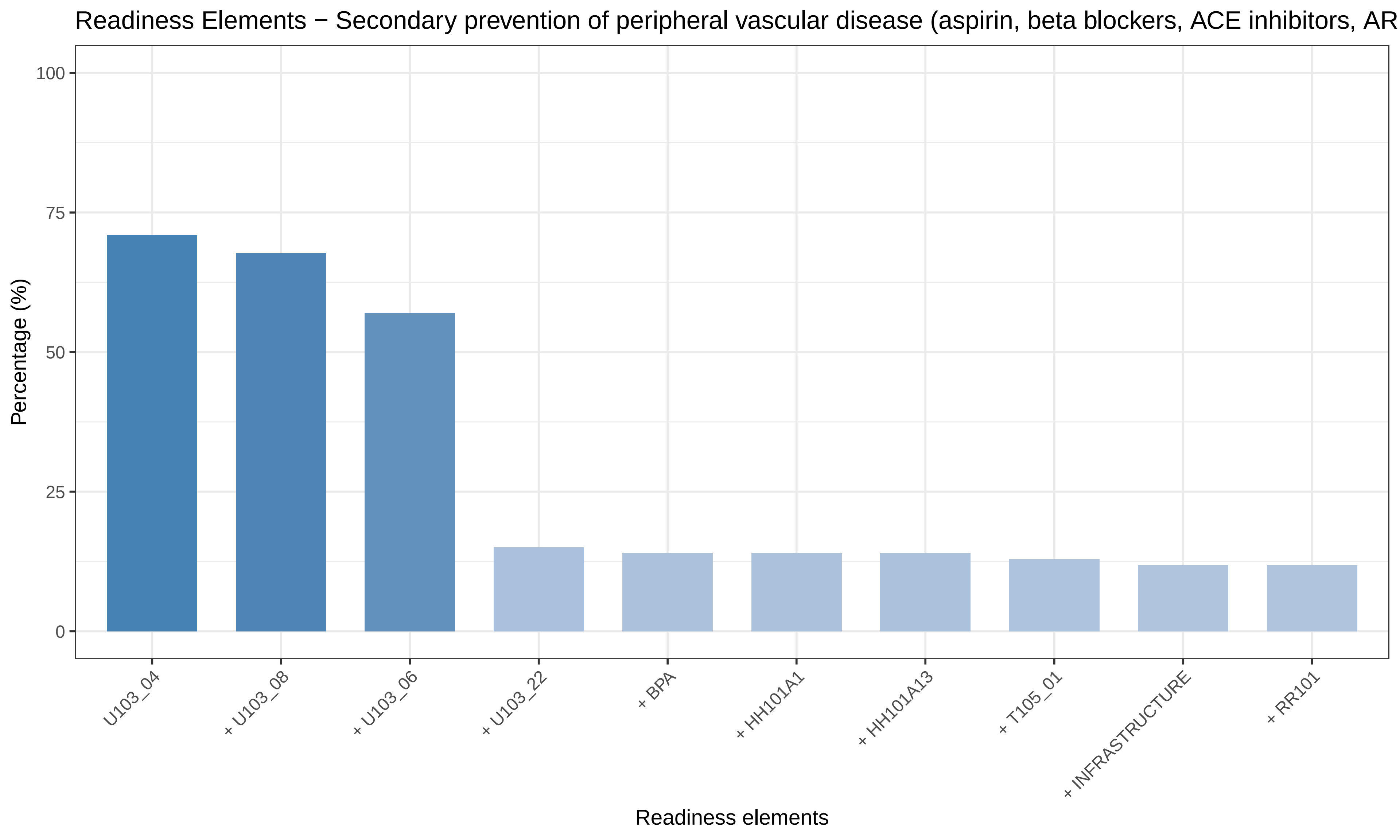
Readiness Elements − Secondary prevention of peripheral vascular disease (aspirin, beta blockers, ACE inhibitors, AR
Percentage (%)
100
75
50
25
0
U103_04
+ U103_08
+ U103_06
+ U103_22
+ BPA
+ HH101A1
+ HH101A13
+ T105_01
+ INFRASTRUCTURE
+ RR101
Readiness elements

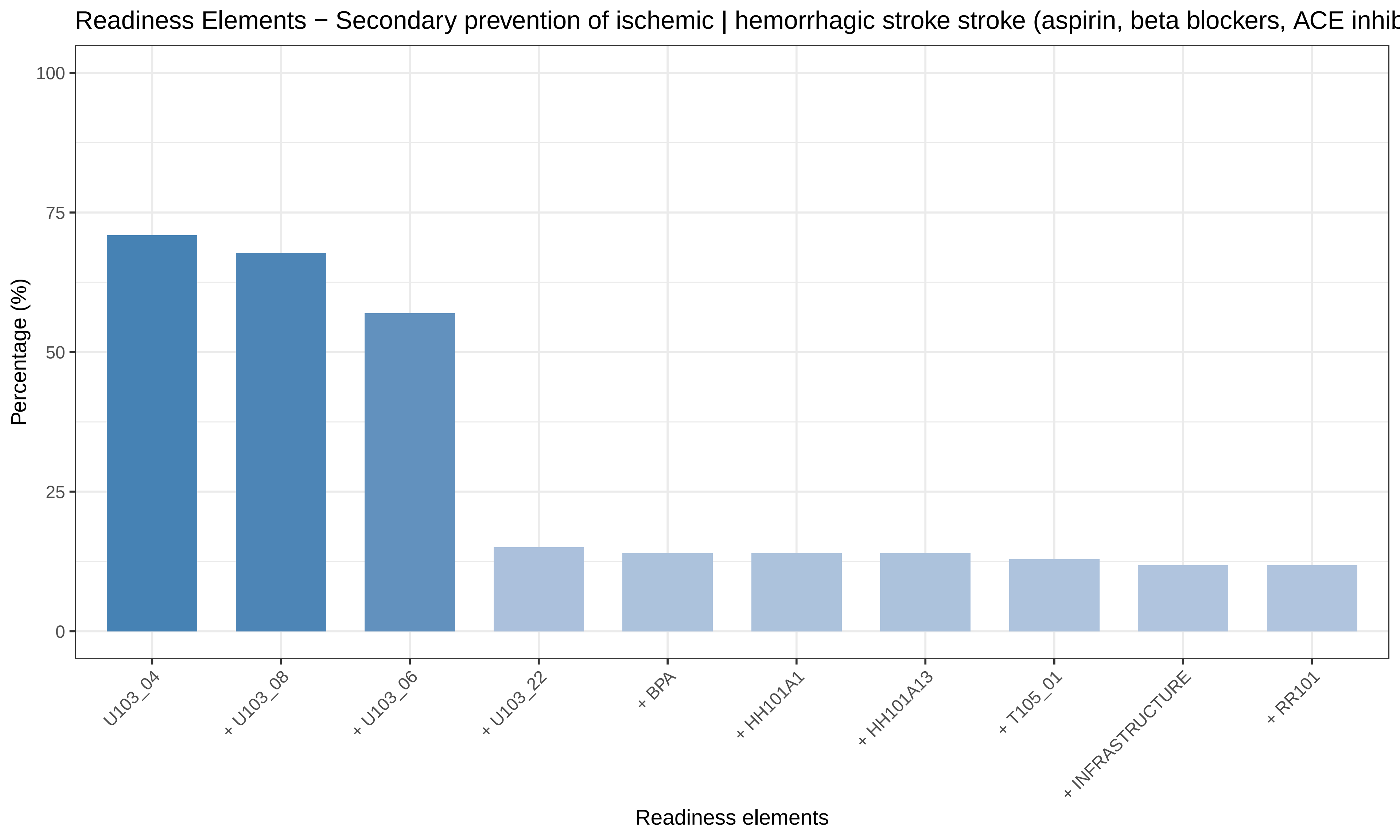

Readiness Elements − Secondary prevention of ischemic | hemorrhagic stroke stroke (aspirin, beta blockers, ACE inhib
Percentage (%)
100
75
50
25
0
U103_04
+ U103_08
+ U103_06
+ U103_22
+ BPA
+ HH101A1
+ HH101A13
+ T105_01
+ INFRASTRUCTURE
+ RR101
Readiness elements

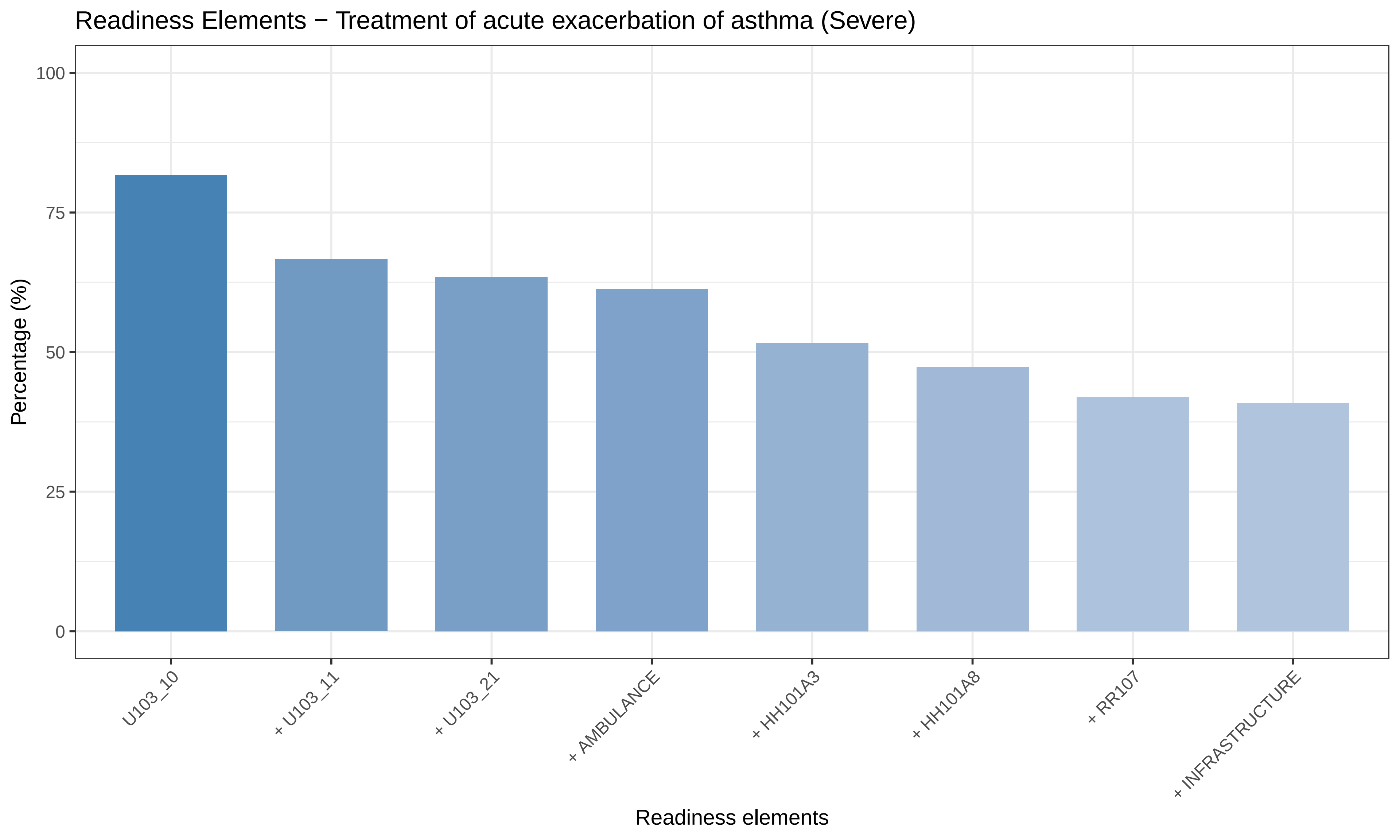
Readiness Elements − Treatment of acute exacerbation of asthma (Severe)
Percentage (%)
100
75
50
25
0
U103_10
+ U103_11
+ U103_21
+ AMBULANCE
+ HH101A3
+ HH101A8
+ RR107
+ INFRASTRUCTURE
Readiness elements

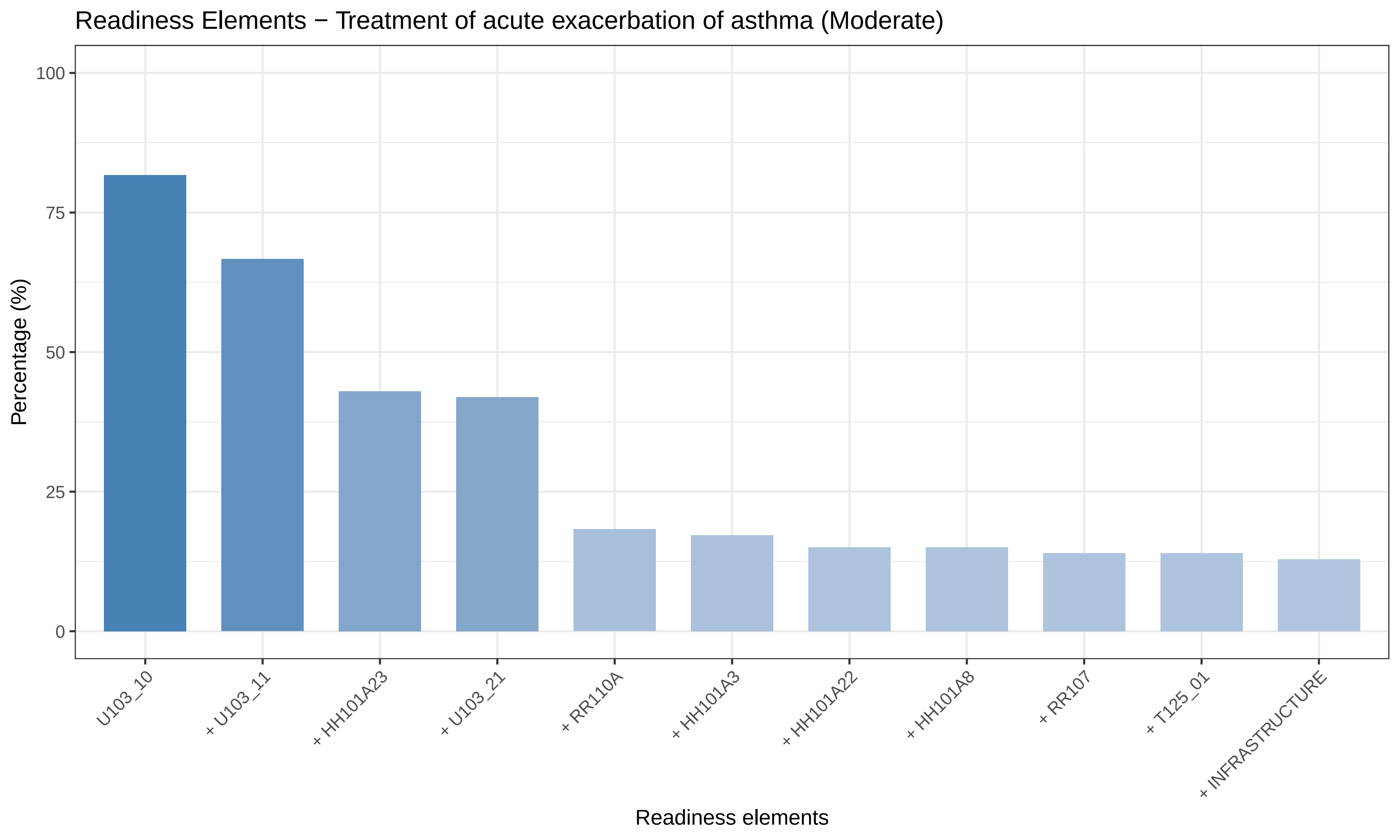
Readiness Elements − Treatment of acute exacerbation of asthma (Moderate)
Percentage (%)
100
75
50
25
0
U103_10
+ U103_11
+ HH101A23
+ U103_21
+ RR110A
+ HH101A3
+ HH101A22
+ HH101A8
+ RR107
+ T125_01
+ INFRASTRUCTURE
Readiness elements

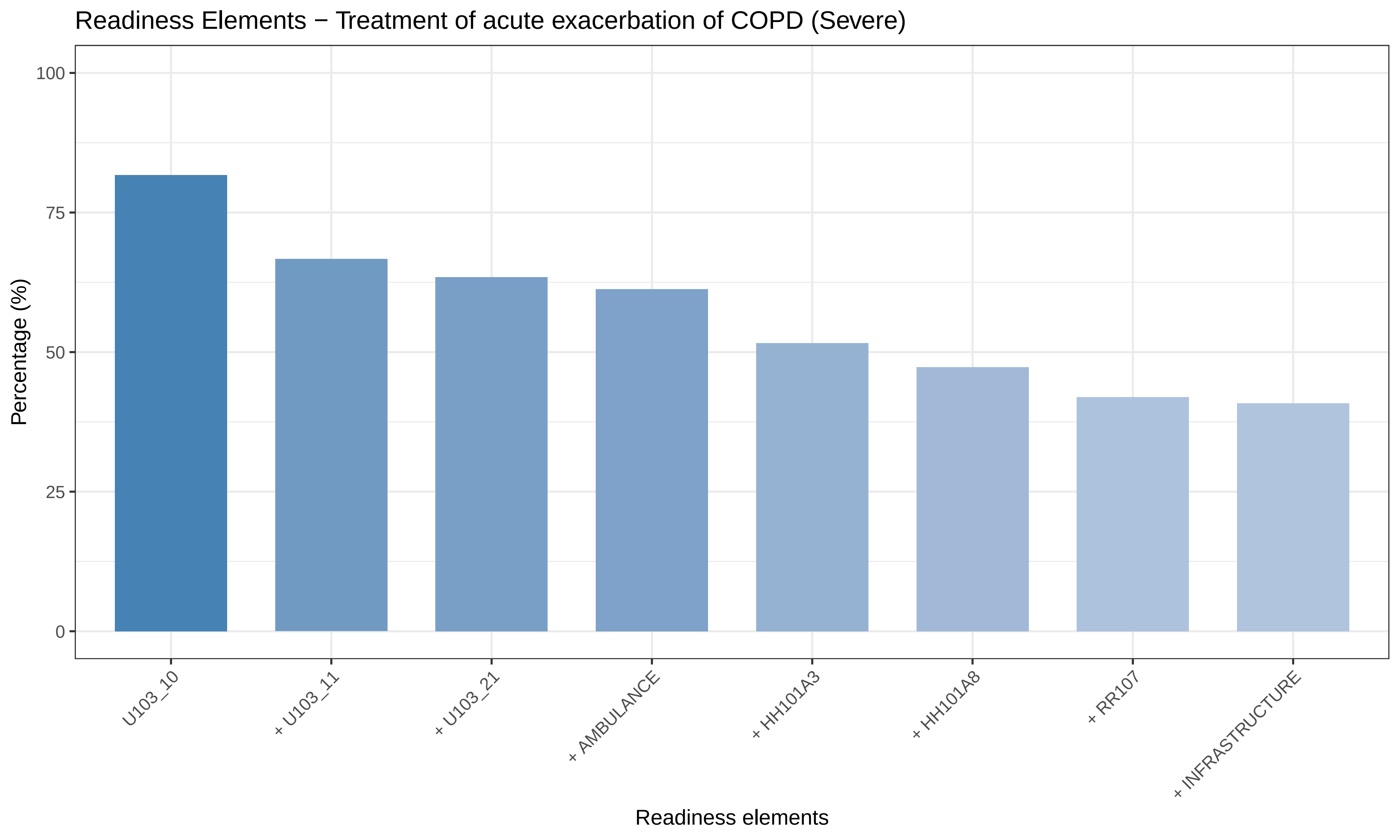
Readiness Elements − Treatment of acute exacerbation of COPD (Severe)
Percentage (%)
100
75
50
25
0
U103_10
+ U103_11
+ U103_21
+ AMBULANCE
+ HH101A3
+ HH101A8
+ RR107
+ INFRASTRUCTURE
Readiness elements

Readiness Elements − Management of acute exacerbation of COPD (Moderate)

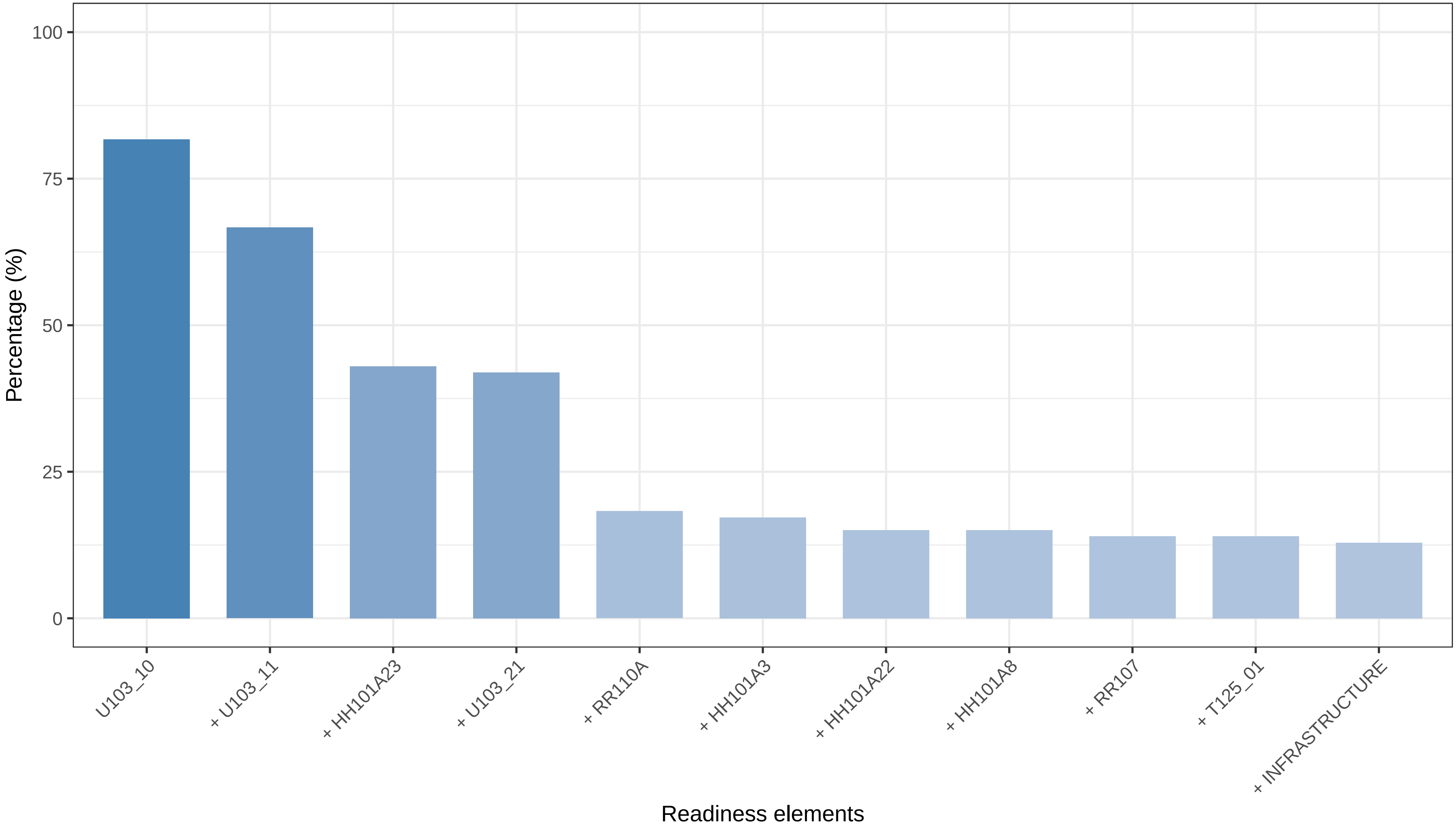

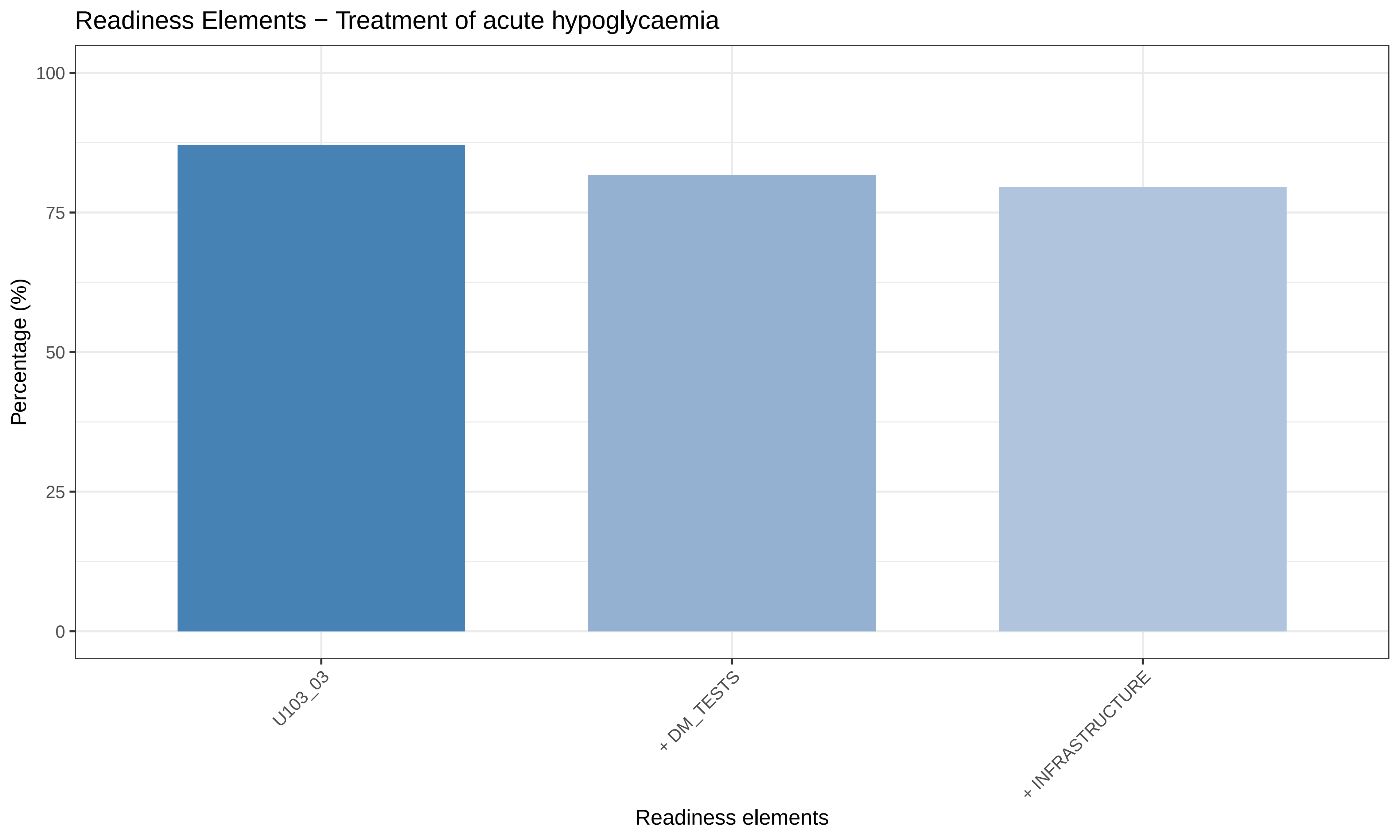

Readiness Elements − Treatment of acute hypoglycaemia
Percentage (%)
100
75
50
25
0
U103_03
+ DM_TESTS
+ INFRASTRUCTURE
Readiness elements

Readiness Elements − Treatment of acute hyperglycaemia

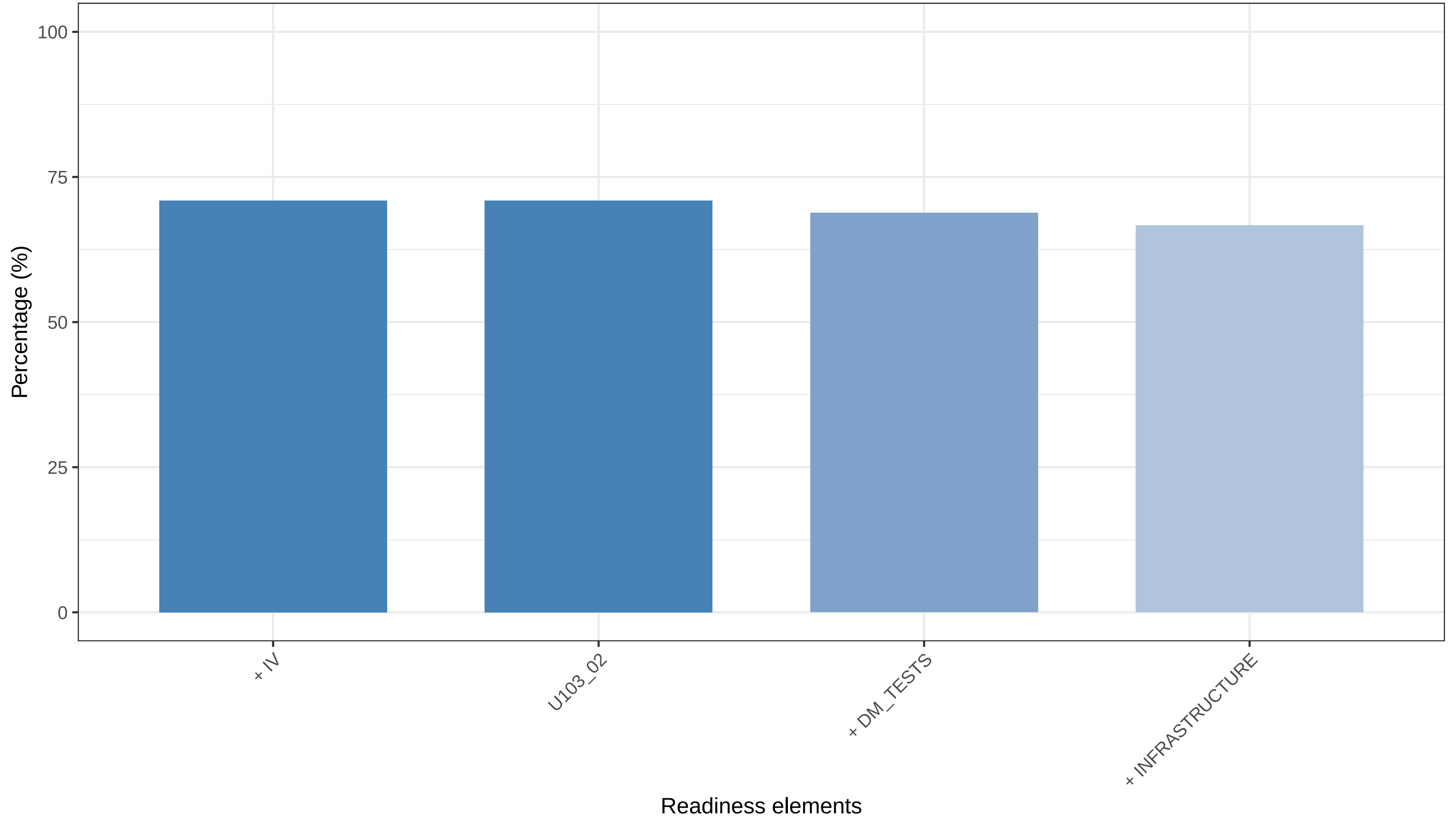

Readiness Elements − Management of anxiety disorders

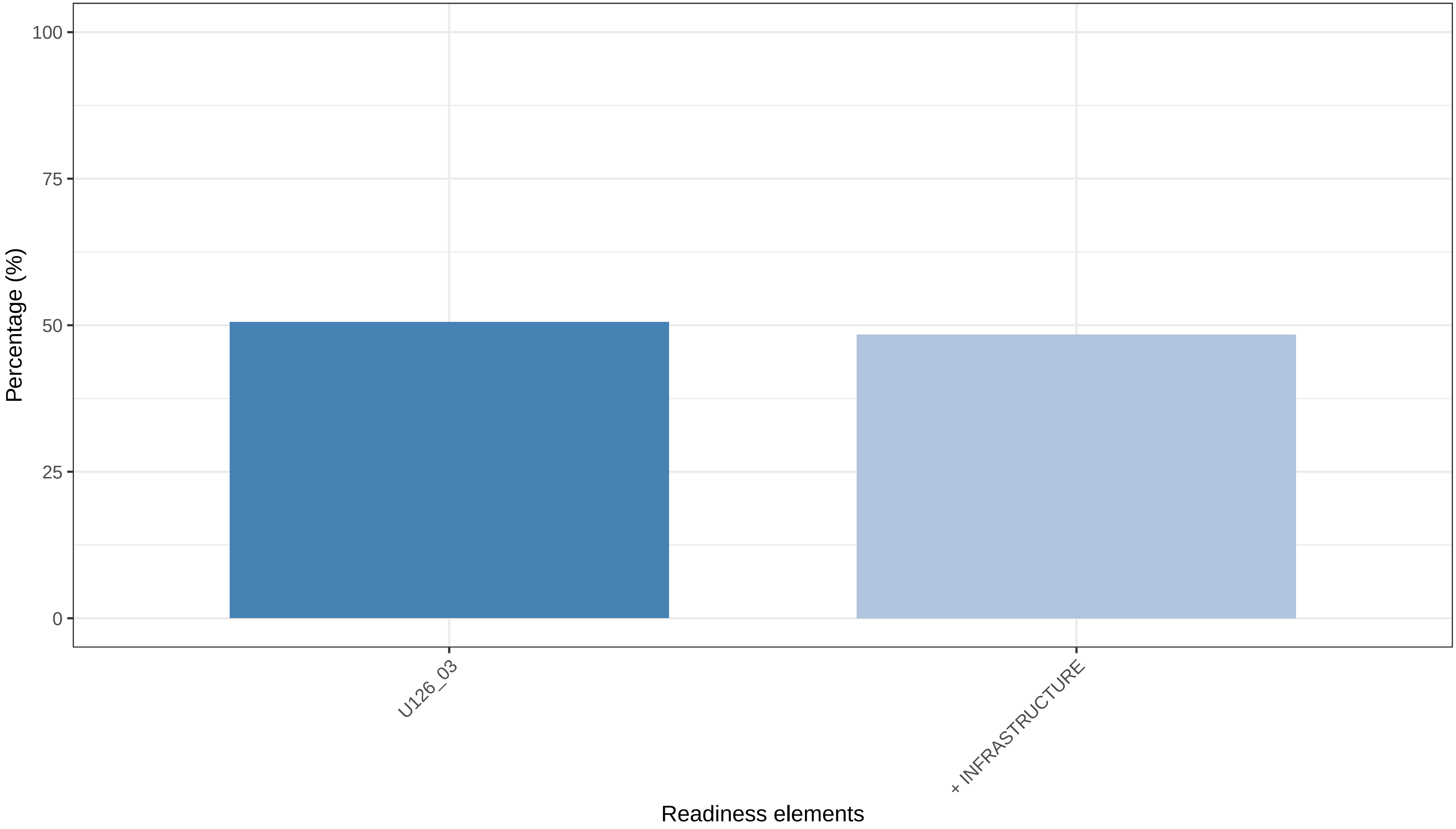

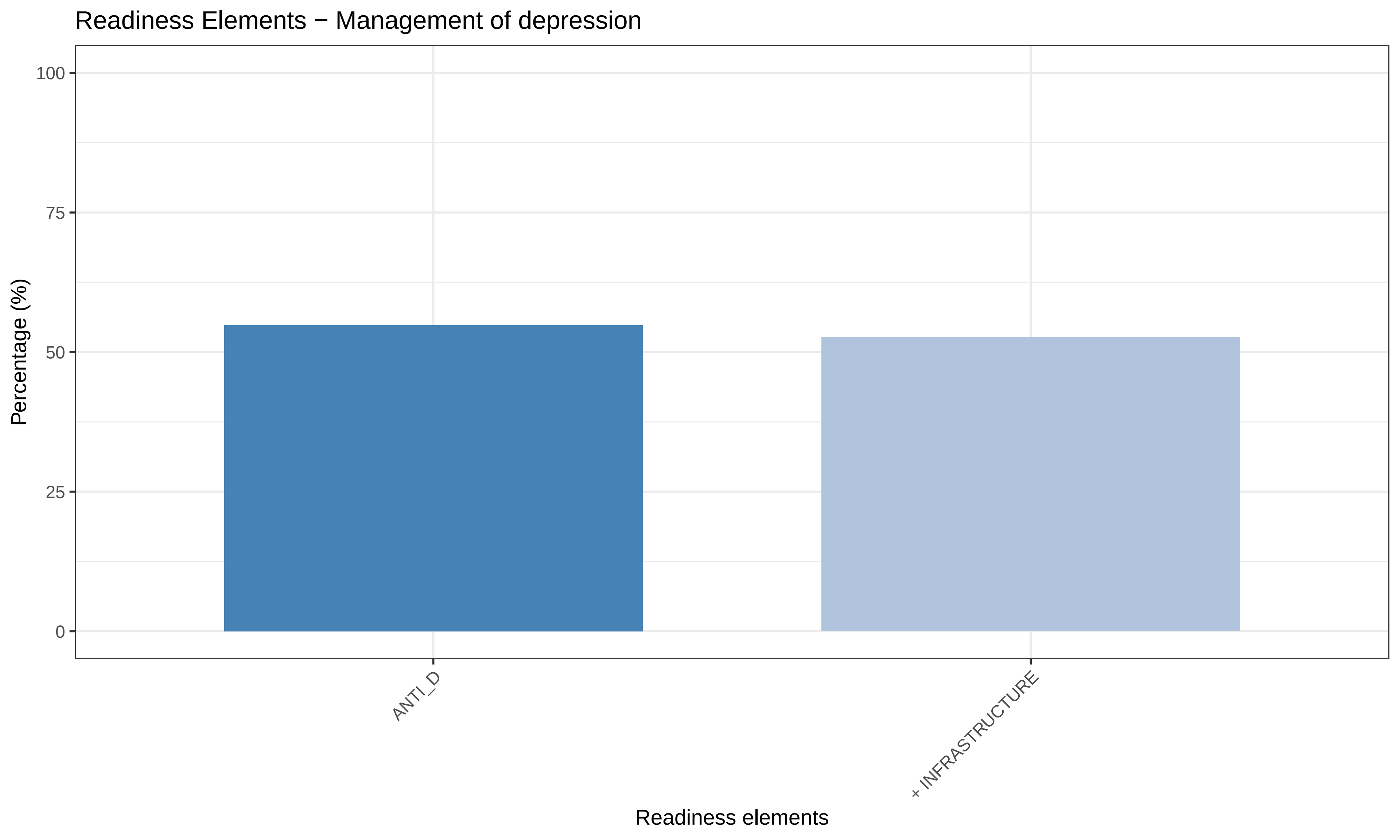
Readiness Elements − Management of depression
Percentage (%)
100
75
50
25
0
ANTI_D
+ INFRASTRUCTURE
Readiness elements

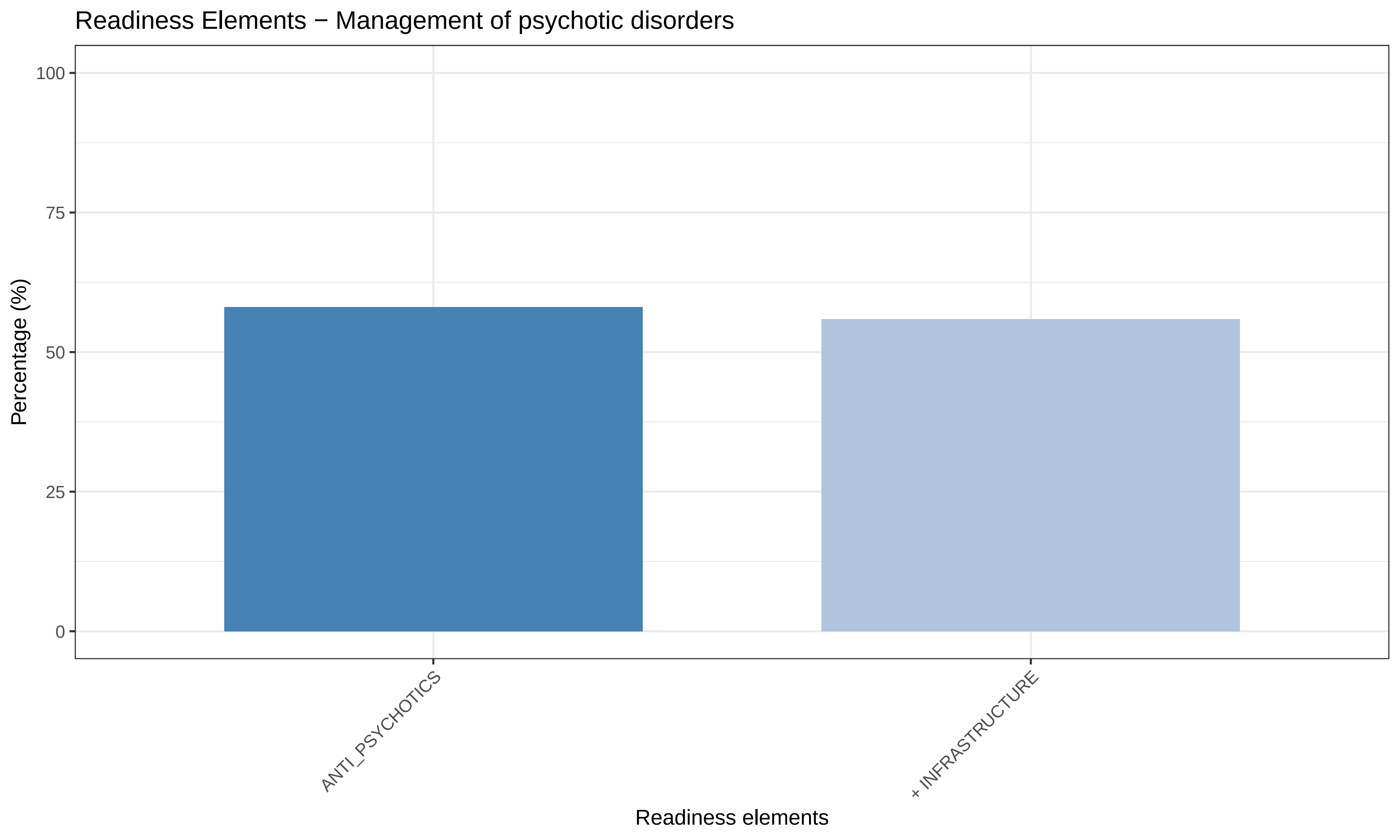
Readiness Elements − Management of psychotic disorders
Percentage (%)
100
75
50
25
0
ANTI_PSYCHOTICS
+ INFRASTRUCTURE
Readiness elements

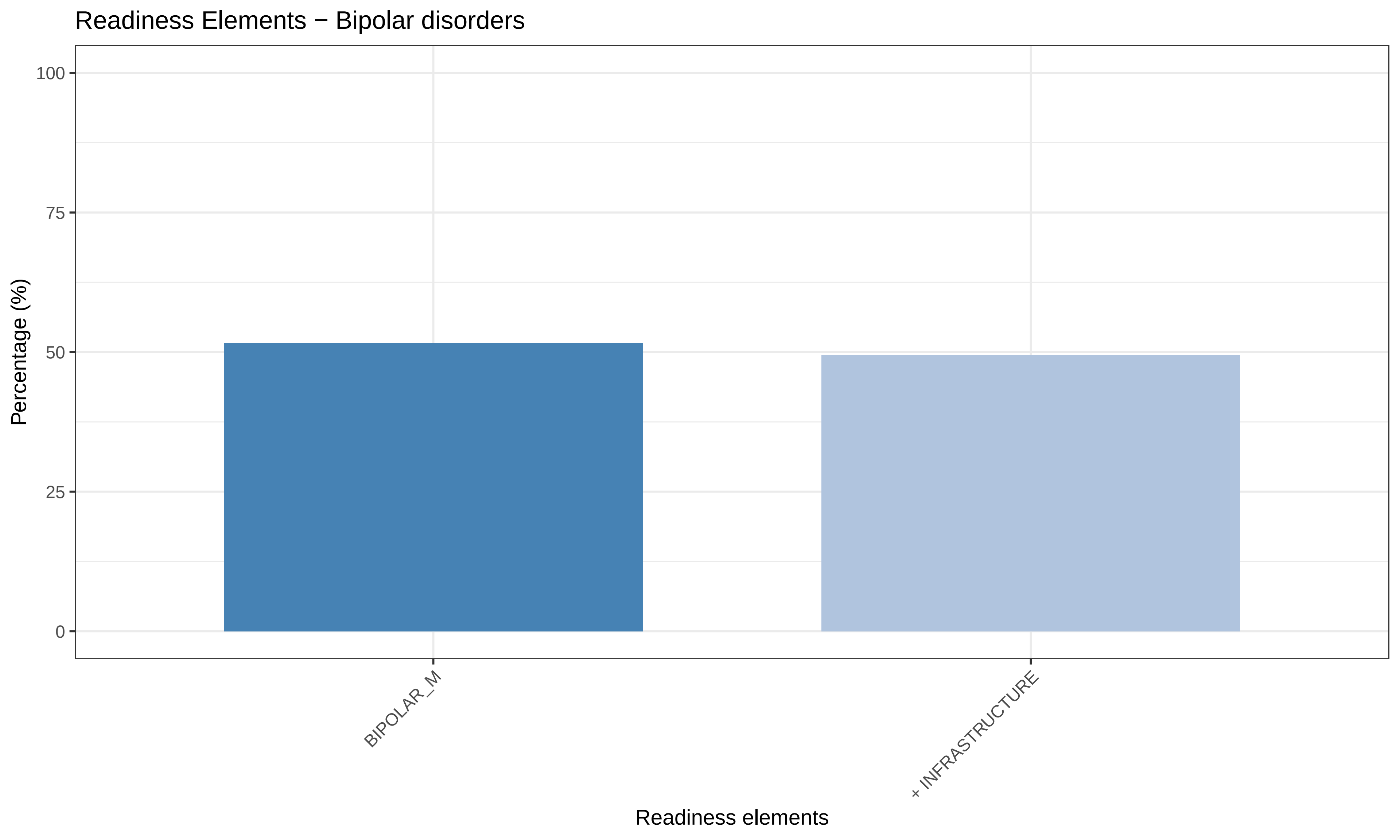

Readiness Elements − Bipolar disorders
100
75
50
25
0
Percentage (%)
BIPOLAR_M
+ INFRASTRUCTURE
Readiness elements

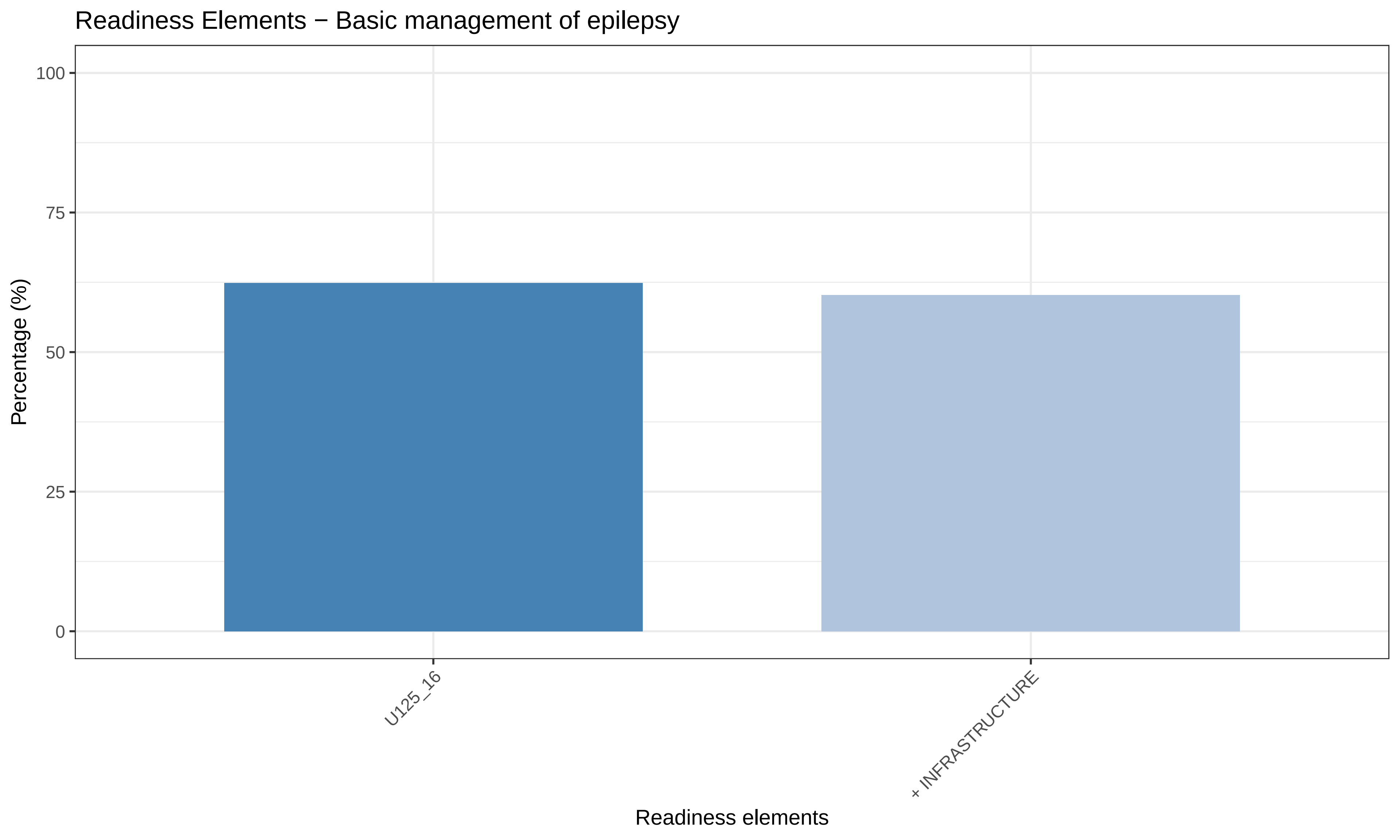
Readiness Elements − Basic management of epilepsy
Percentage (%)
100
75
50
25
0
U125_16
+ INFRASTRUCTURE
Readiness elements

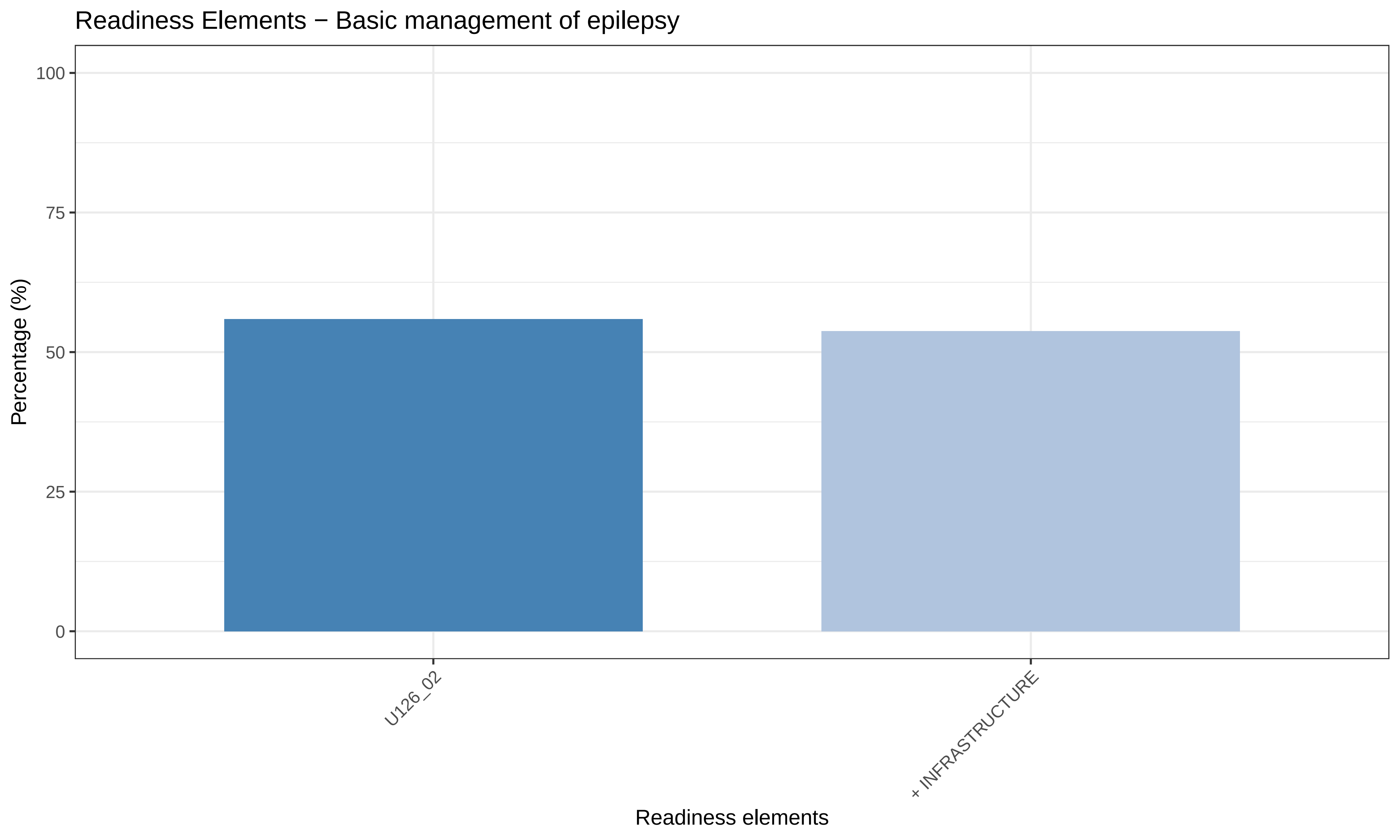

Readiness Elements − Basic management of epilepsy
Percentage (%)
100
75
50
25
0
U126_02
+ INFRASTRUCTURE
Readiness elements

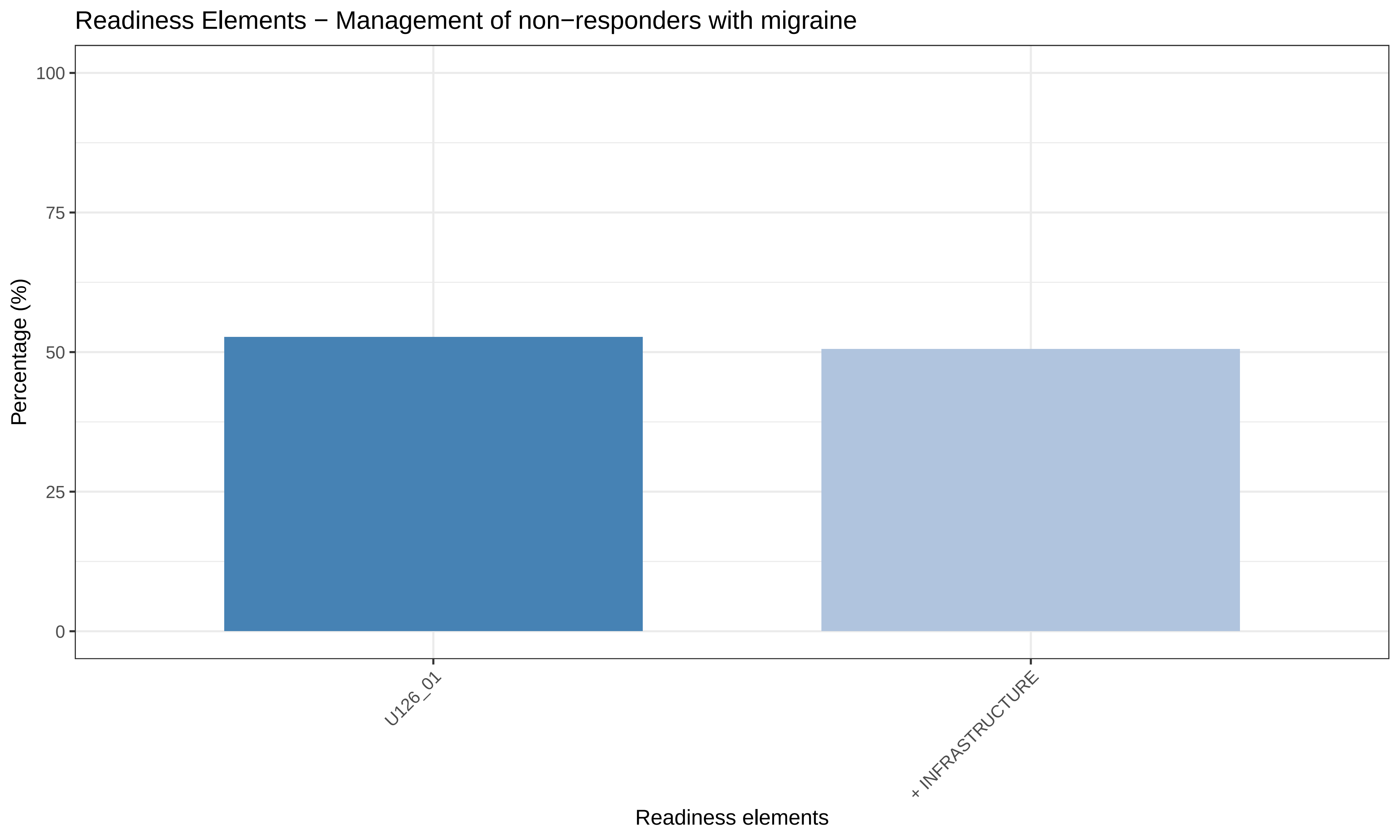
Readiness Elements − Management of non−responders with migraine
Percentage (%)
100
75
50
25
0
U126_01
+ INFRASTRUCTURE
Readiness elements

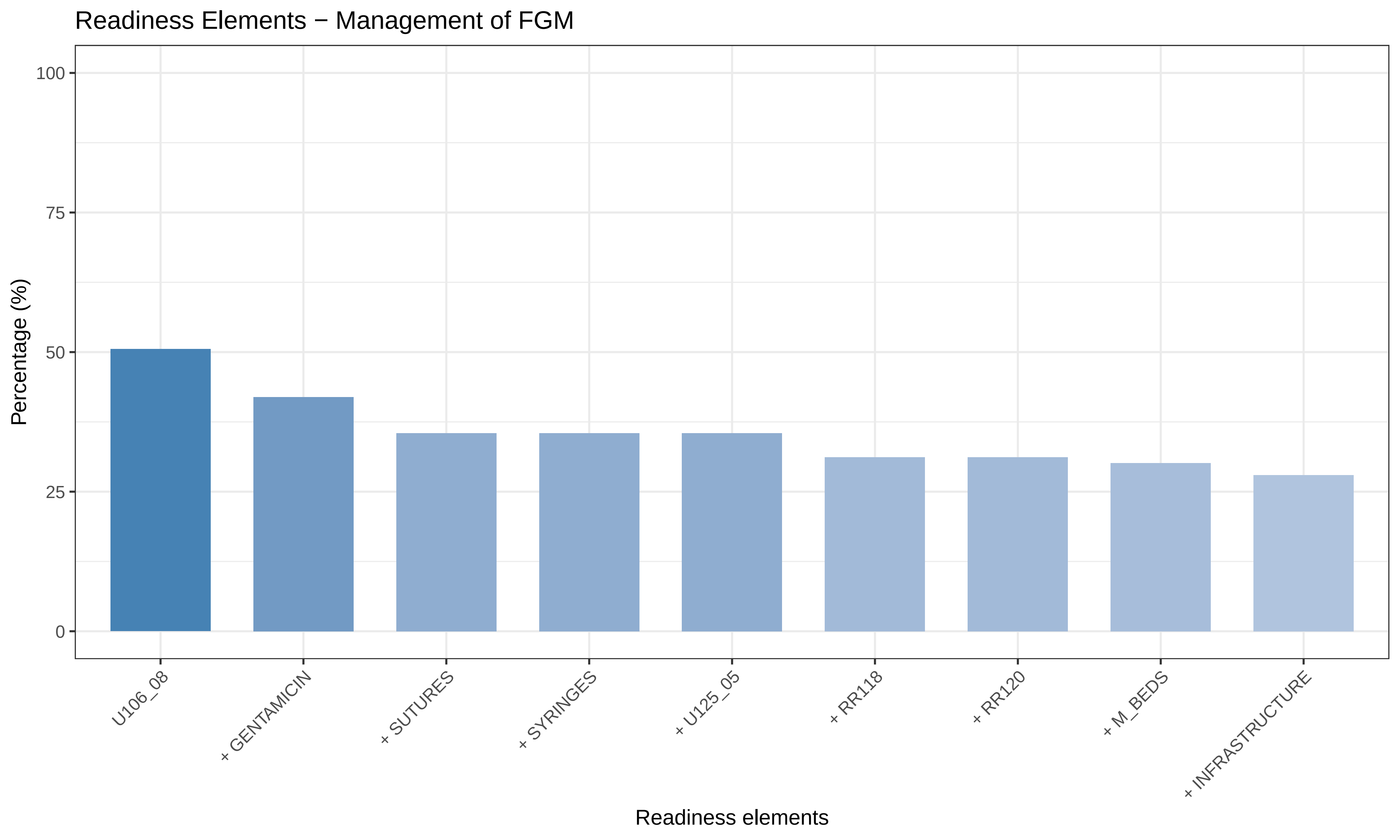
Readiness Elements − Management of FGM
Percentage (%)
100
75
50
25
0
U106_08
+ GENTAMICIN
+ SUTURES
+ SYRINGES
+ U125_05
+ RR118
+ RR120
+ M_BEDS
+ INFRASTRUCTURE
Readiness elements

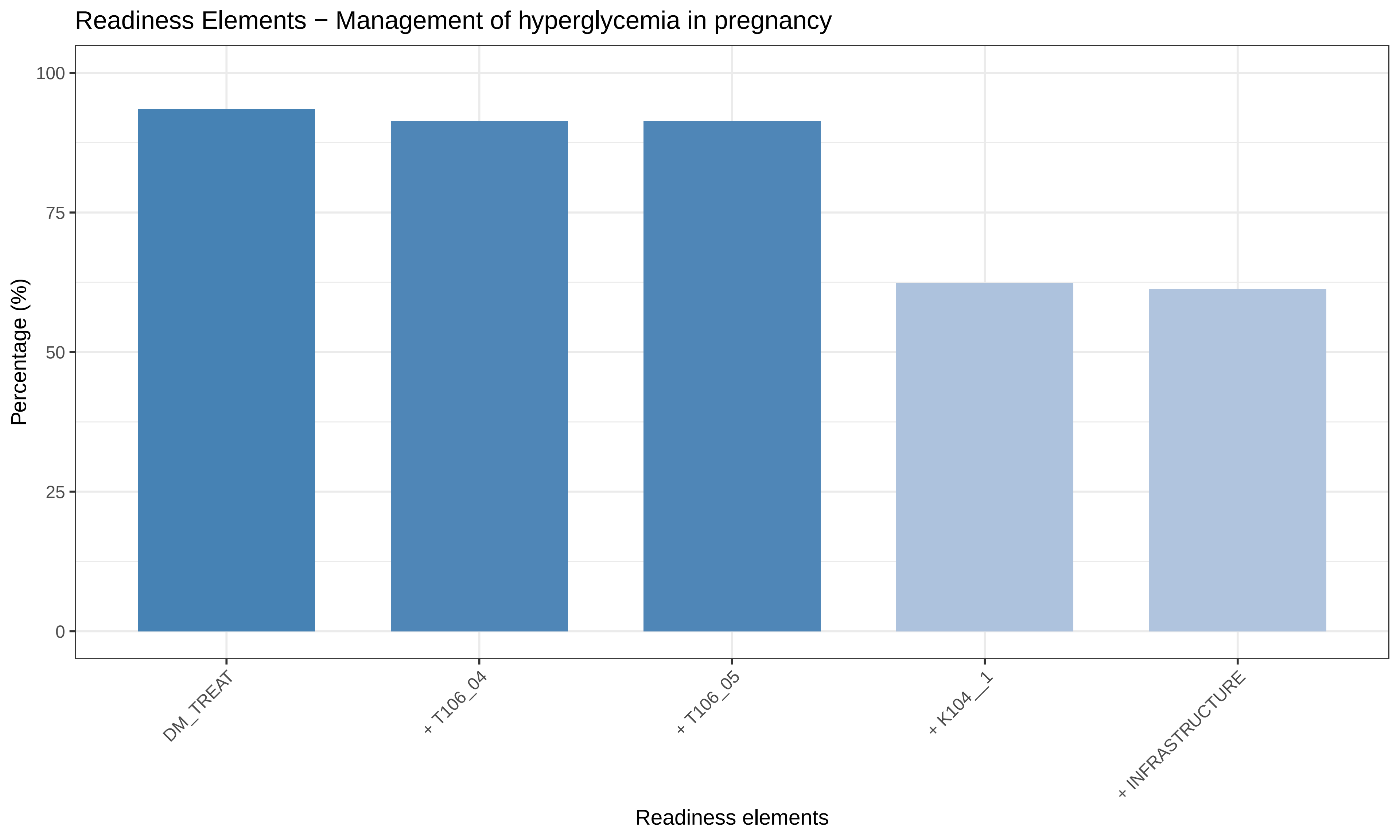
Readiness Elements − Management of hyperglycemia in pregnancy
Percentage (%)
100
75
50
25
0
DM_TREAT
+ T106_04
+ T106_05
+ K104__1
+ INFRASTRUCTURE
Readiness elements

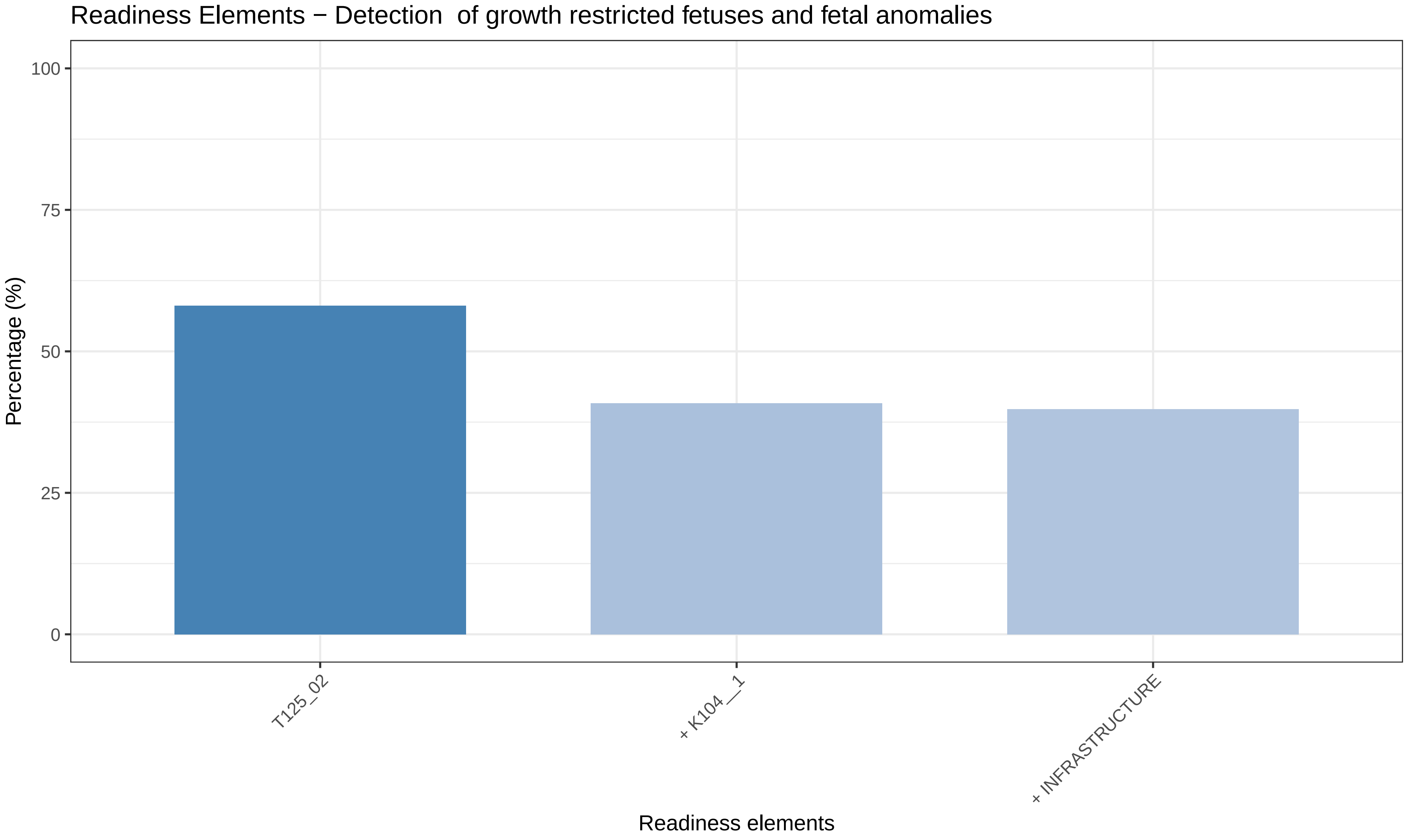
Readiness Elements − Detection of growth restricted fetuses and fetal anomalies
Percentage (%)
100
75
50
25
0
T125_02
+ K104__1
+ INFRASTRUCTURE
Readiness elements

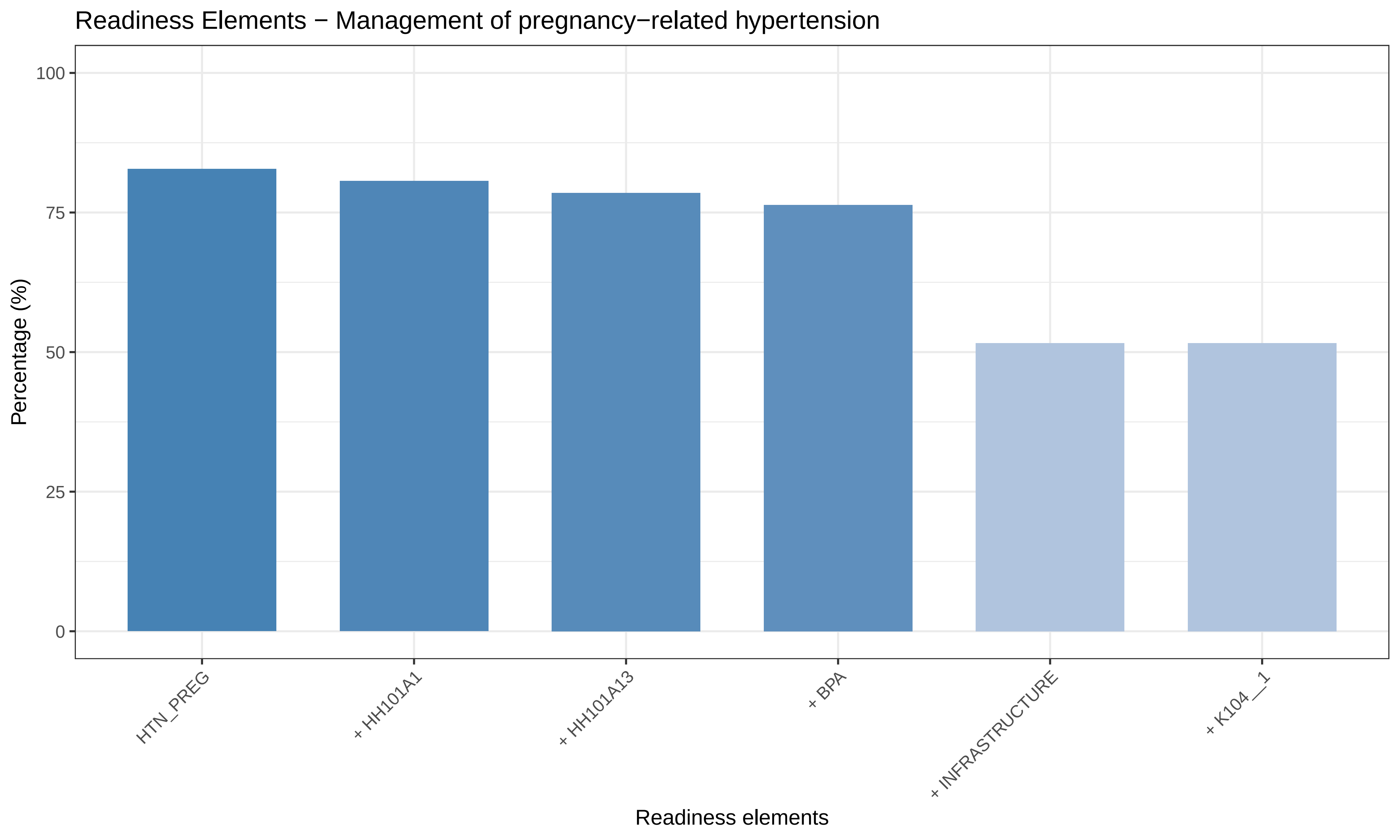
Readiness Elements − Management of pregnancy−related hypertension
Percentage (%)
100
75
50
25
0
HTN_PREG
+ HH101A1
+ HH101A13
+ BPA
+ INFRASTRUCTURE
+ K104__1
Readiness elements

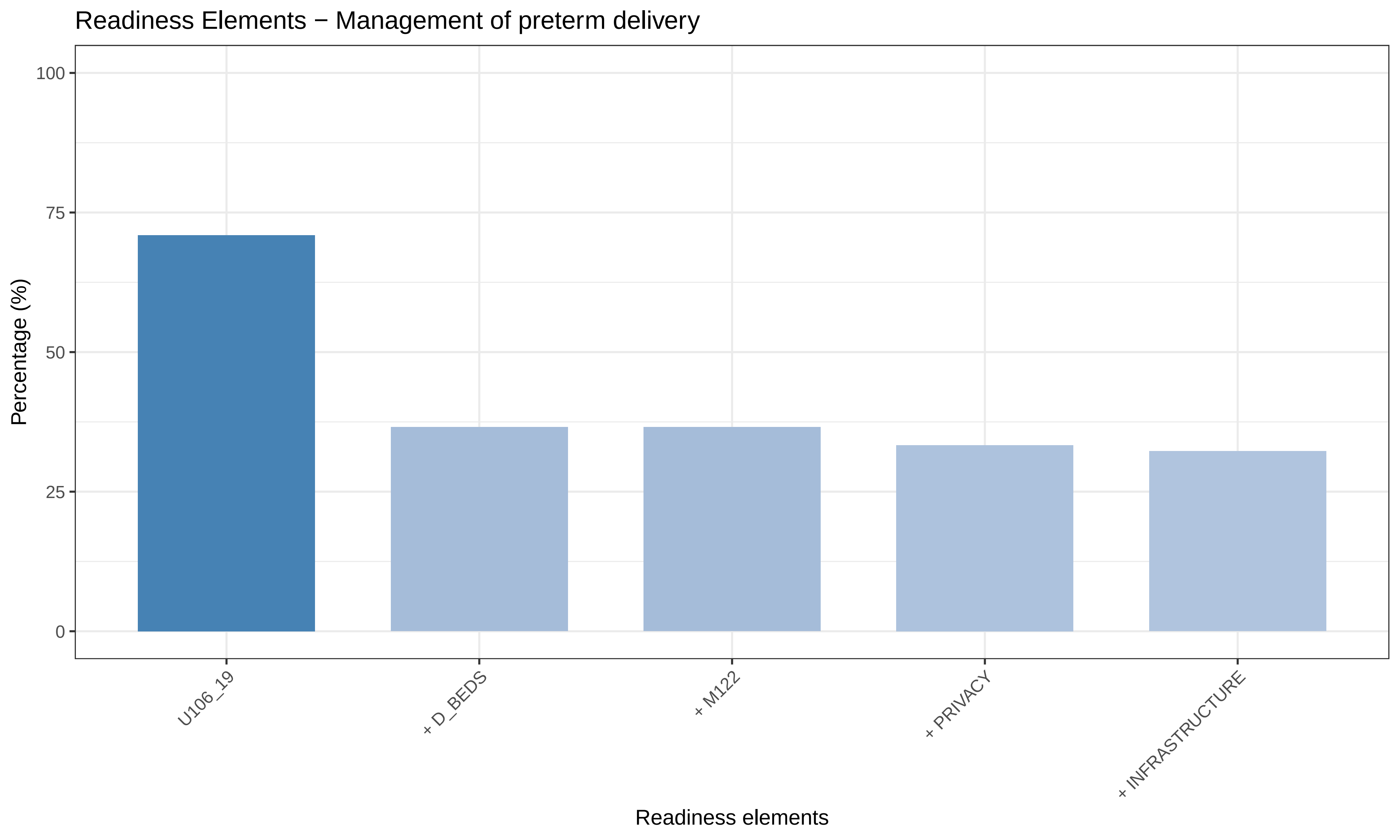
Readiness Elements − Management of preterm delivery
Percentage (%)
100
75
50
25
0
U106_19
+ D_BEDS
+ M122
+ PRIVACY
+ INFRASTRUCTURE
Readiness elements

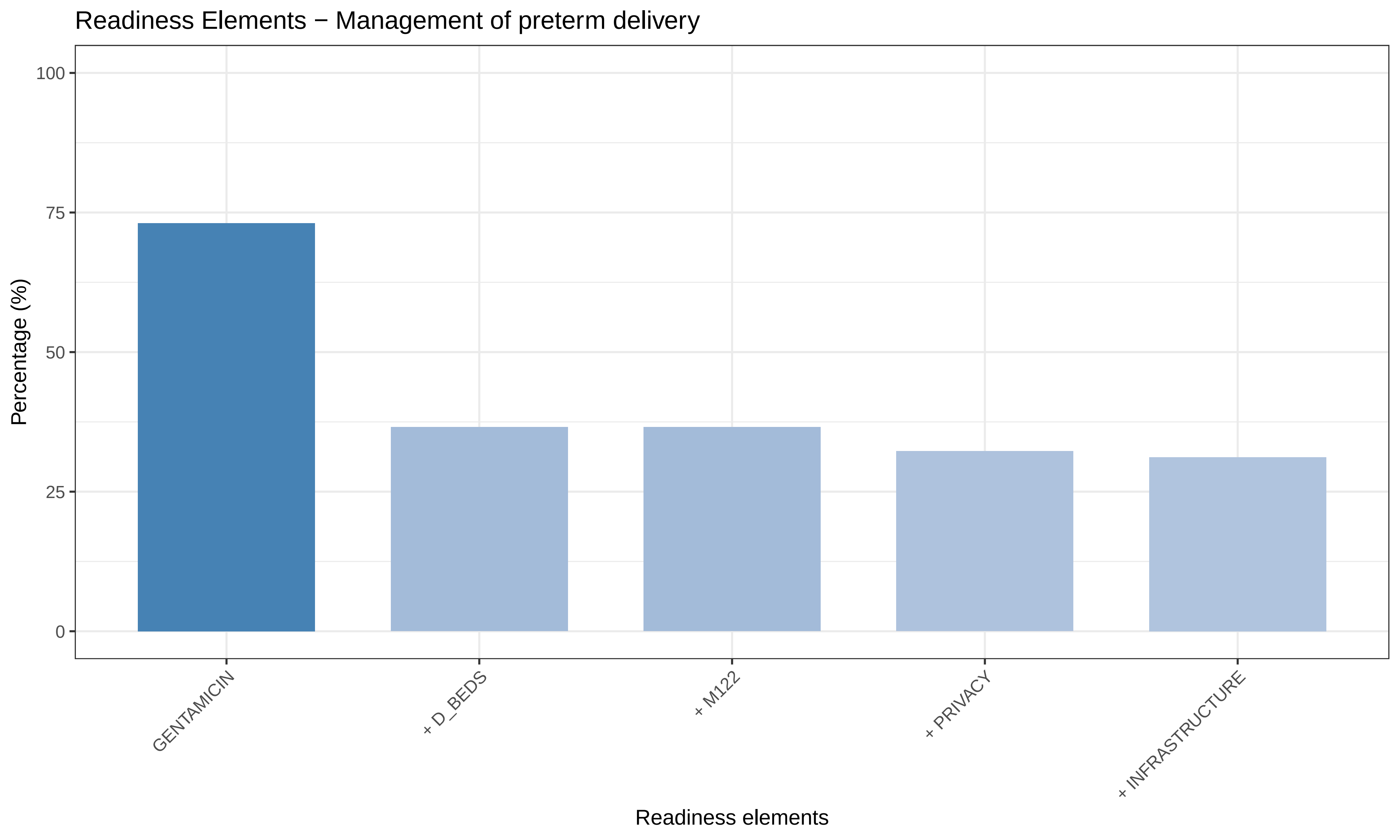
Readiness Elements − Management of preterm delivery
Percentage (%)
100
75
50
25
0
GENTAMICIN
+ D_BEDS
+ M122
+ PRIVACY
+ INFRASTRUCTURE
Readiness elements

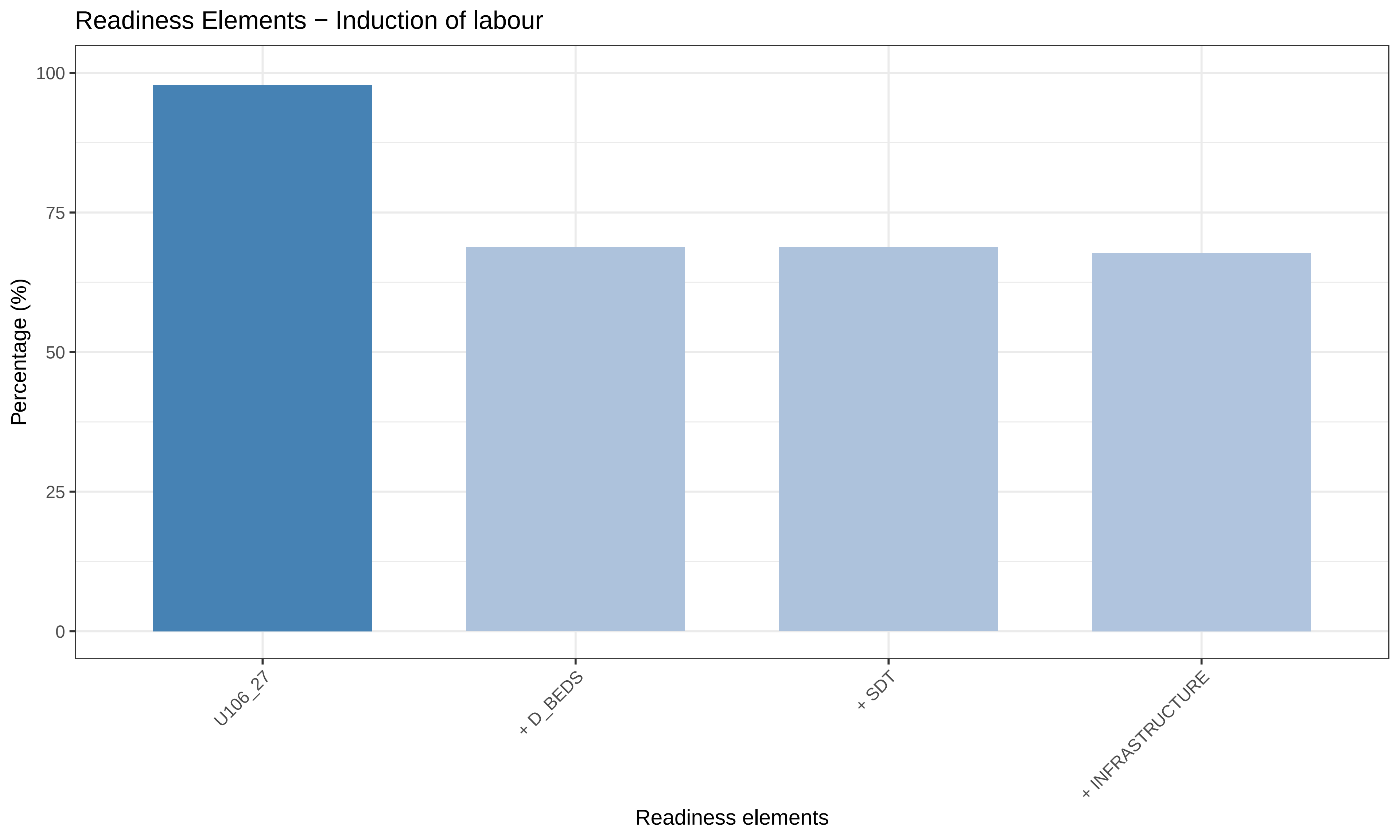
Readiness Elements − Induction of labour
Percentage (%)
100
75
50
25
0
U106_27
+ D_BEDS
+ SDT
+ INFRASTRUCTURE
Readiness elements

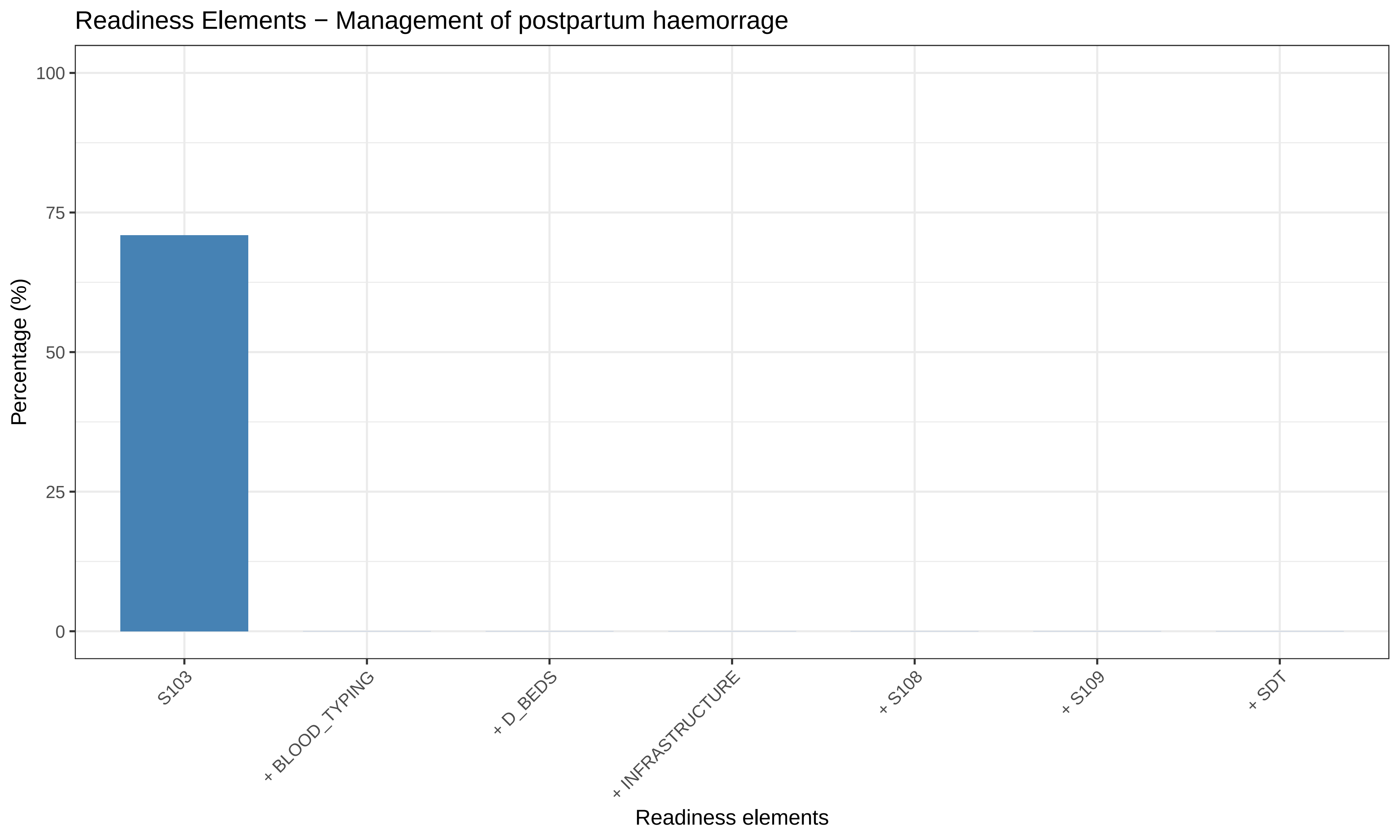

Readiness Elements − Management of postpartum haemorrhage
Percentage (%)
100
75
50
25
0
S103
+ BLOOD_TYPING
+ D_BEDS
+ INFRASTRUCTURE
+ S108
+ S109
+ SDT
Readiness elements

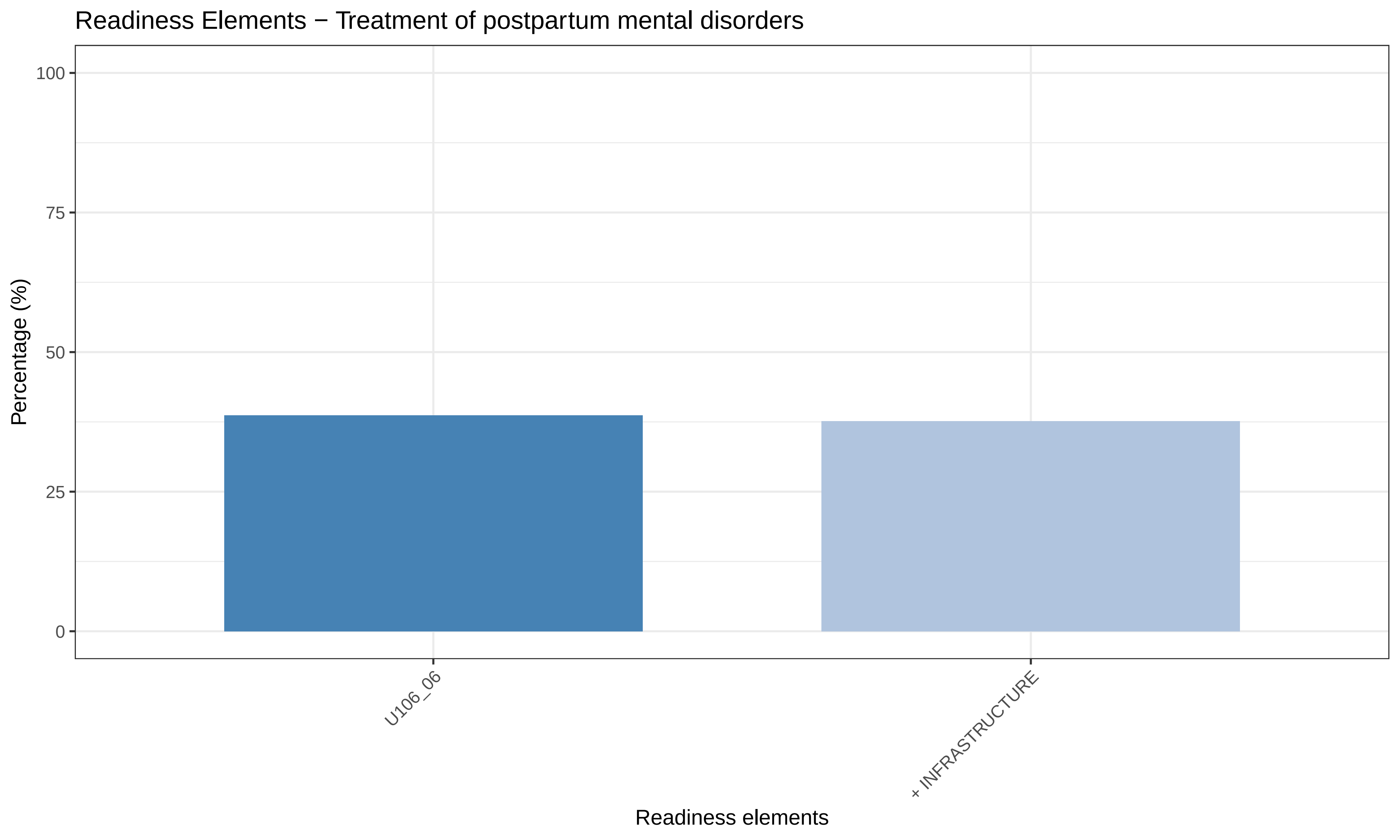
Readiness Elements − Treatment of postpartum mental disorders
Percentage (%)
100
75
50
25
0
U106_06
+ INFRASTRUCTURE
Readiness elements

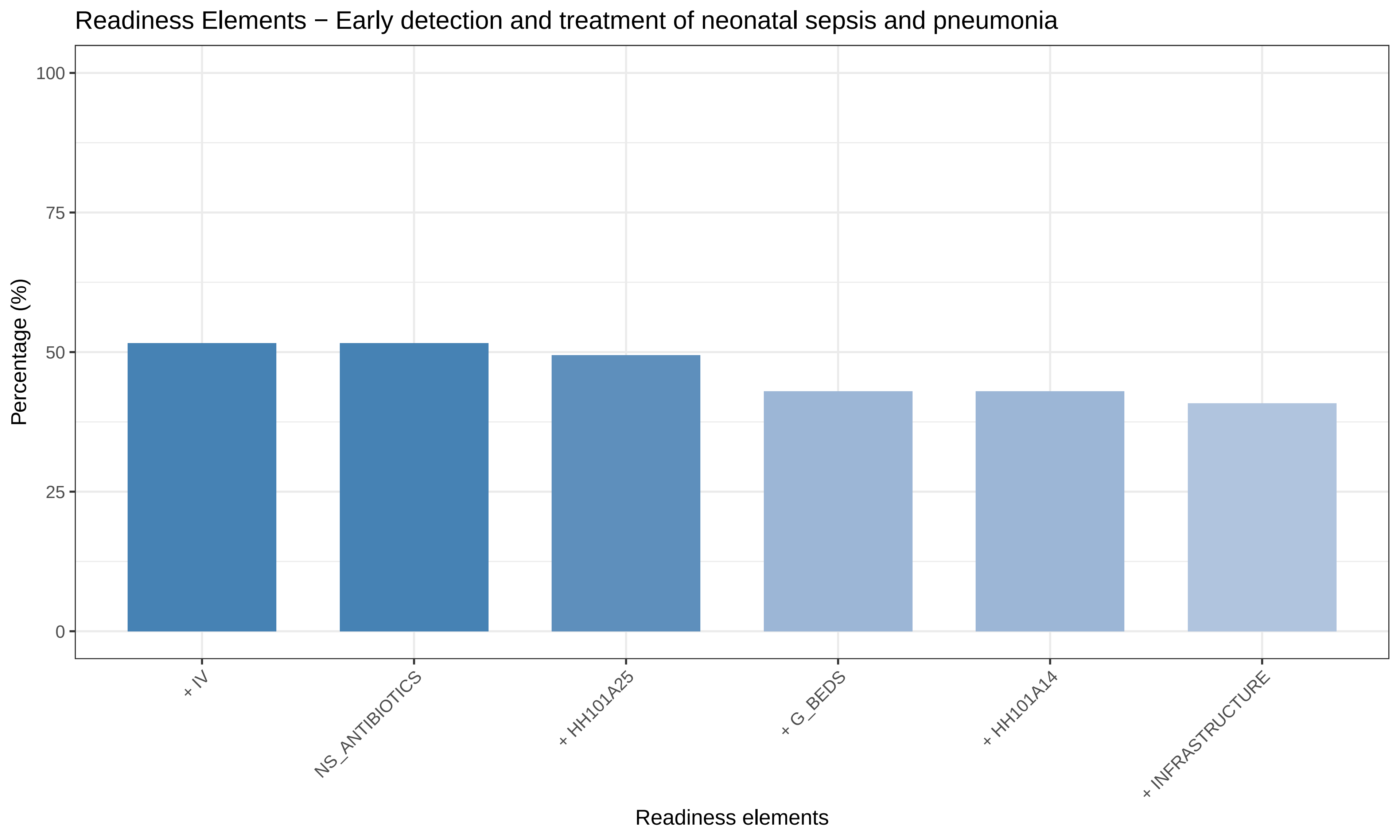
Readiness Elements − Early detection and treatment of neonatal sepsis and pneumonia
Percentage (%)
100
75
50
25
0
+ IV
NS_ANTIBIOTICS
+ HH101A25
+ G_BEDS
+ HH101A14
+ INFRASTRUCTURE
Readiness elements

Readiness Cascades_DH++

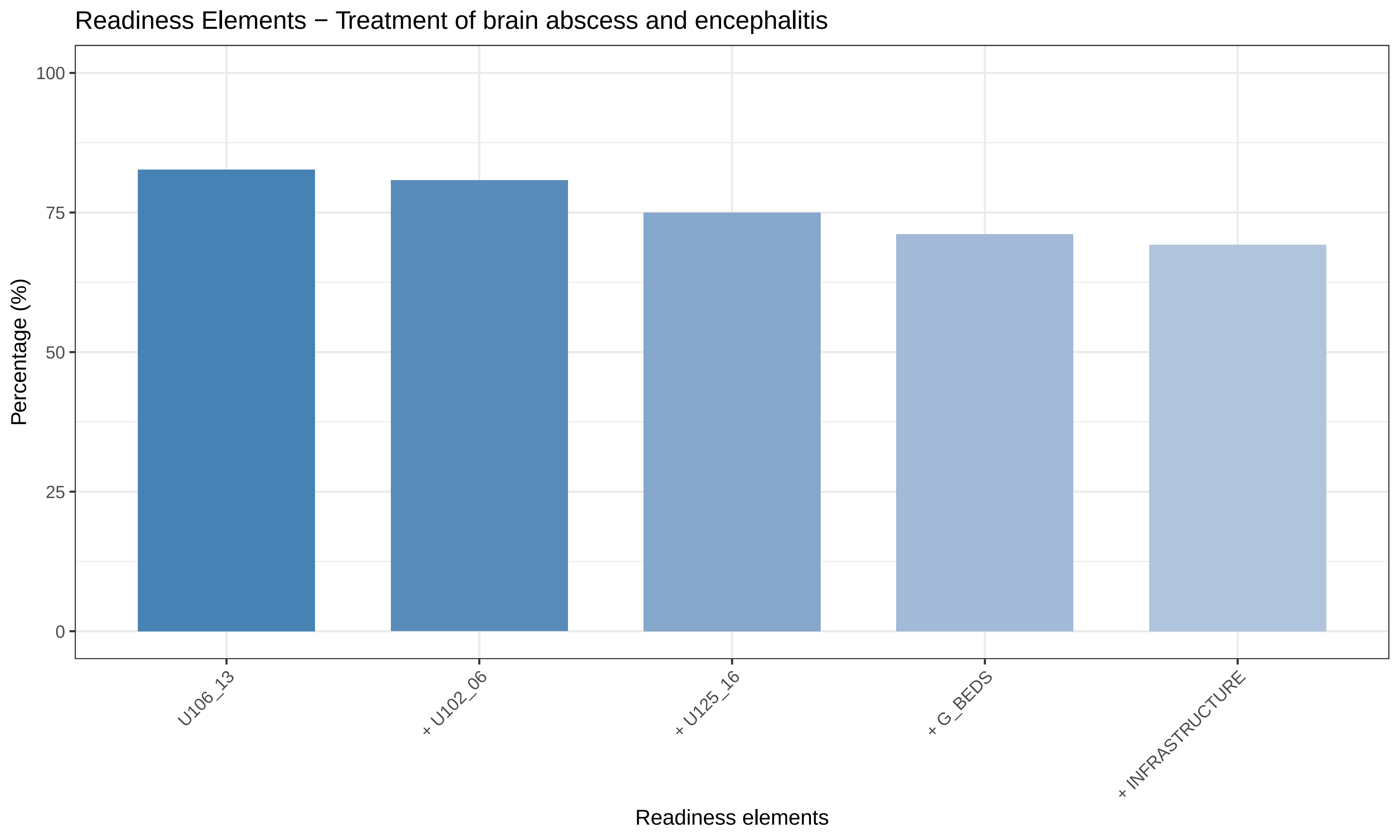
Readiness Elements − Treatment of brain abscess and encephalitis
Percentage (%)
100
75
50
25
0
U106_13
+ U102_06
+ U125_16
+ G_BEDS
+ INFRASTRUCTURE
Readiness elements

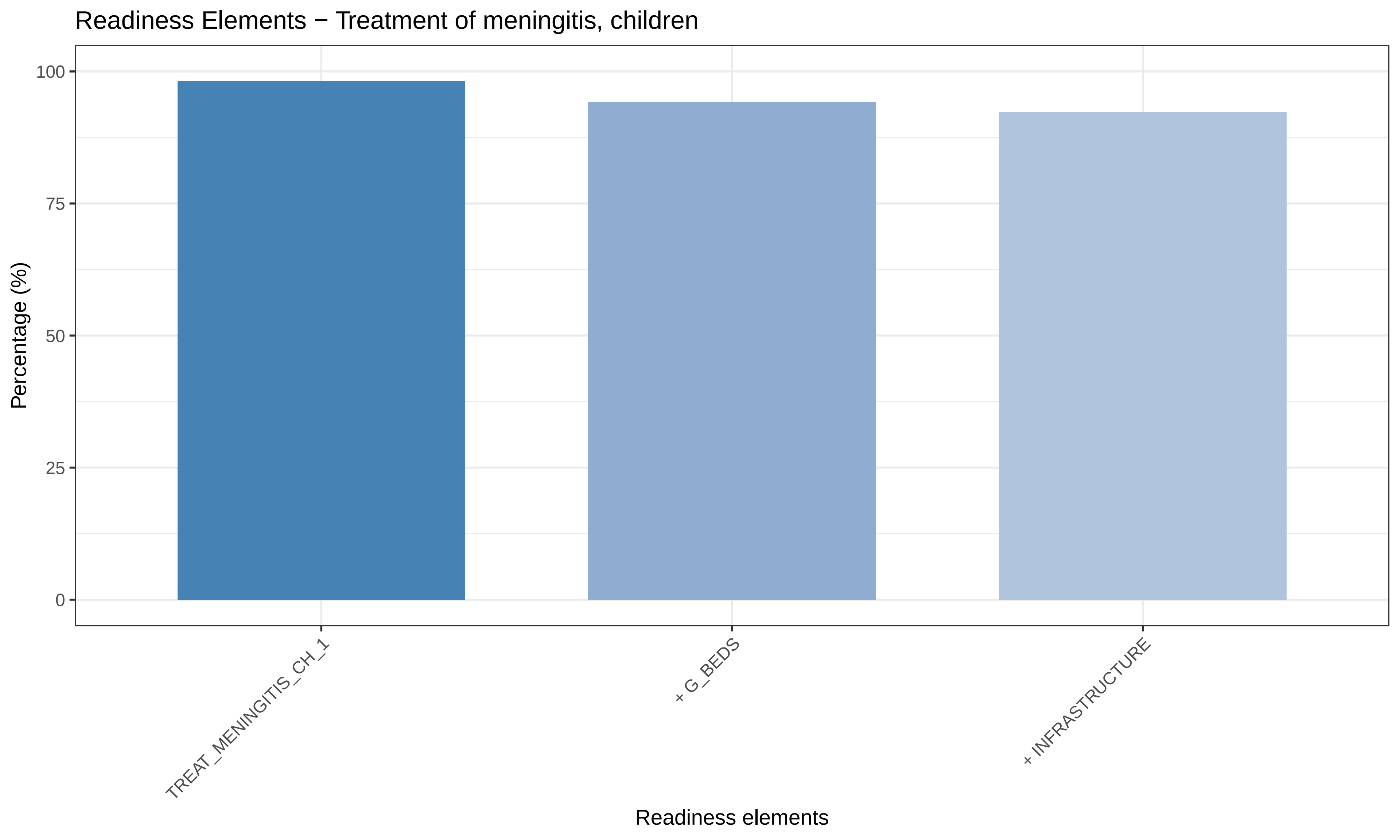
Readiness Elements − Treatment of meningitis, children
Percentage (%)
100
75
50
25
0
TREAT_MENINGITIS_CH_1
+ G_BEDS
+ INFRASTRUCTURE
Readiness elements

Readiness Elements − Treatment of meningitis, adults

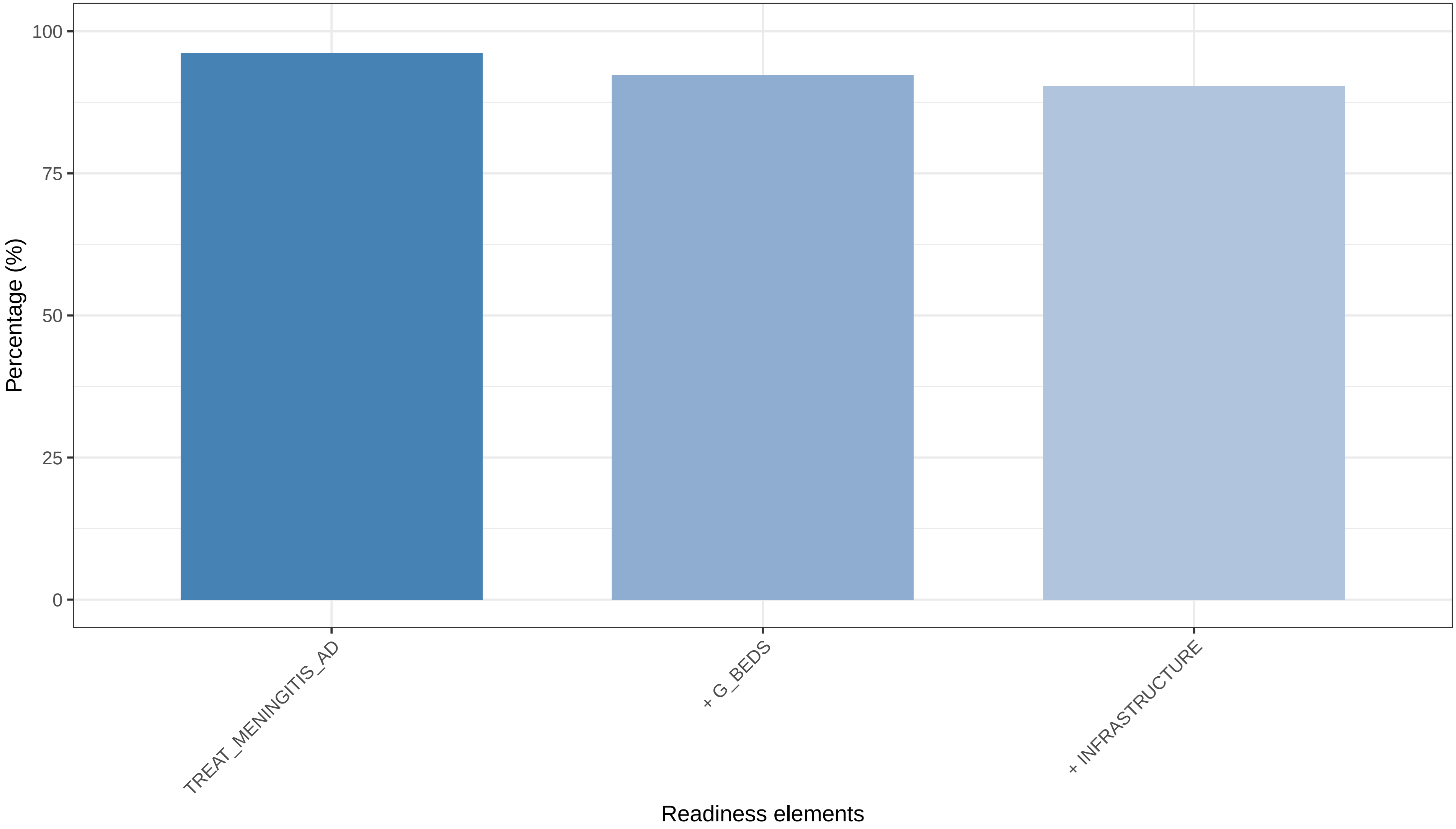

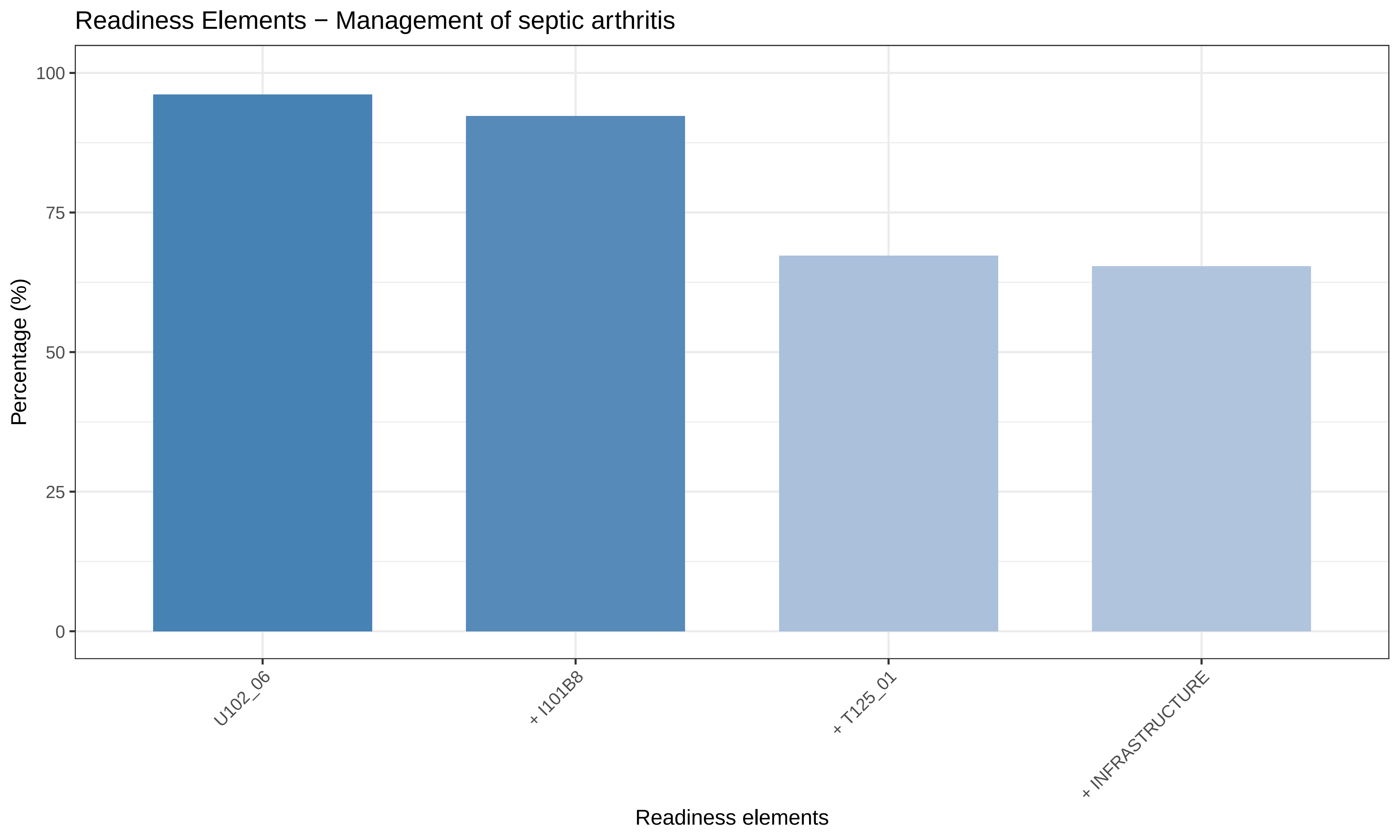
Readiness Elements − Management of septic arthritis
Percentage (%)
100
75
50
25
0
U102_06
+ I101B8
+ T125_01
+ INFRASTRUCTURE
Readiness elements

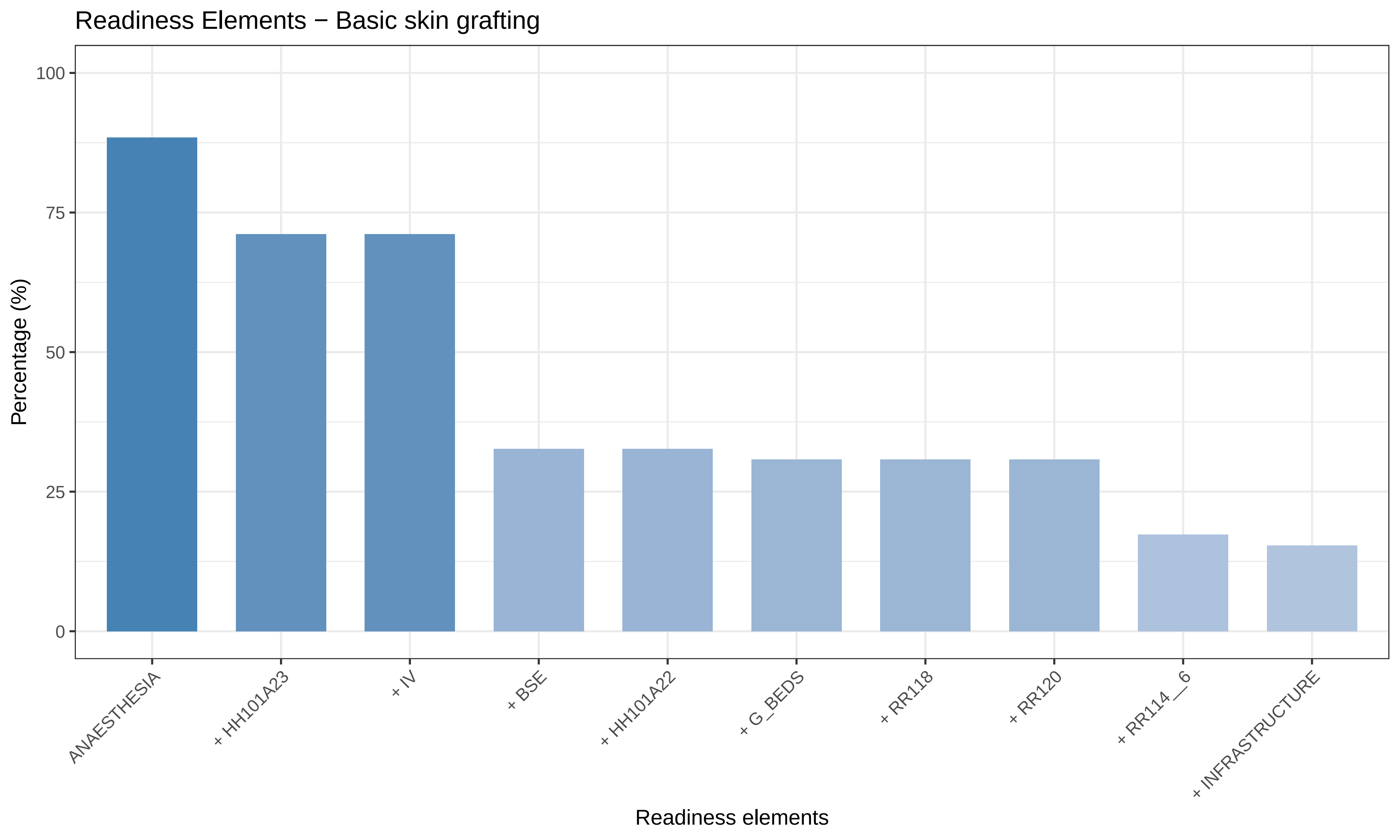
Readiness Elements − Basic skin grafting
Percentage (%)
100
75
50
25
0
ANAESTHESIA
+ HH101A23
+ IV
+ BSE
+ HH101A22
+ G_BEDS
+ RR118
+ RR120
+ RR114__6
+ INFRASTRUCTURE
Readiness elements

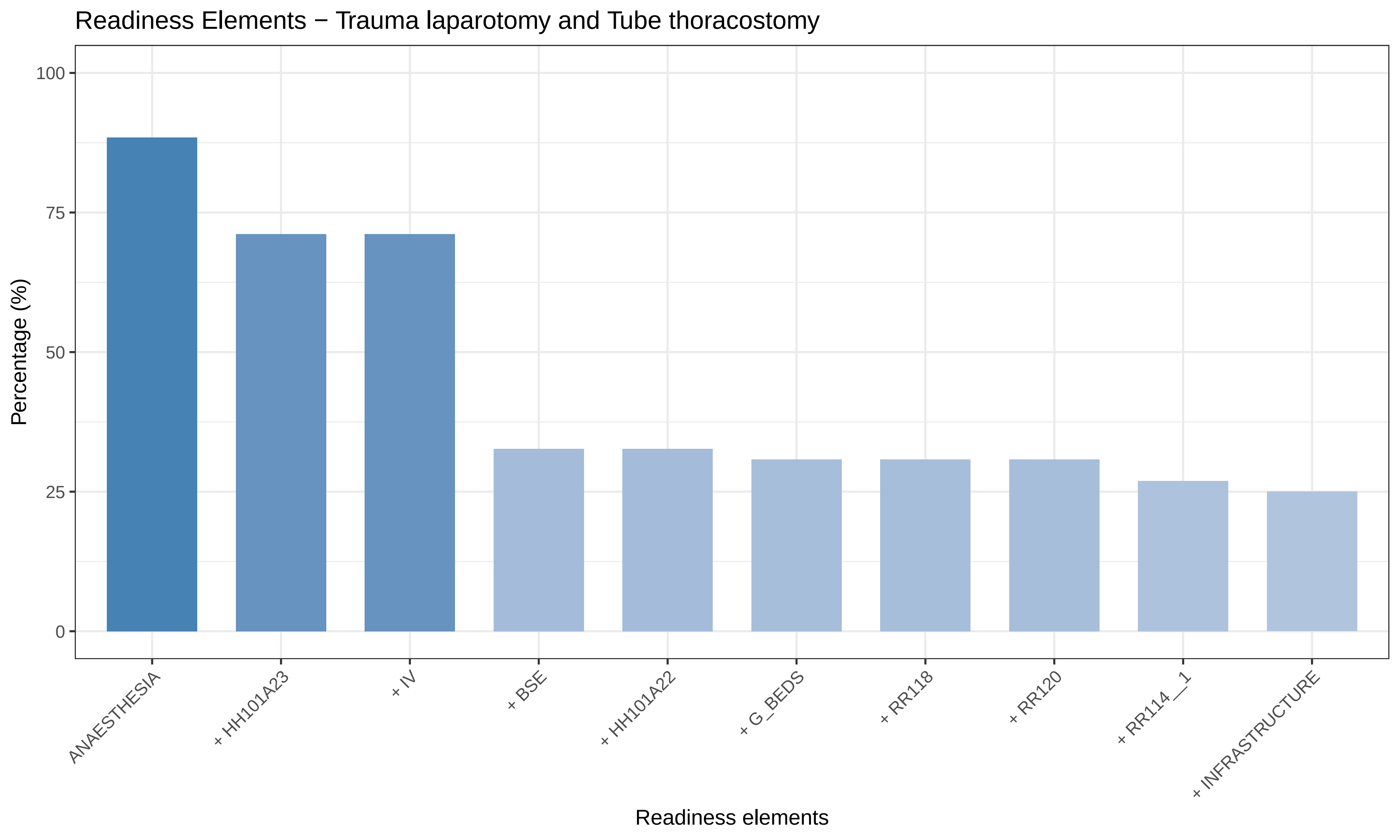
Readiness Elements − Trauma laparotomy and Tube thoracostomy
Percentage (%)
100
75
50
25
0
ANAESTHESIA
+ HH101A23
+ IV
+ BSE
+ HH101A22
+ G_BEDS
+ RR118
+ RR120
+ RR114__1
+ INFRASTRUCTURE
Readiness elements

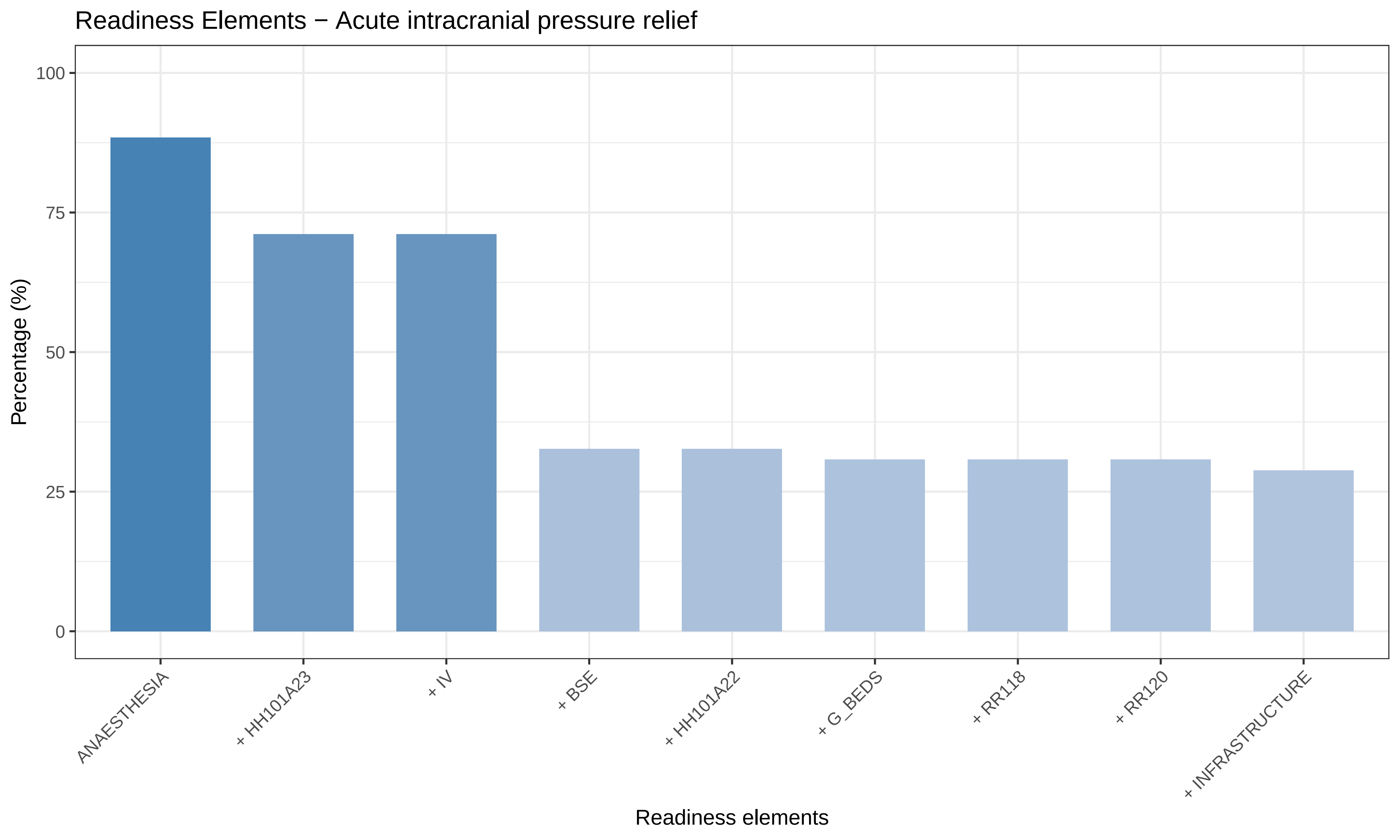

Readiness Elements − Acute intracranial pressure relief
Percentage (%)
100
75
50
25
0
ANAESTHESIA
+ HH101A23
+ IV
+ BSE
+ HH101A22
+ G_BEDS
+ RR118
+ RR120
+ INFRASTRUCTURE
Readiness elements

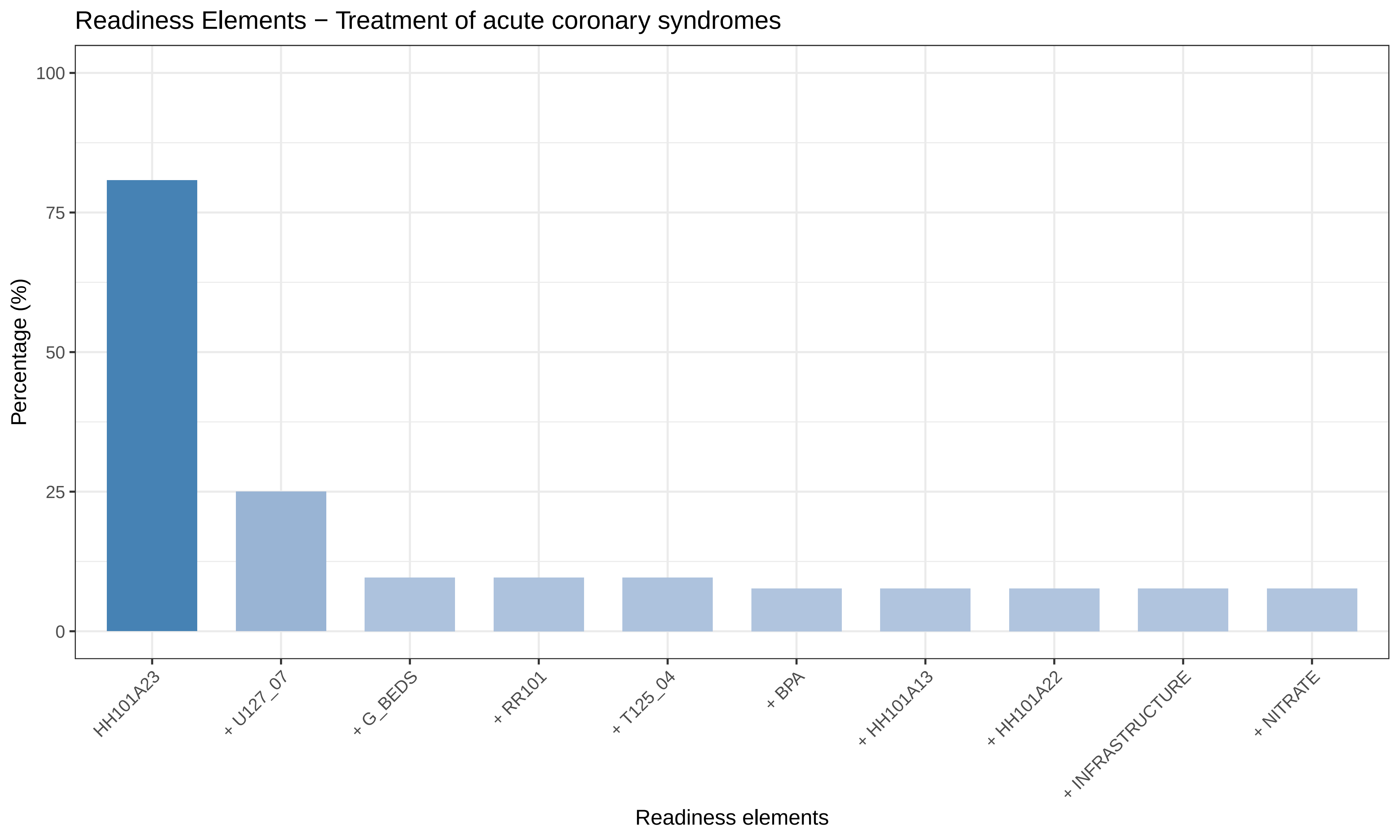
Readiness Elements − Treatment of acute coronary syndromes
Percentage (%)
100
75
50
25
0
HH101A23
+ U127_07
+ G_BEDS
+ RR101
+ T125_04
+ BPA
+ HH101A13
+ HH101A22
+ INFRASTRUCTURE
+ NITRATE
Readiness elements

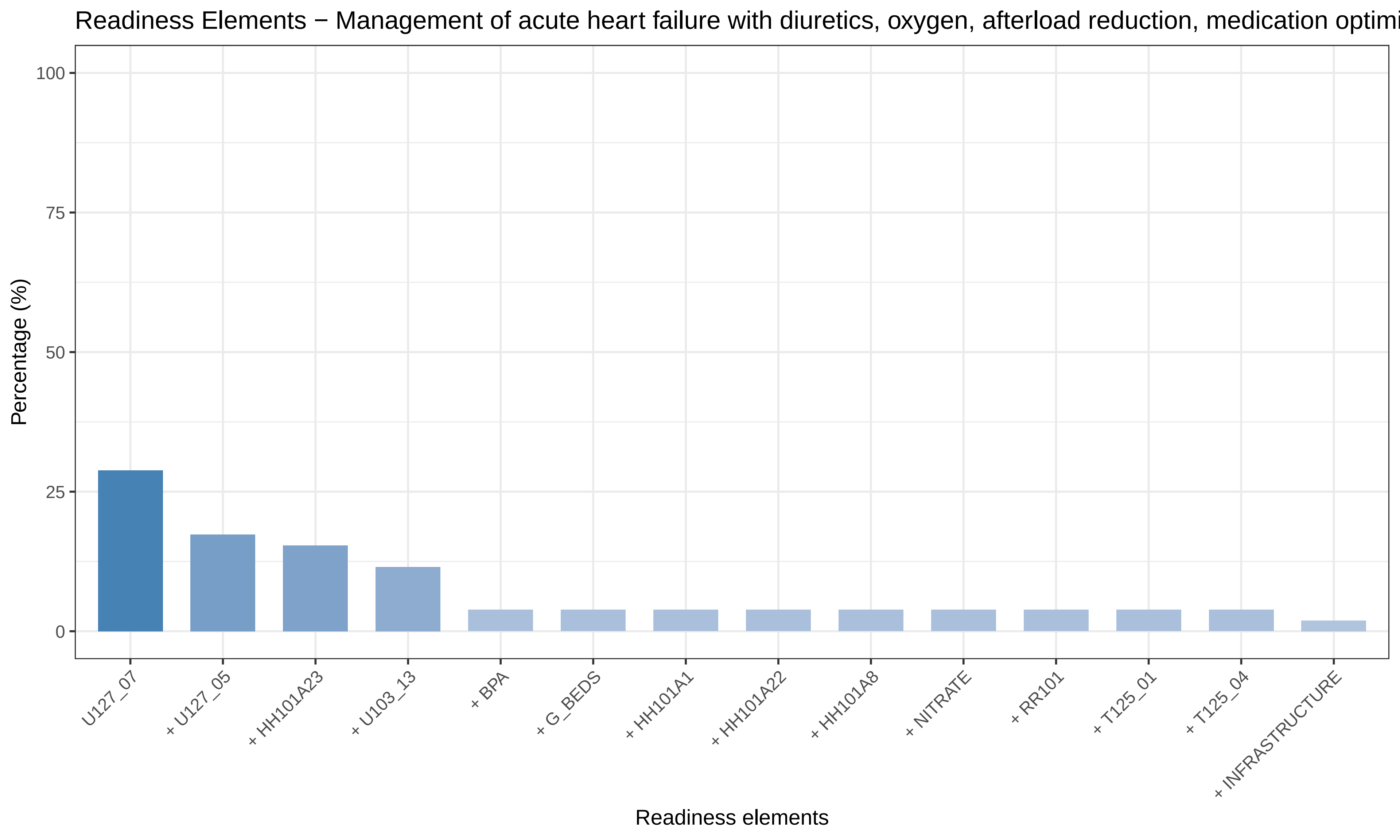
Readiness Elements − Management of acute heart failure with diuretics, oxygen, afterload reduction, medication optimi
100
75
50
25
0
Percentage (%)
U127_07
+ U127_05
+ HH101A23
+ U103_13
+ BPA
+ G_BEDS
+ HH101A1
+ HH101A22
+ HH101A8
+ NITRATE
+ RR101
+ T125_01
+ T125_04
+ INFRASTRUCTURE
Readiness elements

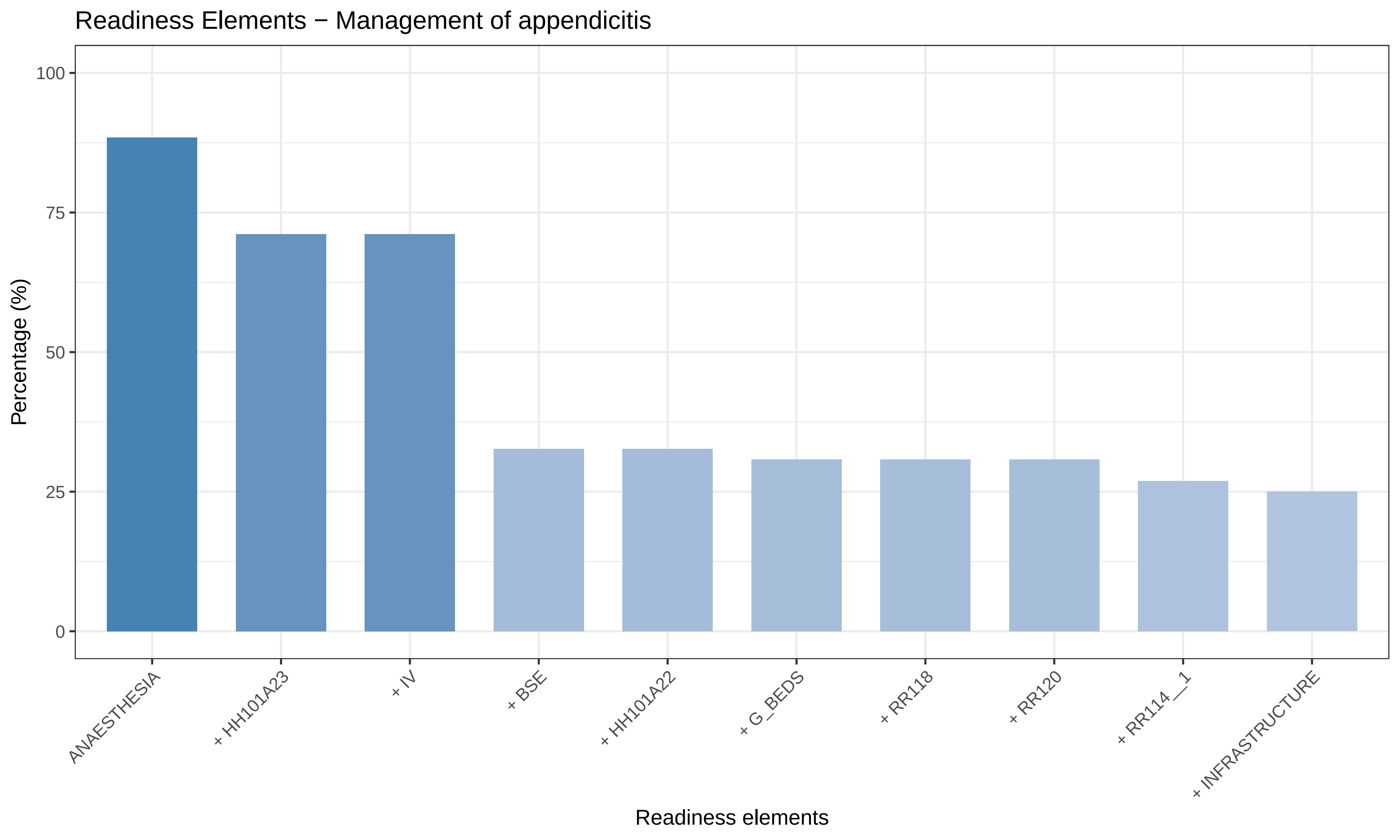
Readiness Elements − Management of appendicitis
Percentage (%)
100
75
50
25
0
ANAESTHESIA
+ HH101A23
+ IV
+ BSE
+ HH101A22
+ G_BEDS
+ RR118
+ RR120
+ RR114__1
+ INFRASTRUCTURE
Readiness elements

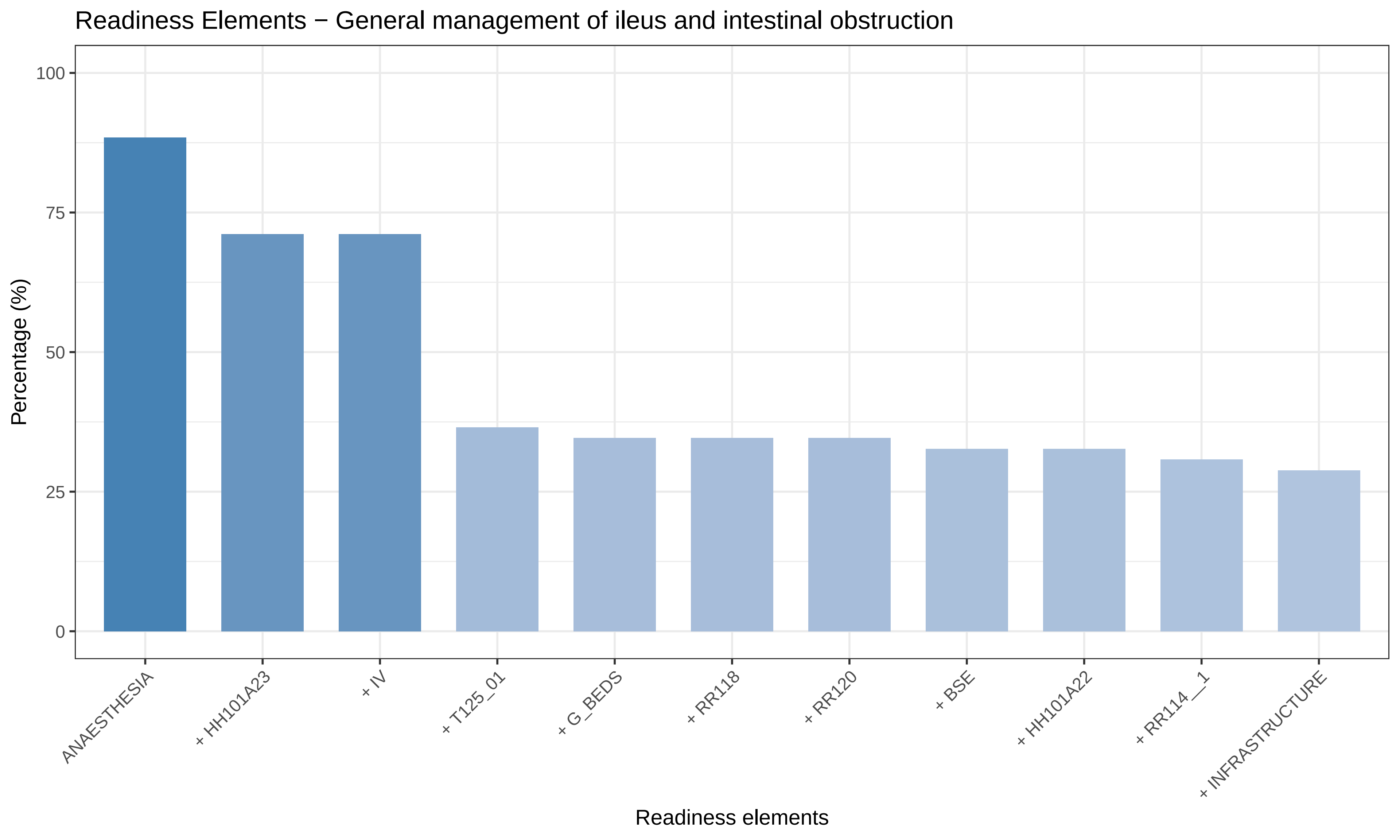
Readiness Elements − General management of ileus and intestinal obstruction
Percentage (%)
100
75
50
25
0
ANAESTHESIA
+ HH101A23
+ IV
+ T125_01
+ G_BEDS
+ RR118
+ RR120
+ BSE
+ HH101A22
+ RR114__1
+ INFRASTRUCTURE
Readiness elements

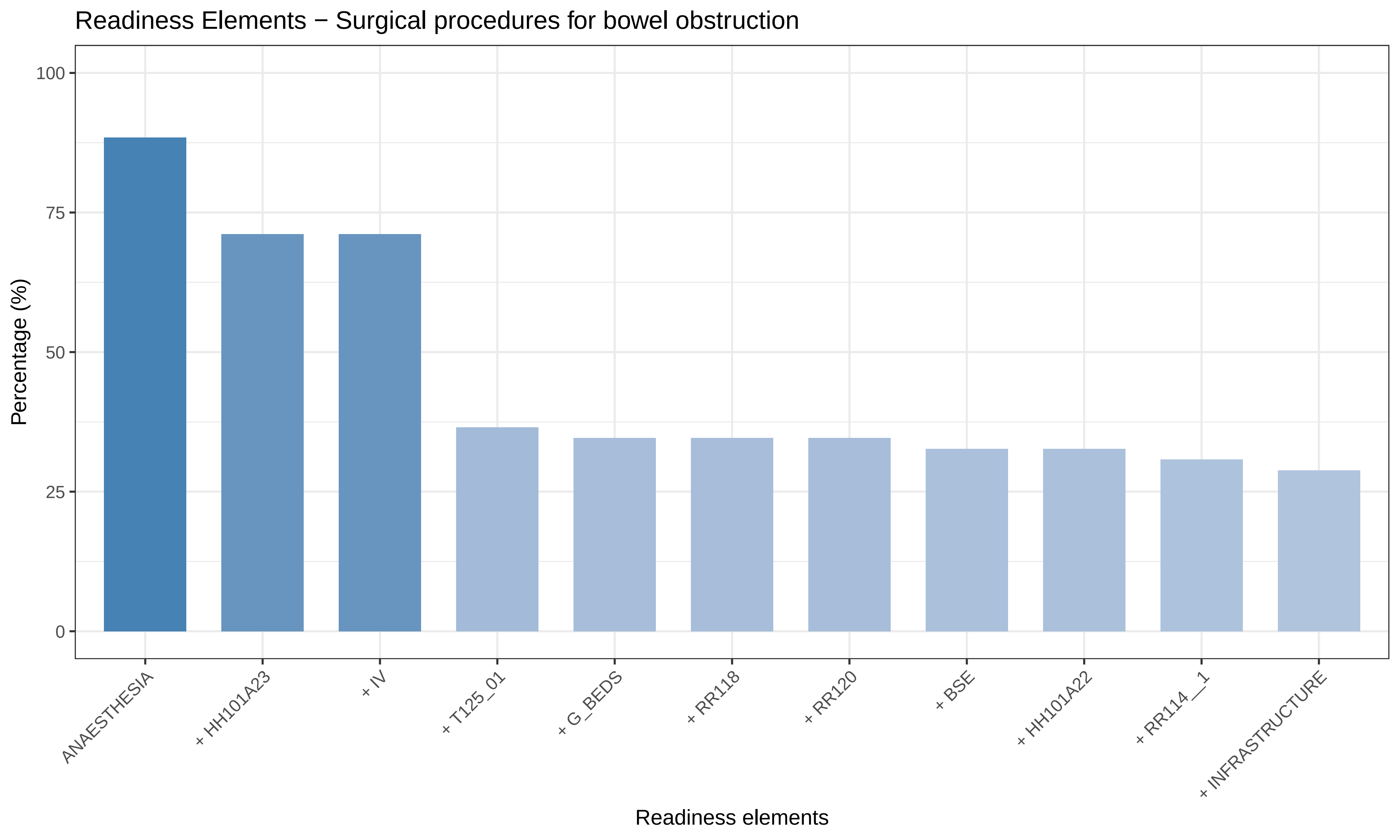

Readiness Elements − Surgical procedures for bowel obstruction
Percentage (%)
100
75
50
25
0
ANAESTHESIA
+ HH101A23
+ IV
+ T125_01
+ G_BEDS
+ RR118
+ RR120
+ BSE
+ HH101A22
+ RR114__1
+ INFRASTRUCTURE
Readiness elements

Readiness Elements − Management of hernias (inguinal, femoral, abdominal)

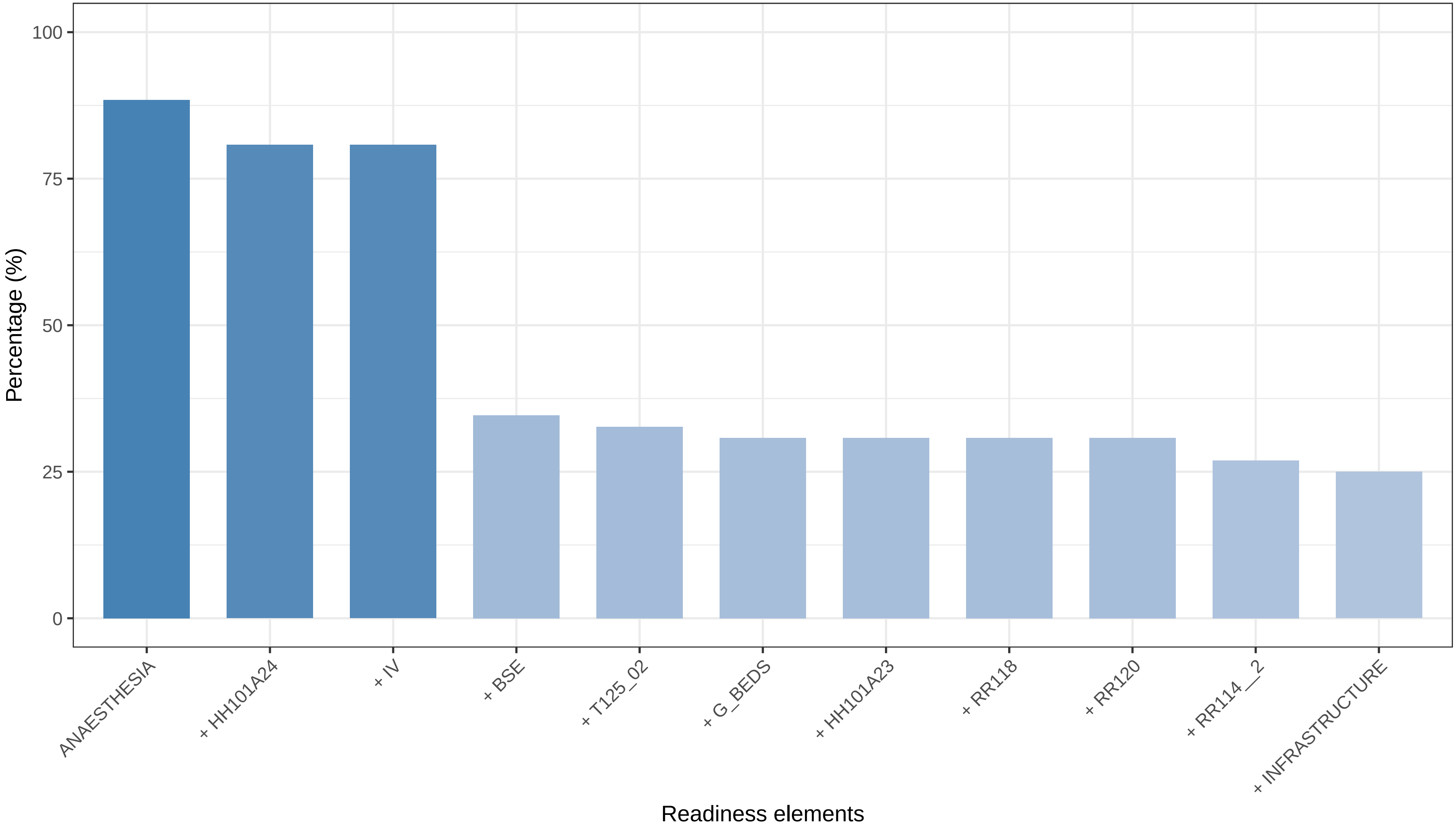

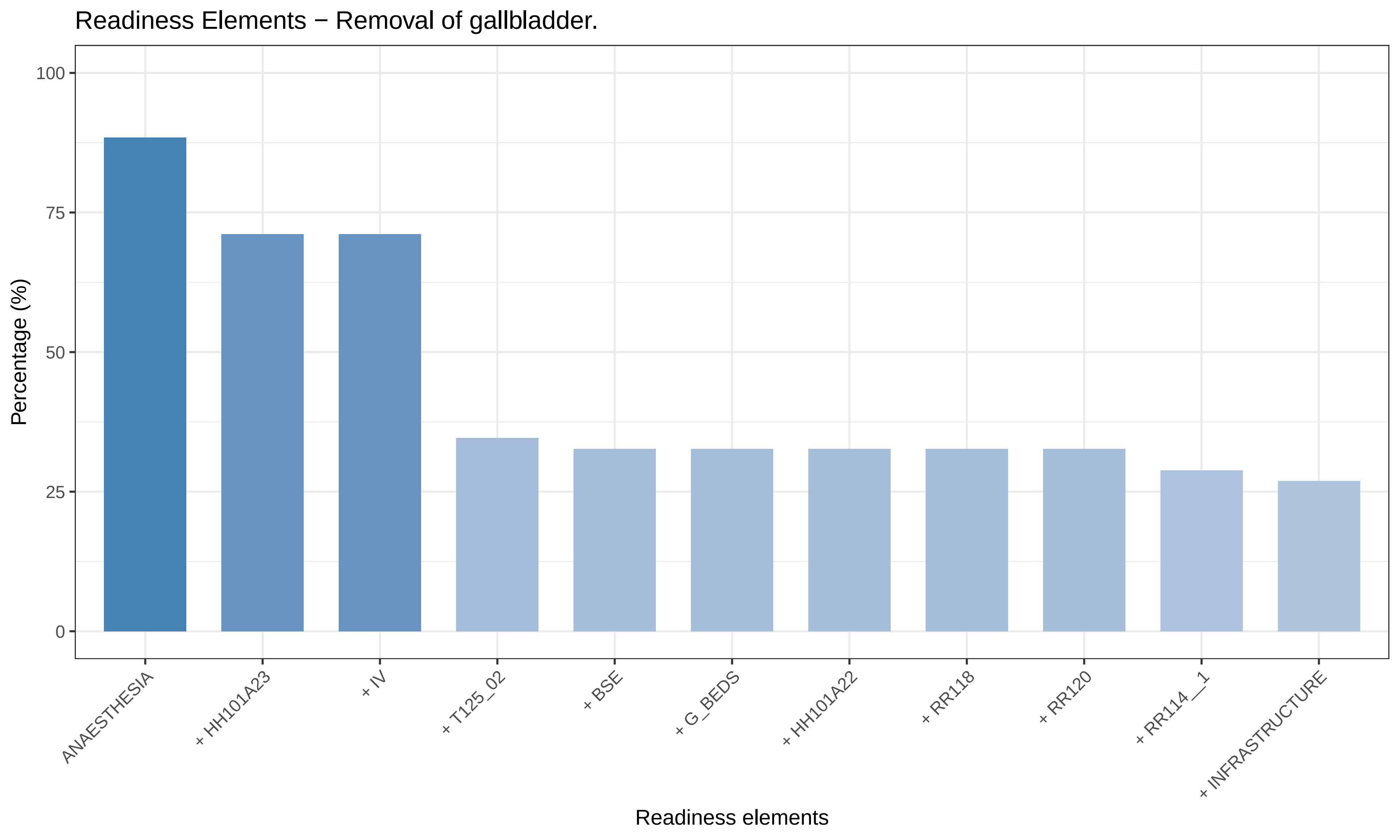

Readiness Elements − Removal of gallbladder.
Percentage (%)
100
75
50
25
0
ANAESTHESIA
+ HH101A23
+ IV
+ T125_02
+ BSE
+ G_BEDS
+ HH101A22
+ RR118
+ RR120
+ RR114__1
+ INFRASTRUCTURE
Readiness elements

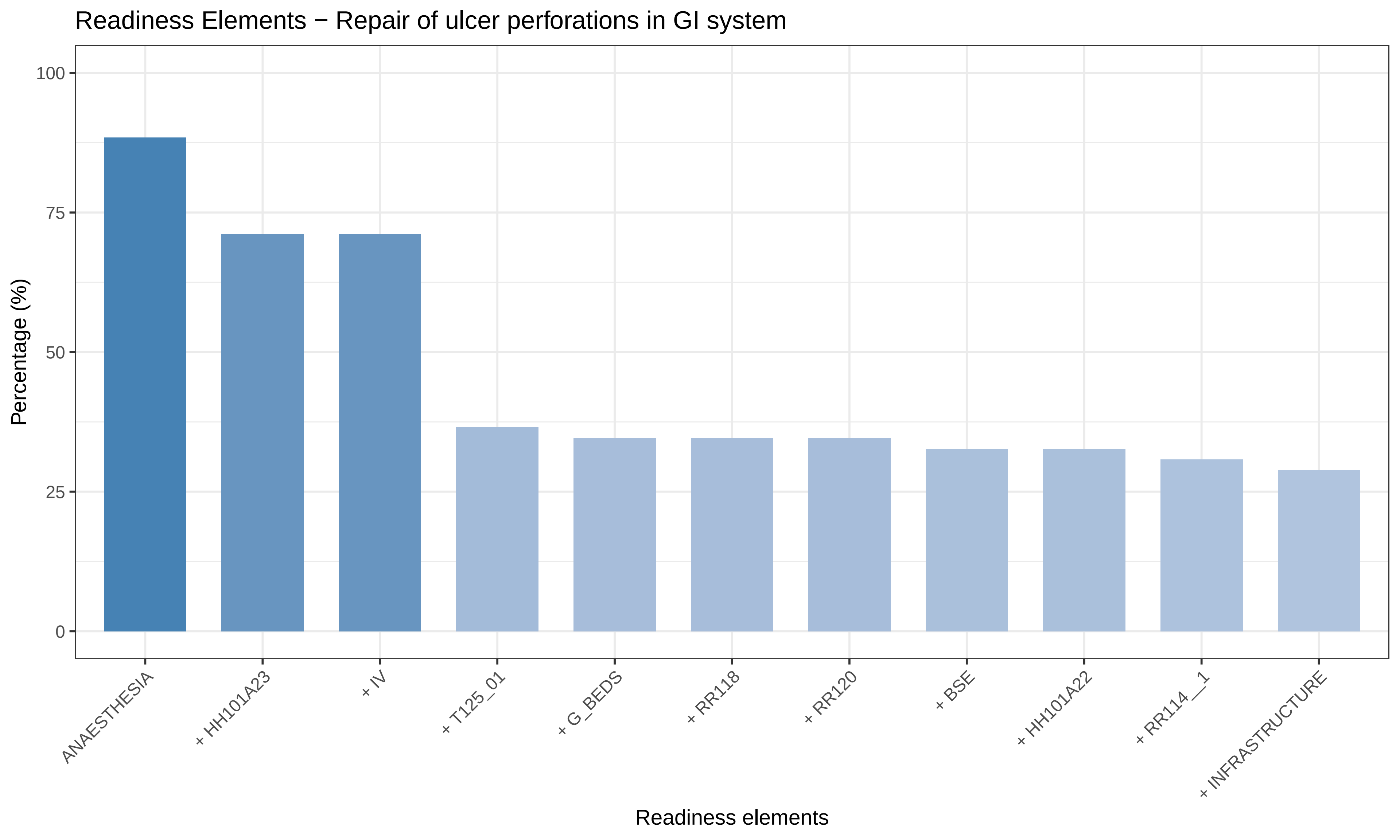
Readiness Elements − Repair of ulcer perforations in GI system
Percentage (%)
100
75
50
25
0
ANAESTHESIA
+ HH101A23
+ IV
+ T125_01
+ G_BEDS
+ RR118
+ RR120
+ BSE
+ HH101A22
+ RR114__1
+ INFRASTRUCTURE
Readiness elements

Readiness Elements − Repair of ileal perforation due to typhoid

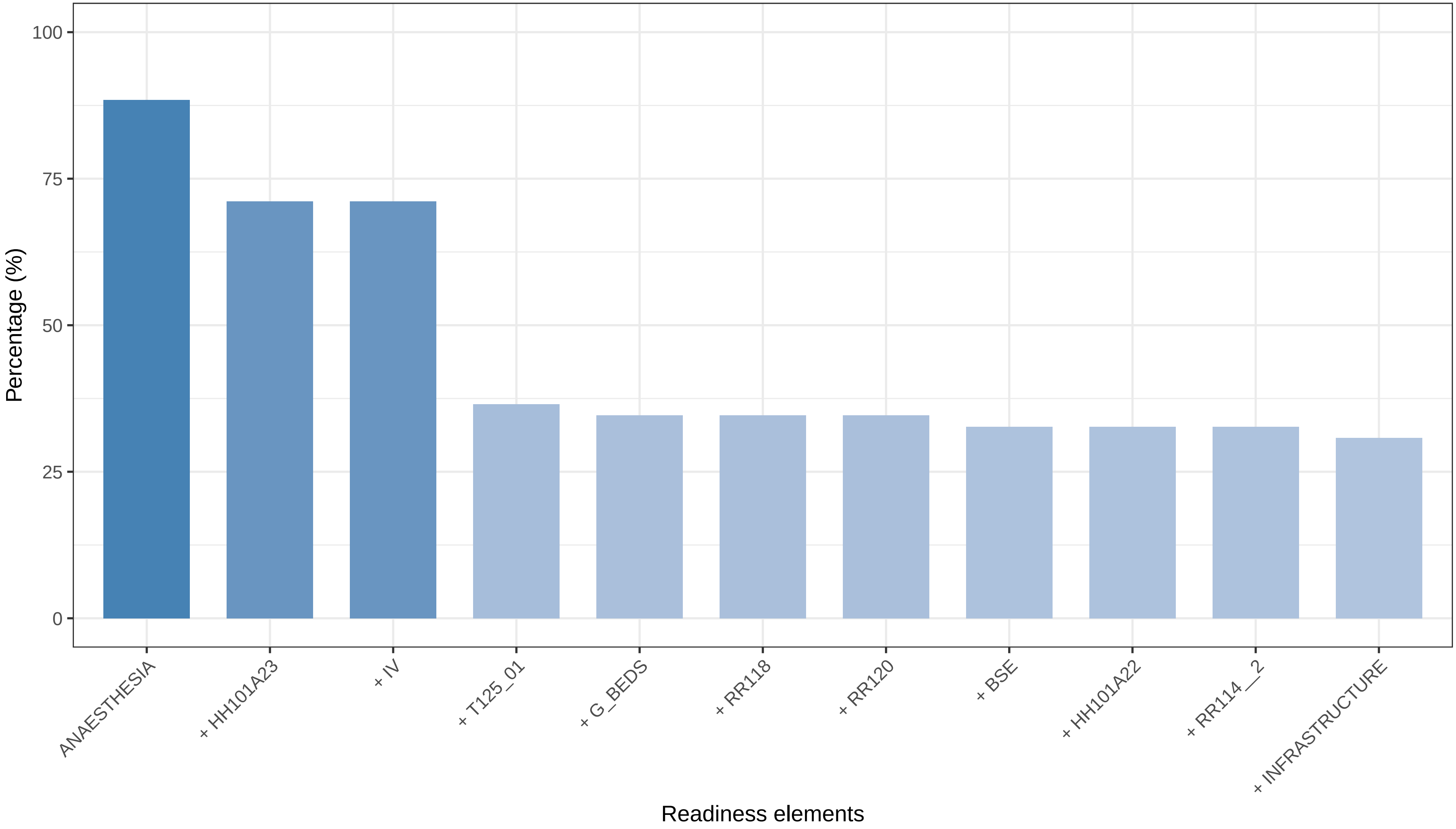

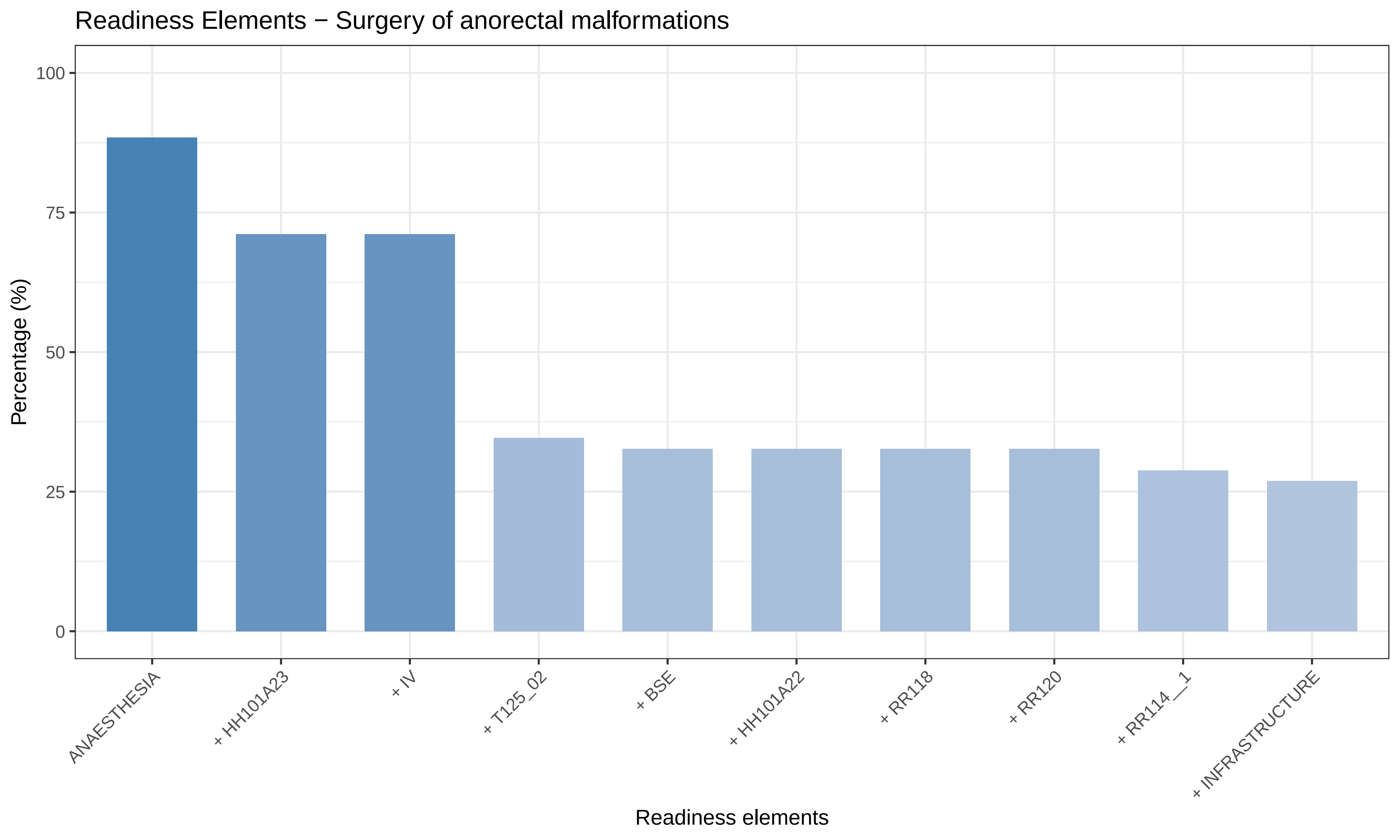
Readiness Elements − Surgery of anorectal malformations
Percentage (%)
100
75
50
25
0
ANAESTHESIA
+ HH101A23
+ IV
+ T125_02
+ BSE
+ HH101A22
+ RR118
+ RR120
+ RR114__1
+ INFRASTRUCTURE
Readiness elements

Readiness Elements − Surgical repair for congenital talipes

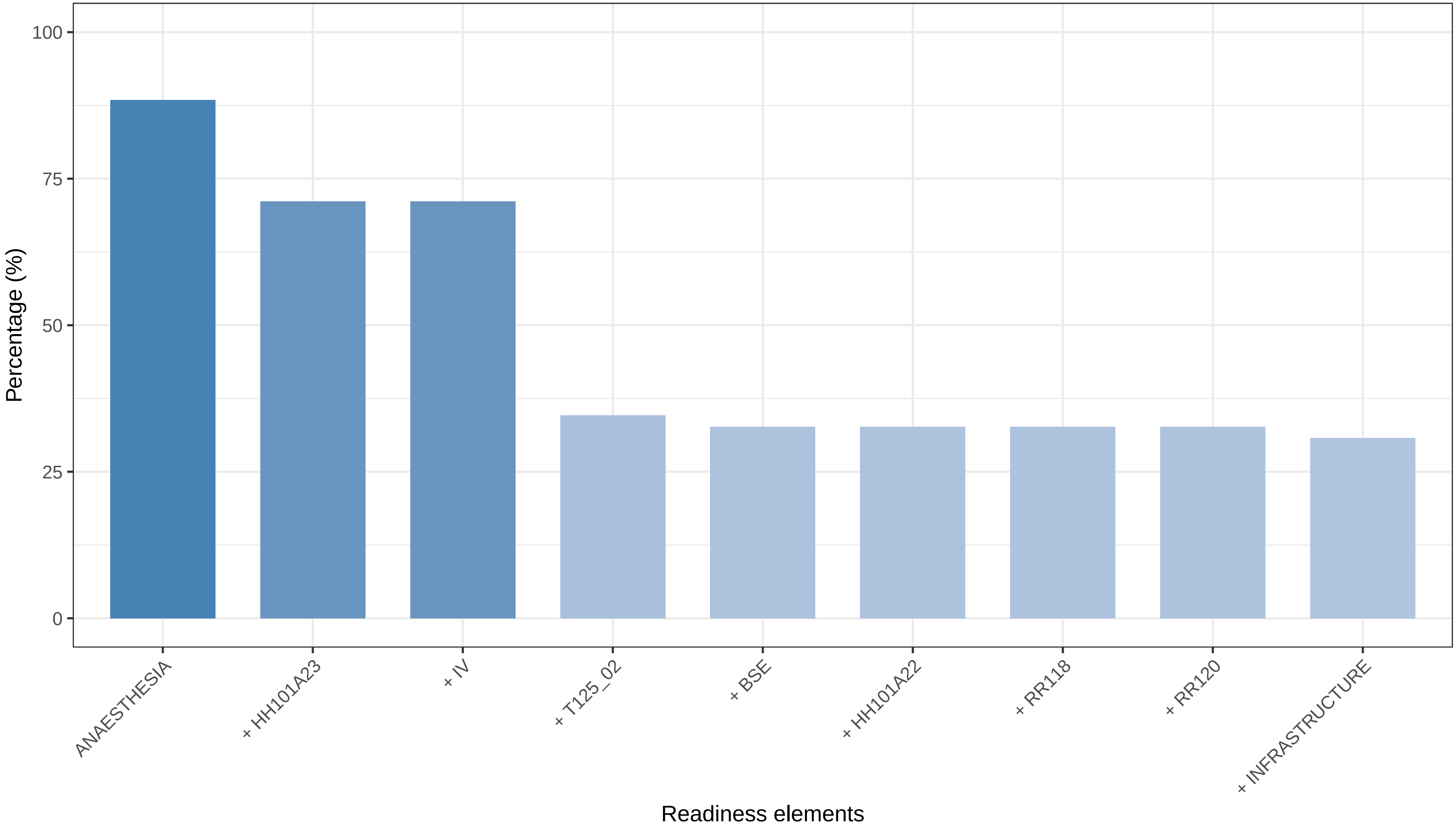

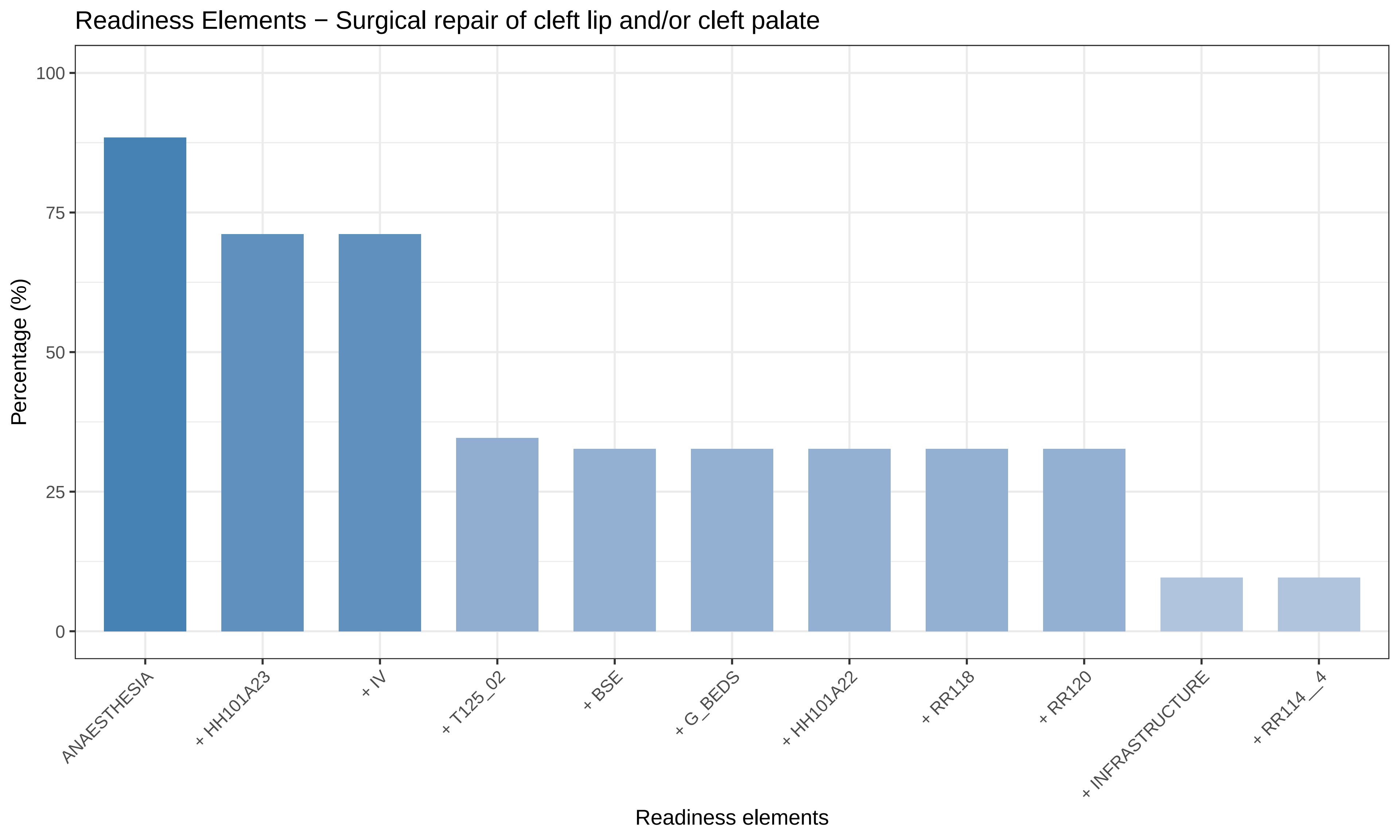

Readiness Elements − Surgical repair of cleft lip and/or cleft palate
Percentage (%)
100
75
50
25
0
ANAESTHESIA
+ HH101A23
+ IV
+ T125_02
+ BSE
+ G_BEDS
+ HH101A22
+ RR118
+ RR120
+ INFRASTRUCTURE
+ RR114__4
Readiness elements

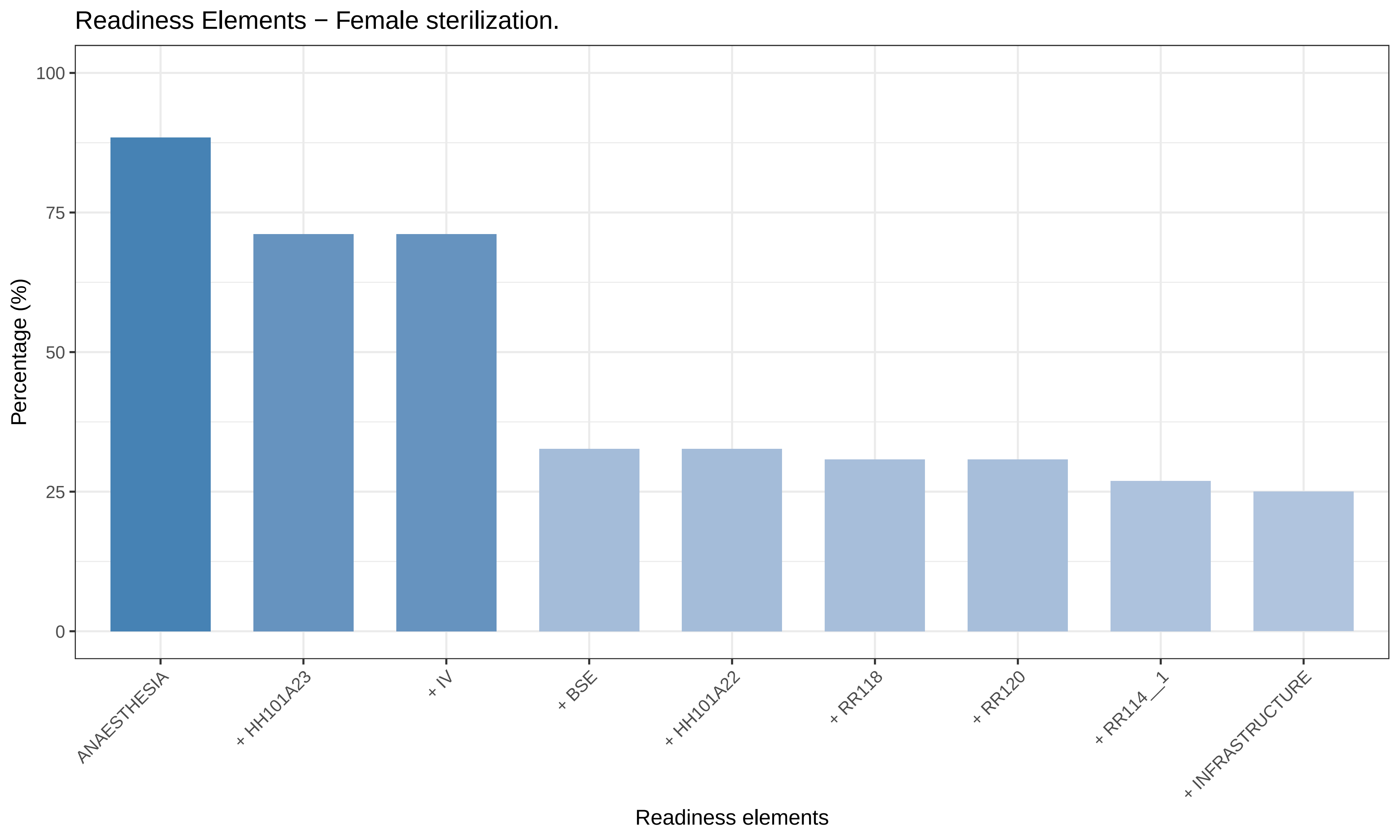
Readiness Elements − Female sterilization.
Percentage (%)
100
75
50
25
0
ANAESTHESIA
+ HH101A23
+ IV
+ BSE
+ HH101A22
+ RR118
+ RR120
+ RR114__1
+ INFRASTRUCTURE
Readiness elements

Readiness Elements − Dilatation and evacuation

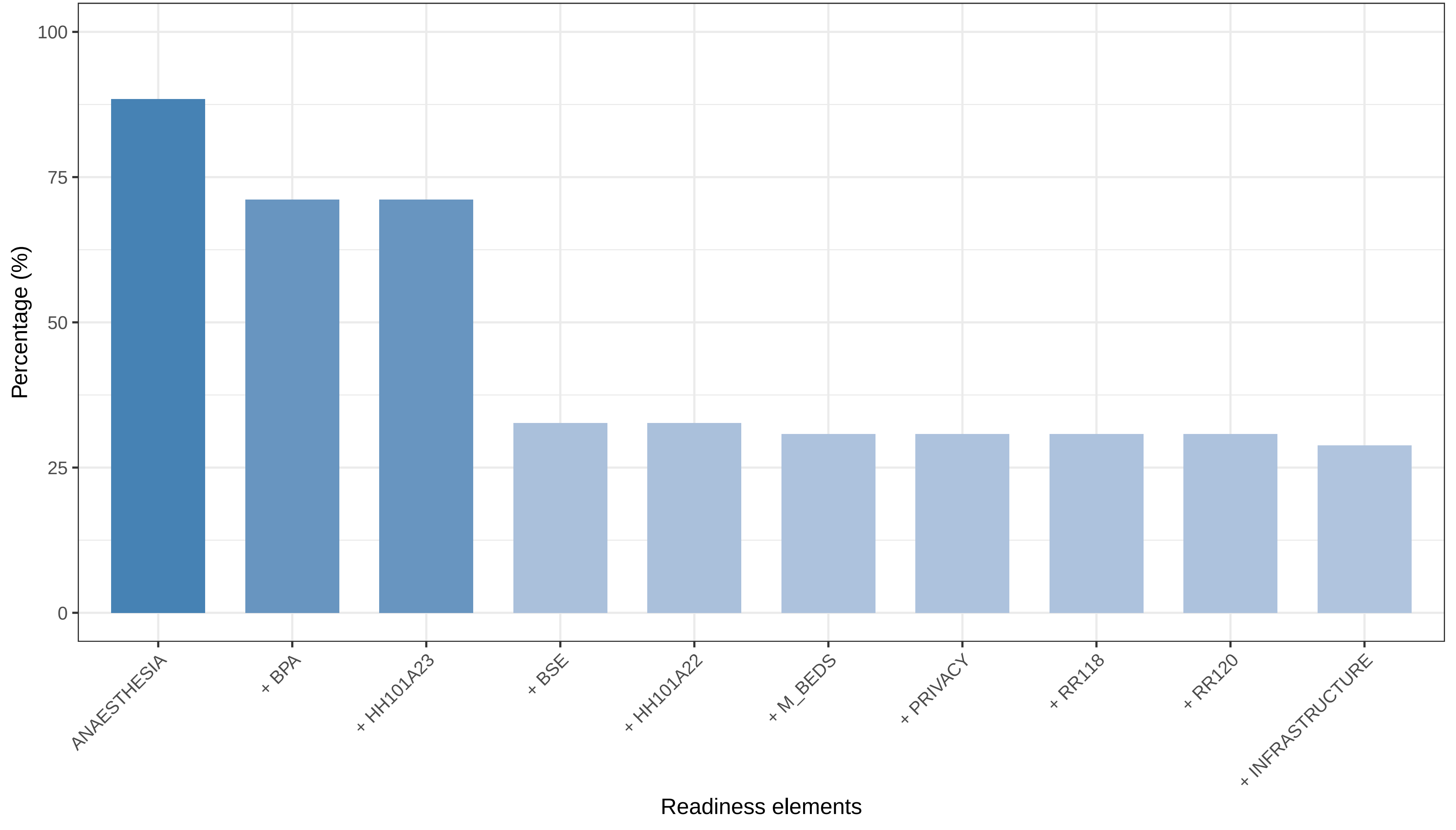

Readiness Elements − Oral prostaglandins and progesterone receptor antagonists for abortion

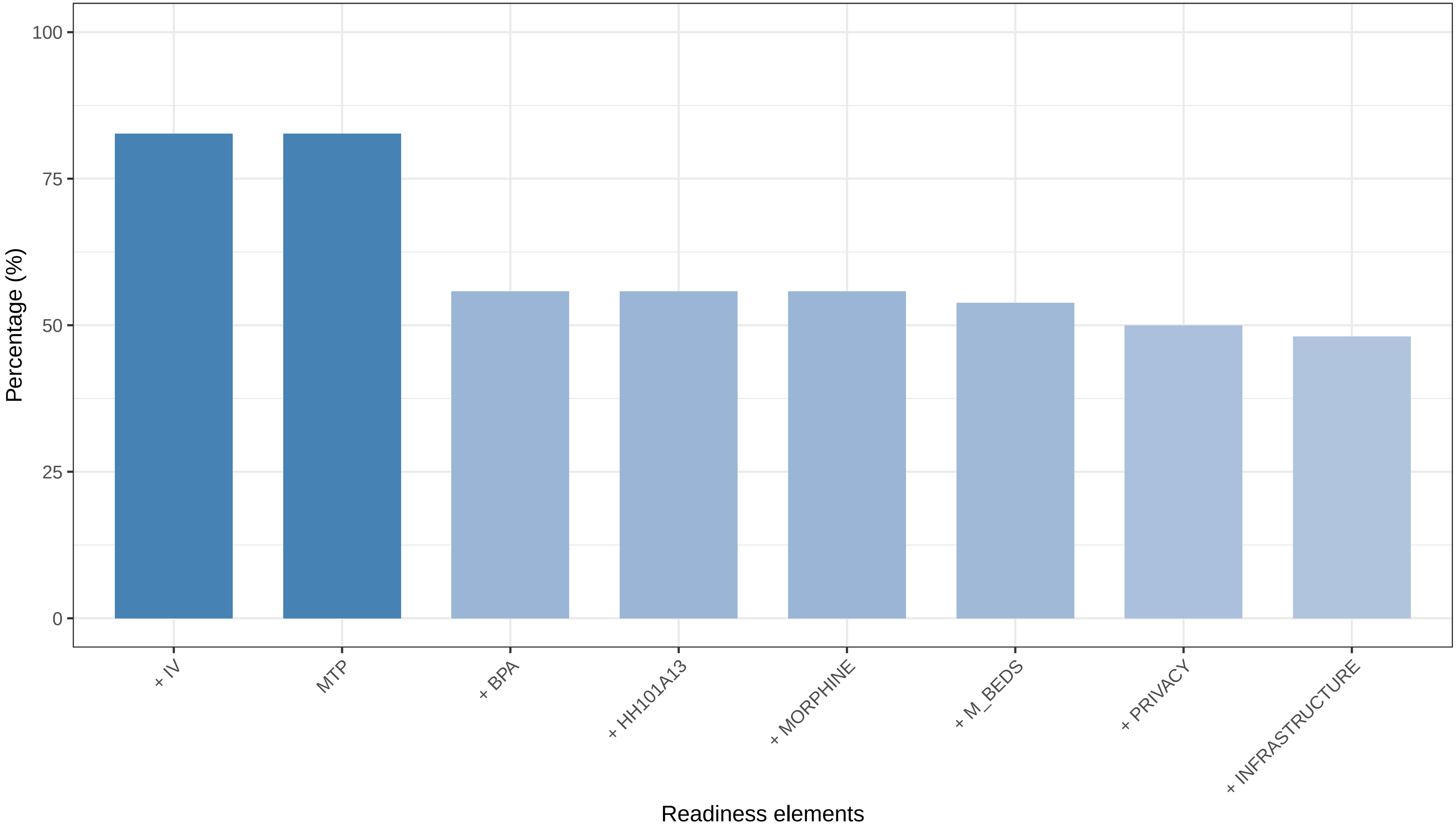

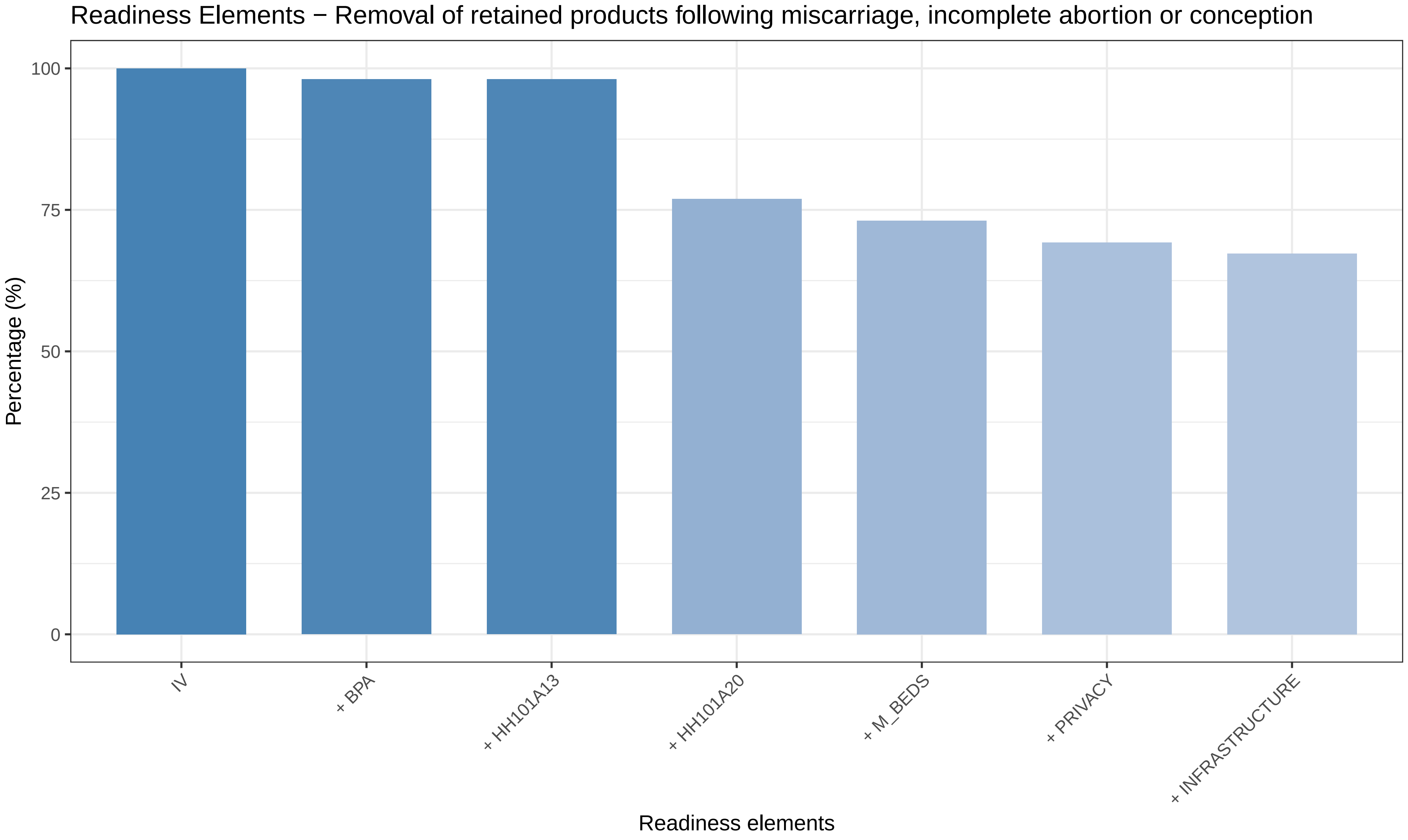

Readiness Elements − Removal of retained products following miscarriage, incomplete abortion or conception
Percentage (%)
100
75
50
25
0
IV
+ BPA
+ HH101A13
+ HH101A20
+ M_BEDS
+ PRIVACY
+ INFRASTRUCTURE
Readiness elements

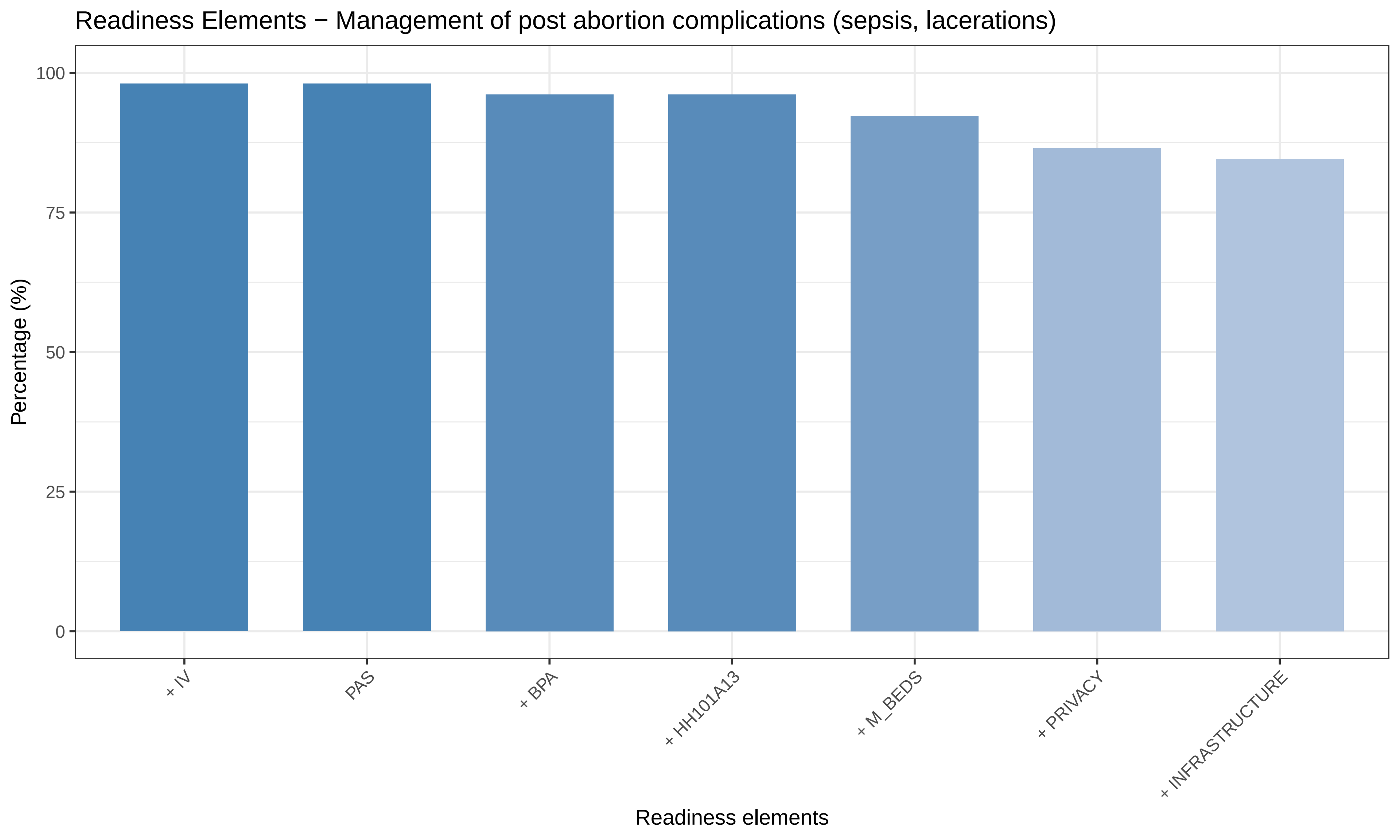
Readiness Elements − Management of post abortion complications (sepsis, lacerations)
Percentage (%)
100
75
50
25
0
+ IV
PAS
+ BPA
+ HH101A13
+ M_BEDS
+ PRIVACY
+ INFRASTRUCTURE
Readiness elements

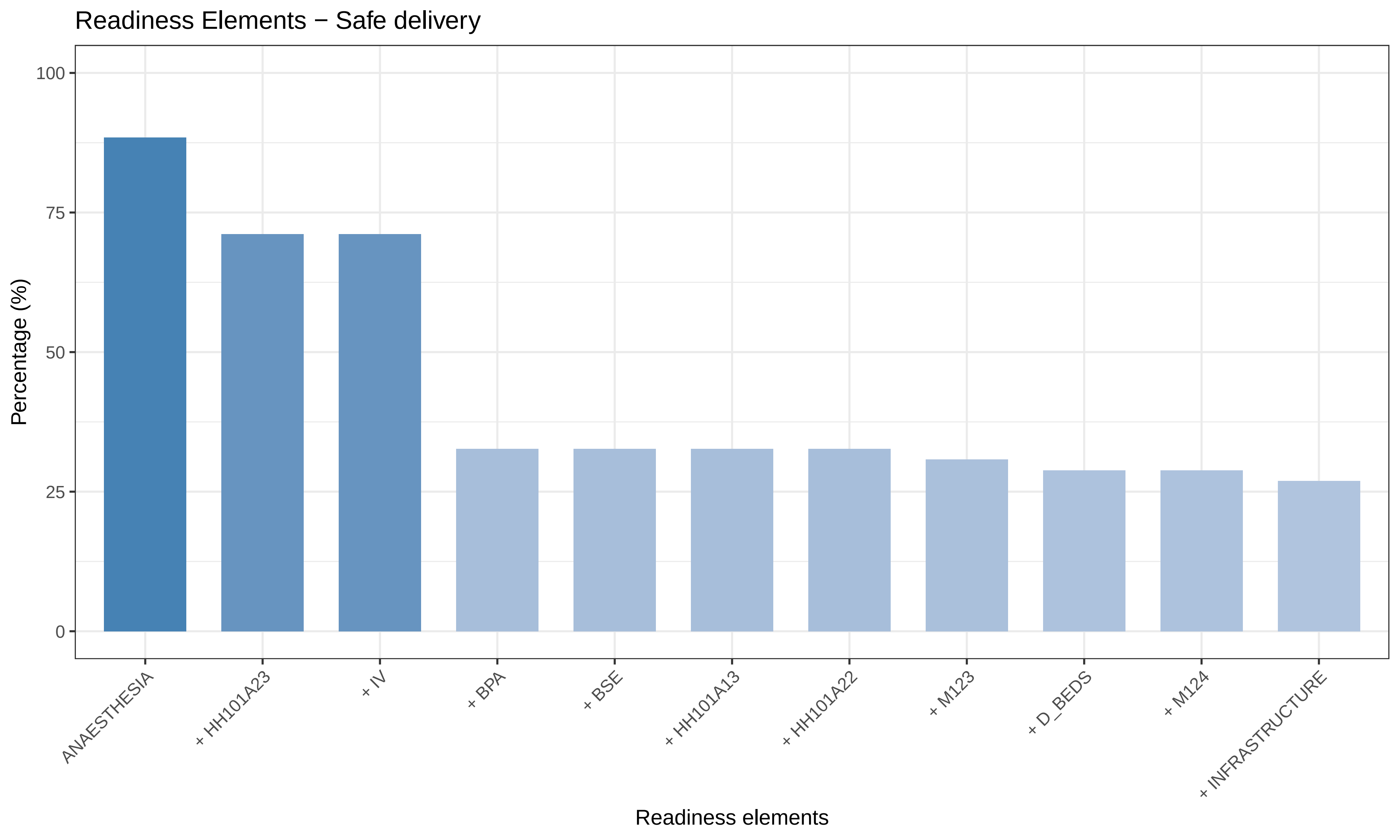
Readiness Elements − Safe delivery
Percentage (%)
100
75
50
25
0
ANAESTHESIA
+ HH101A23
+ IV
+ BPA
+ BSE
+ HH101A13
+ HH101A22
+ M123
+ D_BEDS
+ M124
+ INFRASTRUCTURE
Readiness elements

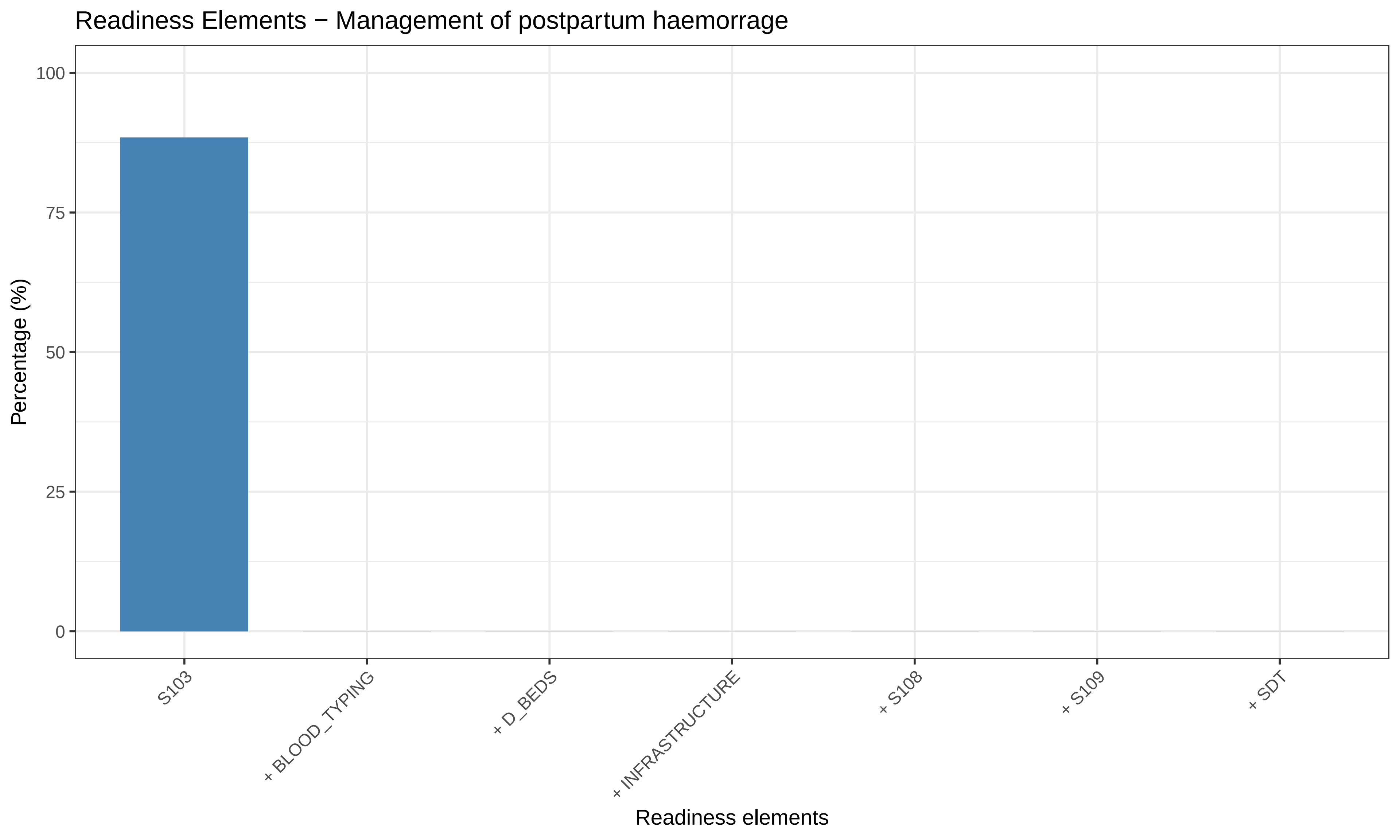
Readiness Elements − Management of postpartum haemorrage
Percentage (%)
100
75
50
25
0
S103
+ BLOOD_TYPING
+ D_BEDS
+ INFRASTRUCTURE
+ S108
+ S109
+ SDT
Readiness elements

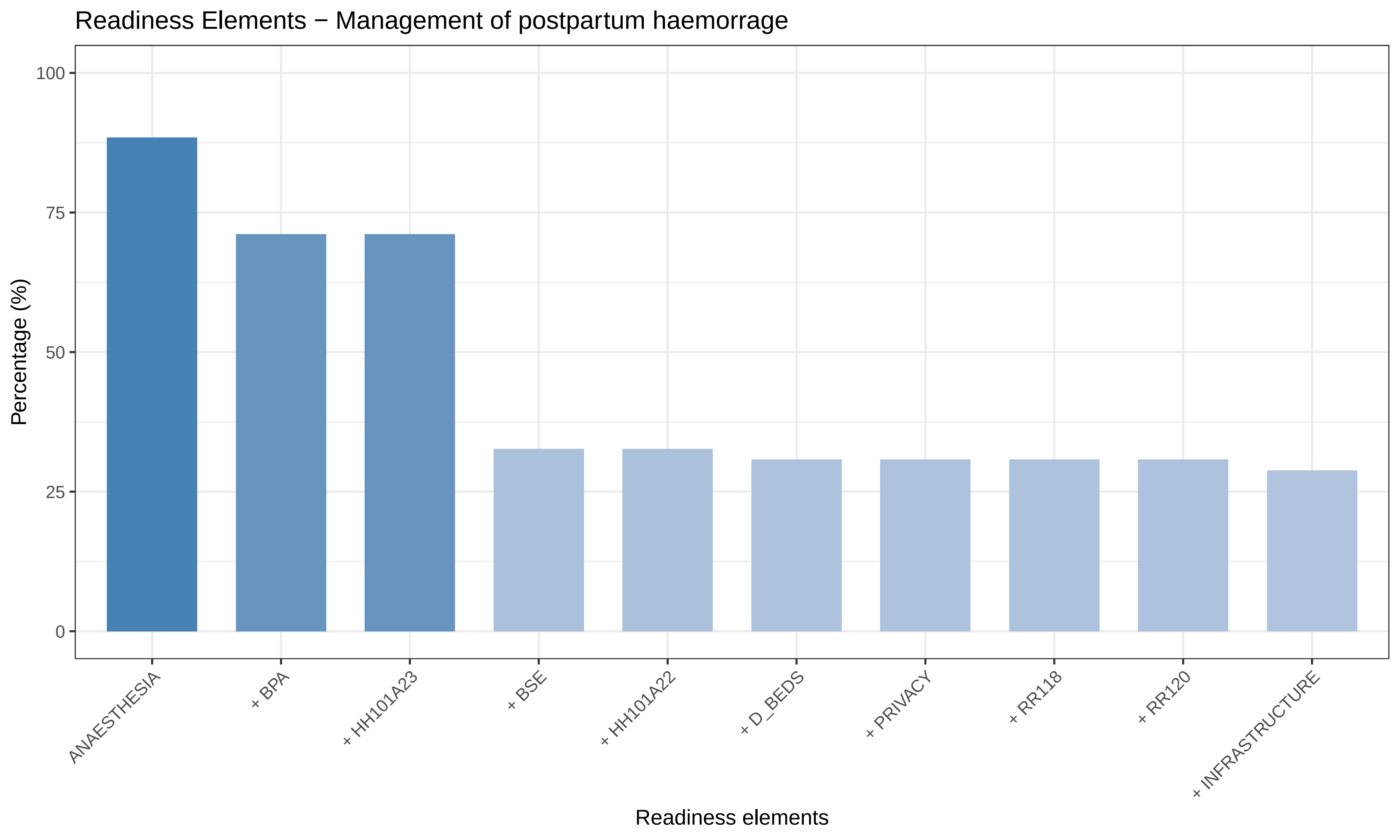

Readiness Elements − Management of postpartum haemorrage
Percentage (%)
100
75
50
25
0
ANAESTHESIA
+ BPA
+ HH101A23
+ BSE
+ HH101A22
+ D_BEDS
+ PRIVACY
+ RR118
+ RR120
+ INFRASTRUCTURE
Readiness elements
