# Supplementary material for: Measures to monitor the implementation of Essential Health Benefit Packages at a national scale
Source: Npj Health Syst. 2026 Apr 29;3:27. doi: 10.1038/s44401-026-00081-4 (PMC13354221; doi:10.1038/s44401-026-00081-4)

District level coverage
Basic Primary Health Care

Routine childhood immunization

Pentavalent vaccine (DPT−HepB−Hib)

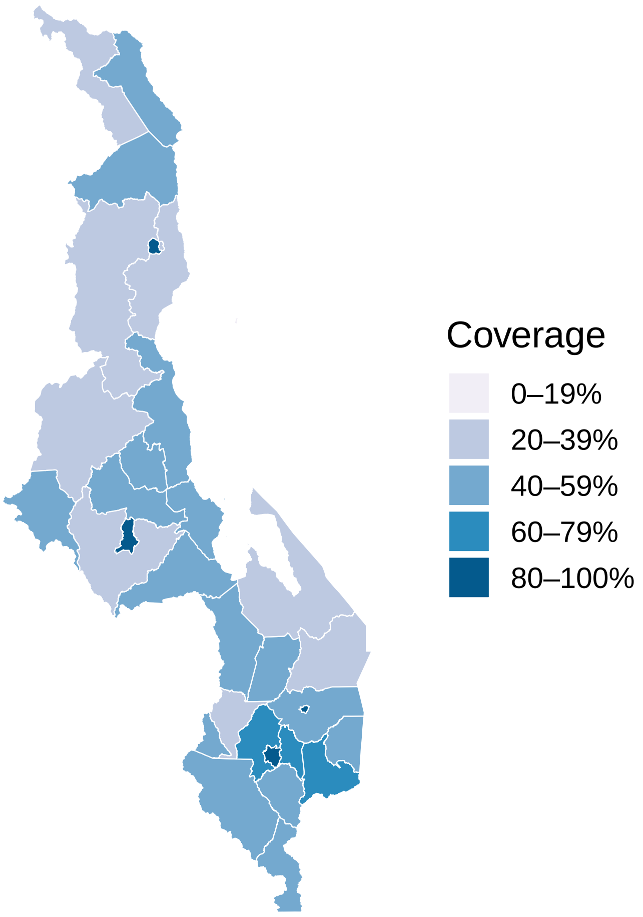

Drug−susceptibleTB management

Management of drug susceptible pulmonary TB

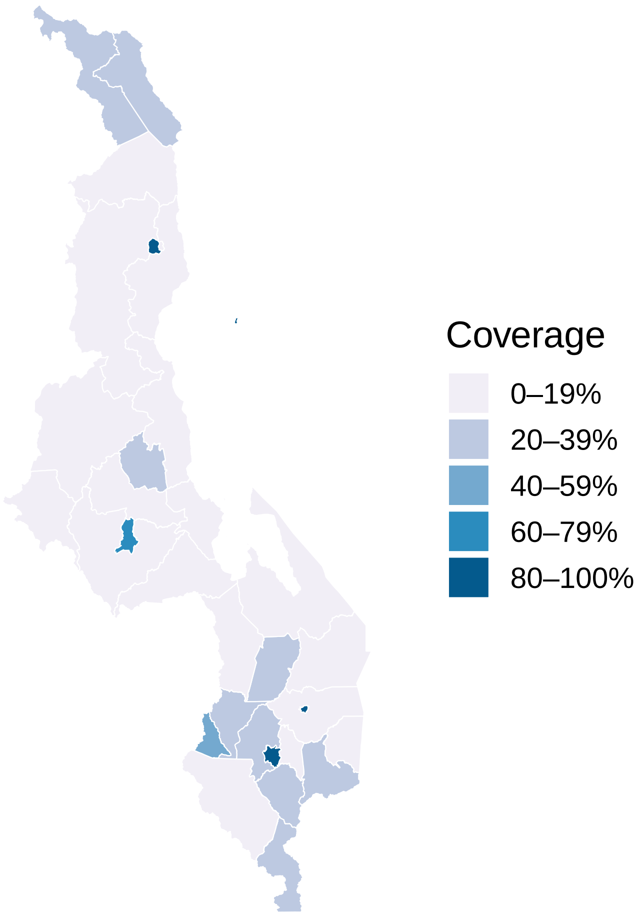

Drug−susceptibleTB management

Management of drug susceptible extrapulmonary TB

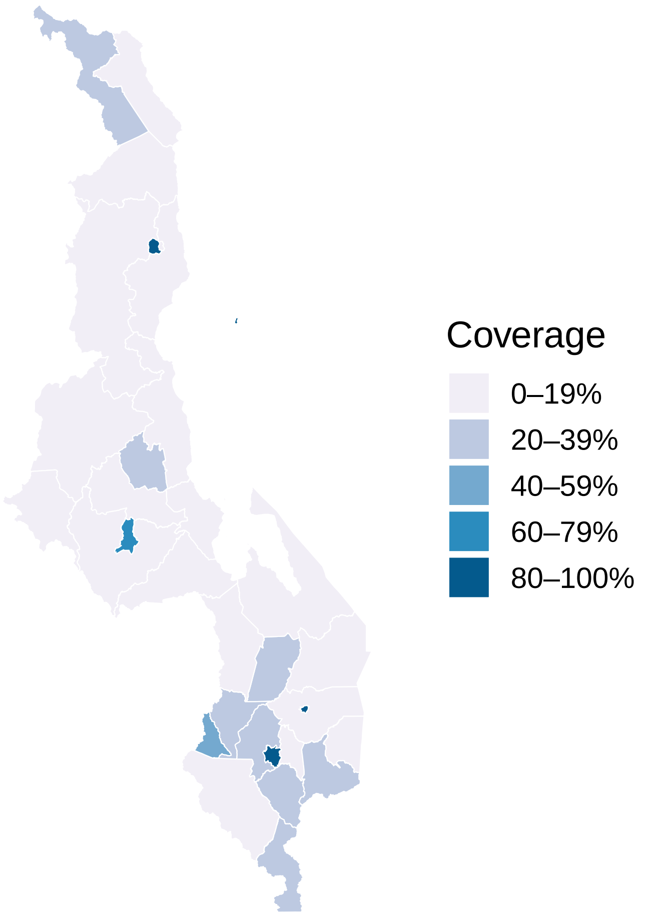

Malaria prevention

Intermittent malaria prevention in infancy

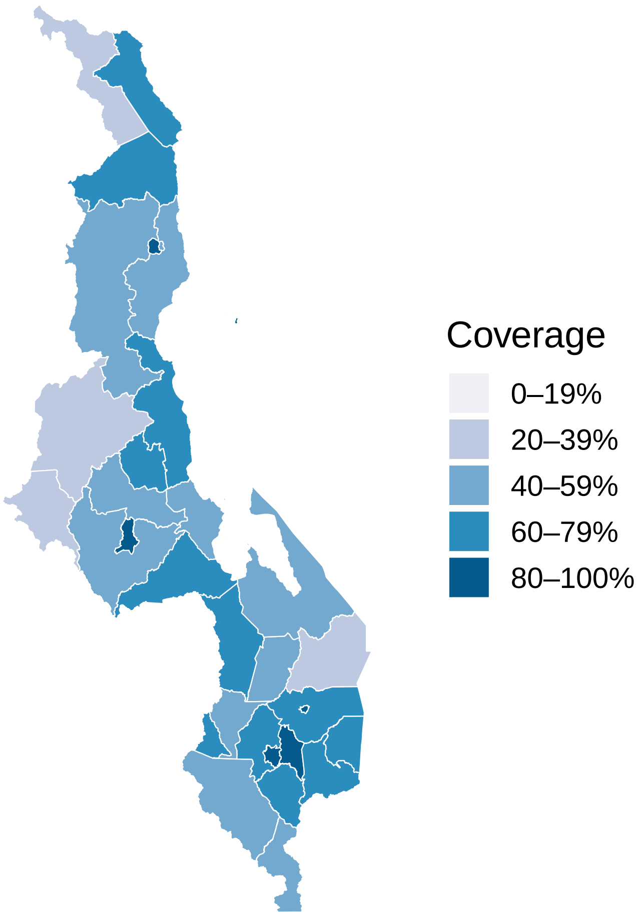

Malaria prevention

Intermittent malaria prevention during pregnancy

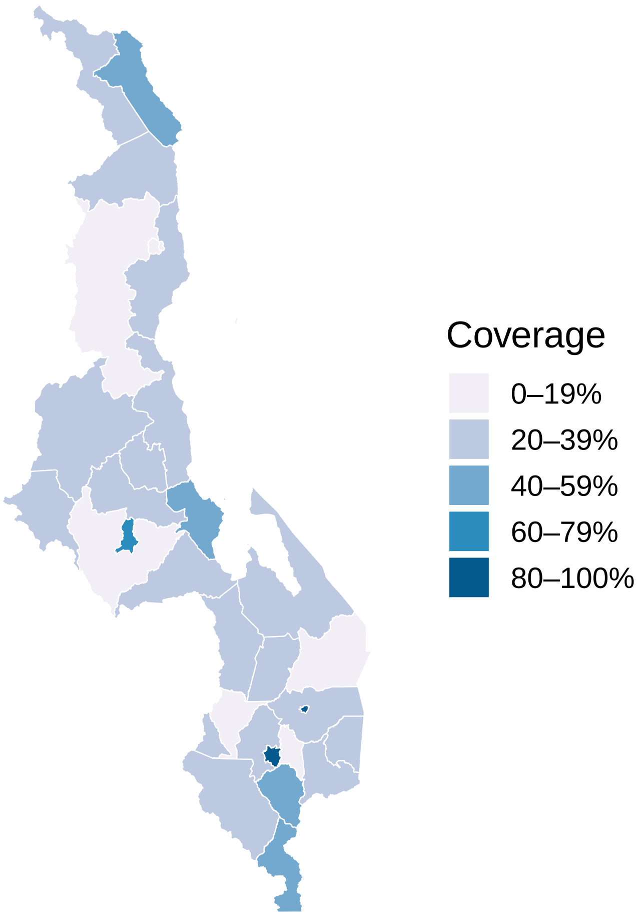

Management of malaria

Treatment of uncomplicated malaria

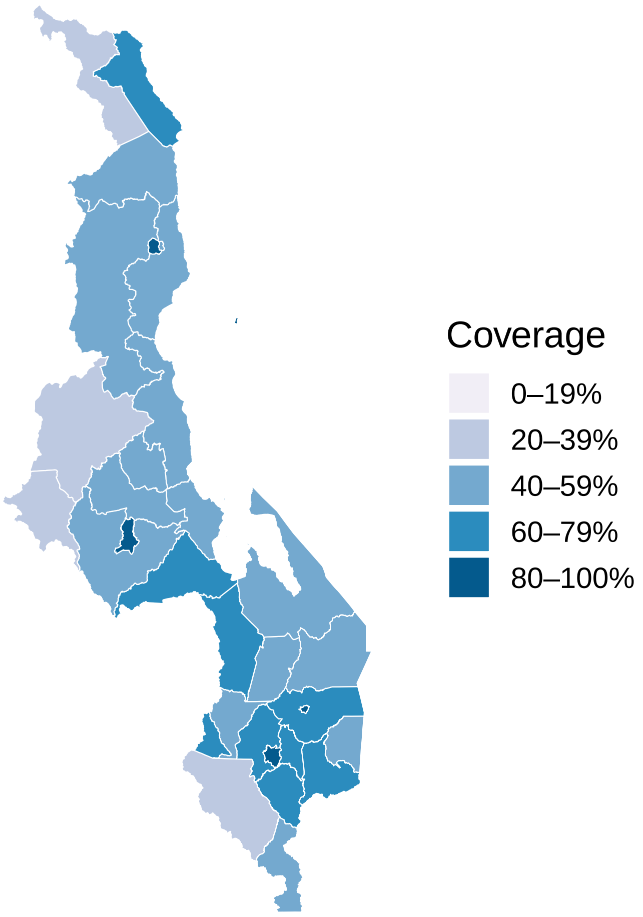

Supportive care for acute hepatitis A

Management of acute hepatitis A in children

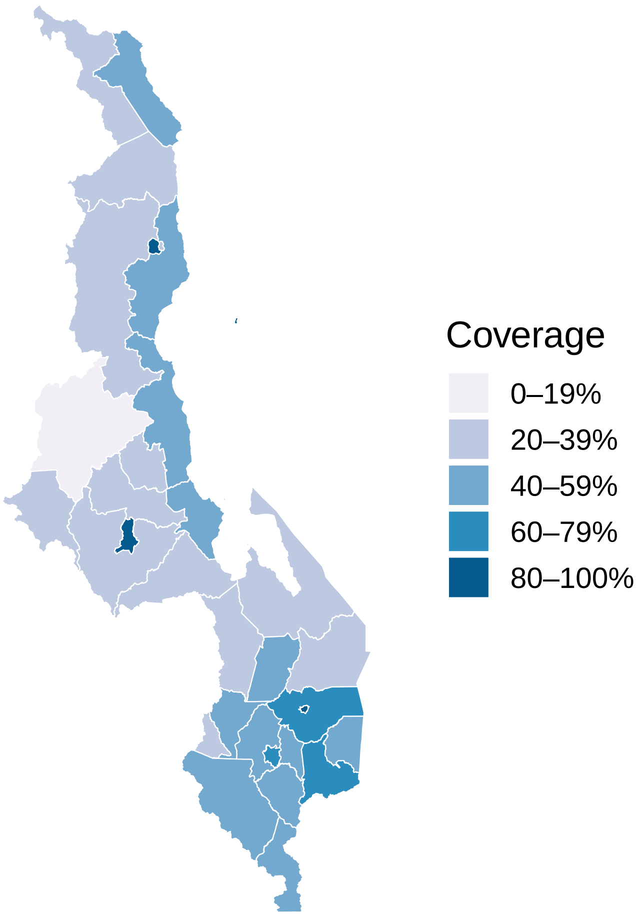

Supportive care for acute hepatitis A

Management of acute hepatitis A in adults

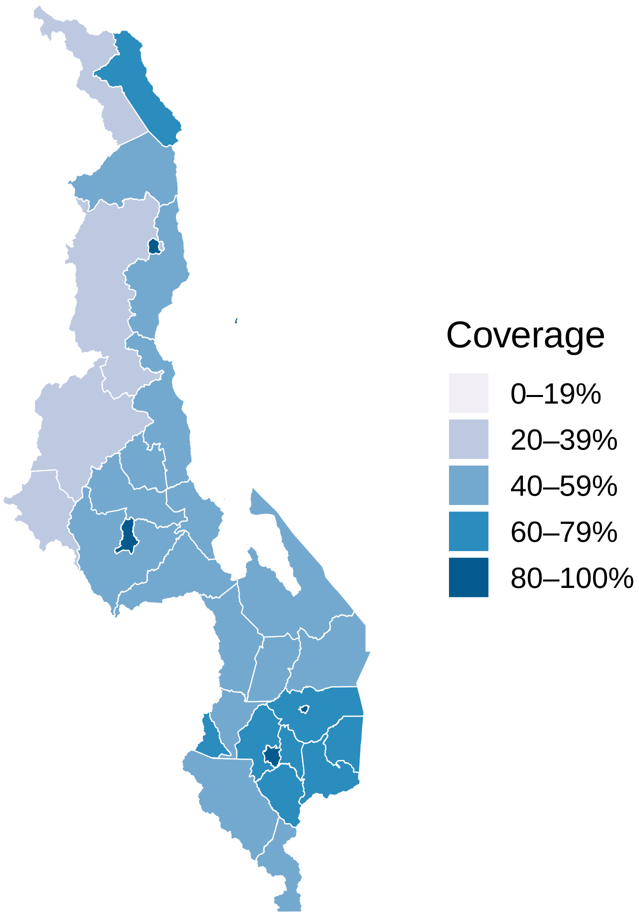

Treatment of acute lower respiratory infections, children

Pneumonia, oral antibiotics

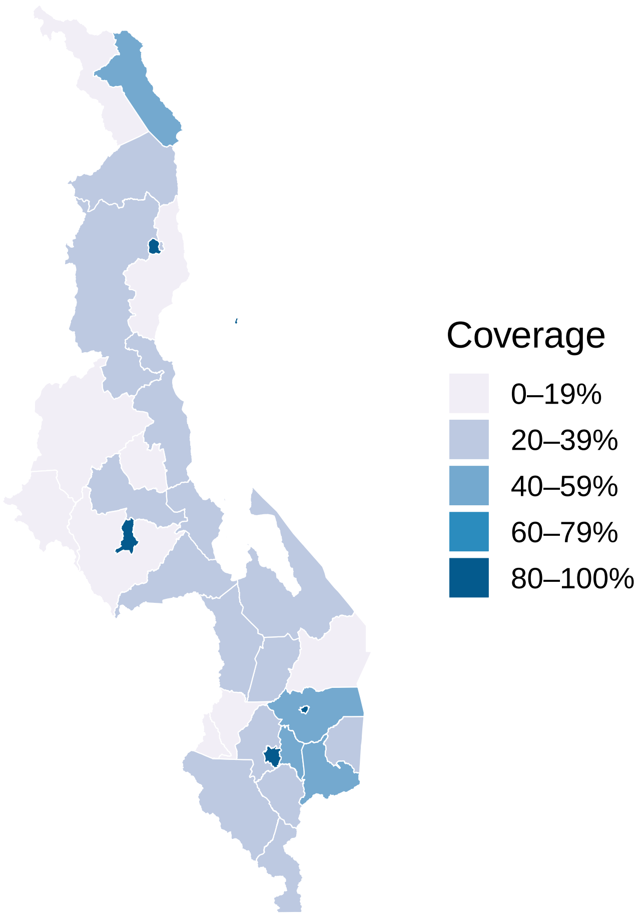

Treatment of acute lower respiratory infections, children

Pneumonia (severe), IV antibiotics

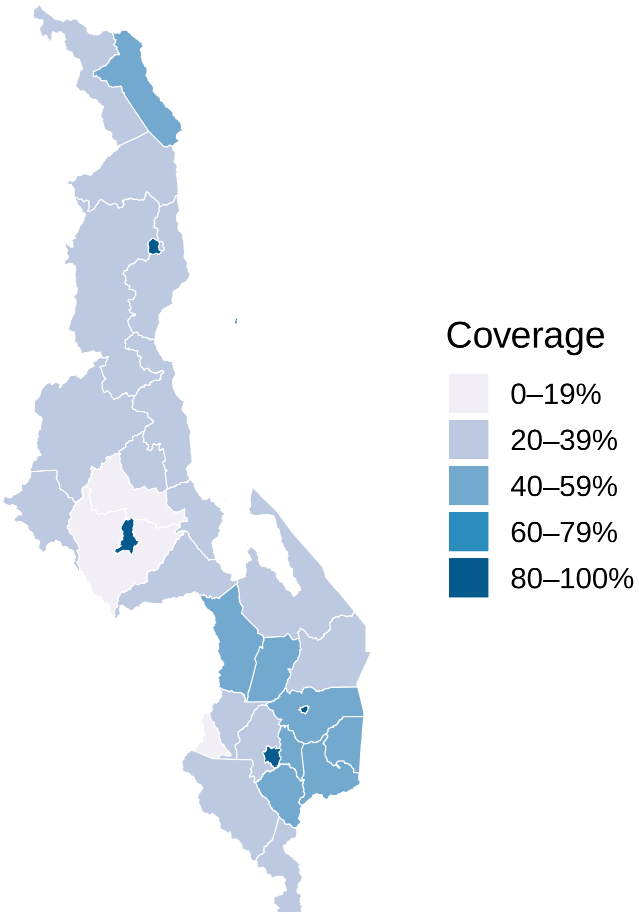

Treatment of acute lower respiratory infections, adults

Pneumonia, oral antibiotics Adults

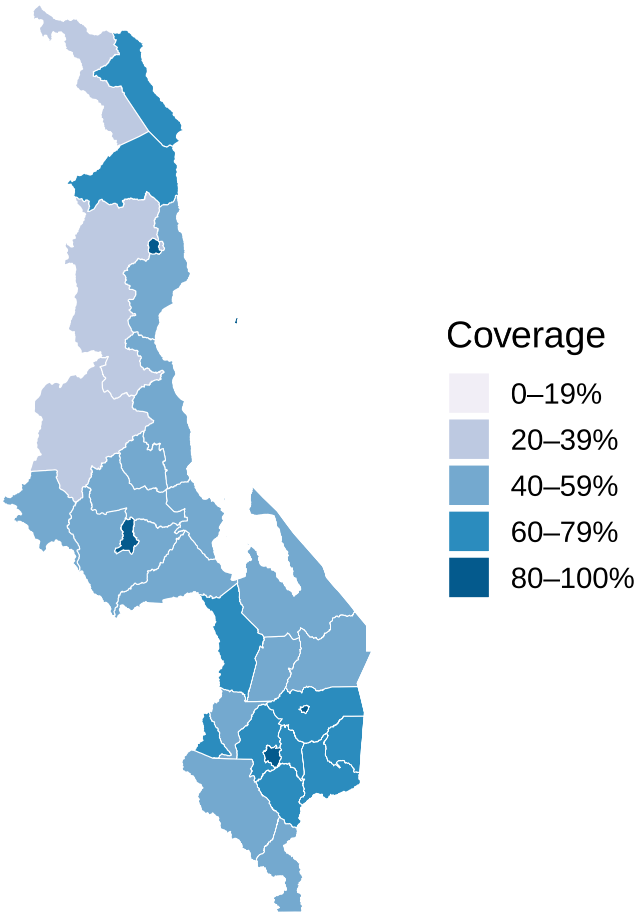

Routine childhood immunization

BCG vaccine

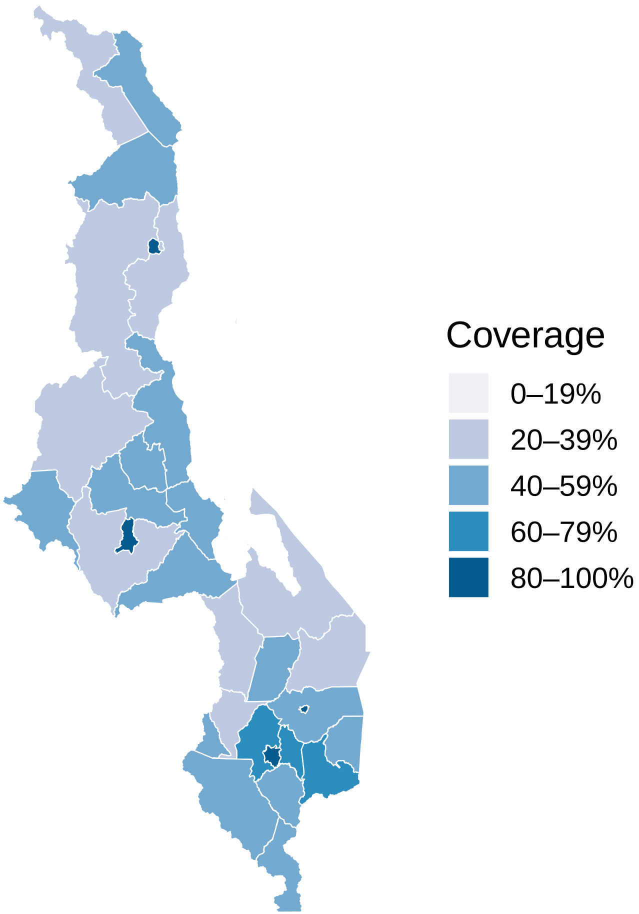

Treatment of acute lower respiratory infections, adults

Pneumonia (severe), IV antibiotics adults

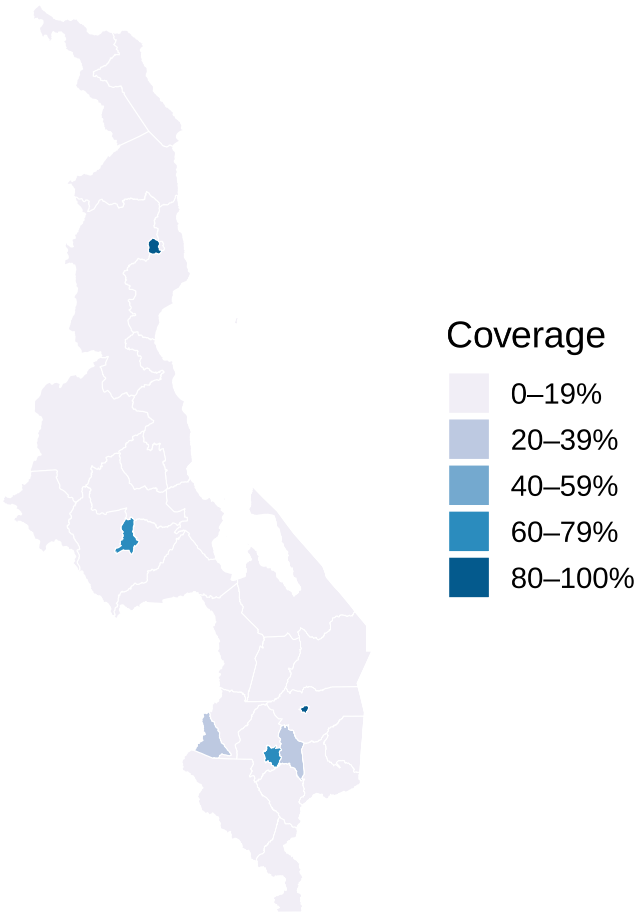

Treatment of diarrheal diseases

Treatment of acute diarrhea in adults

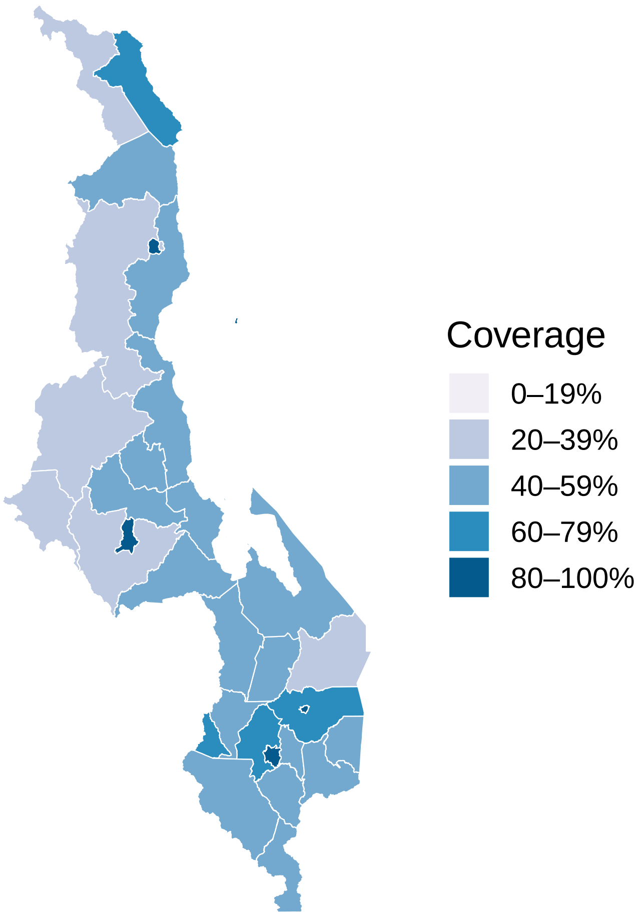

Treatment of diarrheal diseases

Treatment of acute diarrhea in children

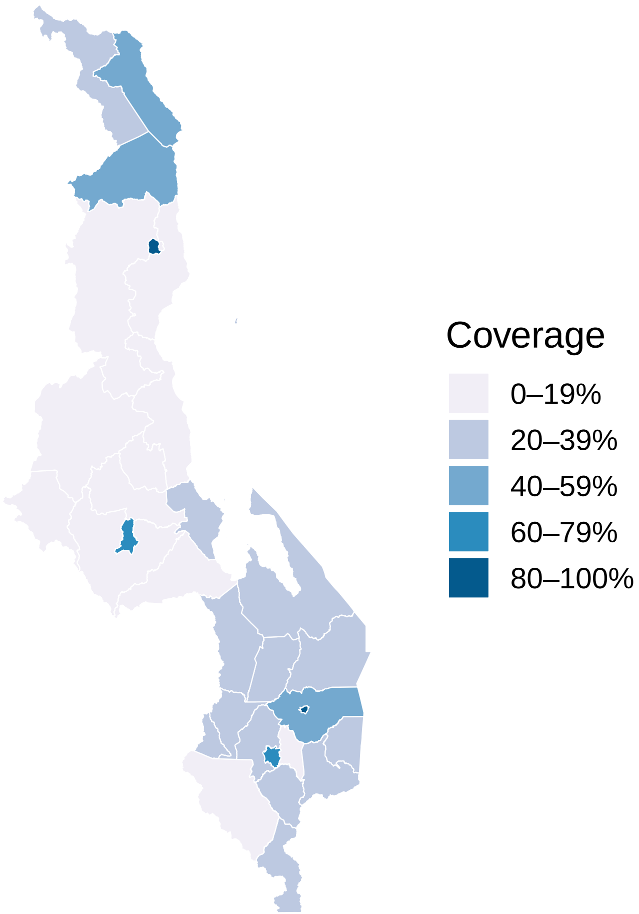

Treatment of diarrheal diseases

Treatment of acute diarrhea in children

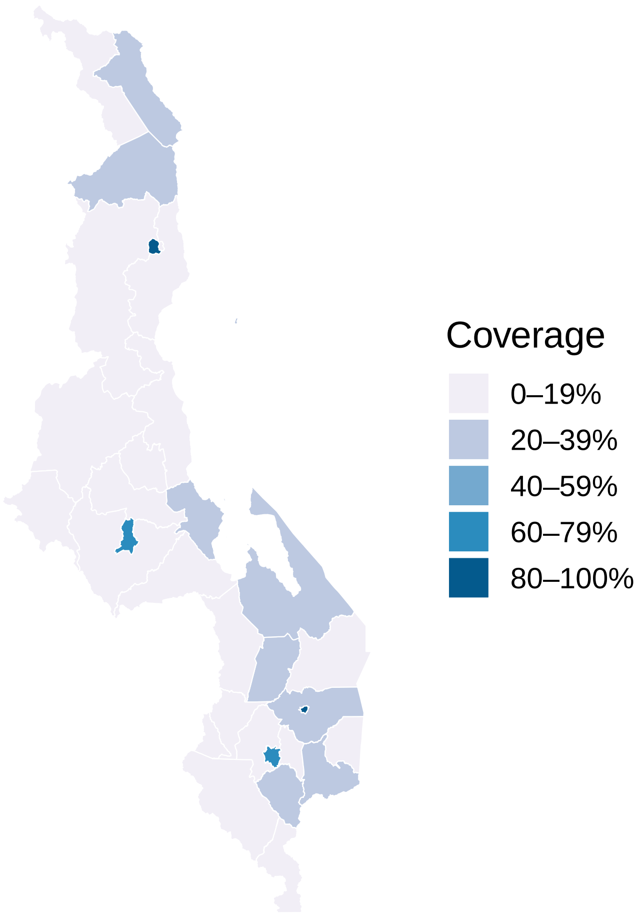

Treatment of typhoid and paratyphoid

Treatment of typhoid and paratyphoid in children

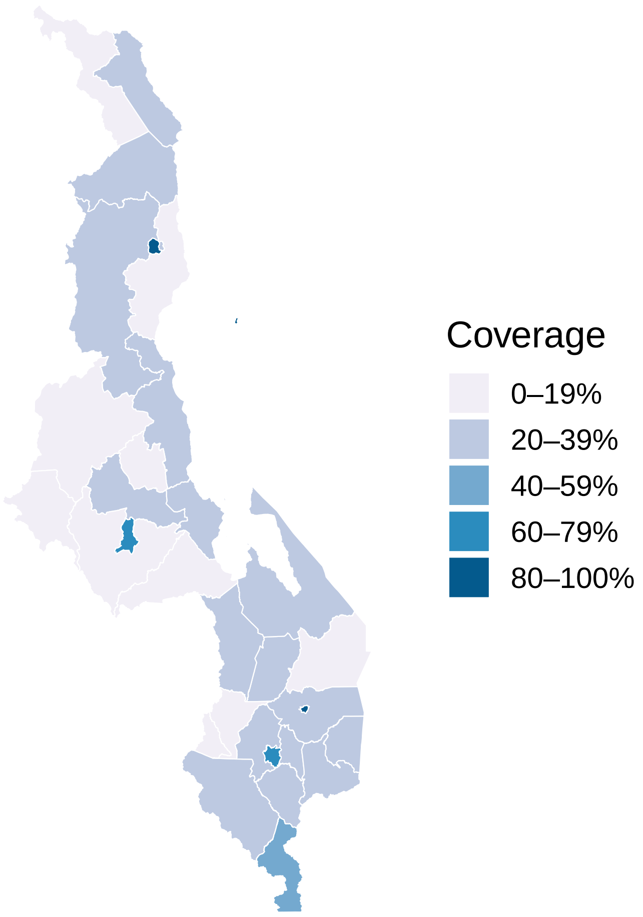

Treatment of typhoid and paratyphoid

Treatment of typhoid and paratyphoid in adults

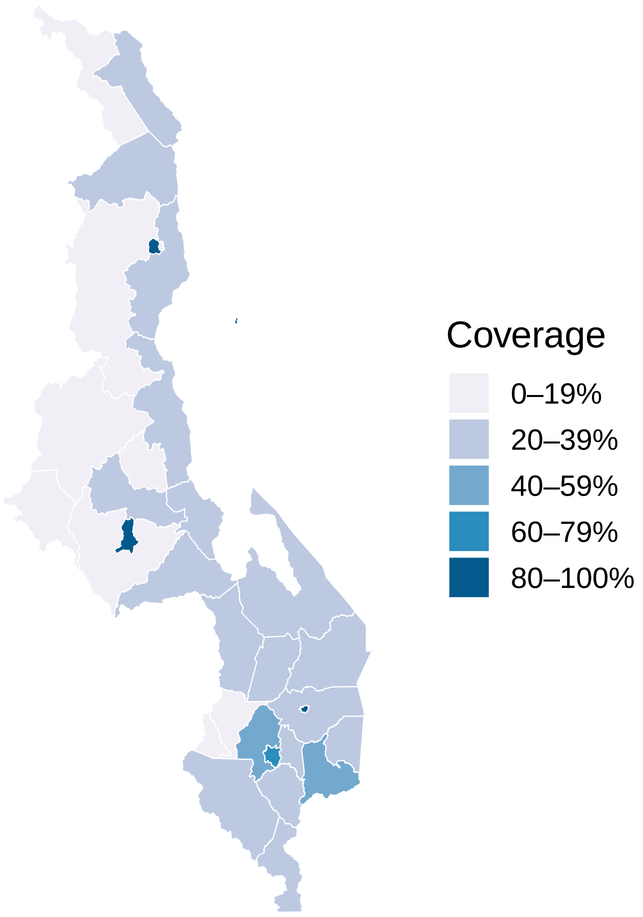

Treatment of syphilis

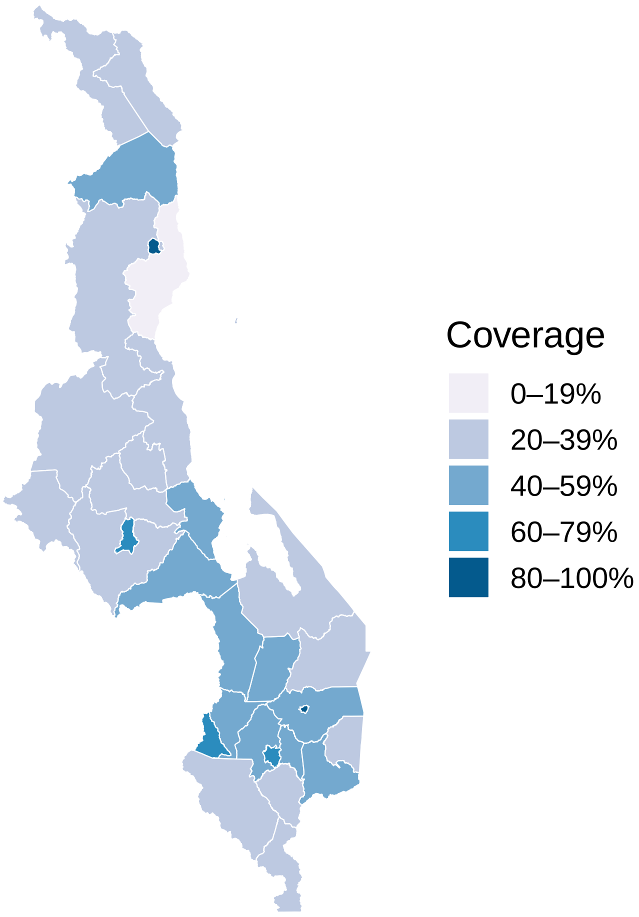

Treatment of gonorrhea

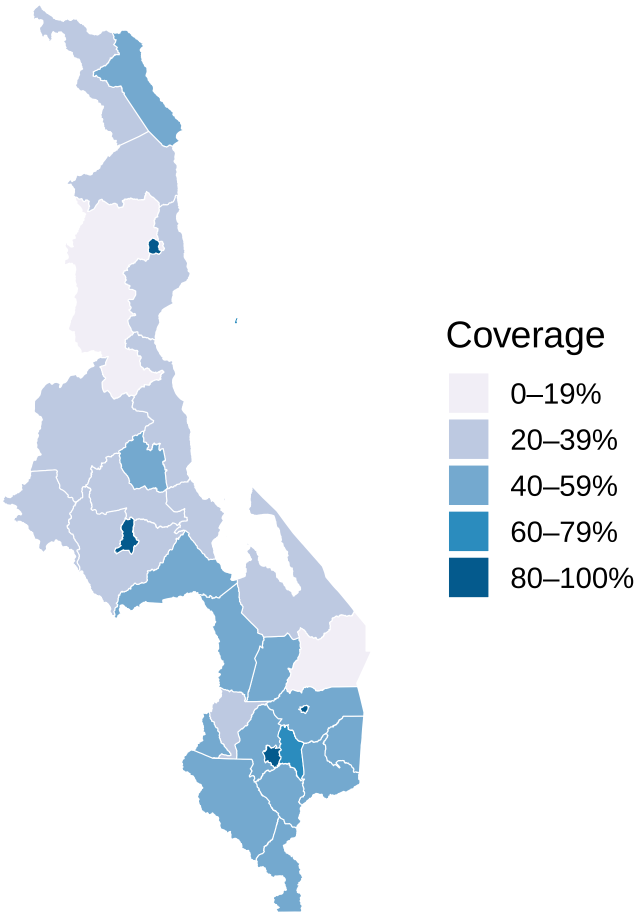

Treatment of chlamydia

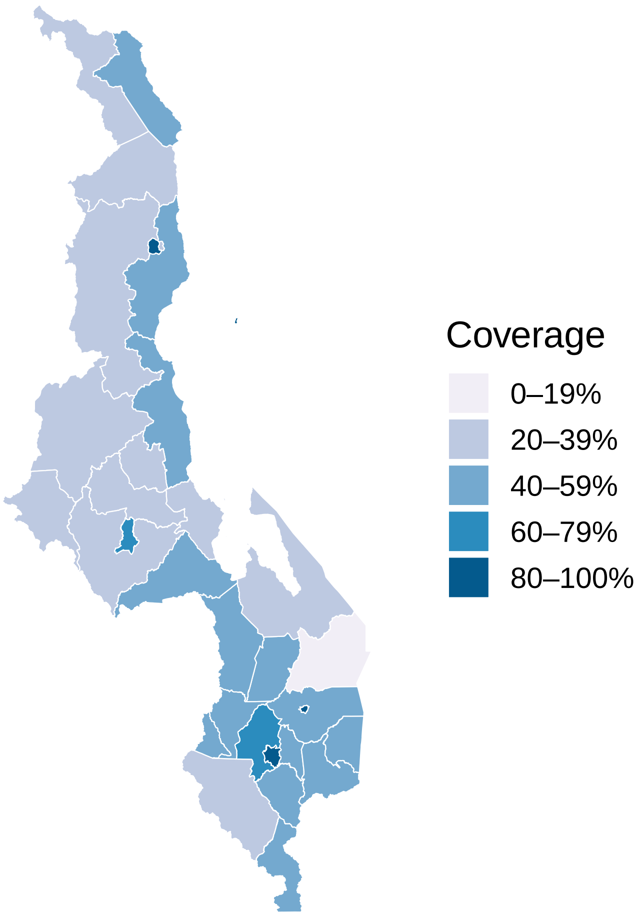

Treatment of trichomoniasis

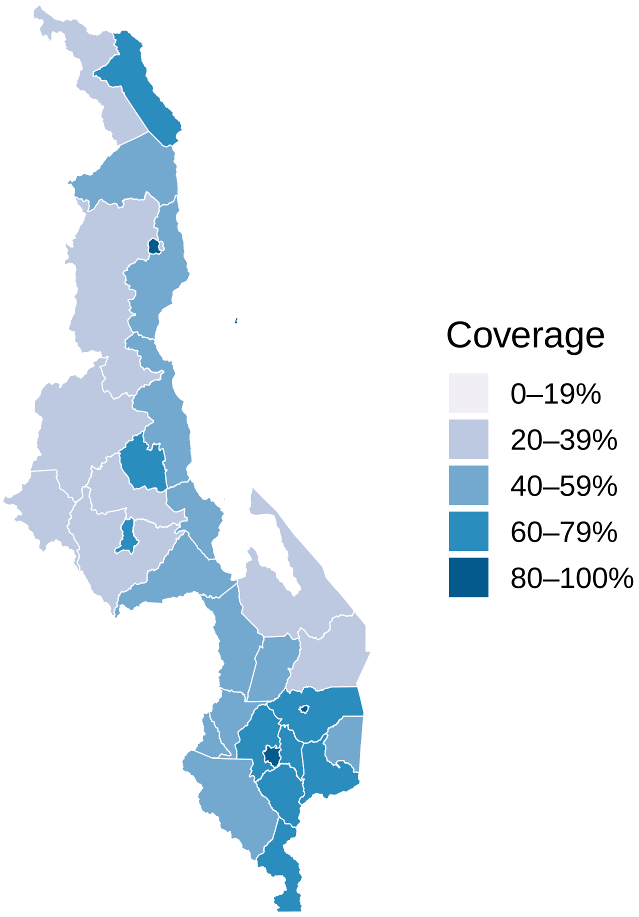

Routine childhood immunization

MMR vaccine

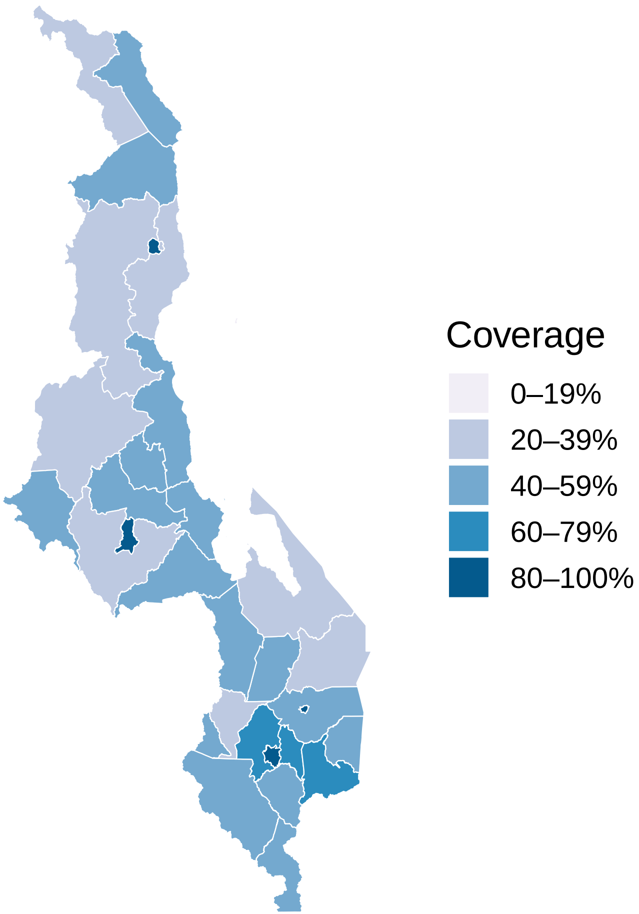

Treatment of urinary tract infection

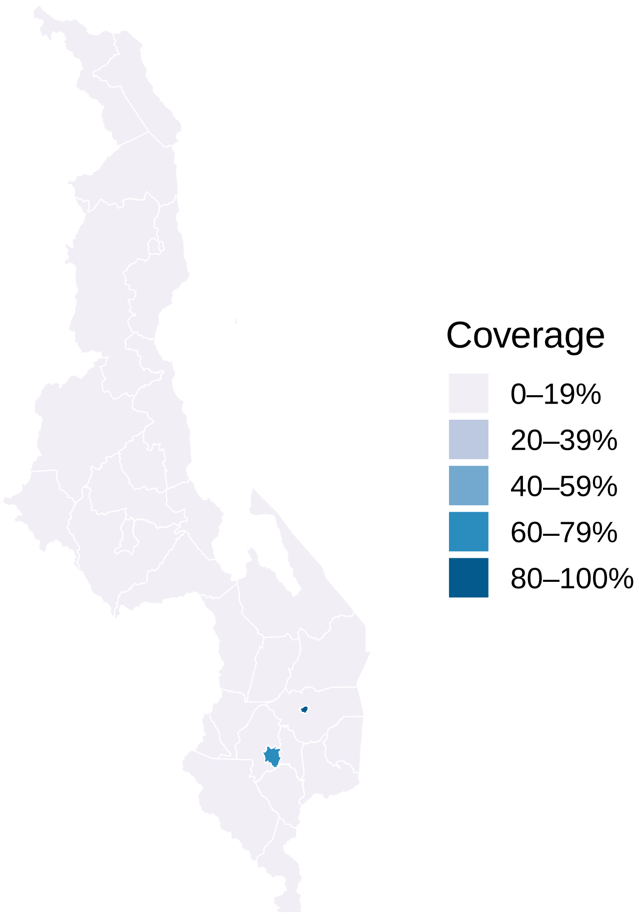

Treatment of ear infections and complications

Treatment of upper respiratory tract infections and ear infections ar

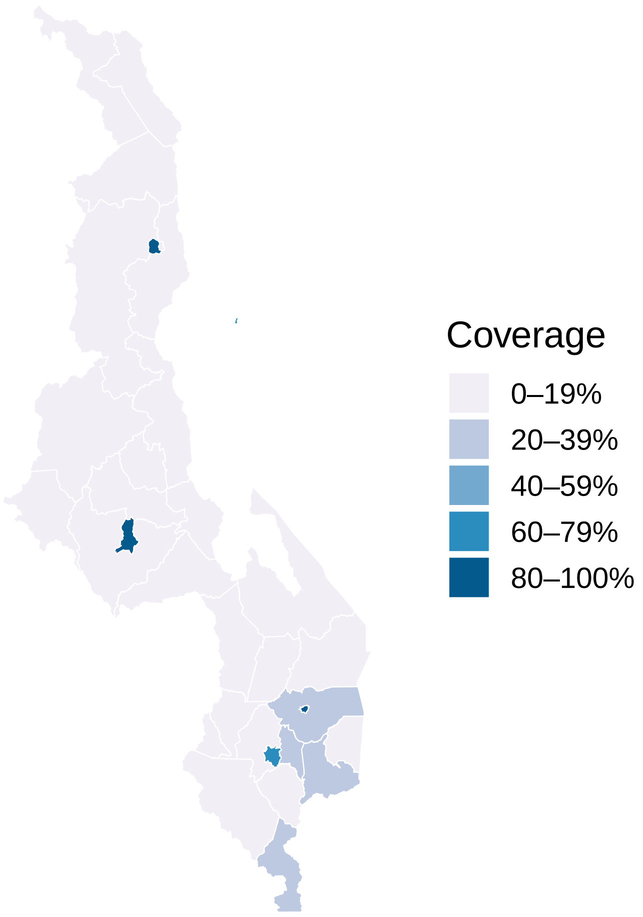

Treatment of measles: including Vitamin A to children 6 to 59 [illegible]

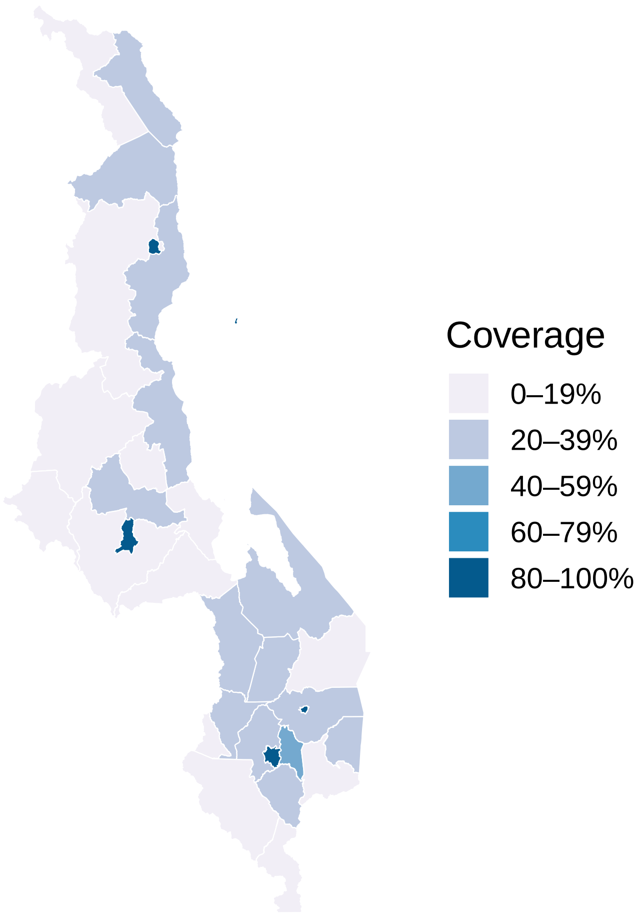

Routine childhood immunization

Polio vaccine (Oral|IPV)

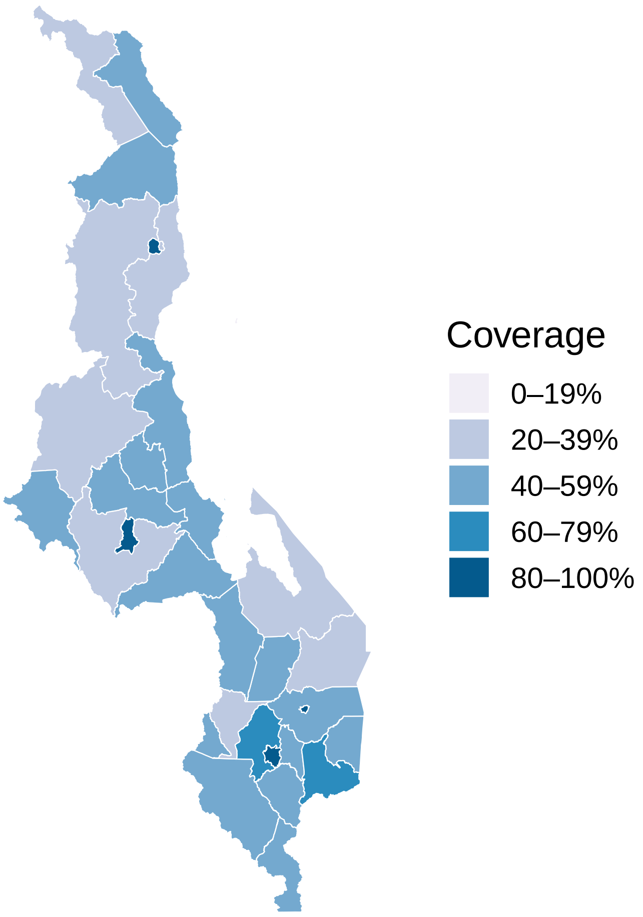

Routine childhood immunization

Pneumococcal vaccine

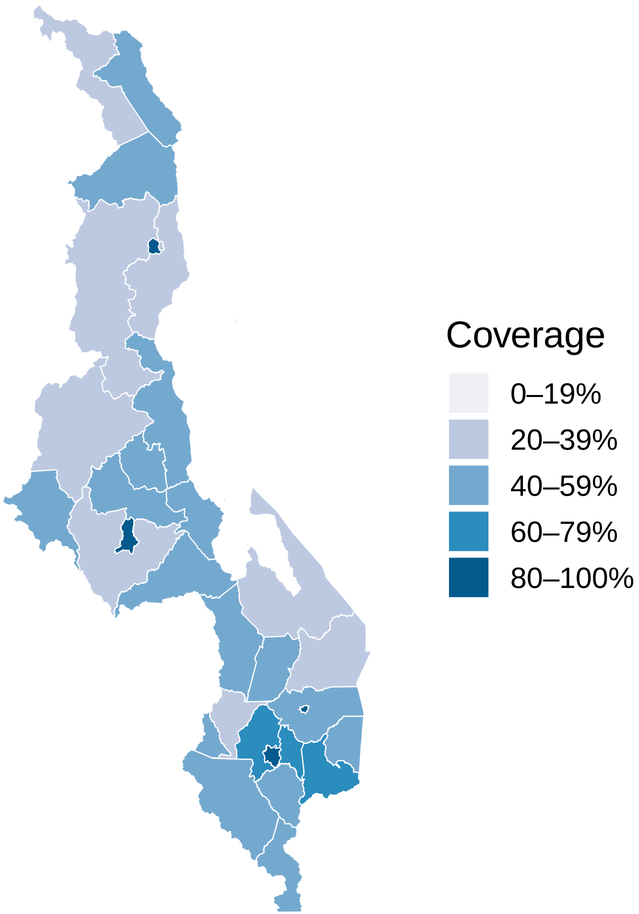

Routine childhood immunization

Rotavirus vaccine

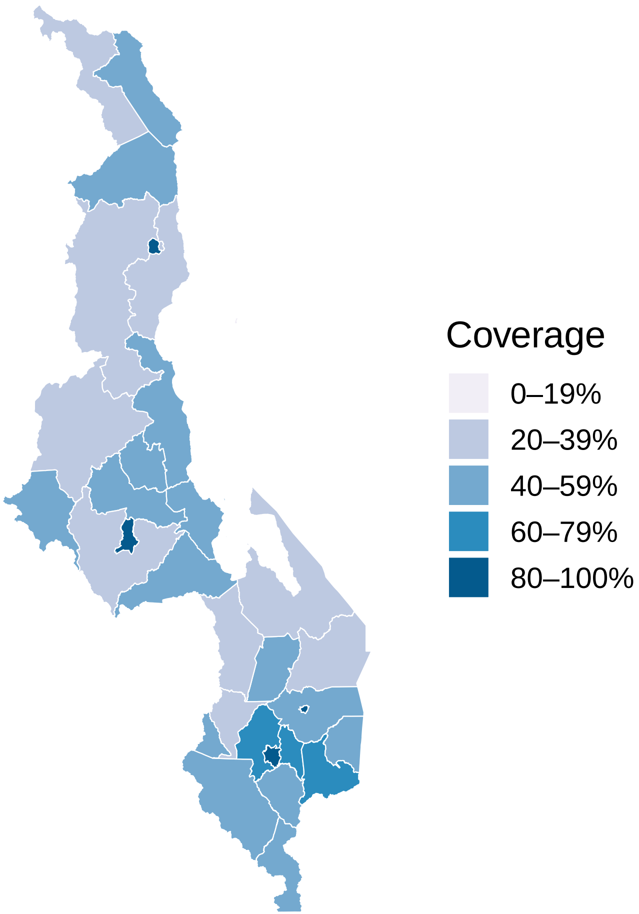

Management of HIV

HIV treatment, ART first−line (no TB)

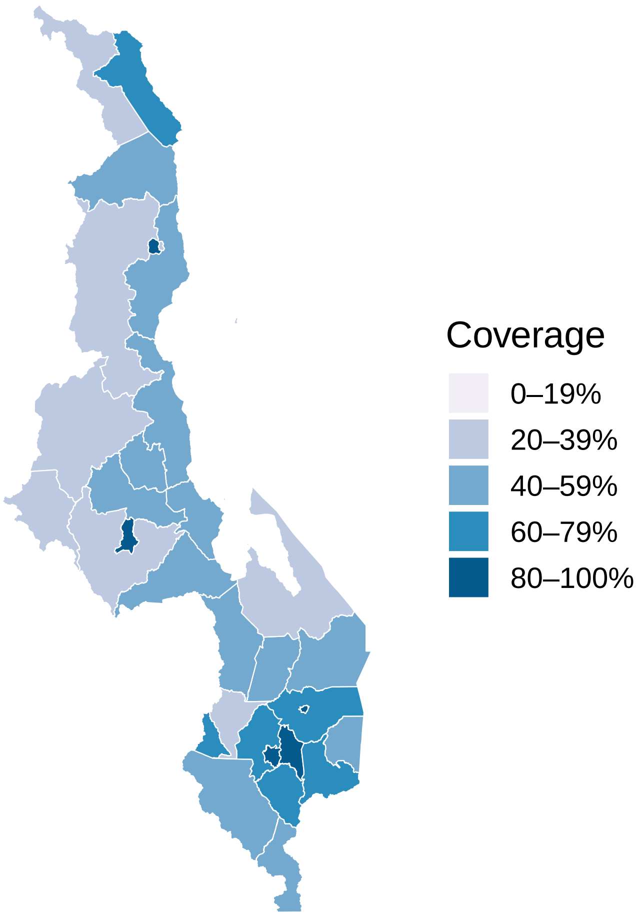

HIV prevention

Voluntary medical male circumcision service in settings with high p

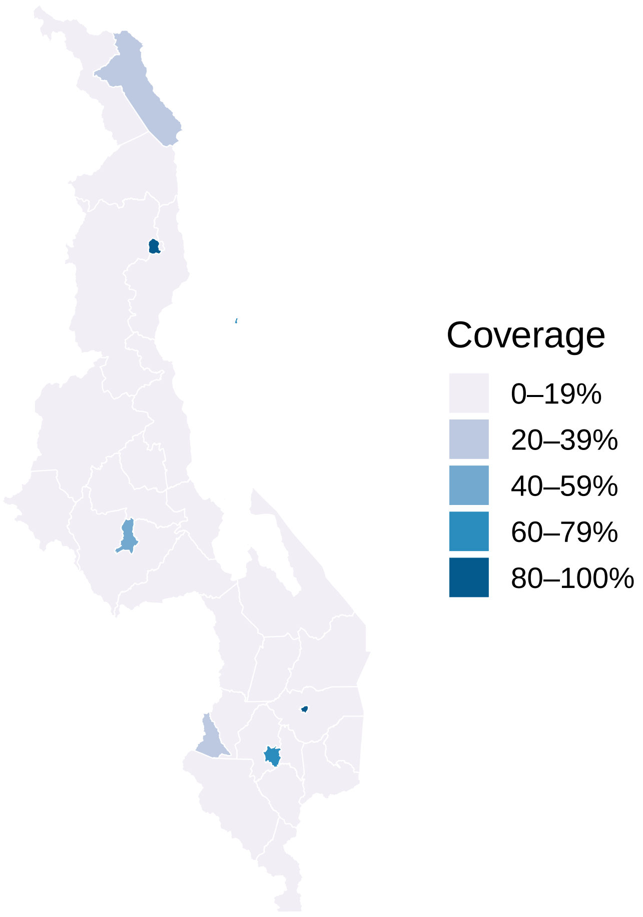

Prevention of tuberculosis

TB preventive therapy(Isoniazide) for high risk people (e.g. PLHIV)

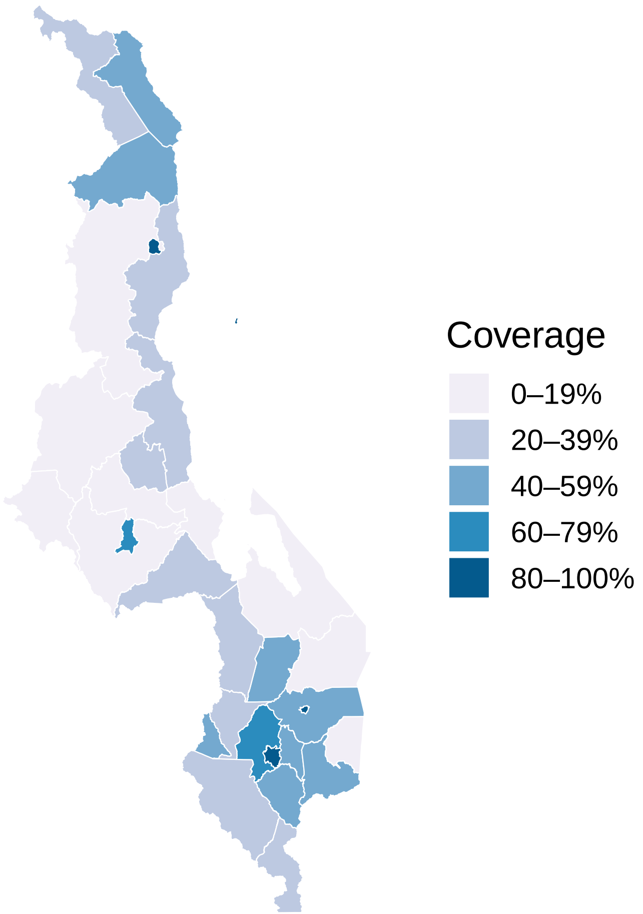

Fever evaluation, basic

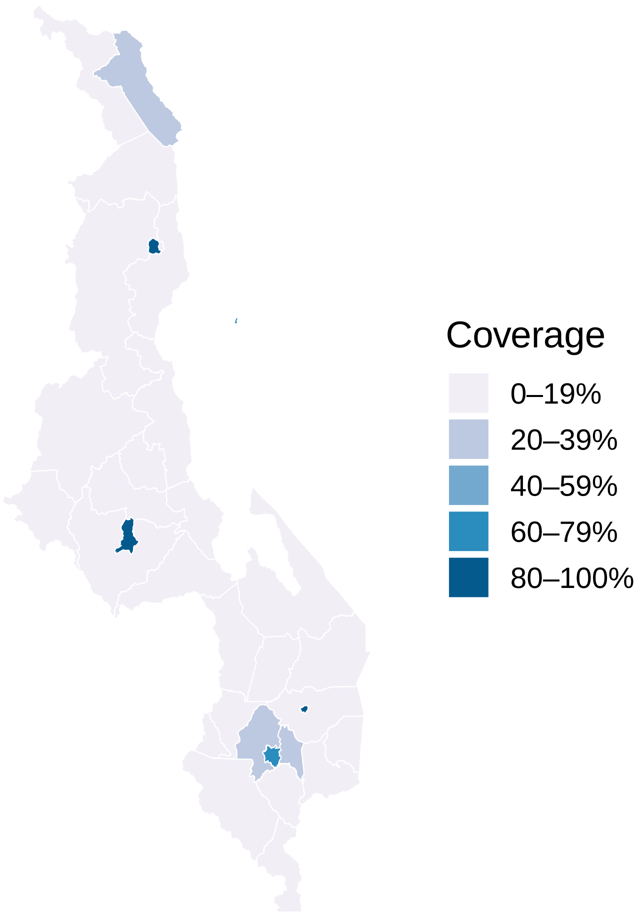

Prehospital triage and stablization, with referral

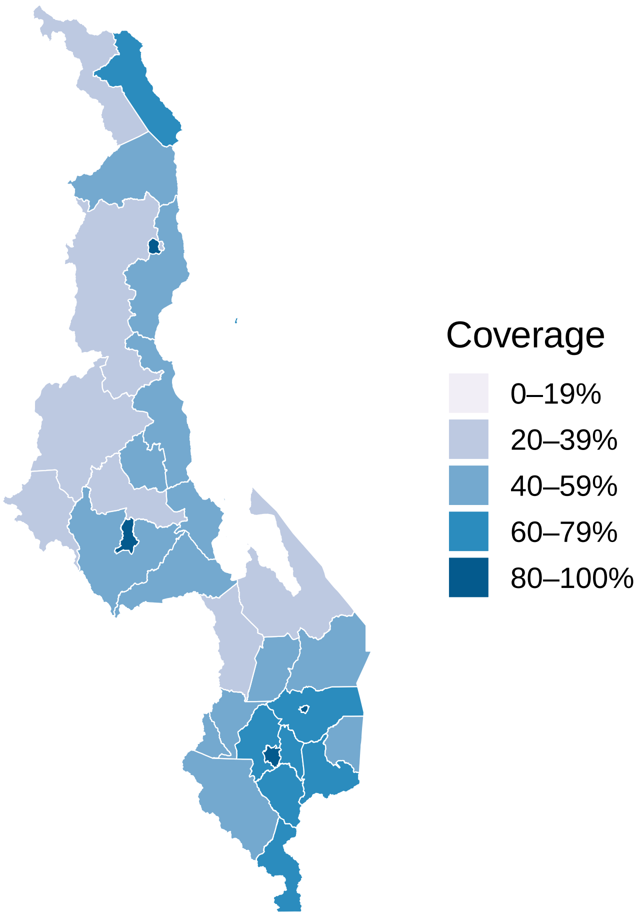

Palliative care

basic analgesics and antipyretics

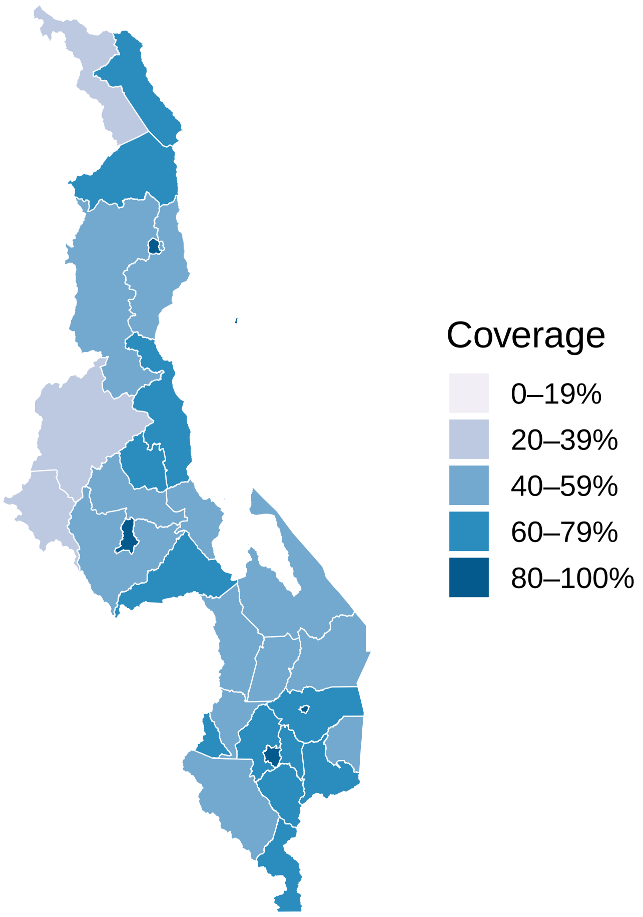

Palliative care

Pain management

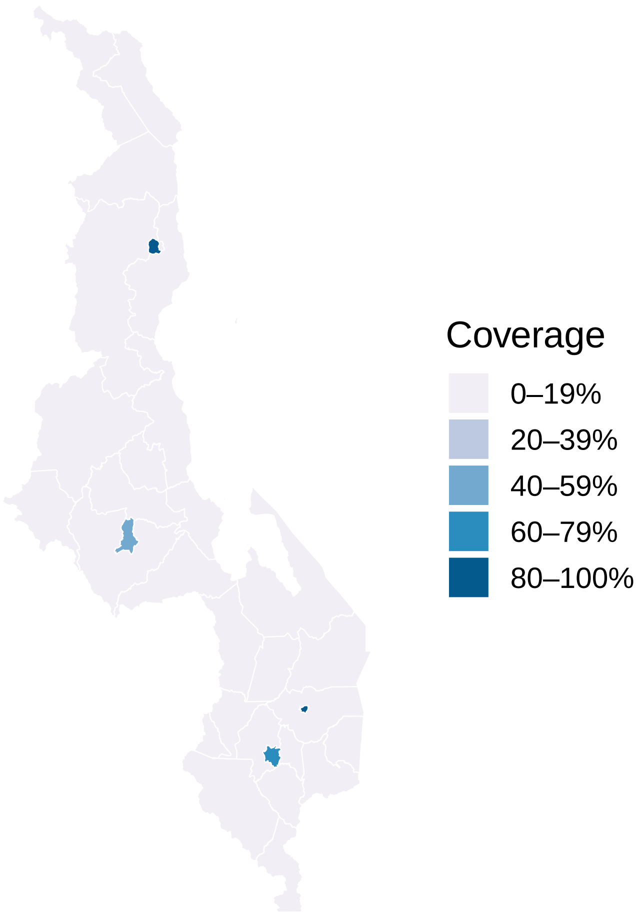

Treatment of acute malnutrition

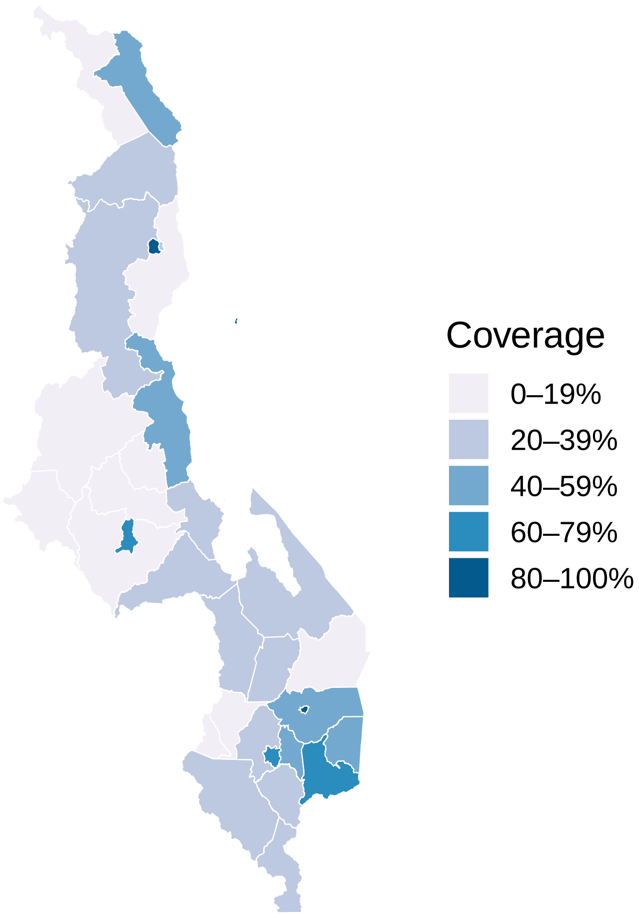

Prevention of rheumatic heart disease

Treatment of acute pharyngitis in children

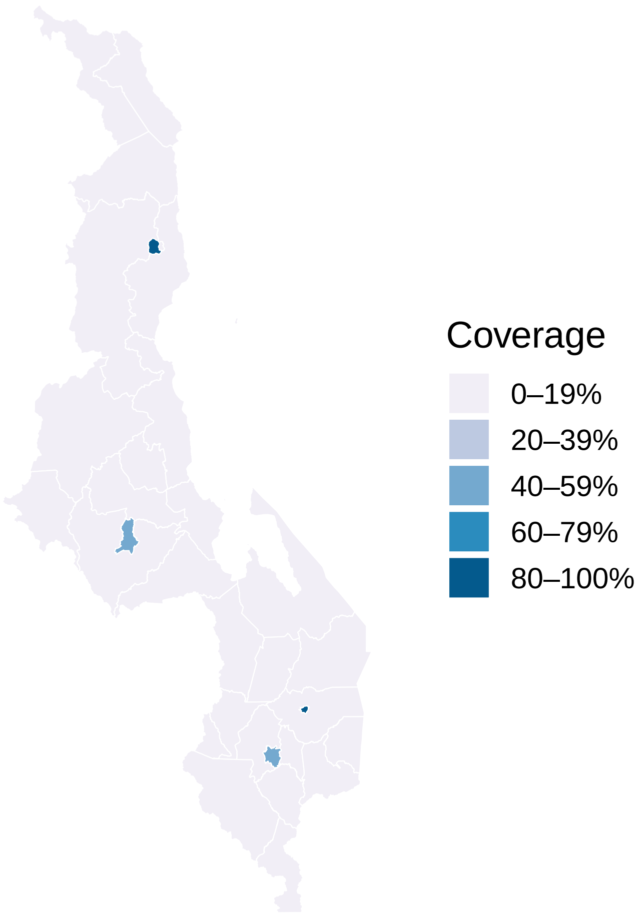

Longitudinal management of asthma

Inhalators, steroids, theophylline

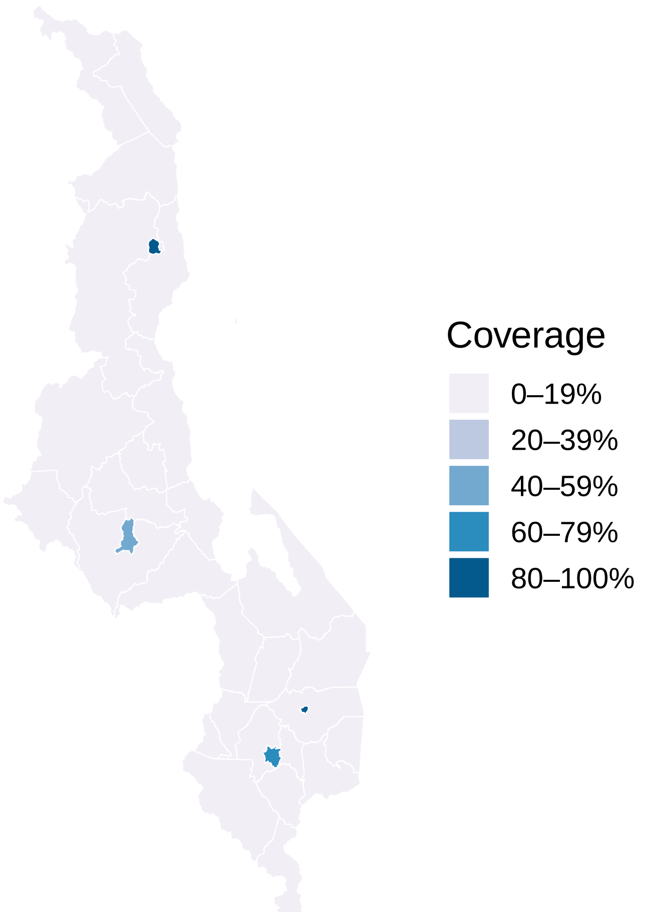

Longitudinal management of COPD

Inhalators|anticholinerg agent|Smoking cessation

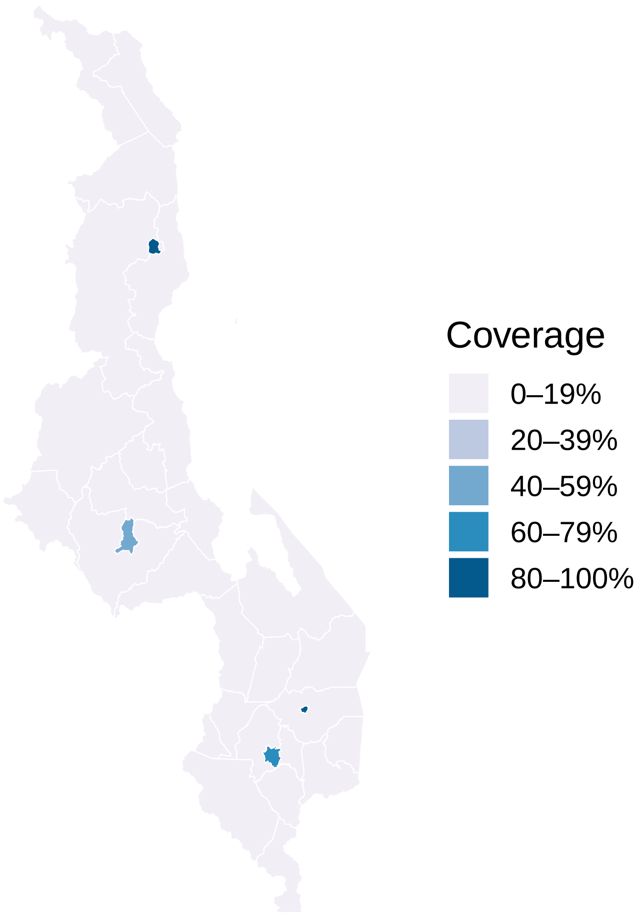

Longitudinal management of diabetes mellitus type 1

Diabetes opportunistic screening

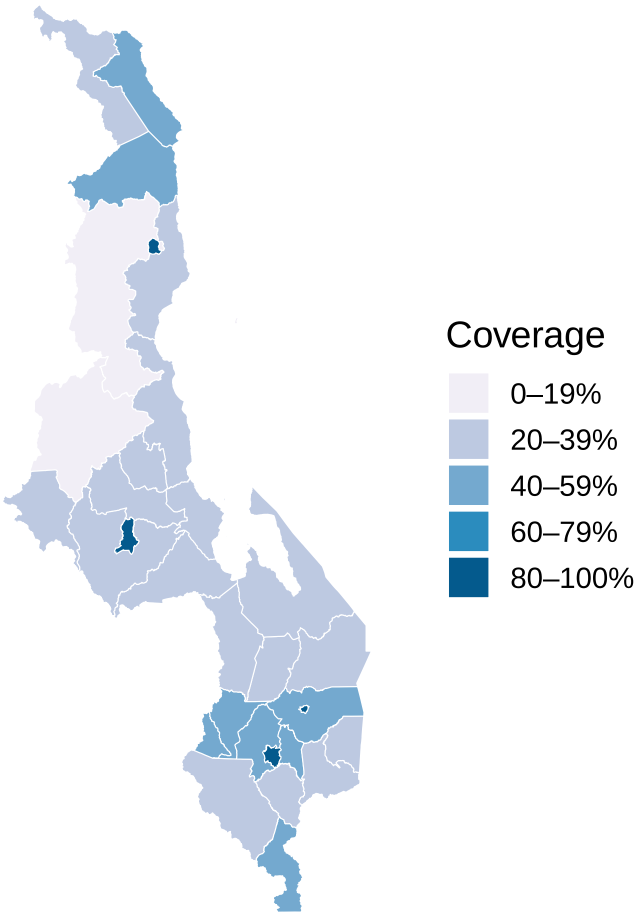

Longitudinal management of diabetes mellitus type 1

tests and Insulin

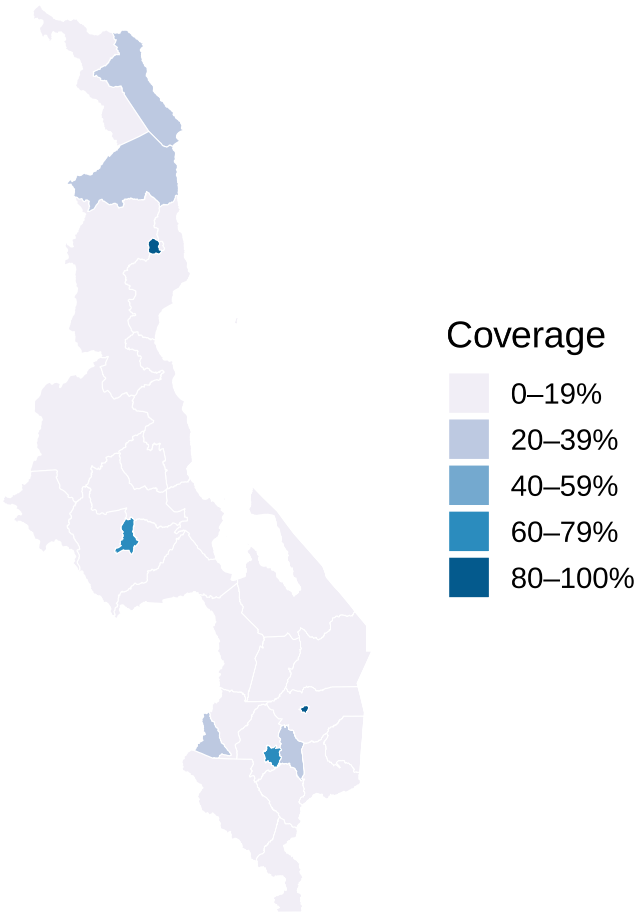

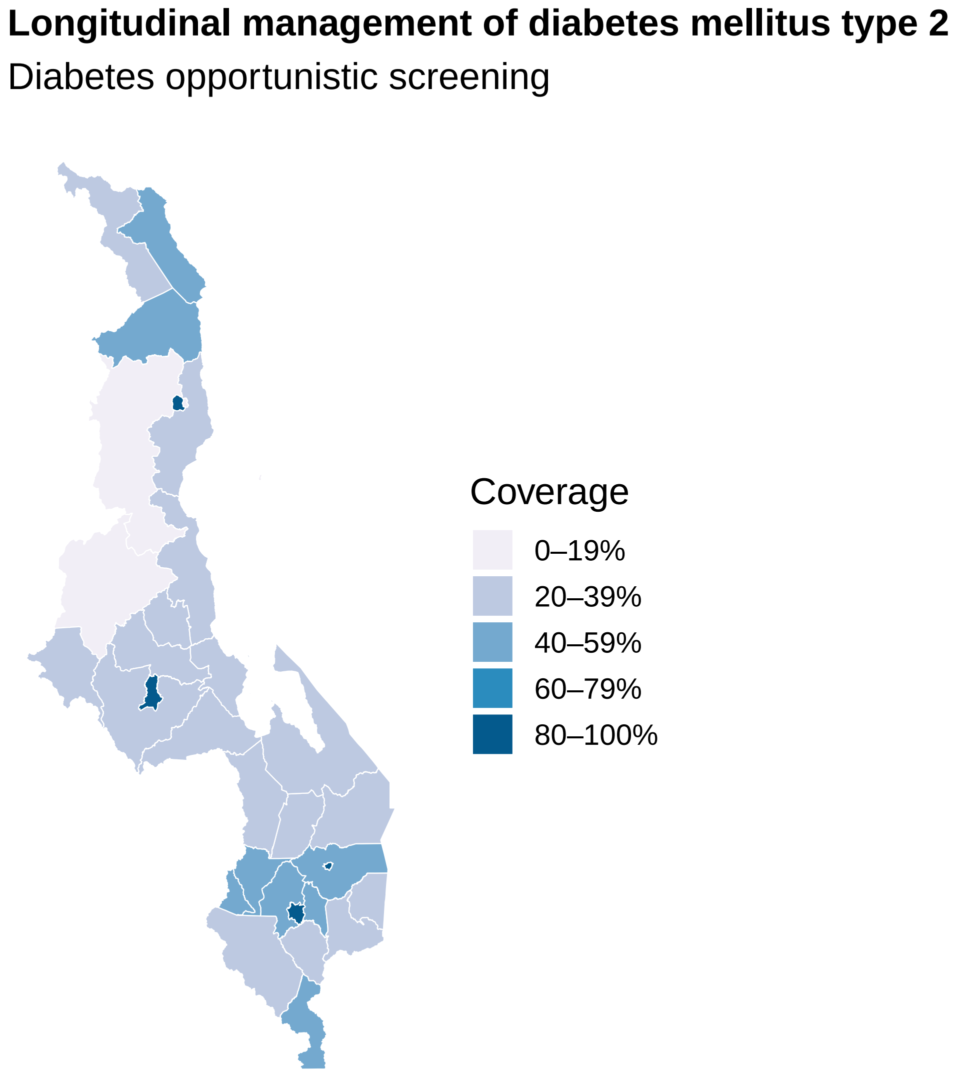

Longitudinal management of diabetes mellitus type 2
Diabetes opportunistic screening
Coverage
0–19%
20–39%
40–59%
60–79%
80–100%

Longitudinal management of diabetes mellitus type 2

Antidiabetic drugs and insulin

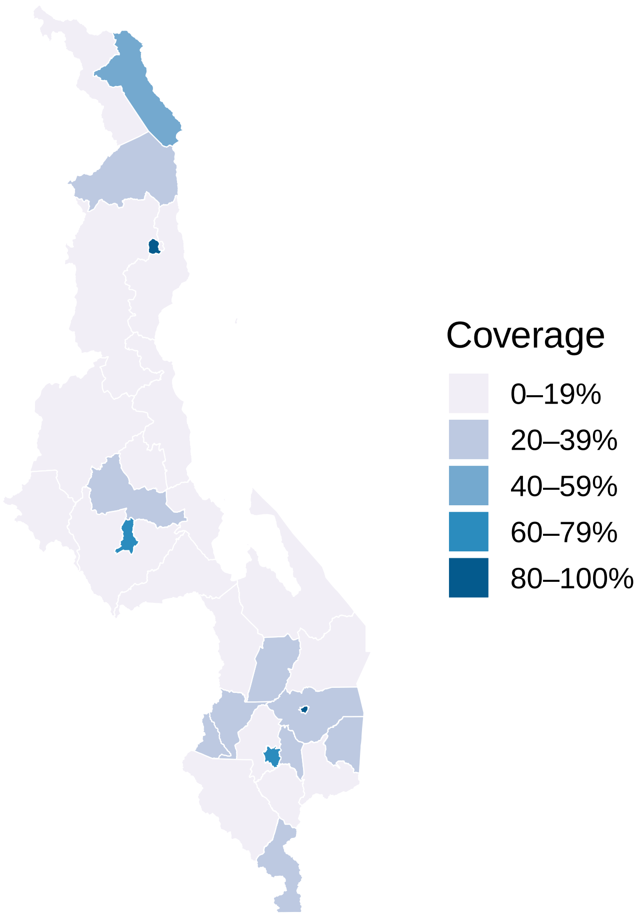

Management of migraine

Basic management of migraine

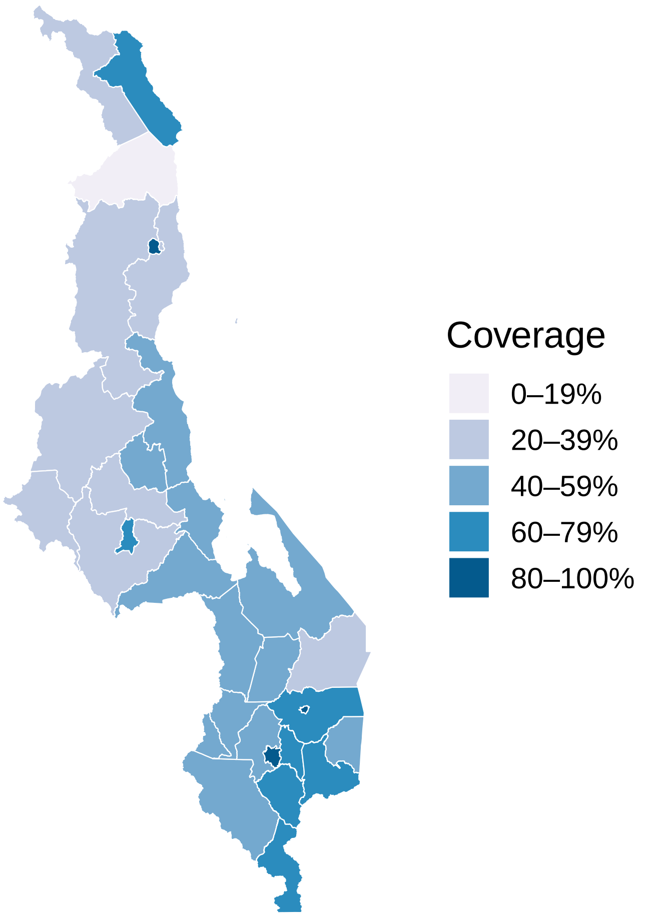

Family planning

at least two methods available

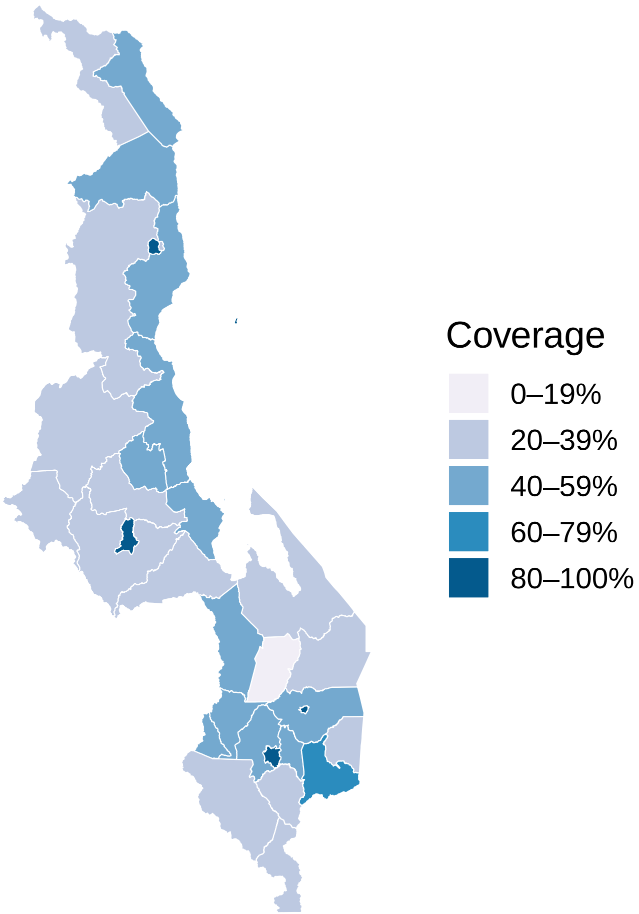

Antenatal care

Uneventful pregnancy

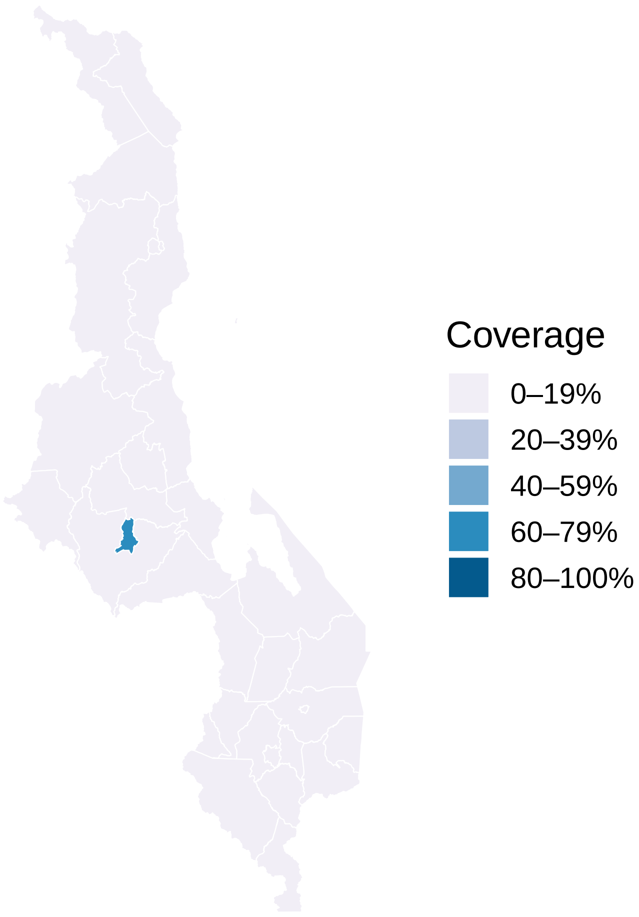

Antenatal care

Prevention of mother to child HIV transmission (PMTCT, option B+)

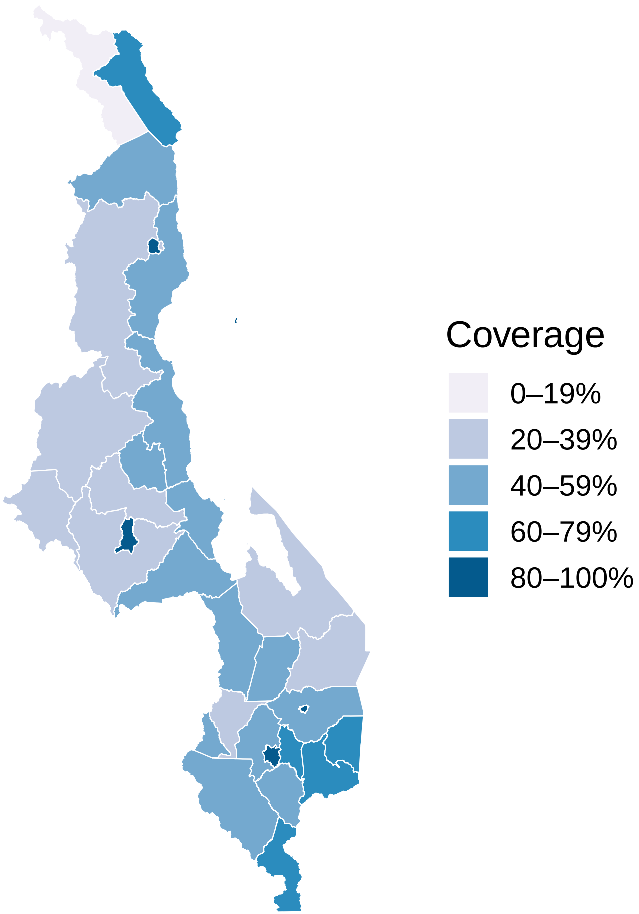

Safe delivery and management of labour complications

Safe delivery

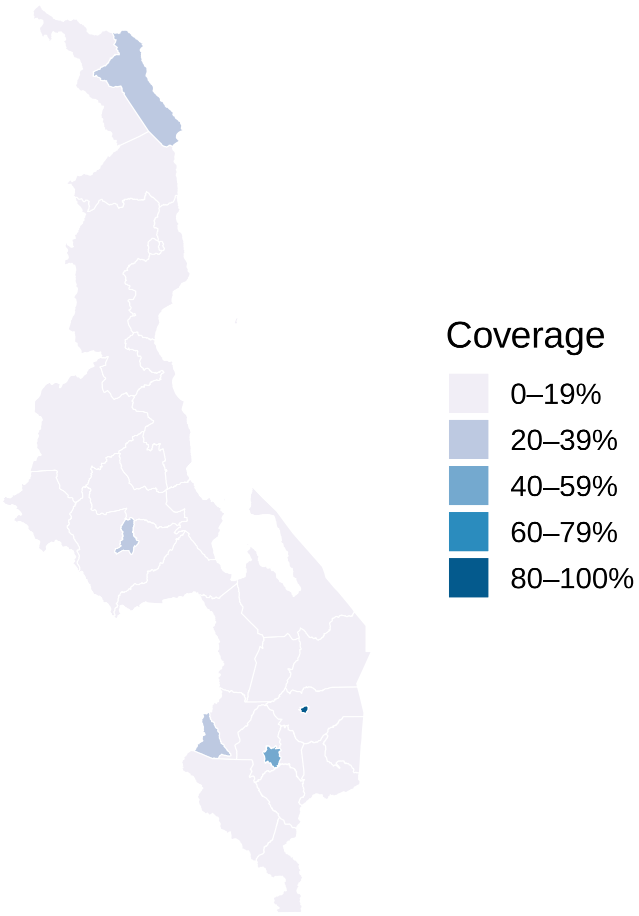

Safe delivery and management of labour complications

Safe delivery

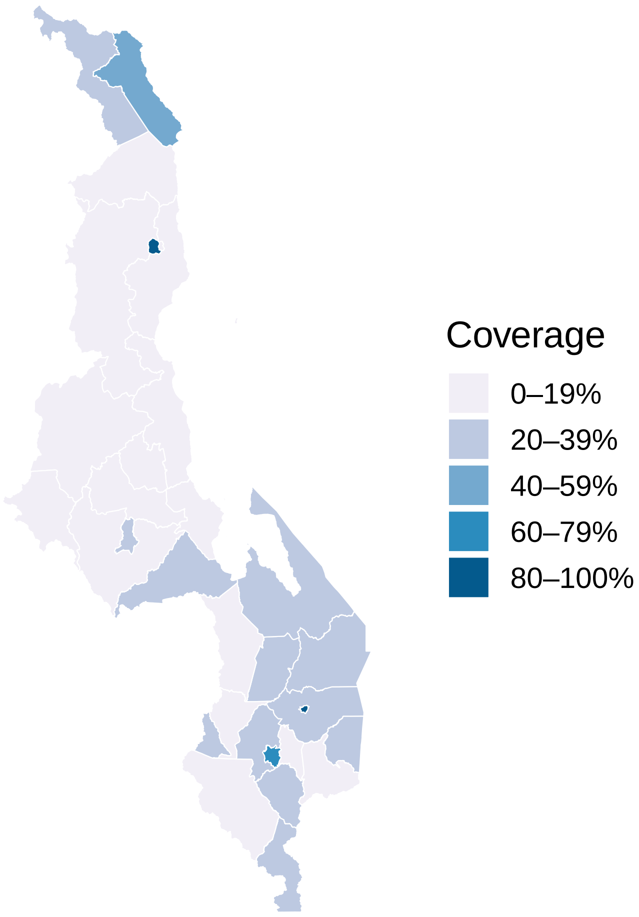

Safe delivery and management of labour complications

Safe delivery

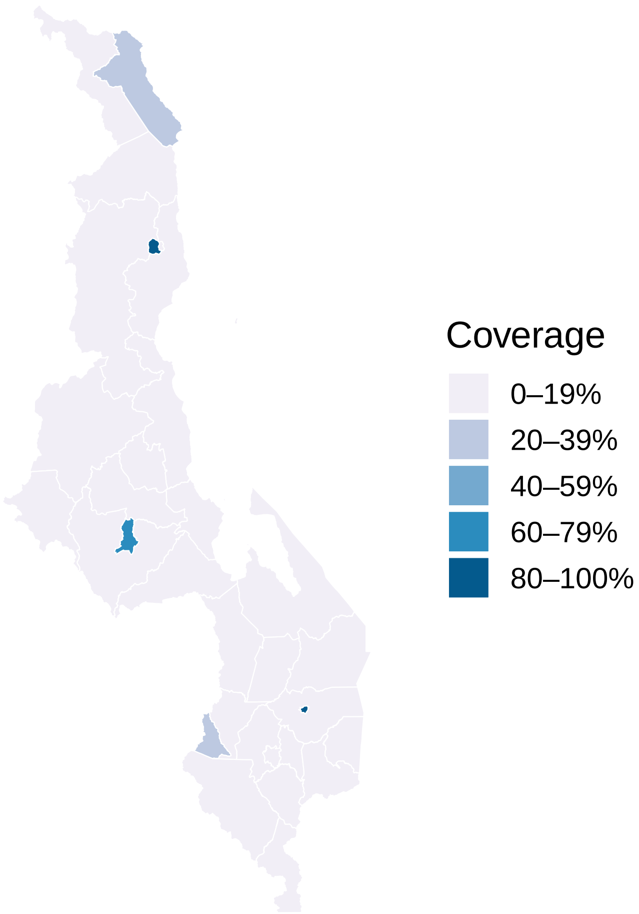

Safe delivery and management of labour complications

Management of maternal sepsis

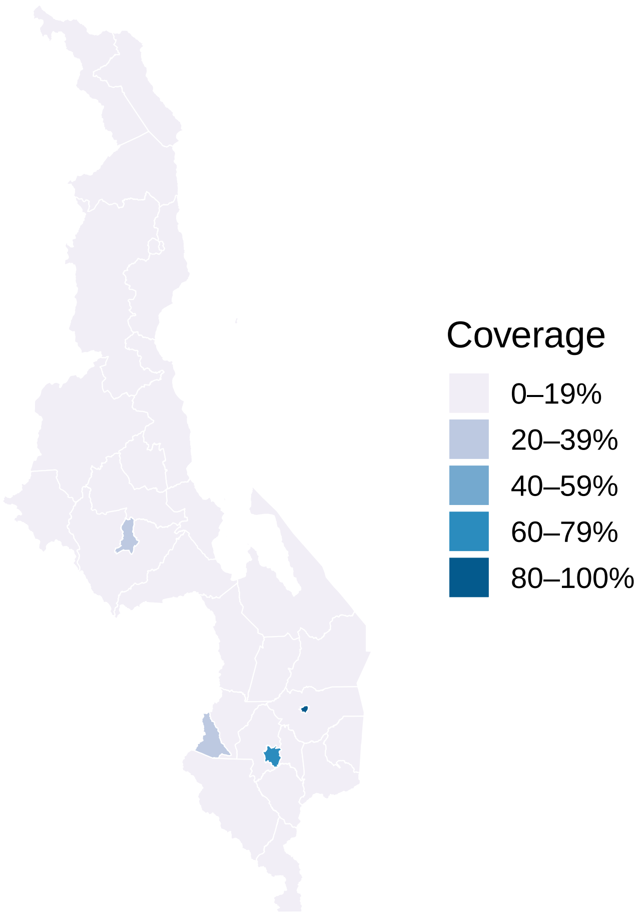

Routine care for postpartum women

Counselling on family planning and contraception

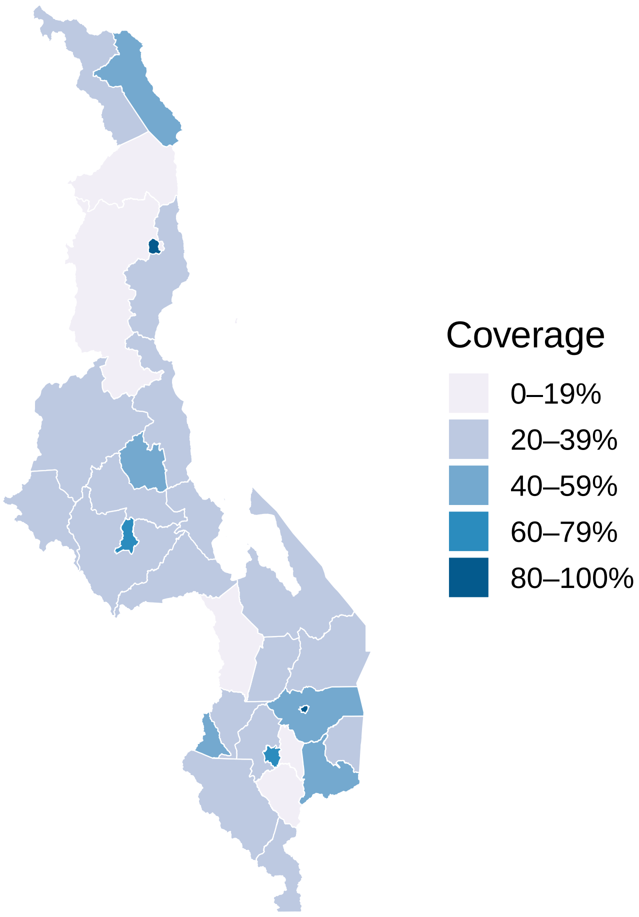

Routine care for postpartum women

Treatment of lactational mastitis

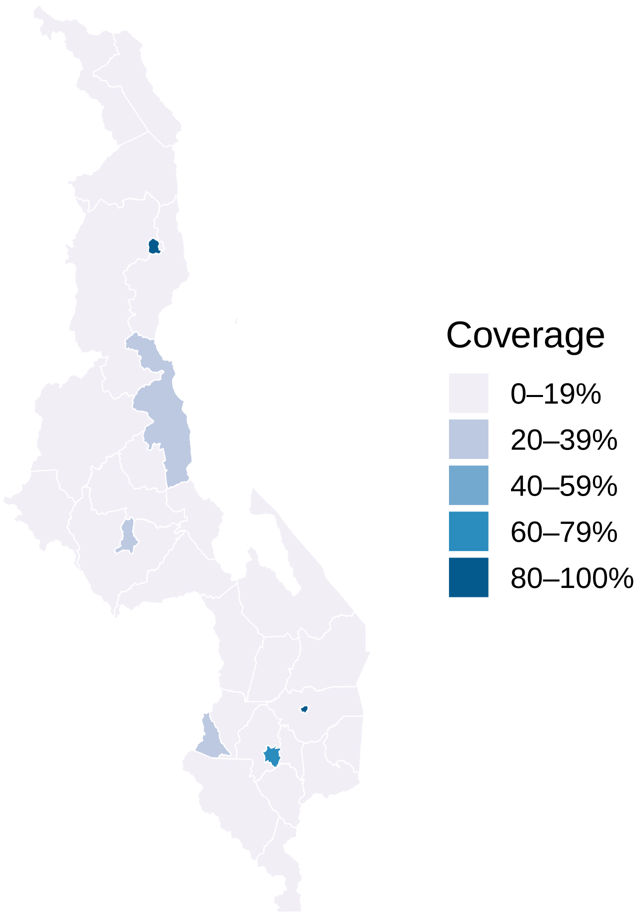

Early care for newborn

Treatment of local infections (eye, skin)

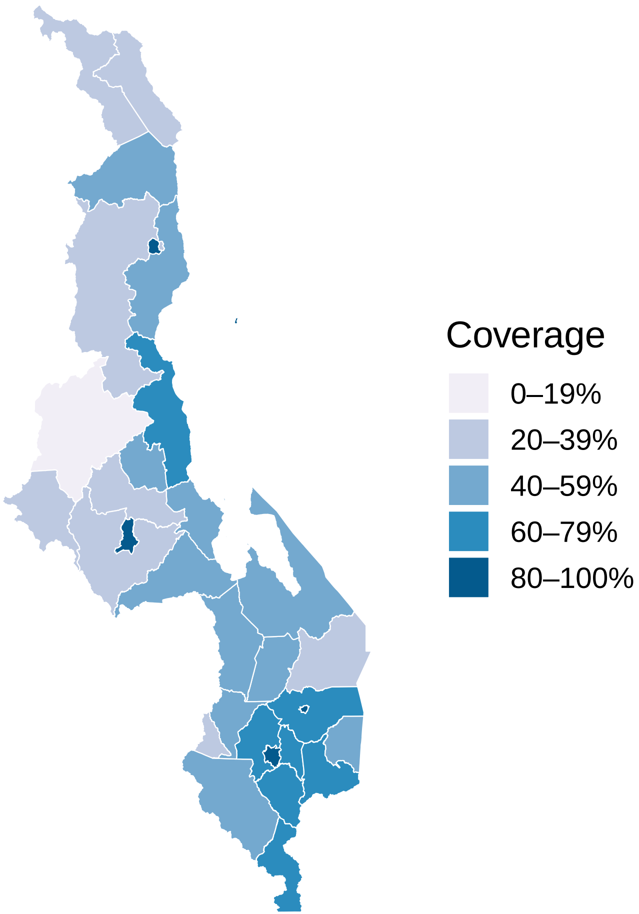

Early care for newborn

Hygienic cord care (chlorhexidine and tetracycline ointment)

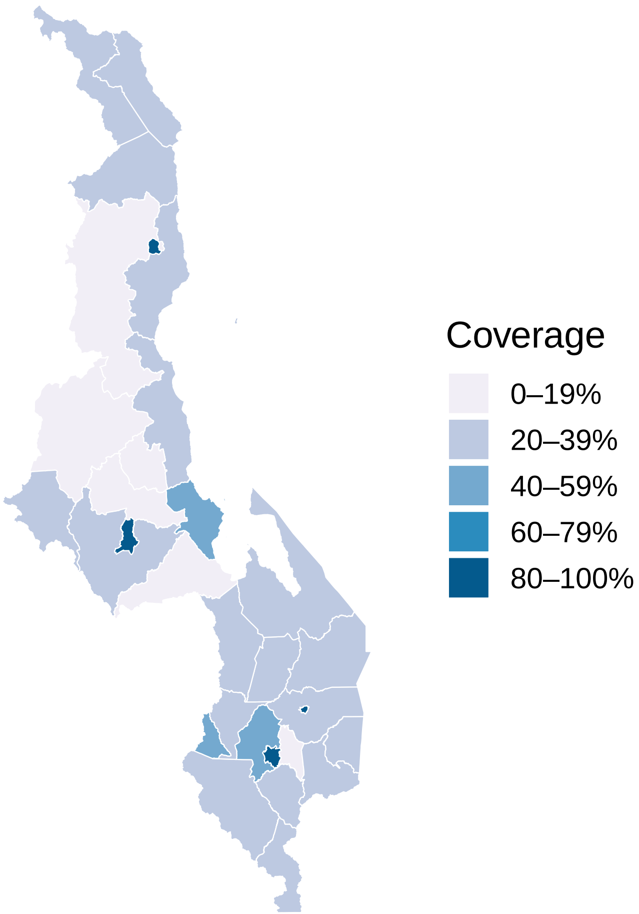

Management of wounds (excluding burns)

Drainage of superficial abscess

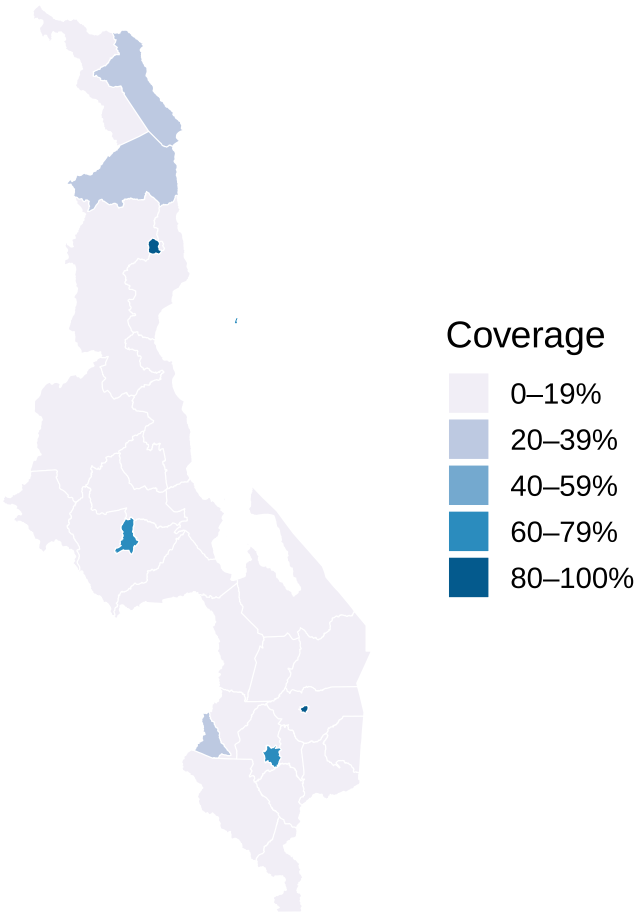

Management of wounds (excluding burns)

Suturing laceration

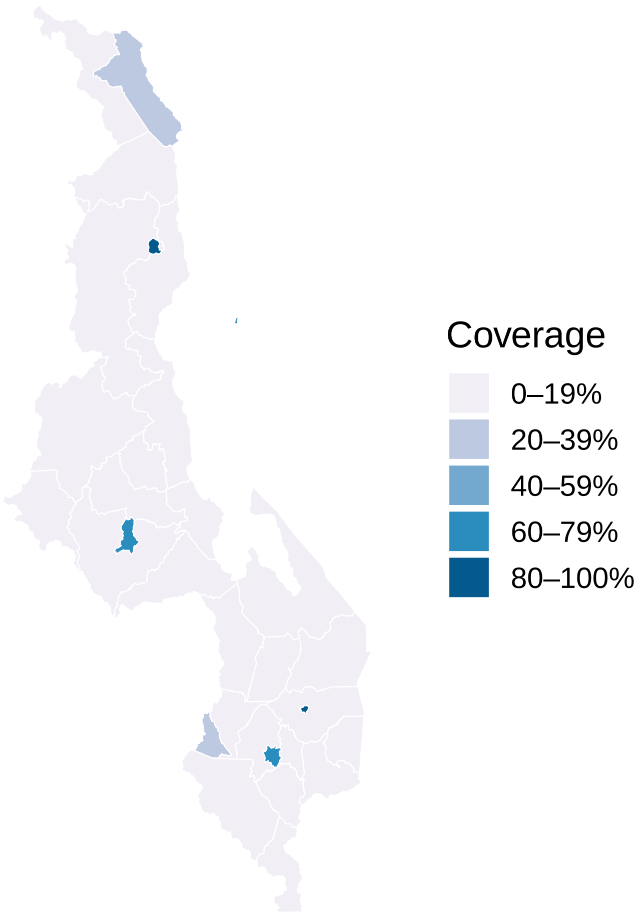

Management of extremity injuries

Management of upper extremity fractures

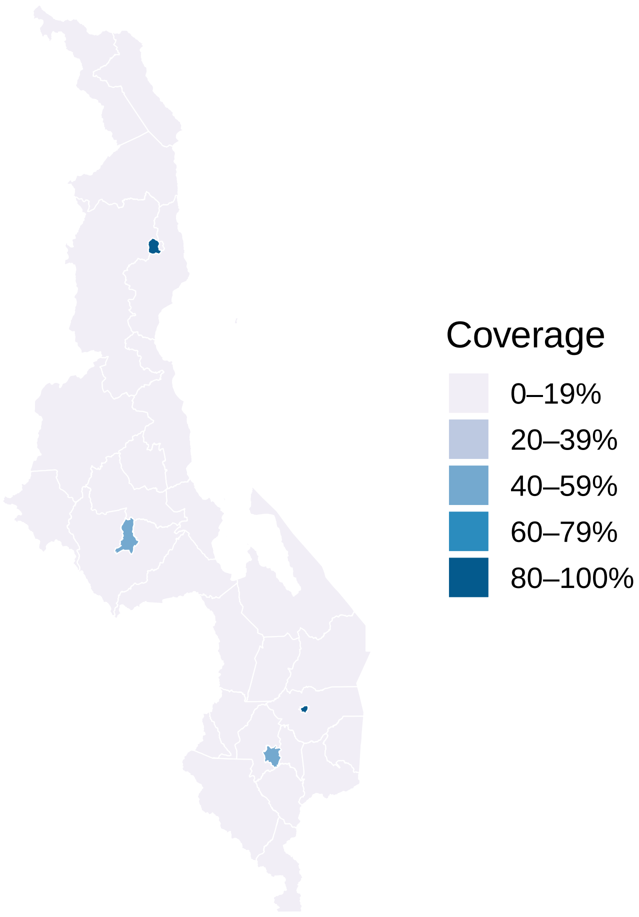

Management of extremity injuries

Management of lower extremity injuries

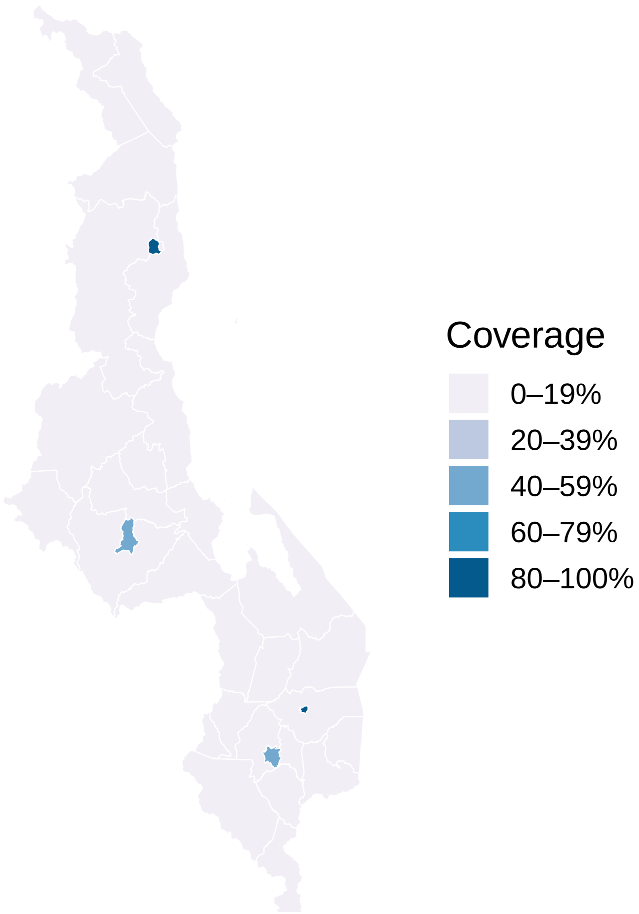

District level coverage
Advanced Primary Health Care

Management of HIV

Management of opportunistic infections associated with HIV|AIDS

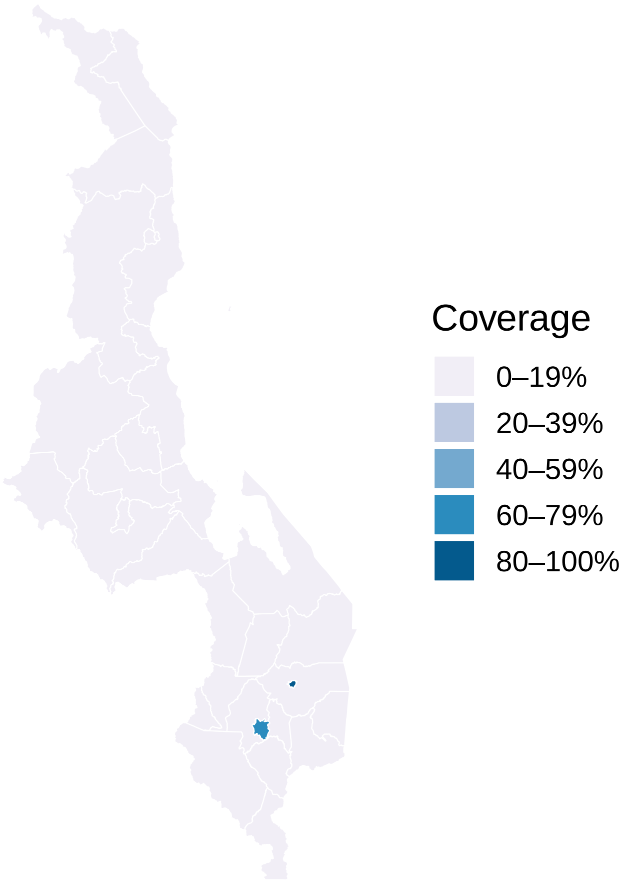

Management of malaria

Comprehensive treatment of severe malaria_1

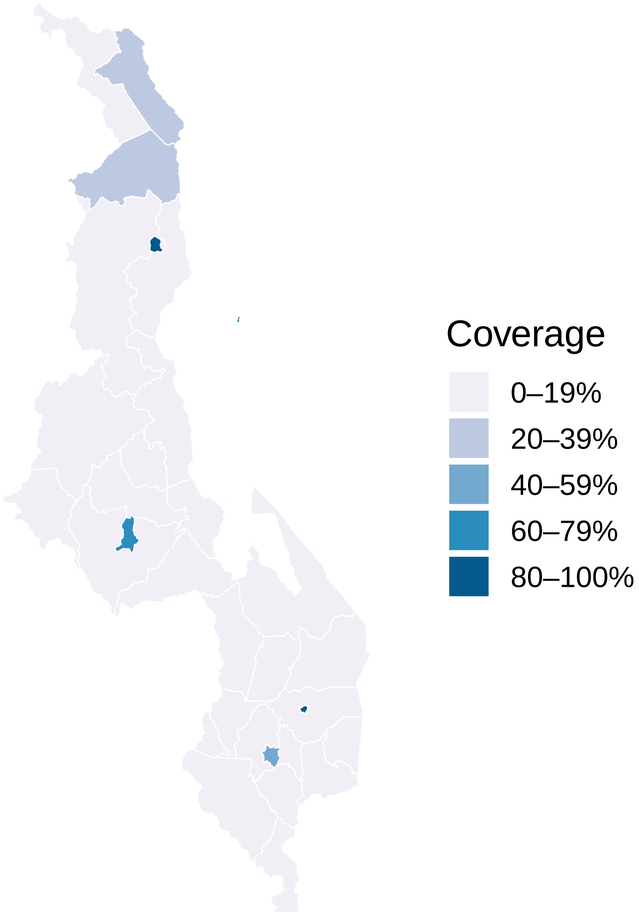

Management of malaria_2

Comprehensive treatment of severe malaria_2

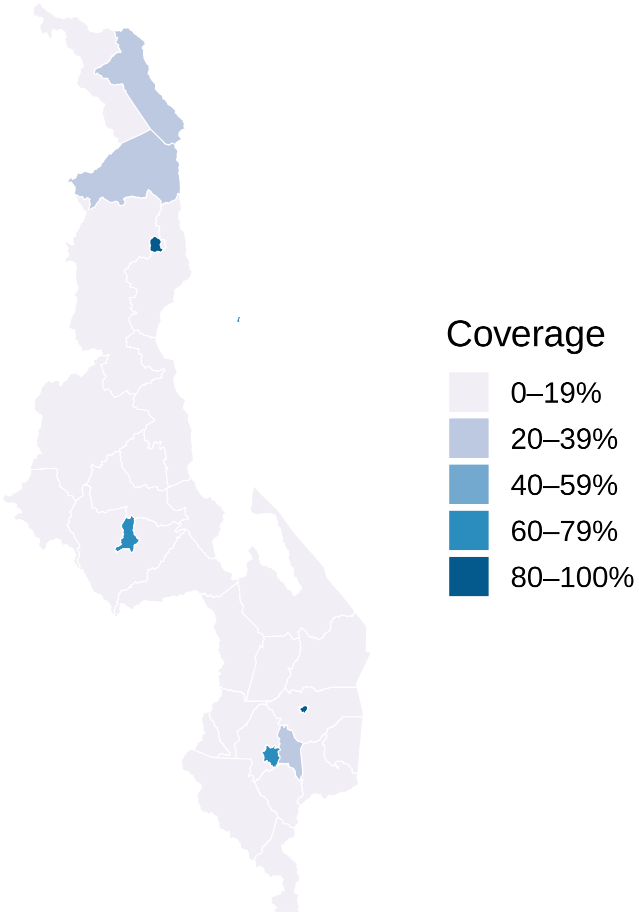

Treatment of PID (Pelvic Inflammatory Disease)

Treatment of PID (Pelvic Inflammatory Disease)

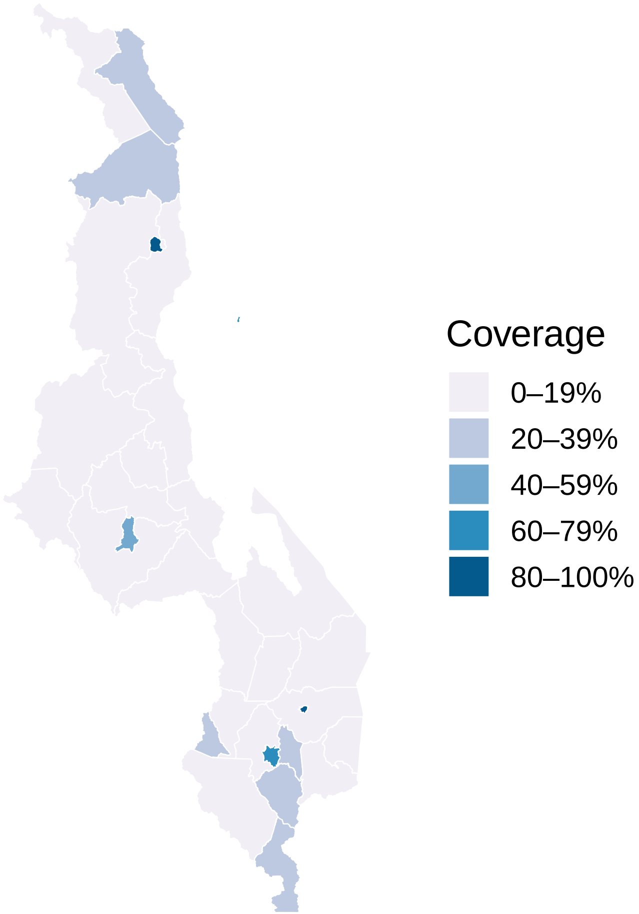

Treatment of acute lower respiratory infections, children

Pneumonia (severe), IV antibiotics

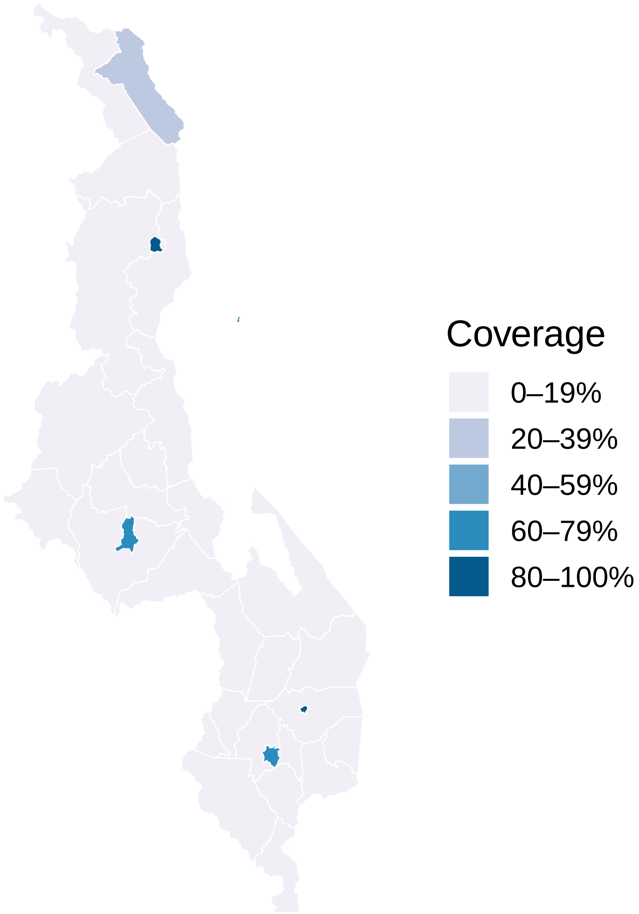

Treatment of acute lower respiratory infections, adults

Pneumonia (severe), IV antibiotics adults

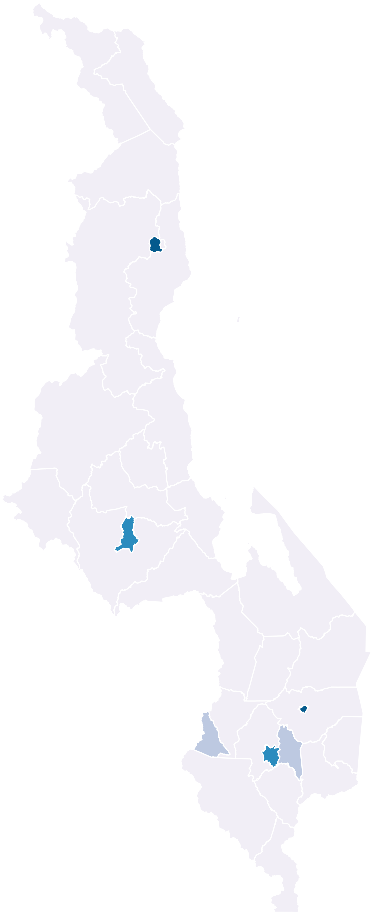

Coverage

	0–19%
	20–39%
	40–59%
	60–79%
	80–100%

Palliative care

IV morphine

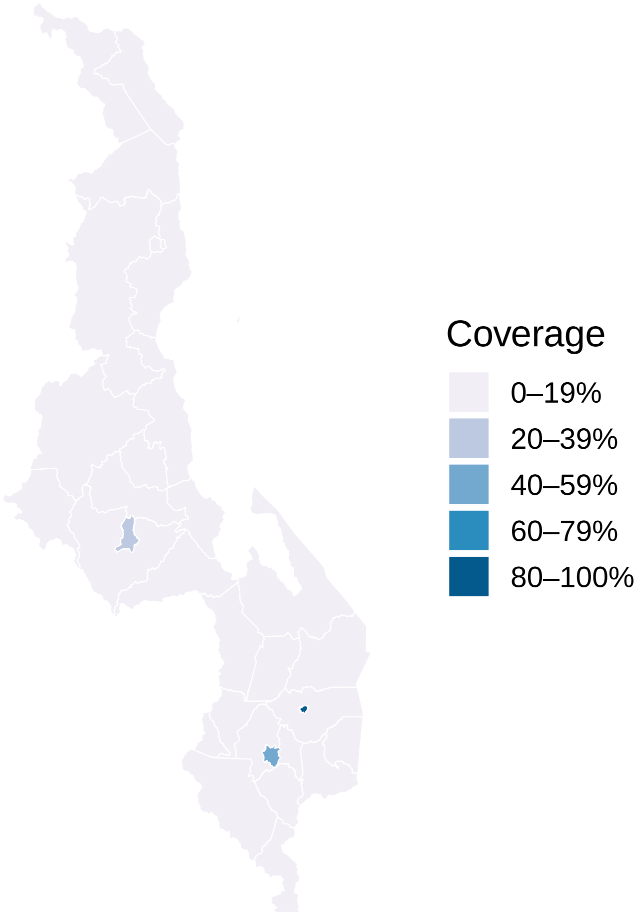

Palliative care

Other palliative needs

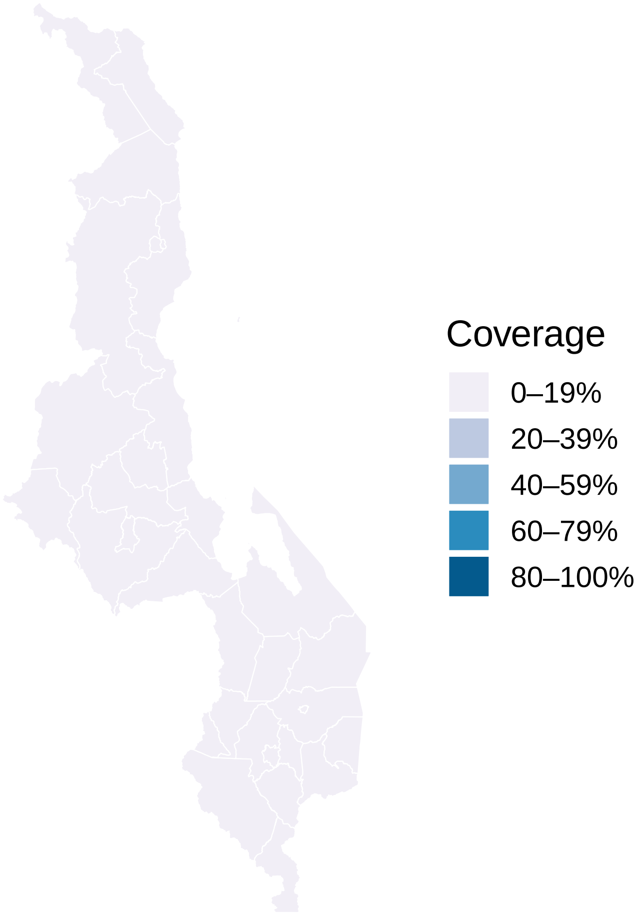

Initial management of sepsis

Initial management of sepsis

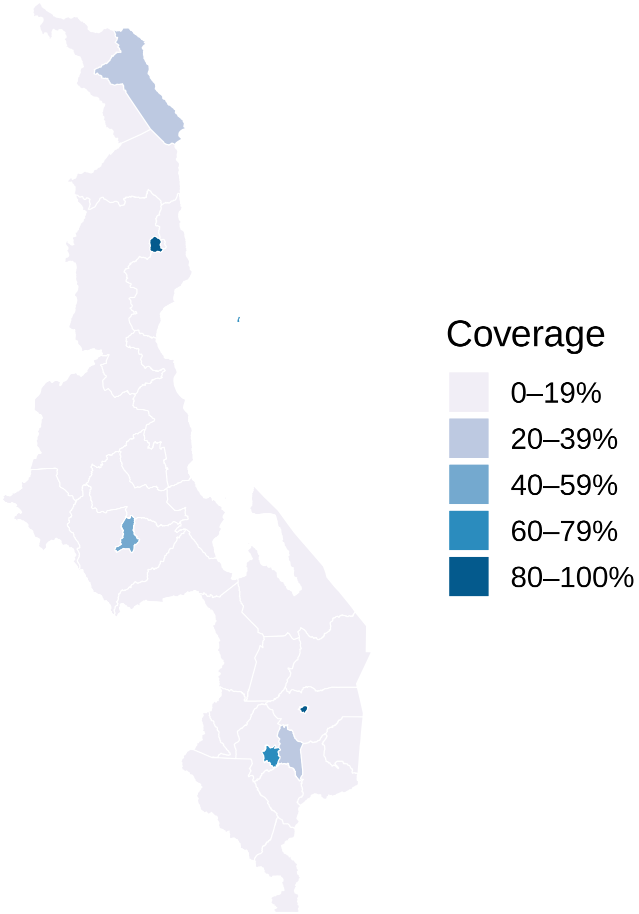

Emergency care, advanced

Resuscitation with advanced life support measures

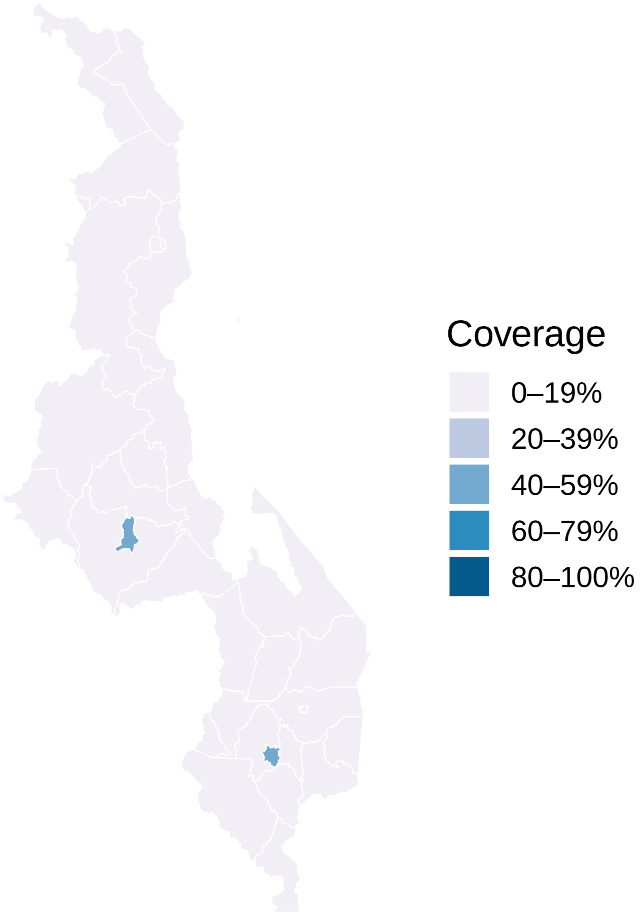

Treatment of severe acute malnutrition

Treatment of severe acute malnutrition

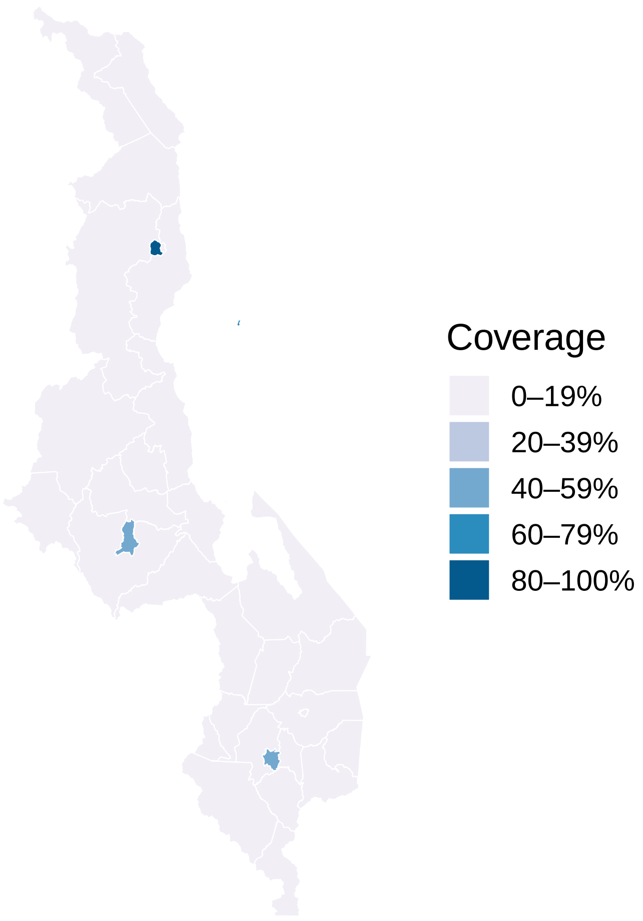

Primary prevention with absolute CVD risk

CVD targeted screening at facility & Primary CVD prevention optimal − absolute CVD risk > 10% (antihypertensives, statins)

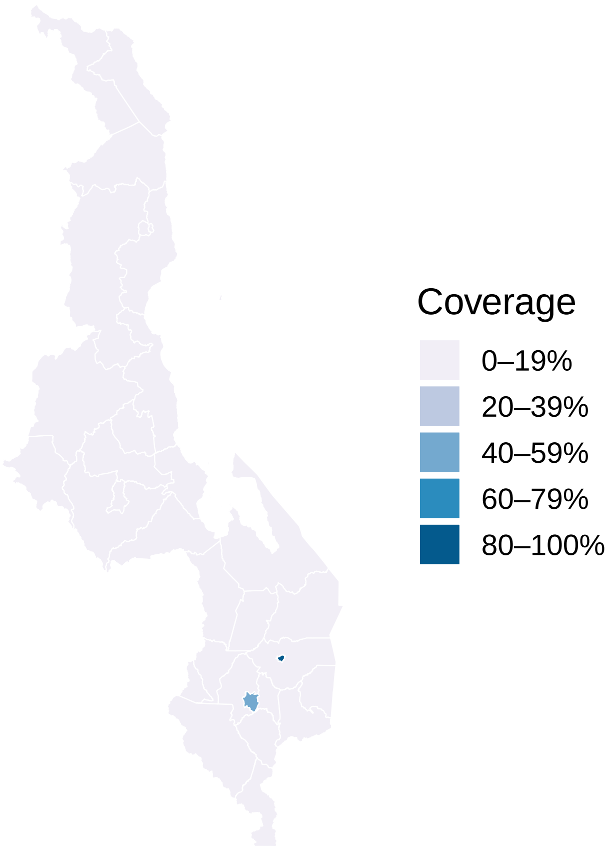

Management of acute ischemic heart disease

IHD basic: Aspirin for all cases of high−risk chest pain

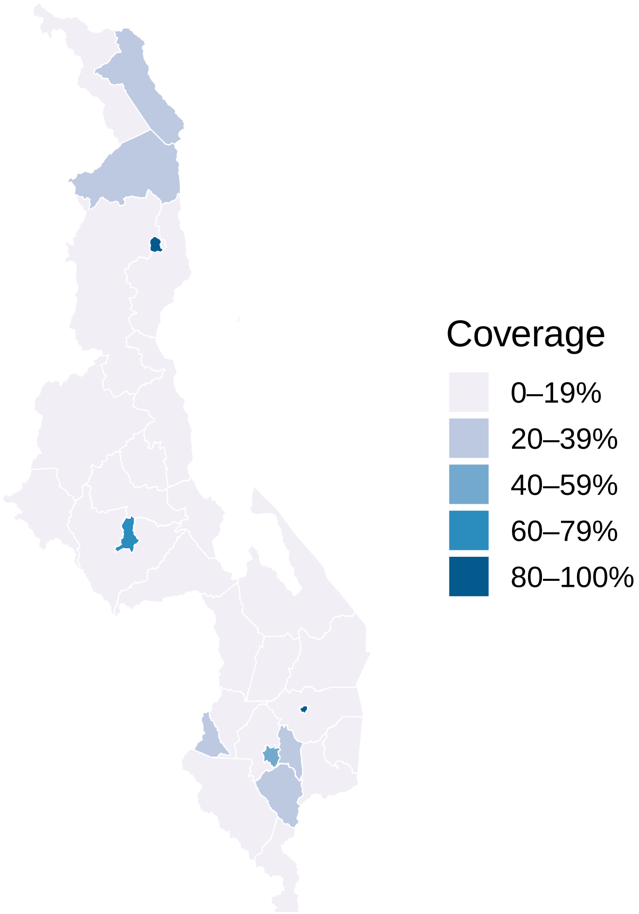

Secondary prevention of ischemic heart disease

aspirin, beta blockers, ACE inhibitors, ARB, statins

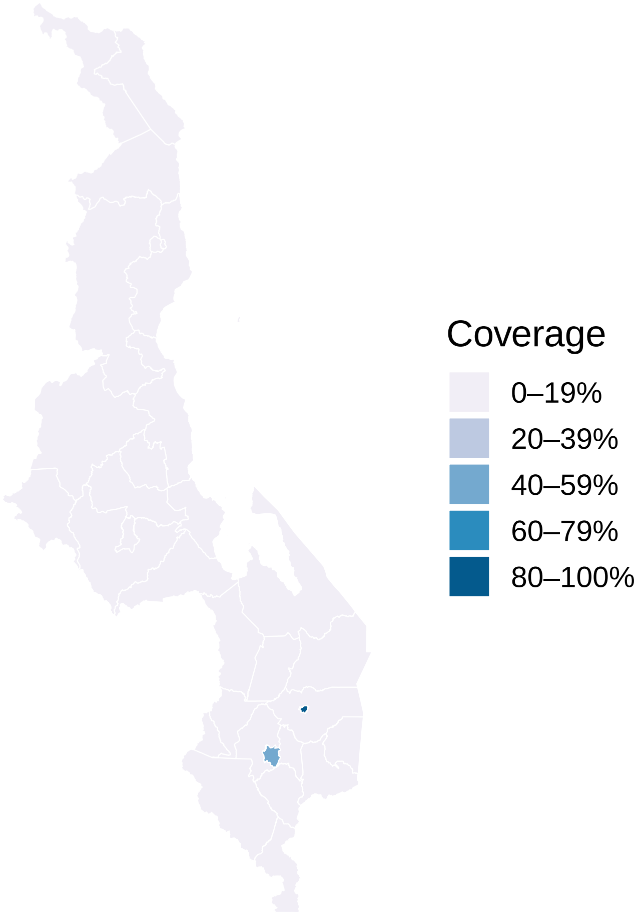

Longitudinal management of heart failure

Management of chronic heart failure with diuretics, beta−blockers, ACE inhibitors, and mineralocorticoid antagonists

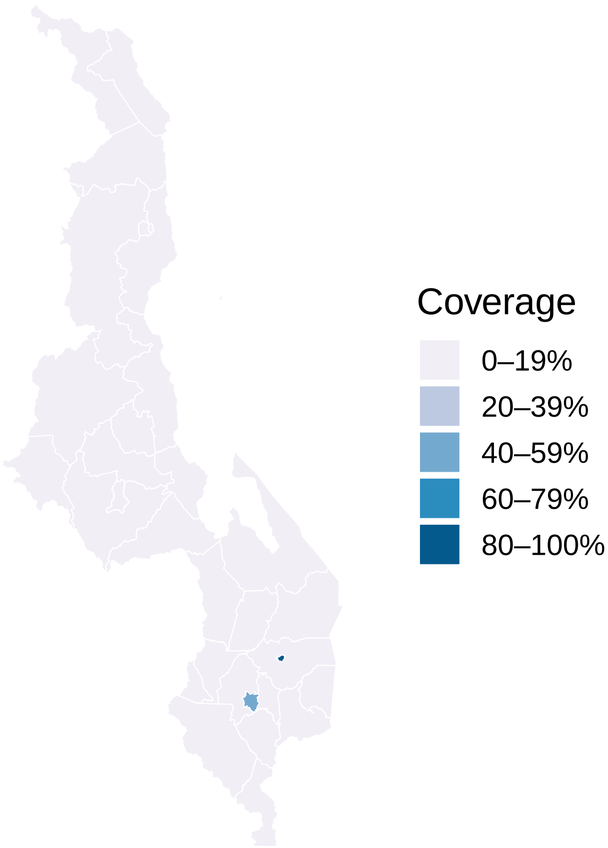

Prevention of rheumatic heart disease

RHD secondary prevention

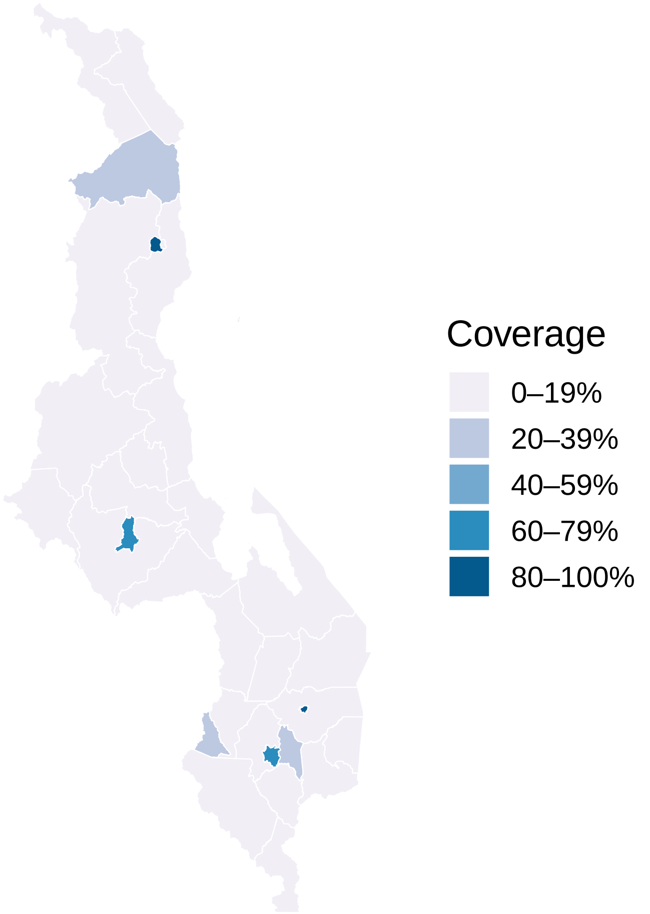

Secondary prevention of peripheral vascular disease (aspirin, beta blockers, ACE inhibitors, ARB, statins)

Secondary prevention of peripheral vascular disease (aspirin, beta blockers, ACE inhibitors, ARB, statins)

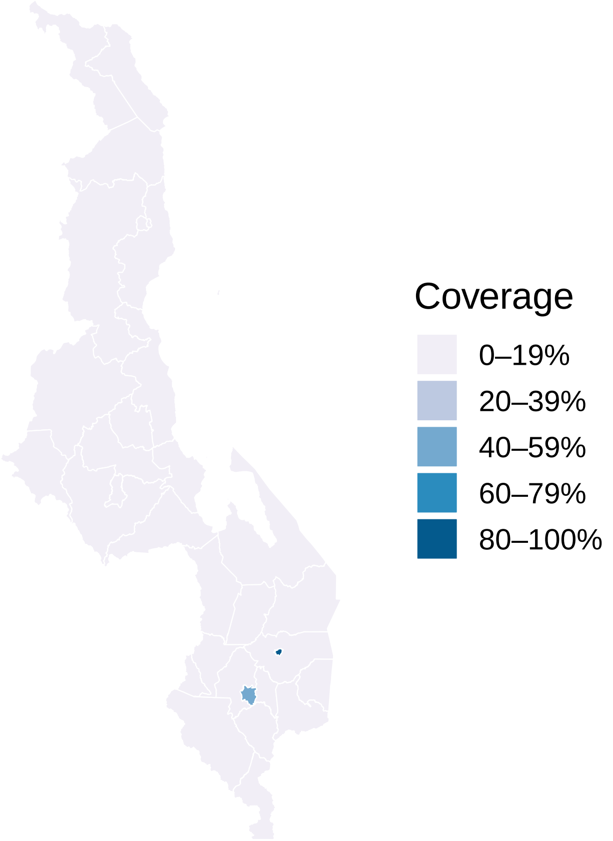

Secondary prevention of stroke

Secondary prevention of ischemic | hemorrhagic stroke stroke (aspirin, beta blockers, ACE inhibitors, ARB, statins)

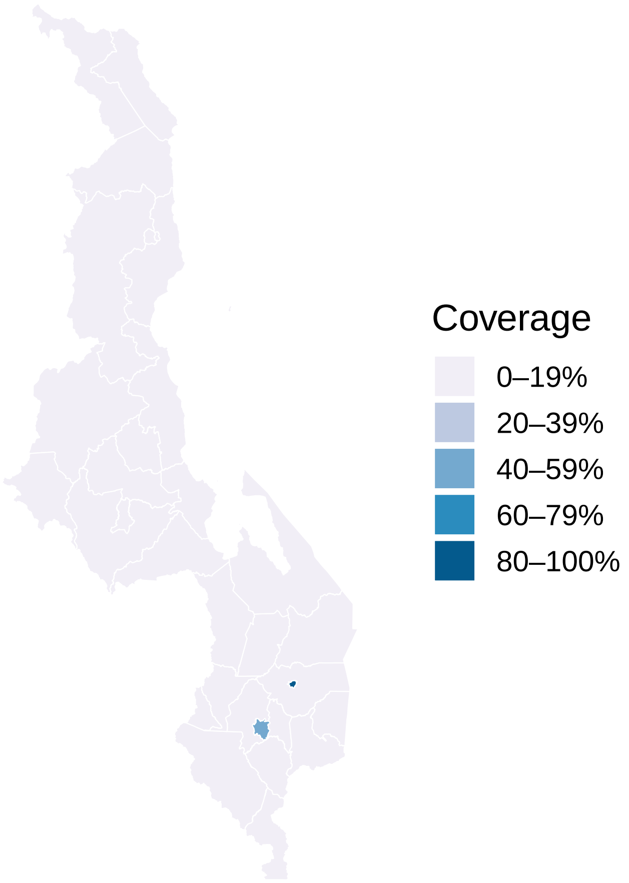

Treatment of acute exacerbation of asthma

Treatment of acute exacerbation of asthma (Severe)

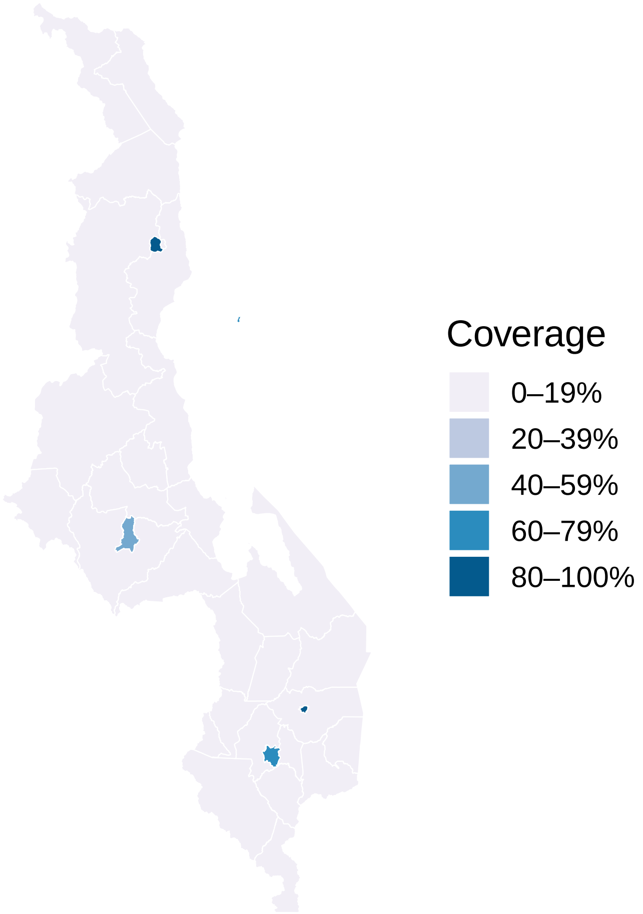

Treatment of acute exacerbation of asthma

Treatment of acute exacerbation of asthma (Moderate)

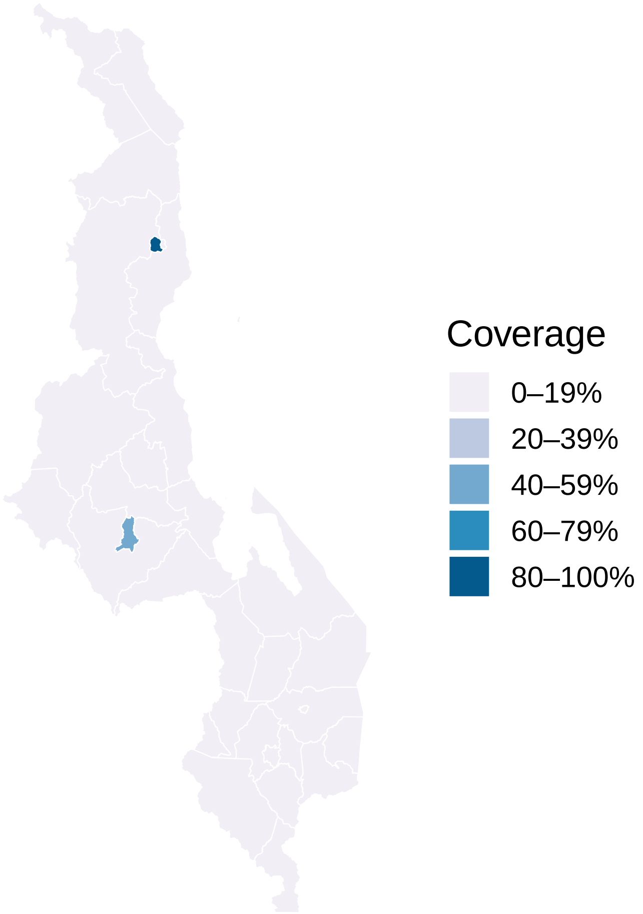

Treatment of acute exacerbation of COPD

Treatment of acute exacerbation of COPD (Severe)

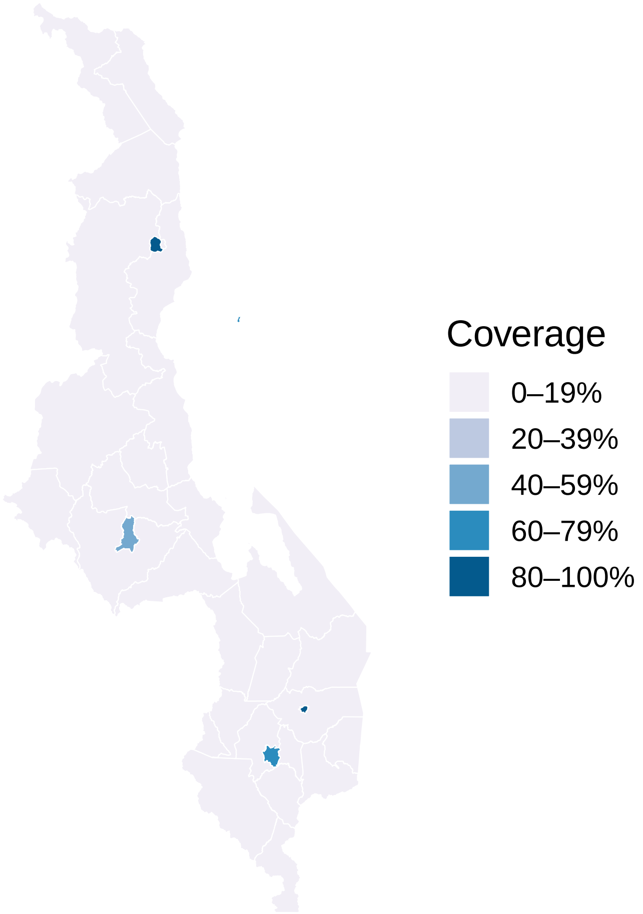

Treatment of acute exacerbation of COPD

Management of acute exacerbation of COPD (Moderate)

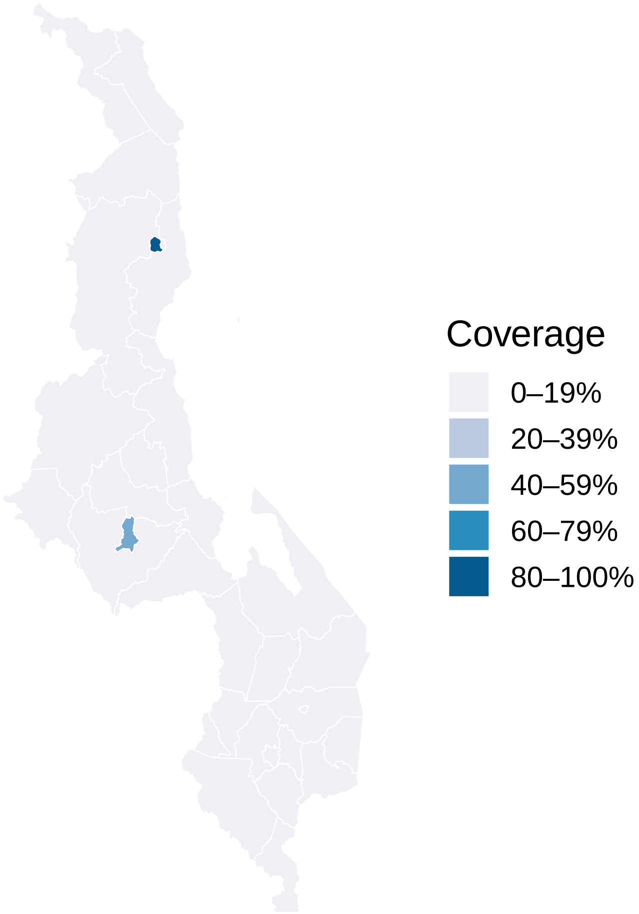

Treatment of acute hypoglycaemia

Treatment of acute hypoglycaemia

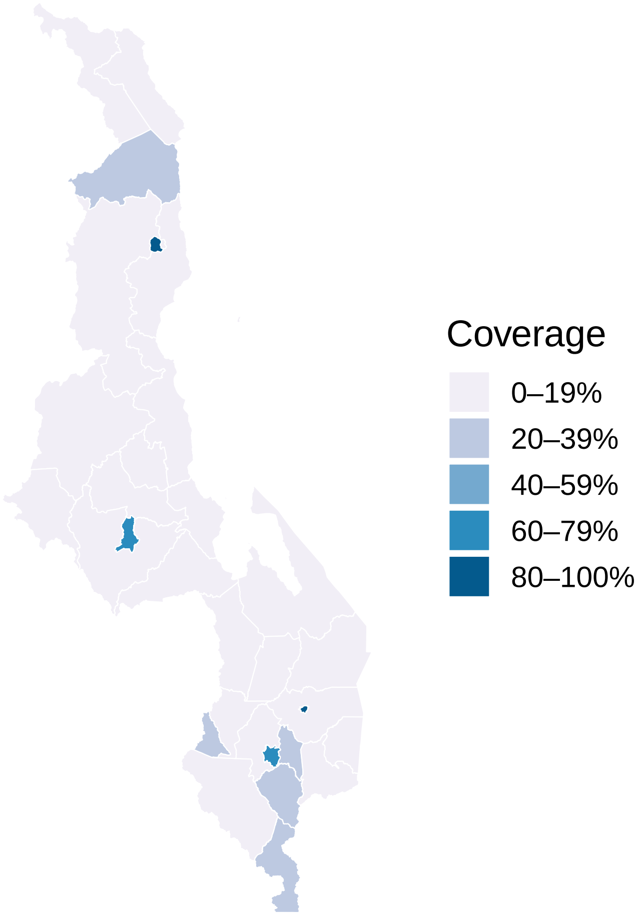

Treatment of acute hyperglycaemia

Treatment of acute hyperglycaemia

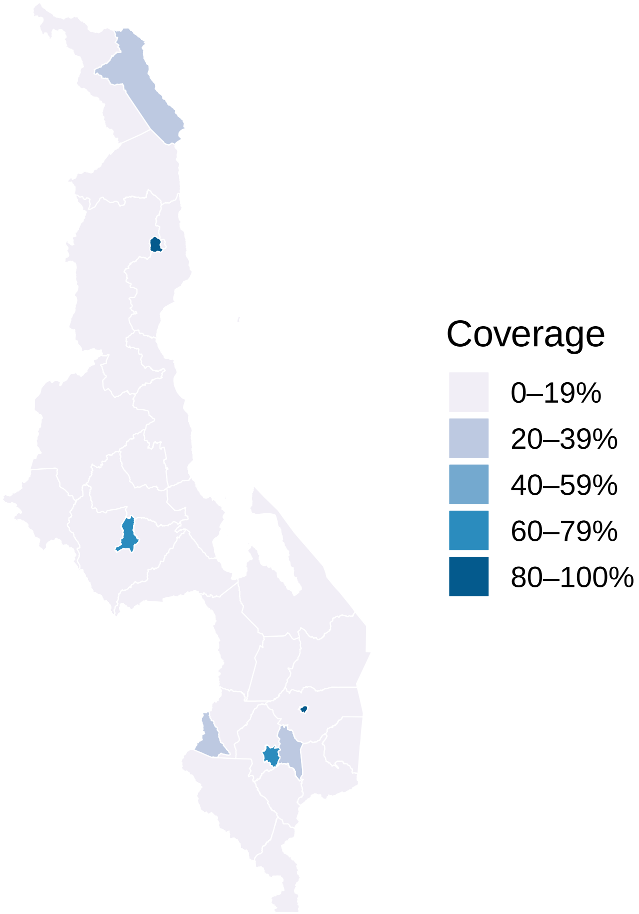

Management of anxiety disorders

Management of anxiety disorders

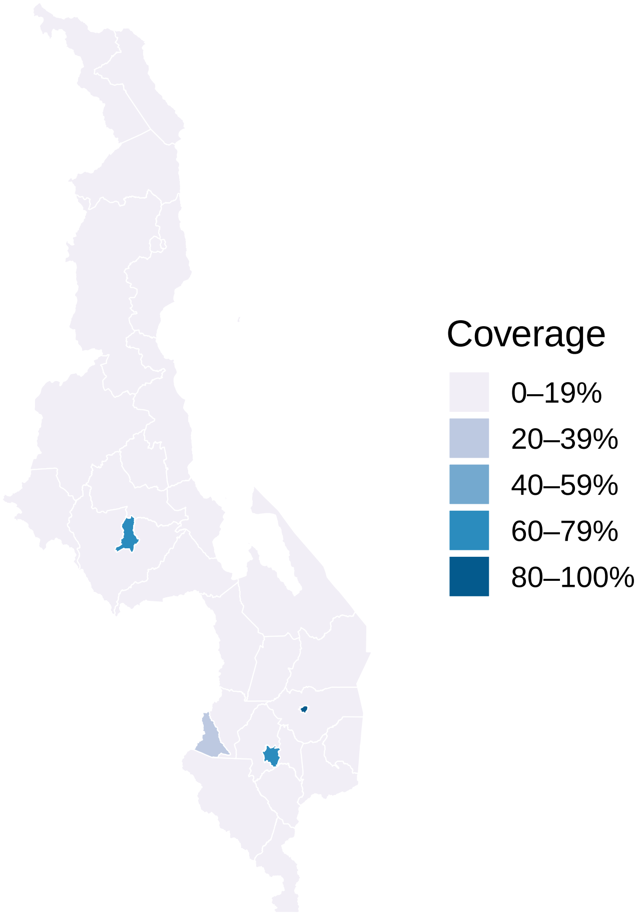

Management of depression

Management of depression

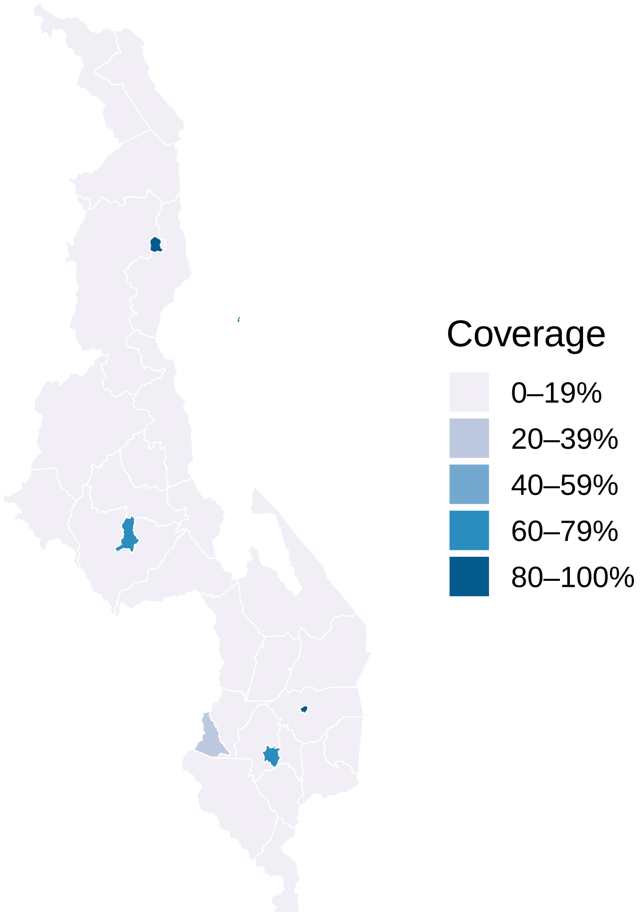

Management of psychotic disorders

Management of psychotic disorders

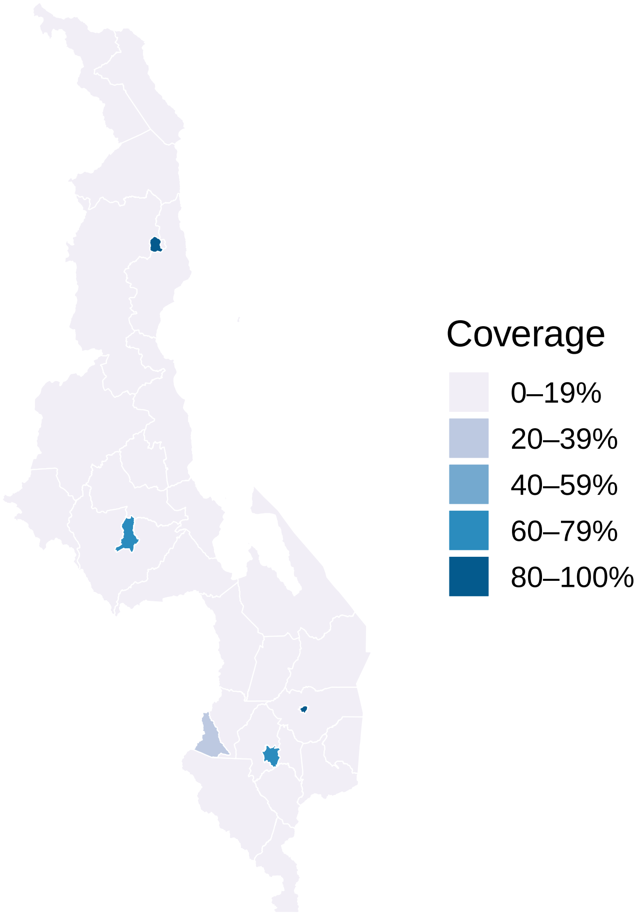

Bipolar disorders

Bipolar disorders

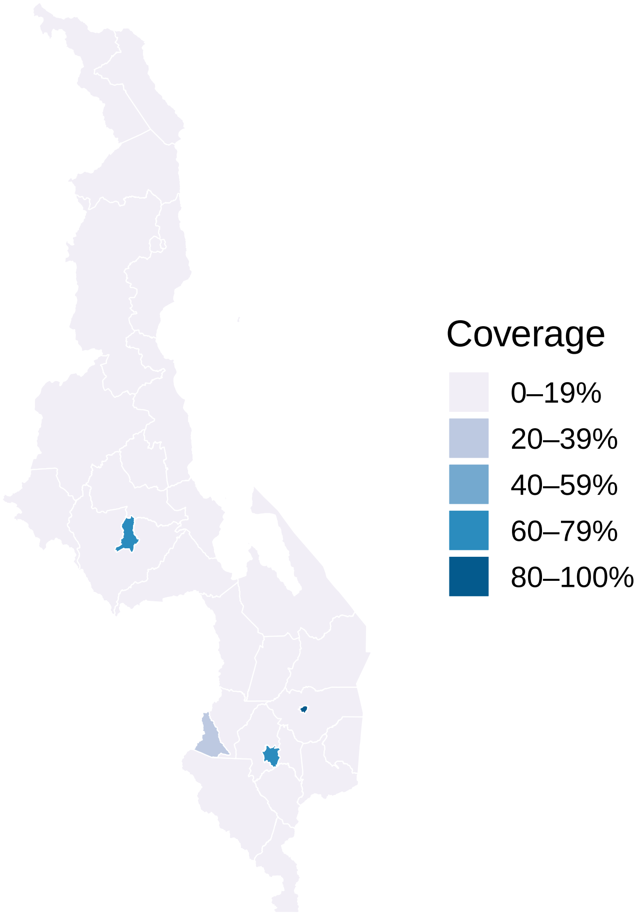

Management of convulsive seizures and epilepsy

Basic management of epilepsy

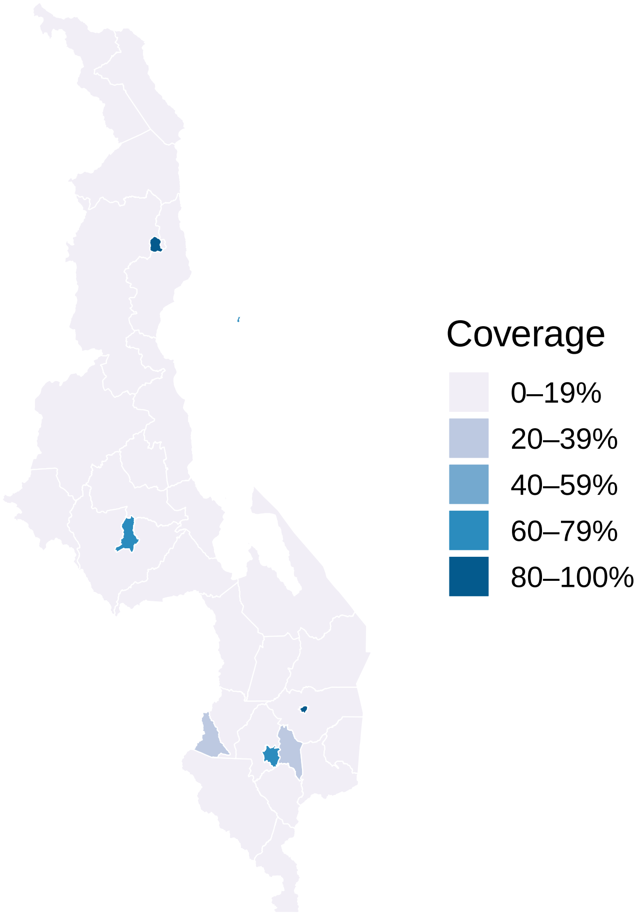

Management of convulsive seizures and epilepsy

Basic management of epilepsy

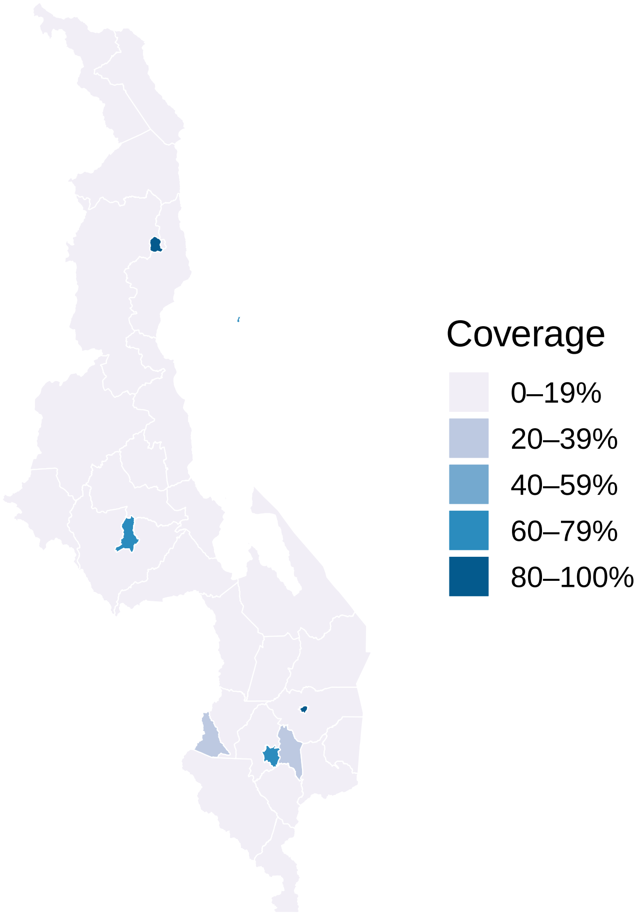

Management of migraine

Management of non−responders with migraine

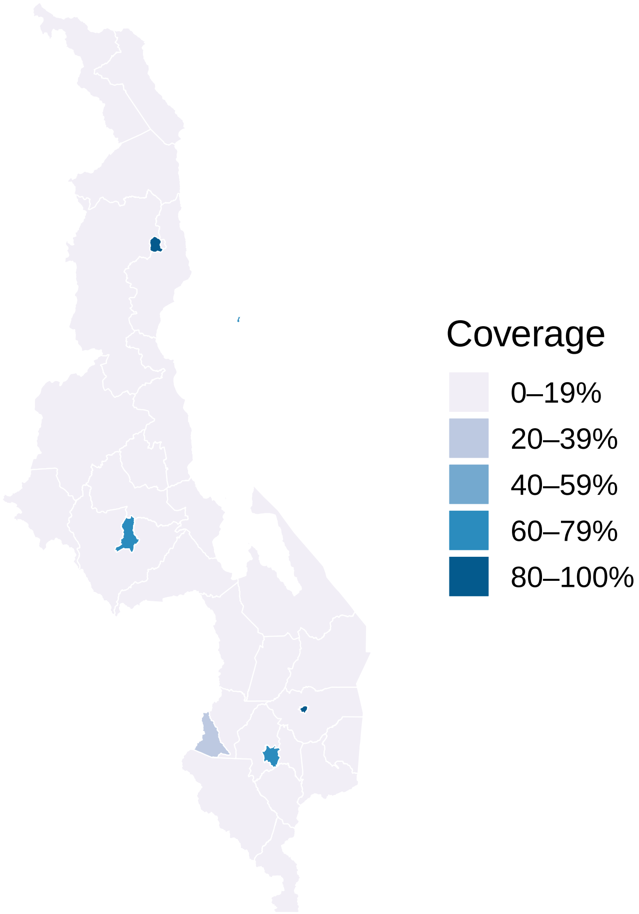

Management of FGM

Management of FGM

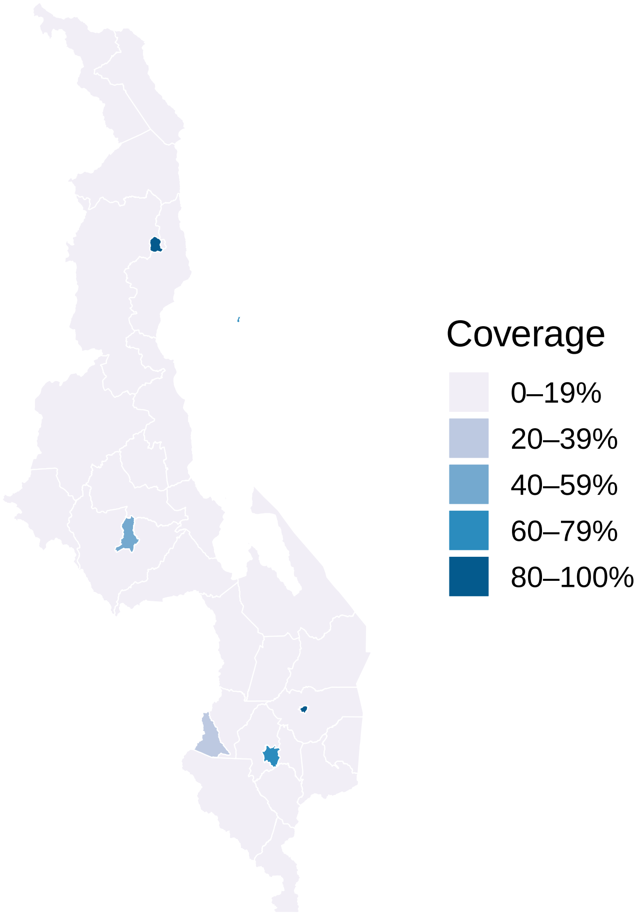

Antenatal care

Management of hyperglycemia in pregnancy

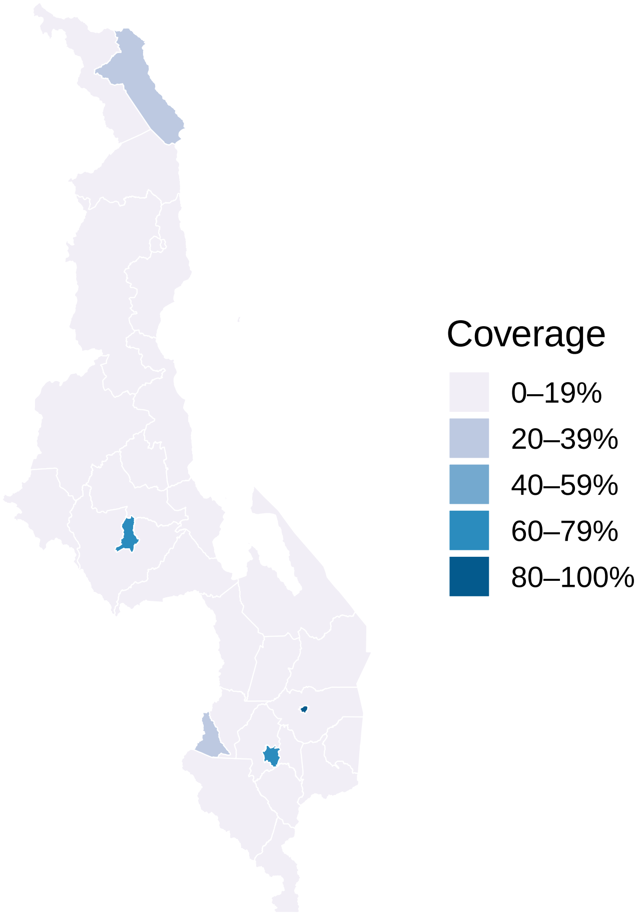

Antenatal care

Detection of growth restricted fetuses and fetal anomalies

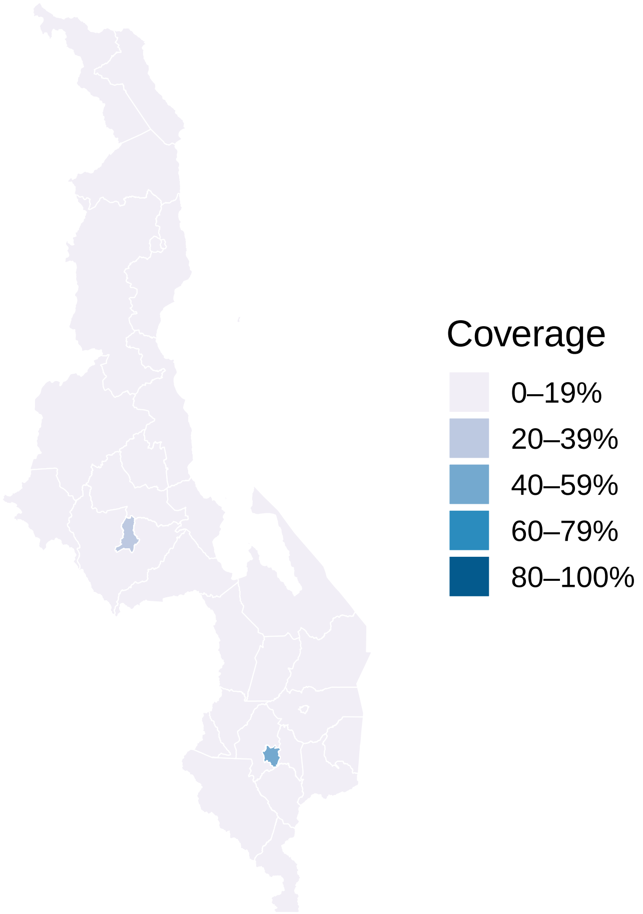

Antenatal care

Management of pregnancy−related hypertension

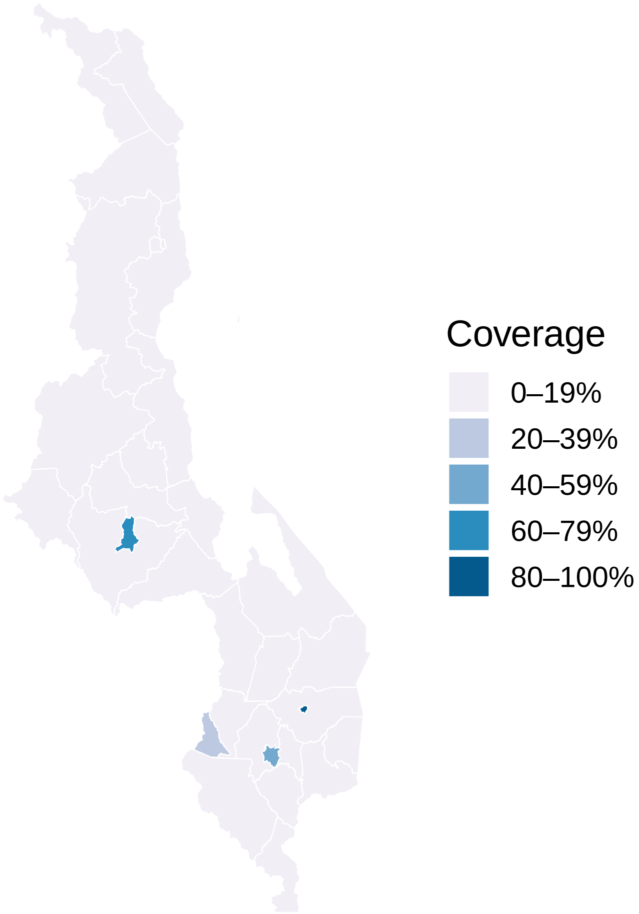

Antenatal care

Management of preterm delivery

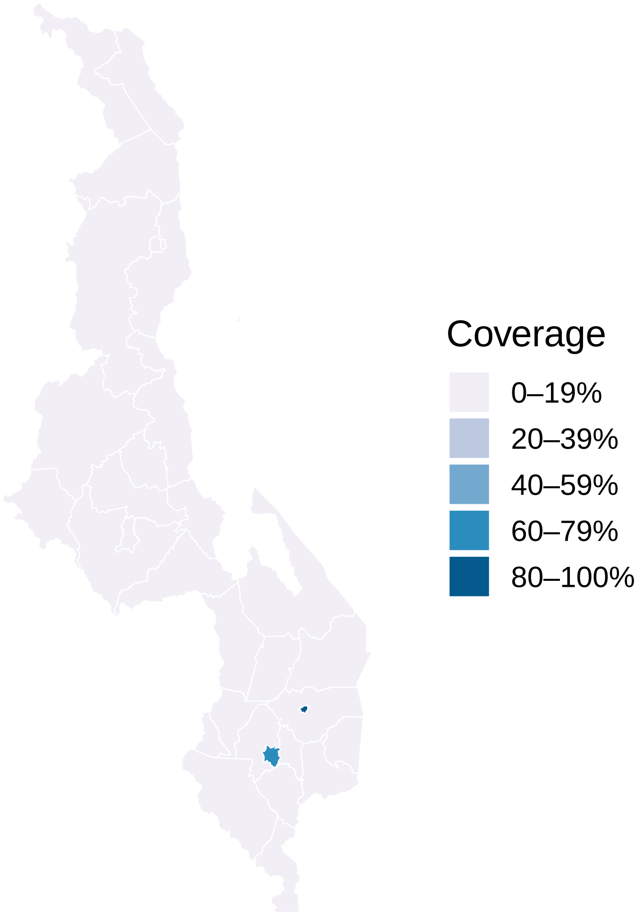

Antenatal care

Management of preterm delivery

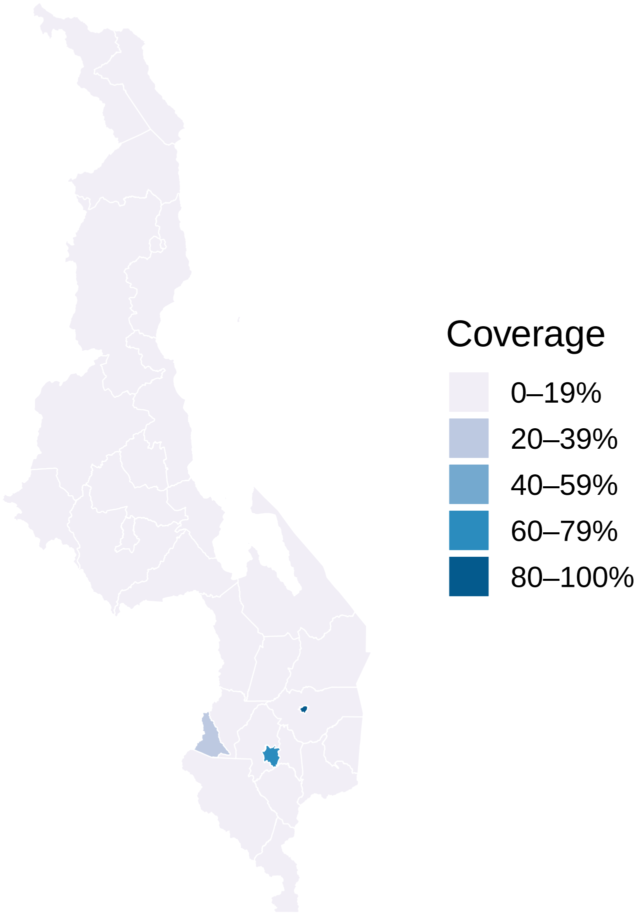

Safe delivery and management of labour complications

Induction of labour

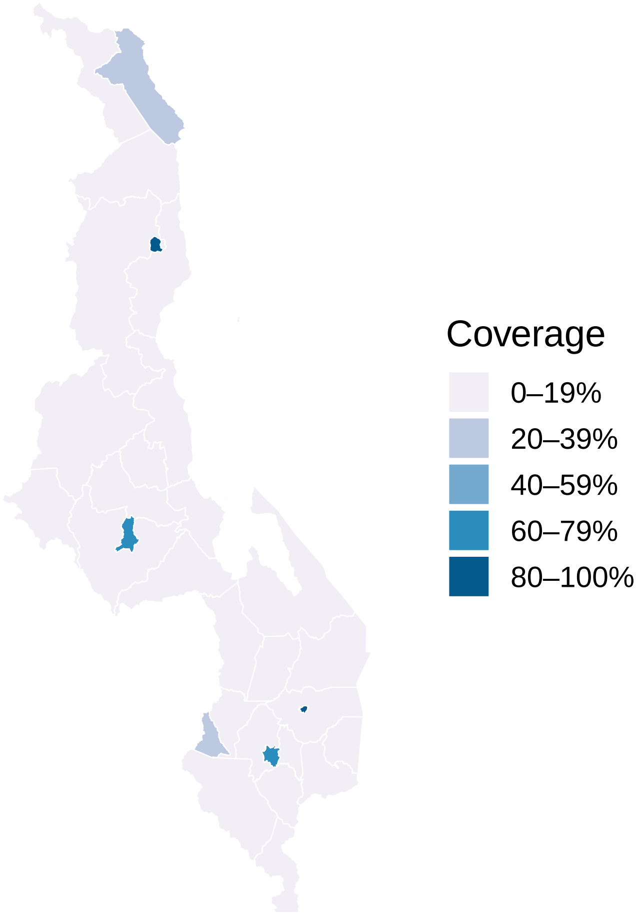

Safe delivery and management of labour complications

Management of postpartum haemorrhage

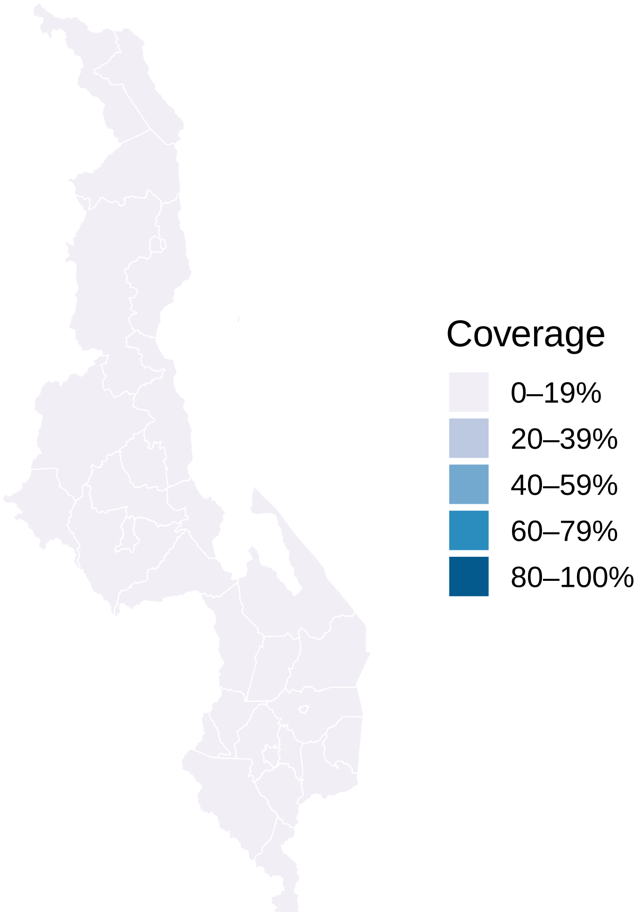

Routine care for postpartum women

Treatment of postpartum mental disorders

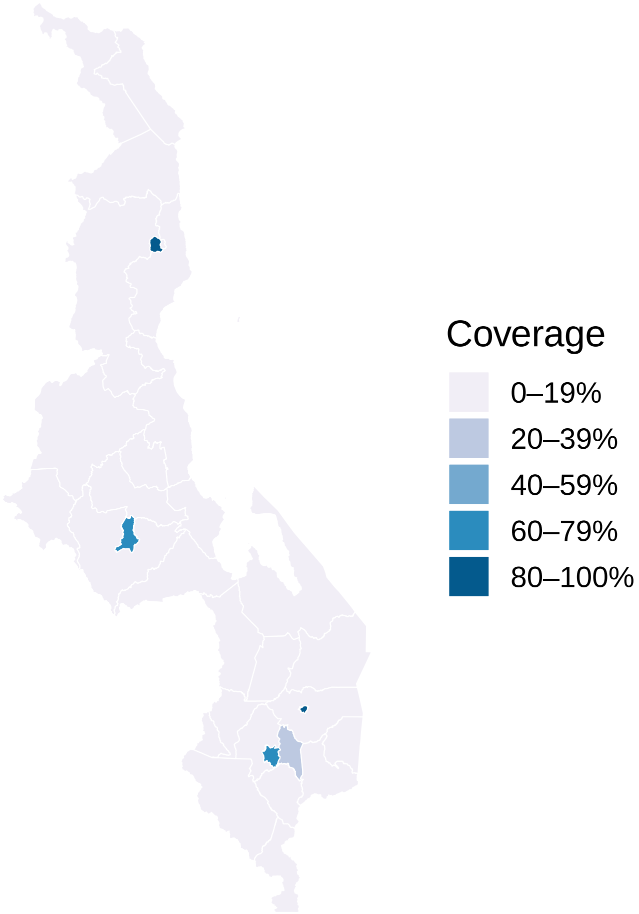

Treatment for neonatal complications

Early detection and treatment of neonatal sepsis and pneumonia

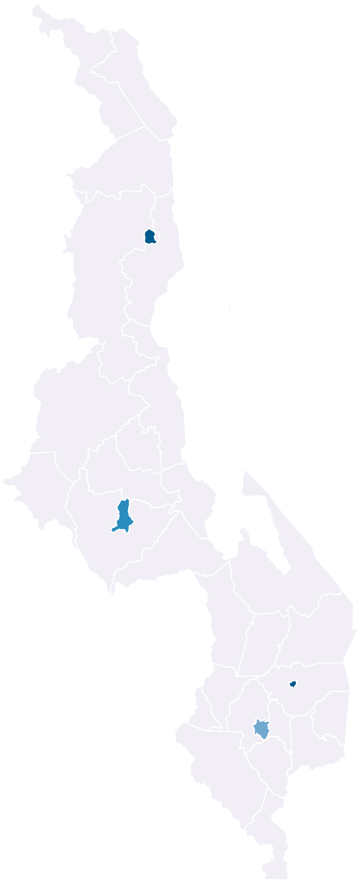

Coverage

- 0–19%
- 20–39%
- 40–59%
- 60–79%
- 80–100%

Management of extremity injuries

Management of upper extremity fractures

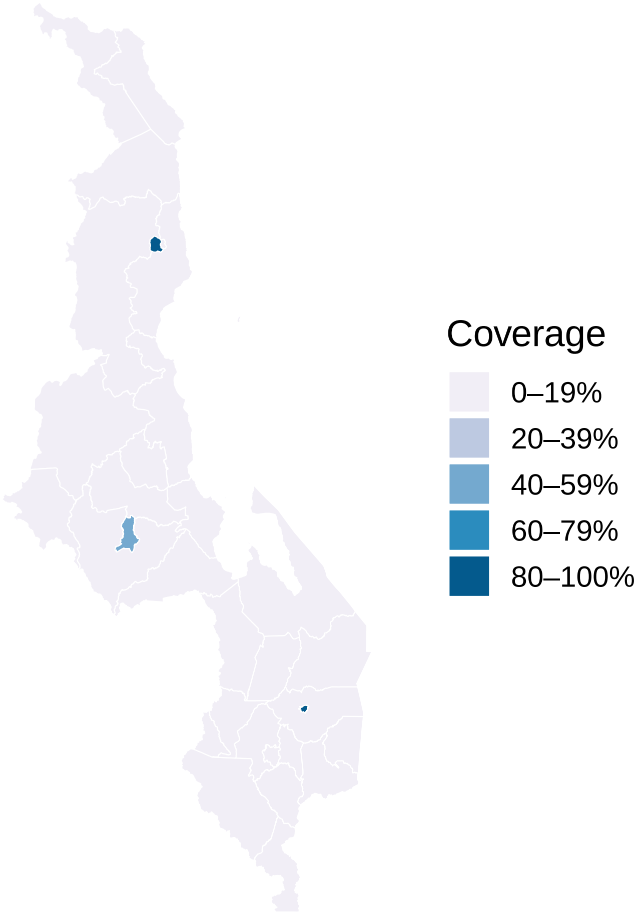

Management of extremity injuries

Management of lower extremity injuries

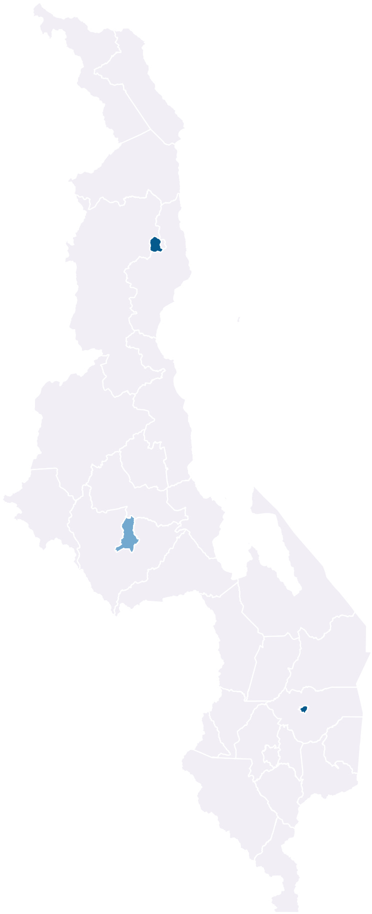

Coverage

- 0–19%
- 20–39%
- 40–59%
- 60–79%
- 80–100%

District level coverage
Specialized and Tertiary Care

Treatment of brain abscess and encephalitis

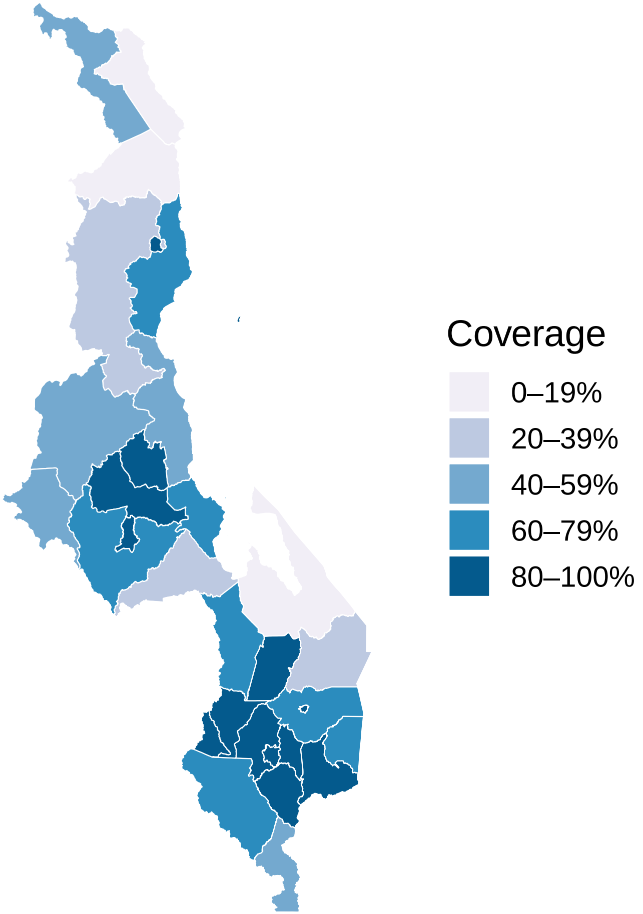

Treatment of meningitis

Treatment of meningitis, children

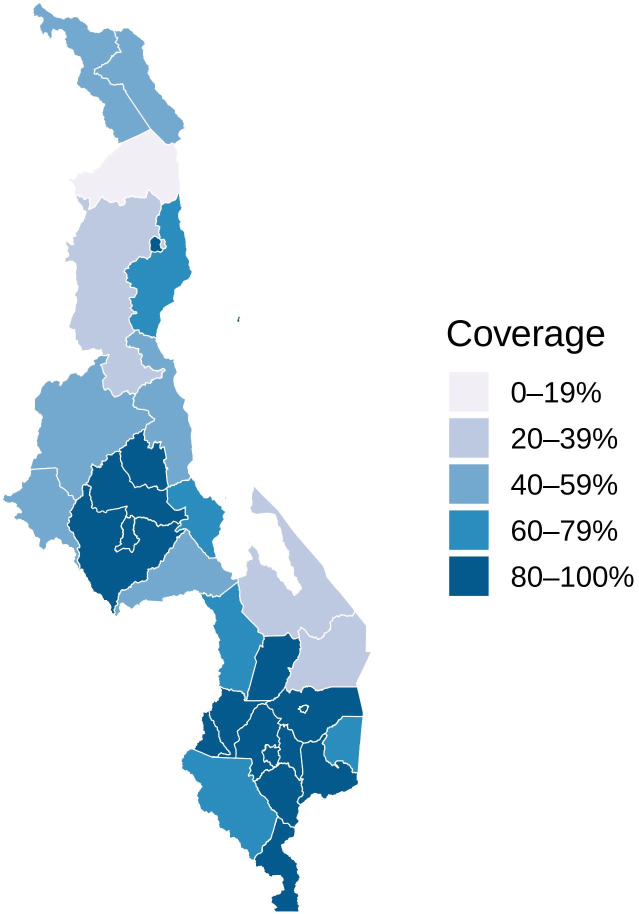

Treatment of meningitis

Treatment of meningitis, adults

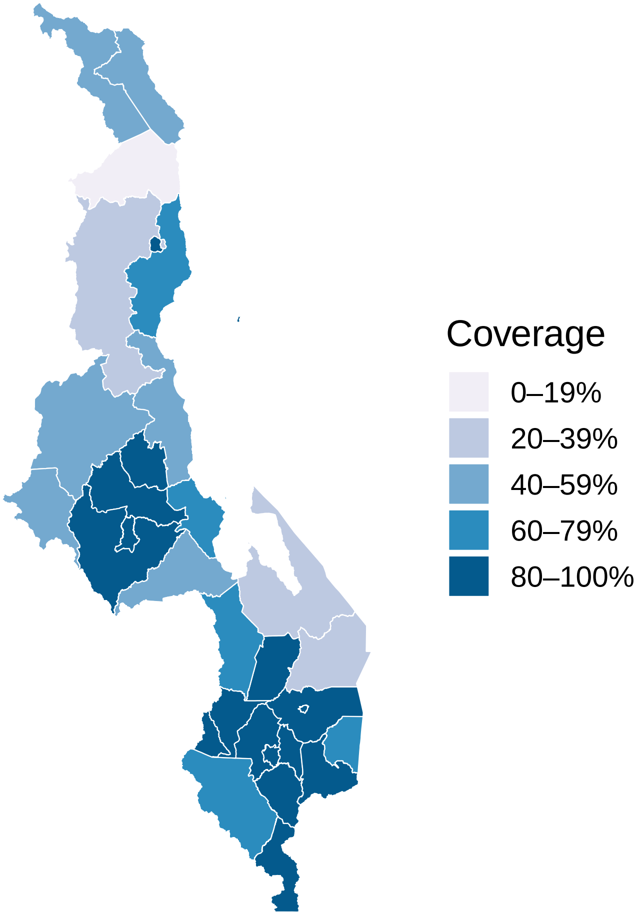

Management of septic arthritis

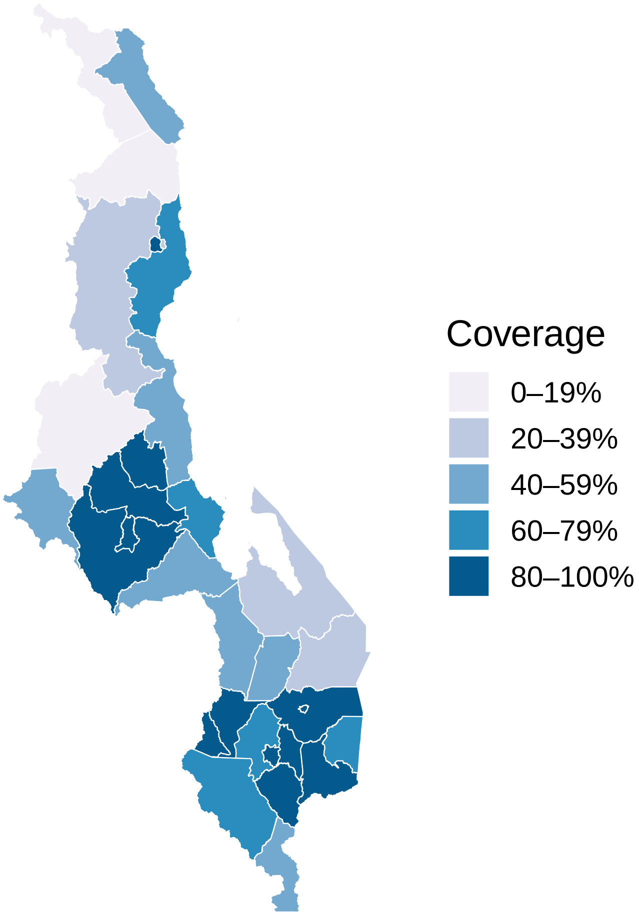

Surgery of anorectal malformations

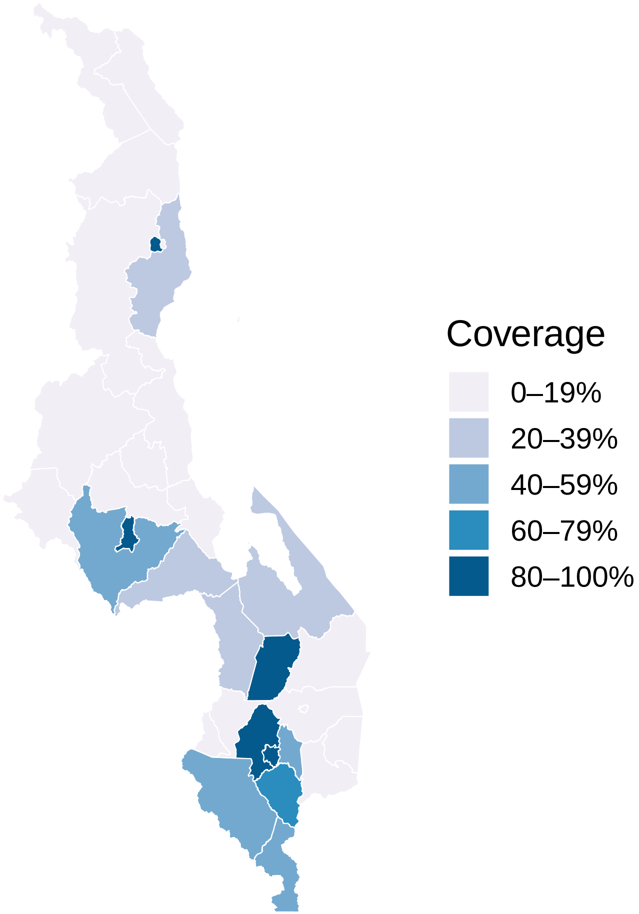

Surgical repair for congenital talipes

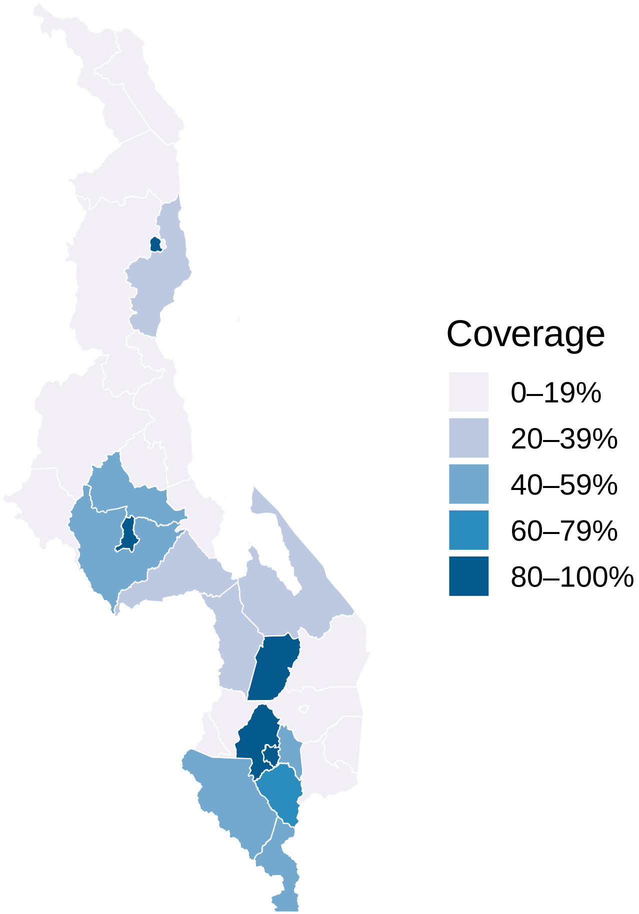

Management of cleft lip and/or cleft palate

Surgical repair of cleft lip and/or cleft palate

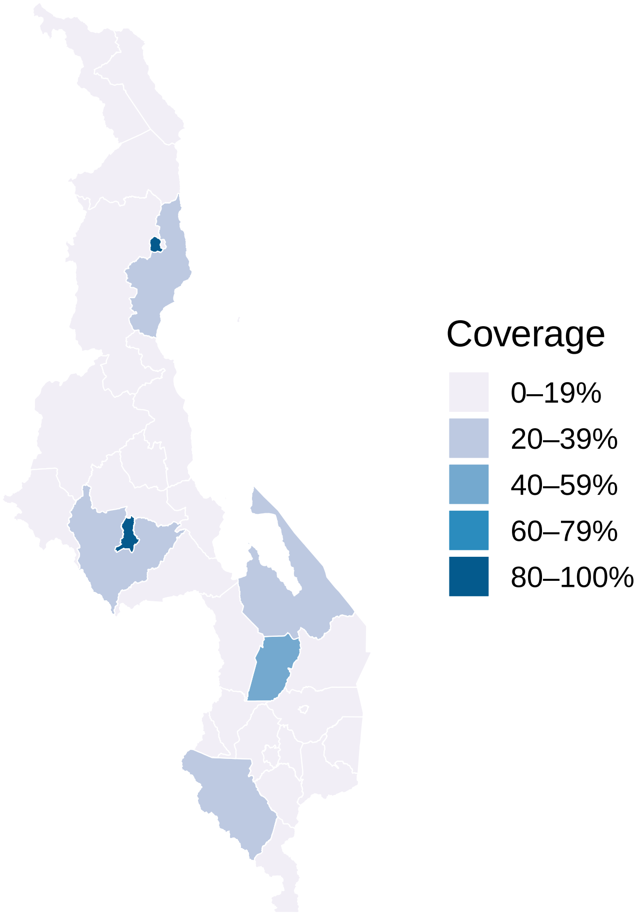

Management of appendicitis

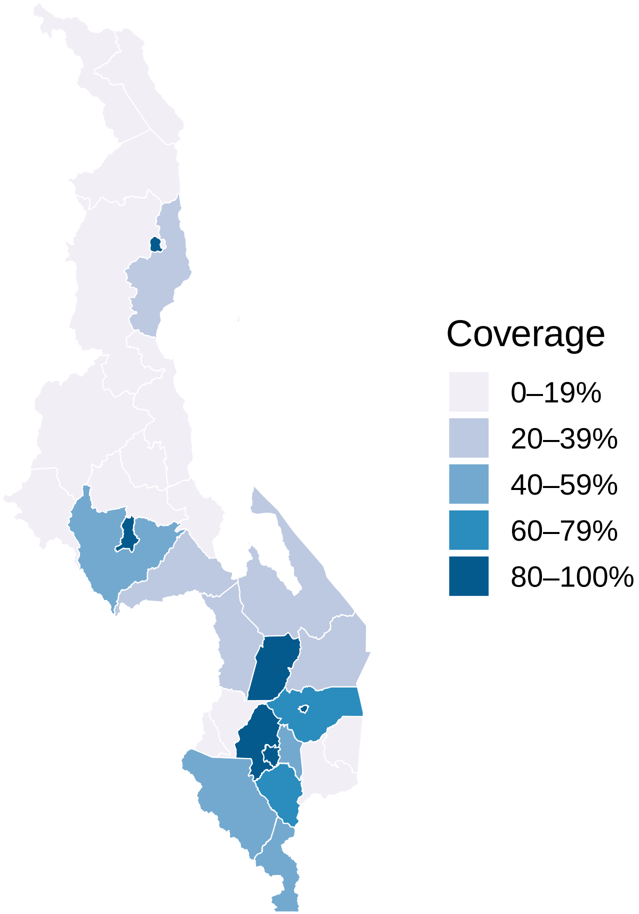

Management of ileus and intestinal obstruction

General management of ileus and intestinal obstruction

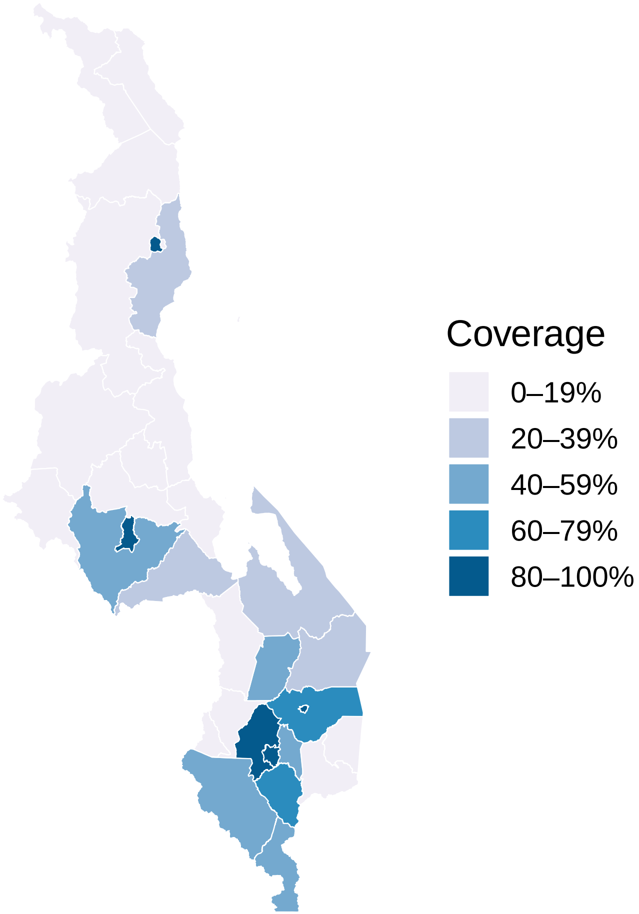

Management of ileus and intestinal obstruction

Surgical procedures for bowel obstruction

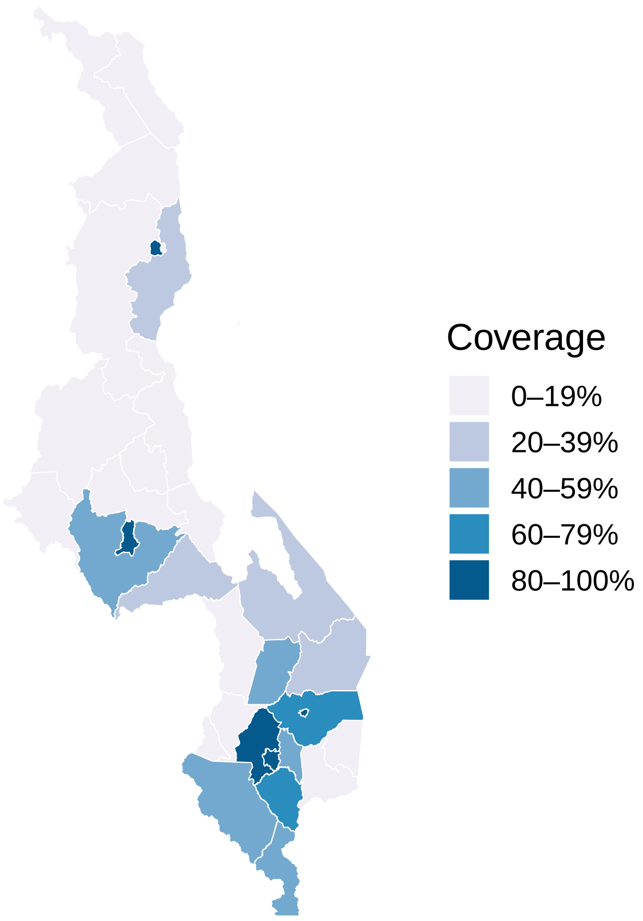

Management of hernias (inguinal, femoral, abdominal)

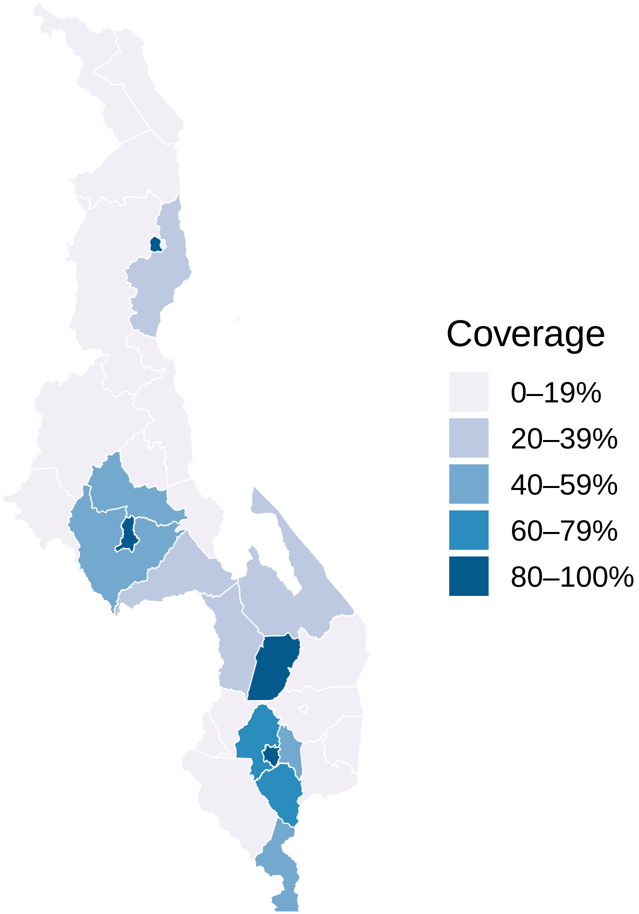

Management of gallbladder and biliary diseases

Removal of gallbladder

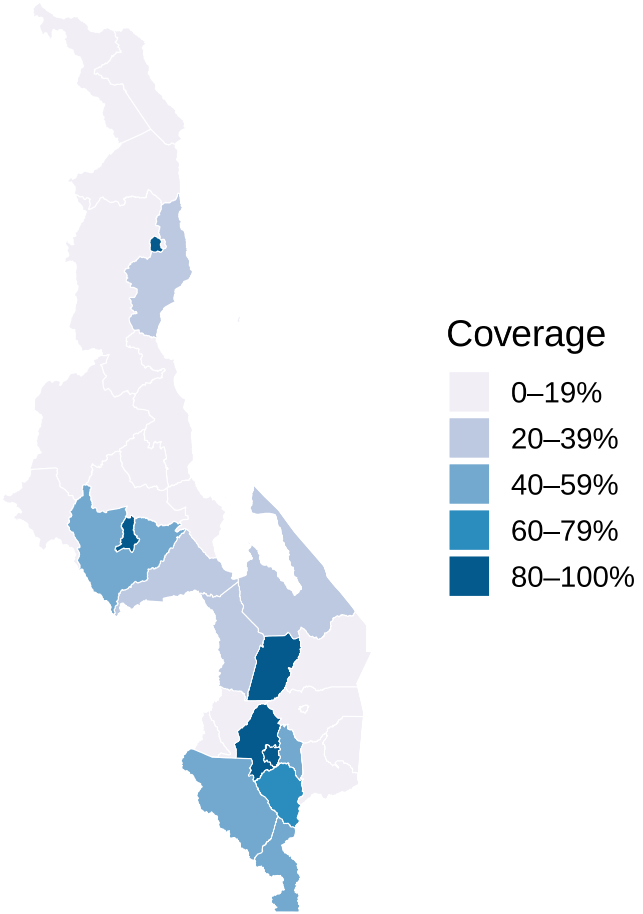

Management of gastrointestinal bleeding

Repair of ulcer perforations in GI system

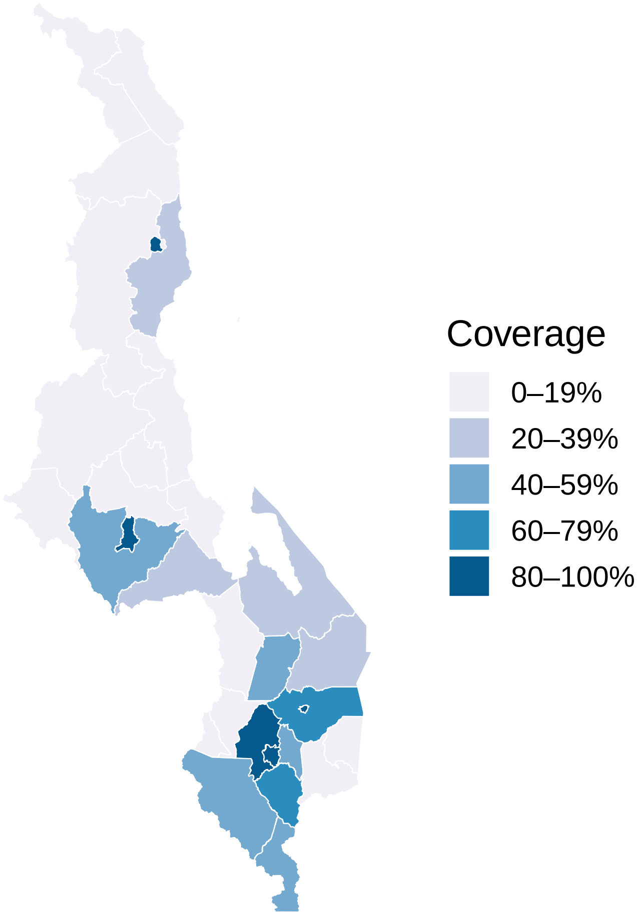

Management of gastrointestinal bleeding

Repair of ileal perforation due to typhoid

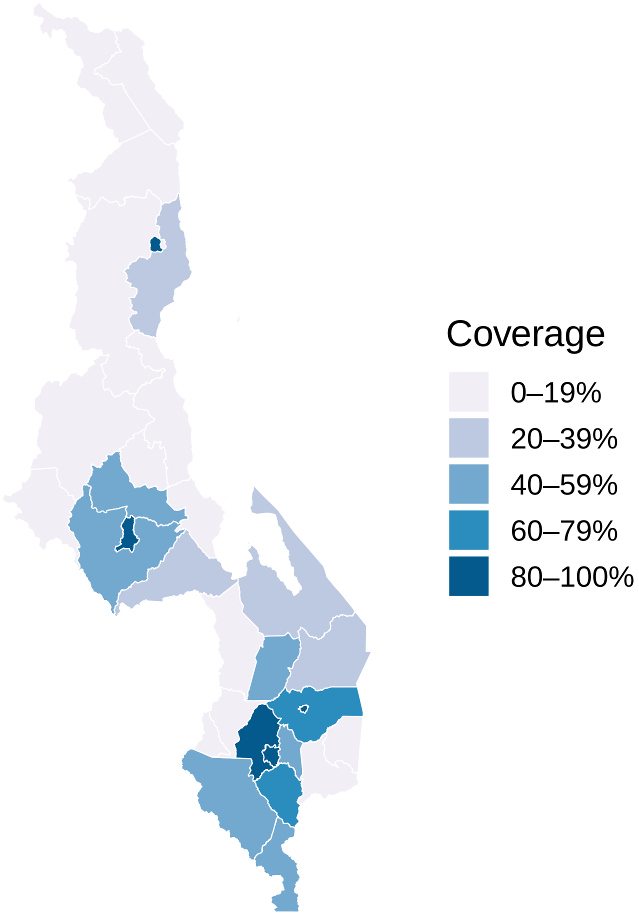

Management of acute ischemic heart disease

Treatment of acute coronary syndromes

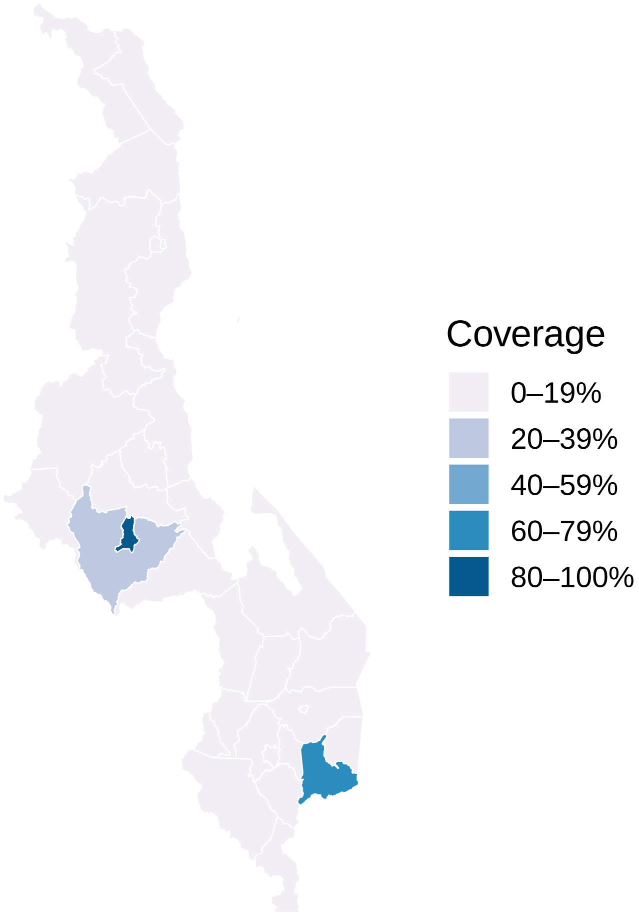

Management of acute heart failure

Management of acute heart failure with diuretics, oxygen, afterload reduction, medication optimization

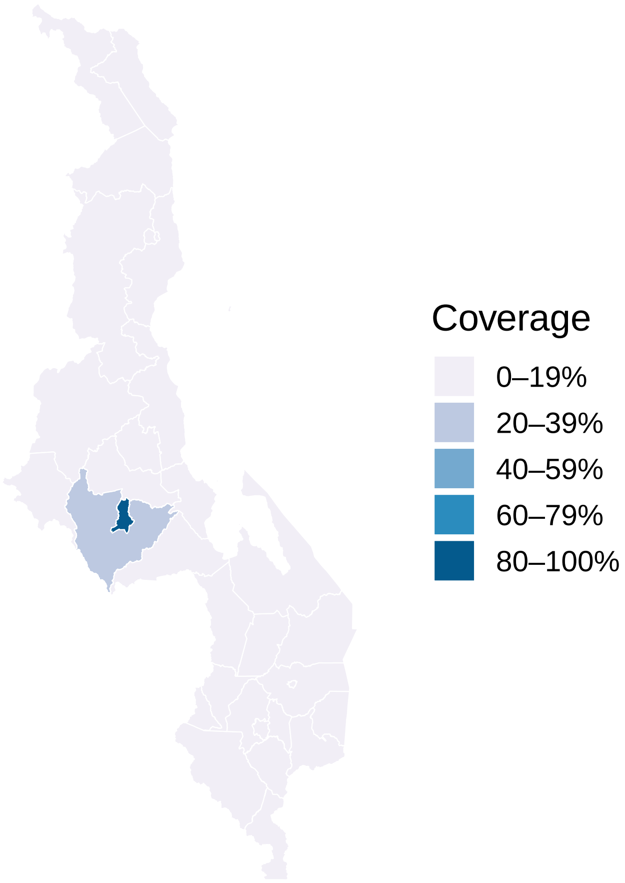

Family planning

Female sterilization

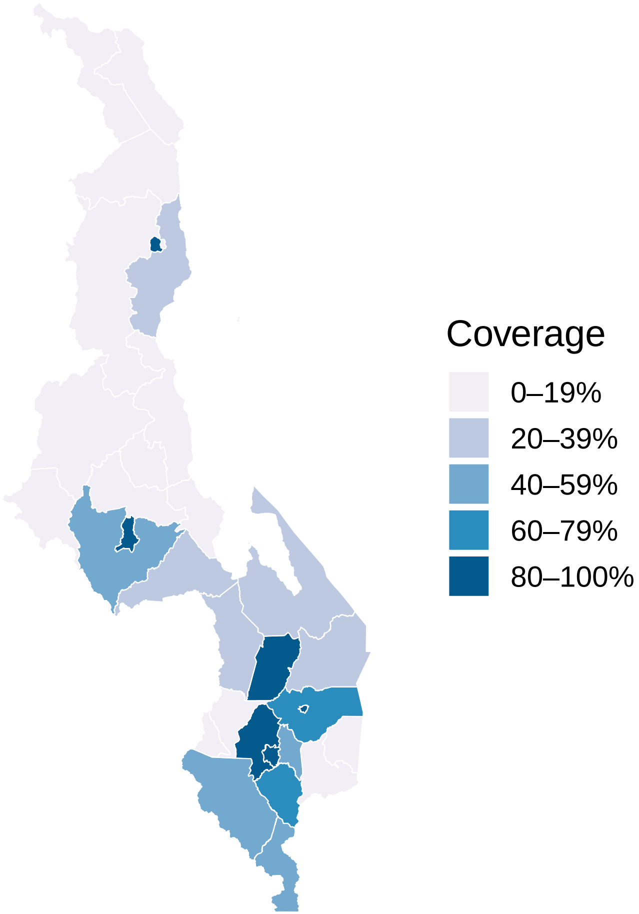

Induced abortion

Dilatation and evacuation

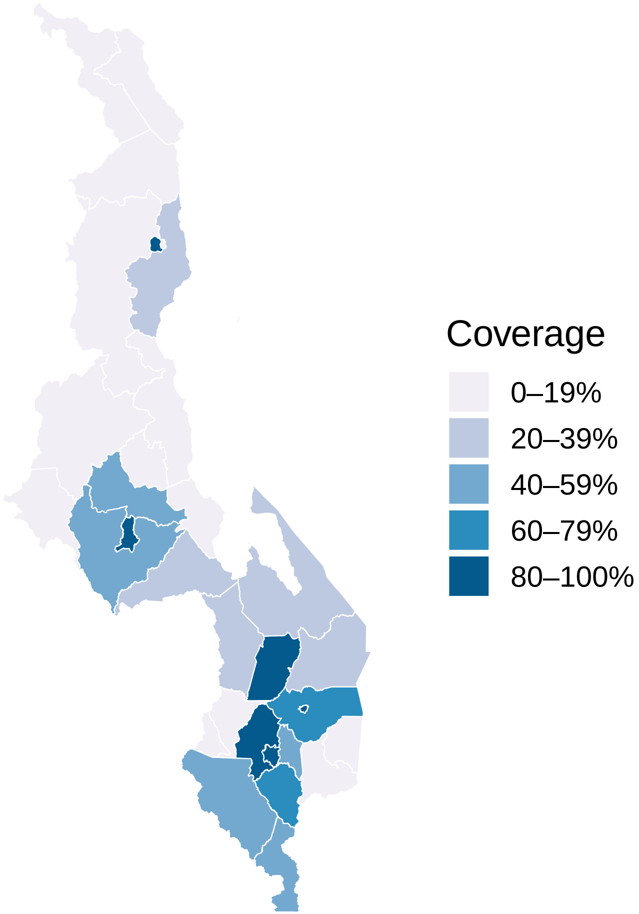

Induced abortion

Oral prostaglandins and progesterone receptor antagonists for abortion

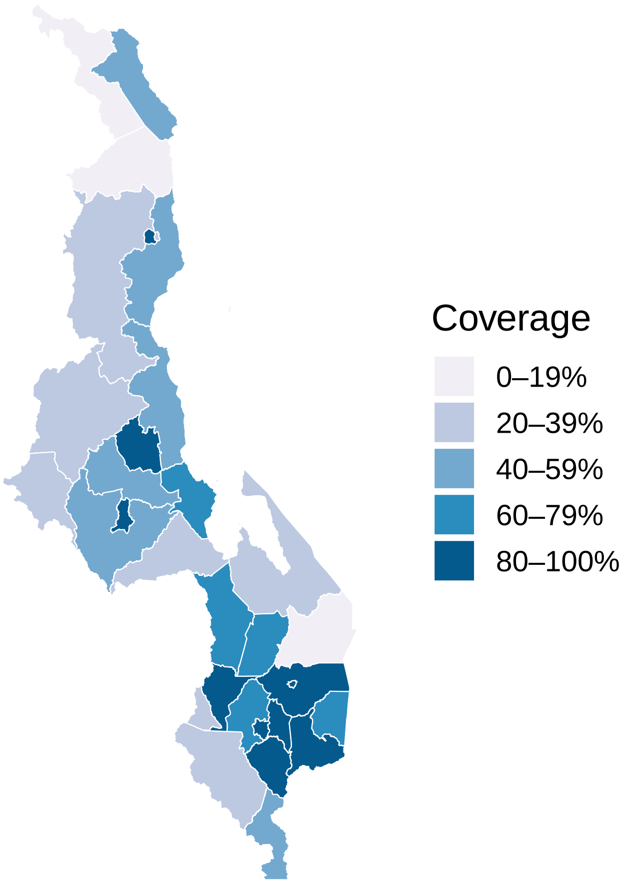

Care for complications of abortion

Removal of retained products following miscarriage, incomplete abortion or conception

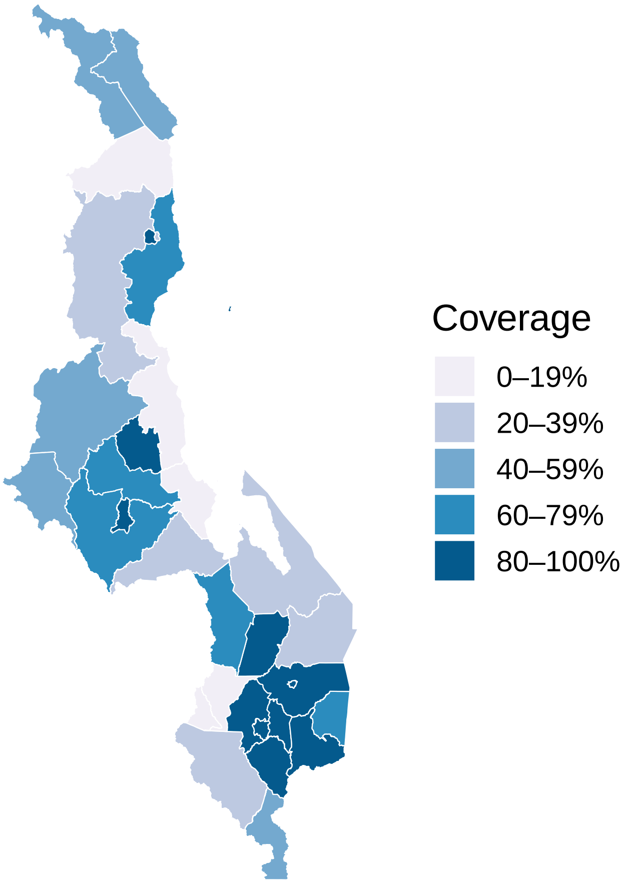

Care for complications of abortion

Management of post abortion complications (sepsis, lacerations)

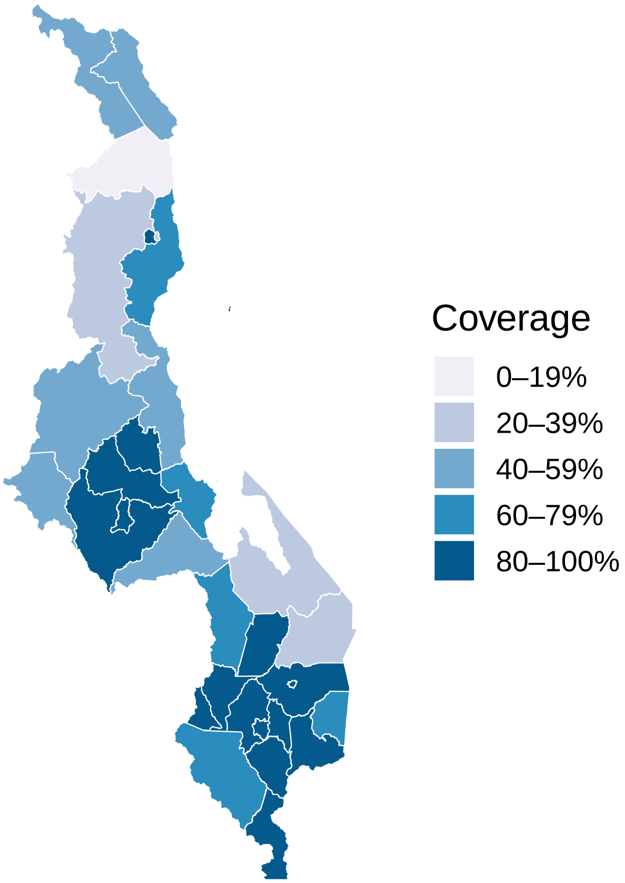

Safe delivery and management of labour complications

Safe delivery

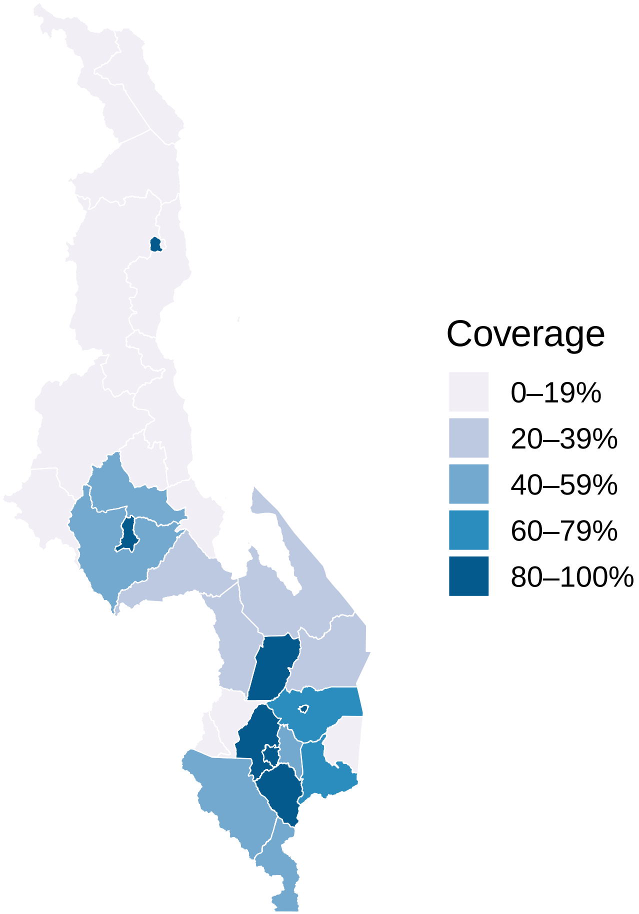

Safe delivery and management of labour complications

Management of postpartum haemorrhage

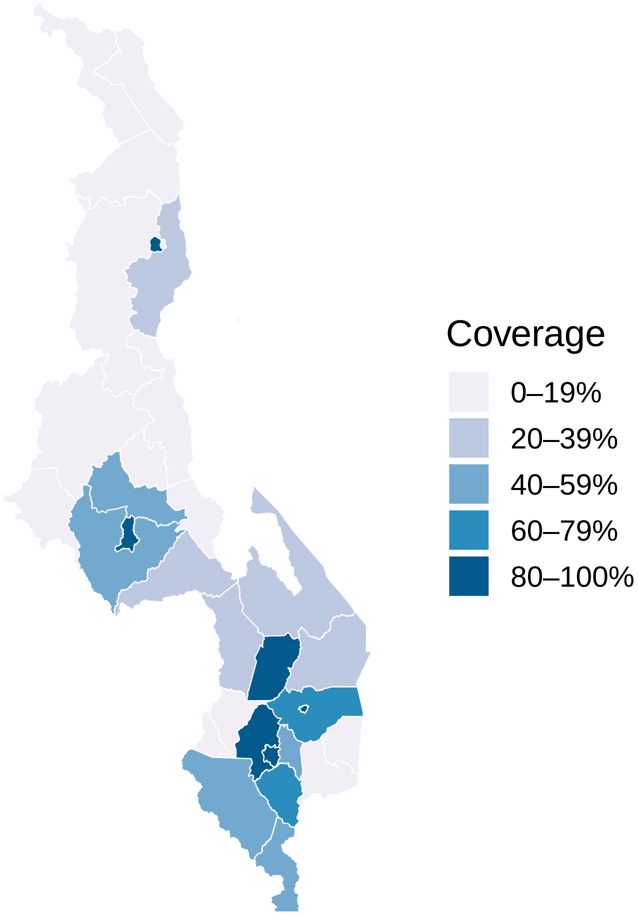

Management of burns

Basic skin grafting

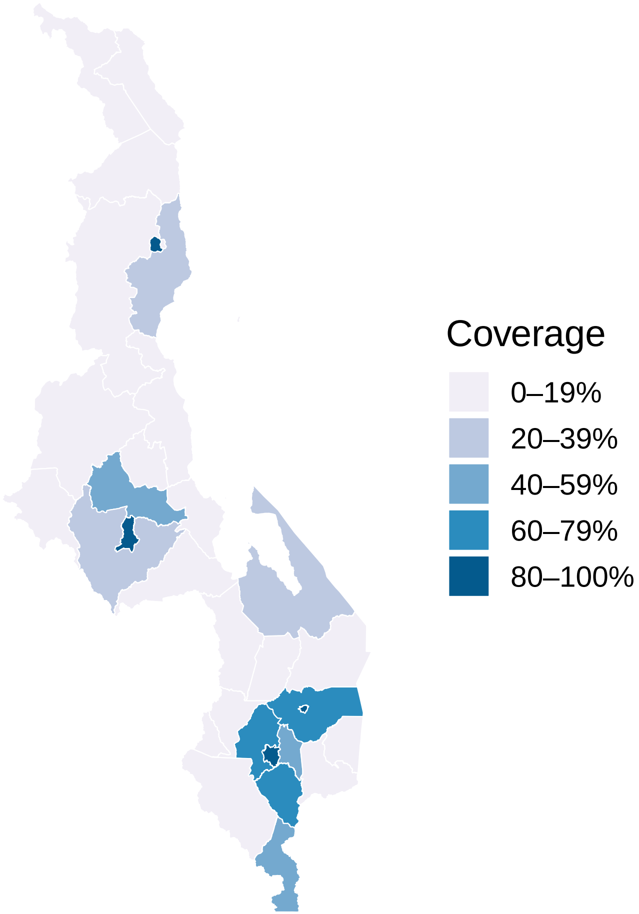

Management of thoracoabdominal injury

Trauma laparotomy and Tube thoracostomy

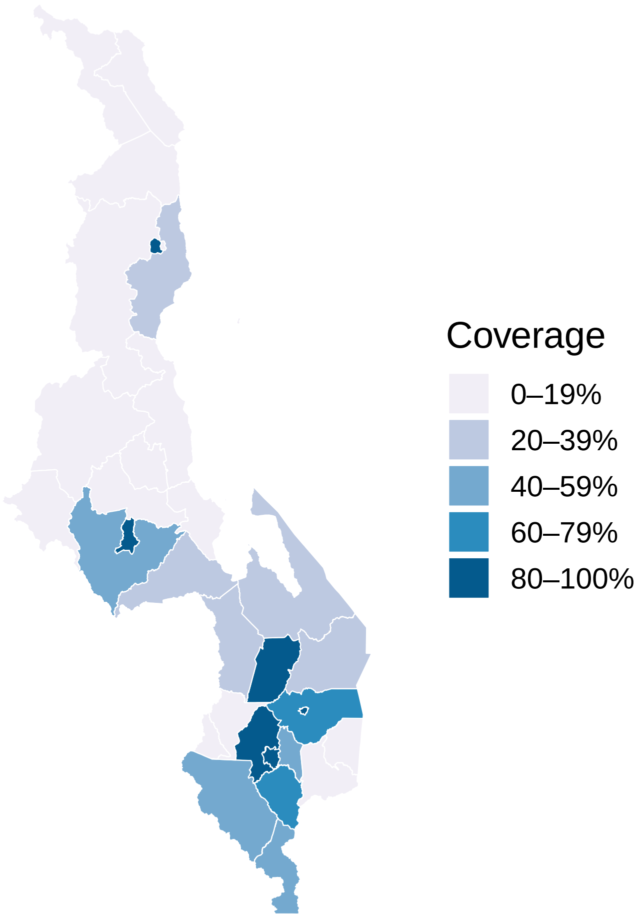

Management of head and face injury

Acute intracranial pressure relief

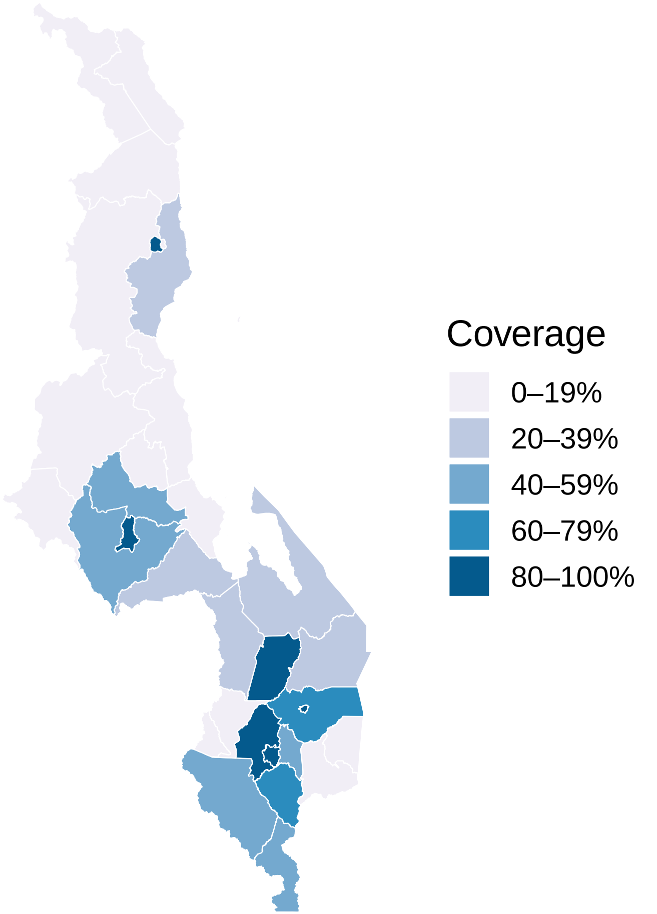

Supplement: Supplementary file 3 — Supplementary File 2 [file 44401_2026_81_MOESM3_ESM.pdf]
